# Supplementary material for: Exercise and incidence of myocardial infarction, stroke, hypertension, type 2 diabetes and site-specific cancers: prospective cohort study of 257 854 adults in South Korea
Source: BMJ Open. 2019 Mar 13;9(3):e025590. doi: 10.1136/bmjopen-2018-025590 (PMC6430026; doi:10.1136/bmjopen-2018-025590)
Supplement: Supplementary data [file bmjopen-2018-025590supp001.pdf]

Supplementary Table 1. Definitions for classification of incident disease outcomes

Incident disease outcome	Definition
Myocardial infarction	ICD 10 codes I21-I23
Stroke	ICD 10 codes I60-I64
Hypertension	ICD 10 codes I10, I11 and I15, systolic blood pressure ≥140 mm Hg, diastolic blood pressure ≥90 mm Hg, or physician diagnosis
Type 2 diabetes	ICD 10 codes E11, fasting glucose ≥126 mg/dL, or physician diagnosis
Stomach cancer	ICD 10 codes C16
Colon cancer	ICD 10 codes C18
Rectum cancer	ICD 10 codes C20
Lung cancer	ICD 10 codes C34
Liver cancer	ICD 10 codes C22
Pancreas cancer	ICD 10 codes C25
Head & neck cancer	ICD 10 codes C00-C06, C09-C14 and C32
Kidney cancer	ICD 10 codes C64
Gallbladder cancer	ICD 10 codes C23
Esophagus cancer	ICD 10 codes C15

Abbreviations: ICD – International Classification of Disease

Supplementary Table 2. Participants' characteristics at 1st, 2nd, 3rd, 4th, 5th and 6th repeat-visit

Repeat visit	Variables	All (n=215,295)	Exercise frequency				
1st			None (n=112,156)	1-2 times/week (n=57,777)	3-4 times/week (n=25,390)	5-6 times/week (n= 6,443)	Almost everyday (n=13,529)
	Sex, %						
	Men	51.1%	45.0%	61.8%	55.3%	50.6%	48.8%
	Women	48.9%	55.0%	38.2%	44.7%	49.4%	51.2%
	Age, years	52.7 (8.6)	53.6 (9.2)	50.9 (7.4)	51.3 (7.5)	52.3 (8.0)	55.6 (9.1)
	Body Mass Index, kg^2/m	23.5 (2.8)	23.4 (2.9)	23.6 (2.7)	23.7 (2.6)	23.6 (2.6)	23.7 (2.7)
	Systolic blood pressure, mm Hg	120.5 (14.4)	120.6 (14.7)	120.4 (13.9)	120.0 (13.9)	119.6 (13.9)	121.4 (14.6)
	Diastolic blood pressure, mm Hg	75.7 (9.9)	75.6 (9.9)	75.9 (9.8)	75.5 (9.8)	75.2 (9.8)	75.7 (9.8)
	Fasting glucose levels, mg/dL	92.6 (18.5)	92.6 (18.8)	92.7 (17.4)	92.4 (17.8)	92.8 (21.0)	93.3 (20.2)
	Total cholesterol, mg/dL	197.3 (36.2)	197.0 (36.8)	197.3 (35.6)	197.9 (35.1)	197.4 (35.9)	197.8 (36.7)
	Family history of heart disease, stroke or hypertension, %	12.6%	10.7%	14.2%	16.7%	16.7%	12.0%
	Family history of cancer, %	14.3%	12.7%	15.5%	17.4%	19.2%	14.5%
	Family history of diabetes, %	6.0%	5.0%	6.7%	8.2%	9.0%	5.8%
	Smoking status, %						
	Never	70.7%	74.8%	62.4%	68.9%	71.7%	74.8%
	Previously	8.6%	5.8%	11.9%	12.4%	11.9%	8.8%
	Currently	20.8%	19.4%	25.7%	18.7%	16.3%	16.4%
	Alcohol Consumption, %						
	Never	59.8%	66.9%	49.2%	52.7%	54.9%	61.8%
	2-3 times/month	15.6%	12.7%	20.5%	18.6%	18.2%	12.8%
	1-2 times/week	15.8%	11.8%	21.7%	19.9%	17.0%	14.8%
	≥3 times/week	8.8%	8.7%	8.6%	8.7%	10.0%	10.6%

Supplementary Table 2. Participants' characteristics at 1st, 2nd, 3rd, 4th, 5th and 6th repeat-visit (cont.)

Repeat visit	Variables	All (n=150,845)	Exercise frequency				
2nd			None (n=73,660)	1-2 times/week (n=44,211)	3-4 times/week (n=19,593)	5-6 times/week (n=4,771)	Almost everyday (n= 8,610)
	Sex, %						
	Men	54.2%	47.0%	64.6%	57.9%	53.8%	53.5%
	Women	45.8%	53.0%	35.4%	42.1%	46.2%	46.5%
	Age, years	53.3 (8.3)	54.2 (8.9)	51.7 (7.2)	52.3 (7.4)	53.4 (7.8)	56.5 (9.1)
	Body Mass Index, kg^2/m	23.5 (2.7)	23.4 (2.8)	23.6 (2.6)	23.7 (2.6)	23.6 (2.6)	23.6 (2.7)
	Systolic blood pressure, mm Hg	120.9 (14.1)	121.0 (14.5)	120.7 (13.7)	120.4 (13.6)	120.3 (13.7)	122.0 (14.5)
	Diastolic blood pressure, mm Hg	75.8 (9.6)	75.8 (9.7)	76.0 (9.5)	75.5 (9.4)	75.4 (9.4)	76.0 (9.8)
	Fasting glucose levels, mg/dL	93.2 (16.9)	93.1 (17.4)	93.5 (17.0)	93.1 (15.5)	93.5 (15.9)	93.7 (16.2)
	Total cholesterol, mg/dL	197.6 (35.9)	197.7 (36.5)	197.4 (35.4)	197.8 (35.2)	196.7 (34.9)	198.1 (36.5)
	Family history of heart disease, stroke or hypertension, %	13.4%	11.2%	14.6%	17.5%	18.3%	13.1%
	Family history of cancer, %	14.9%	12.9%	16.4%	17.4%	19.7%	15.4%
	Family history of diabetes, %	6.3%	5.2%	7.1%	8.2%	9.0%	6.1%
	Smoking status, %						
	Never	71.0%	76.5%	62.3%	68.7%	71.2%	74.2%
	Previously	9.0%	5.5%	12.5%	13.1%	13.1%	9.4%
	Currently	20.0%	18.0%	25.3%	18.2%	15.8%	16.5%
	Alcohol Consumption, %						
	Never	59.0%	67.6%	48.1%	51.9%	54.1%	60.0%
	2-3 times/month	16.1%	12.6%	20.9%	19.0%	18.9%	13.6%
	1-2 times/week	16.7%	12.1%	22.8%	20.7%	17.5%	15.8%
	≥3 times/week	8.1%	7.8%	8.2%	8.3%	9.5%	10.7%

Supplementary Table 2. Participants' characteristics at 1st, 2nd, 3rd, 4th, 5th and 6th repeat-visit (cont.)

Repeat visit	Variables	All (n=80,639)	Exercise frequency				
3rd			None (n= 36,123)	1-2 times/week (n=26,505)	3-4 times/week (n=11,432)	5-6 times/week (n=2,391)	Almost everyday (n=4,188)
	Sex, %						
	Men	63.1%	53.9%	73.0%	68.6%	64.5%	63.4%
	Women	36.9%	46.1%	27.0%	31.4%	35.6%	36.7%
	Age, years	52.3 (7.2)	52.9 (7.8)	51.2 (6.2)	51.9 (6.6)	52.8 (7.0)	55.2 (8.4)
	Body Mass Index, kg^2/m	23.5 (2.7)	23.3 (2.8)	23.6 (2.6)	23.7 (2.5)	23.7 (2.6)	23.7 (2.7)
	Systolic blood pressure, mm Hg	121.1 (13.6)	120.9 (13.9)	120.9 (13.4)	121.0 (13.2)	120.6 (13.3)	122.0 (14.0)
	Diastolic blood pressure, mm Hg	76.1 (9.4)	76.0 (9.5)	76.4 (9.4)	76.1 (9.1)	75.6 (9.5)	76.3 (9.6)
	Fasting glucose levels, mg/dL	93.7 (16.8)	93.3 (17.1)	94.1 (16.7)	93.9 (15.9)	94.1 (16.5)	94.5 (17.0)
	Total cholesterol, mg/dL	197.1 (35.2)	197.1 (35.5)	197.4 (34.8)	196.9 (34.7)	196.5 (35.2)	196.7 (35.4)
	Family history of heart disease, stroke or hypertension, %	13.5%	11.4%	14.8%	16.7%	18.6%	11.7%
	Family history of cancer, %	14.8%	13.0%	15.8%	17.8%	18.9%	14.7%
	Family history of diabetes, %	6.4%	5.3%	7.0%	8.0%	9.5%	6.2%
	Smoking status, %						
	Never	66.6%	73.8%	57.6%	63.9%	65.5%	68.8%
	Previously	10.7%	6.1%	14.0%	15.5%	16.6%	12.4%
	Currently	22.8%	20.2%	28.4%	20.6%	17.9%	18.7%
	Alcohol Consumption, %						
	Never	53.6%	64.2%	42.5%	47.2%	47.4%	53.9%
	2-3 times/month	18.5%	14.0%	23.7%	20.8%	21.4%	15.8%
	1-2 times/week	19.5%	14.0%	25.6 %	22.8%	20.7%	18.8%
	≥3 times/week	8.4%	7.8%	8.3%	9.2%	10.5%	11.5%

Supplementary Table 2. Participants' characteristics at 1st, 2nd, 3rd, 4th, 5th and 6th repeat-visit (cont.)

Repeat visit	Variables	All (n=40,910)	Exercise frequency				
4th			None (n=17,678)	1-2 times/week (n=14,624)	3-4 times/week (n= 5,685)	5-6 times/week (n=1,094)	Almost everyday (n=1,829)
	Sex, %						
	Men	72.3%	61.6%	81.1%	81.5%	78.2%	73.9%
	Women	27.7%	38.4%	18.9%	18.5%	21.9%	26.1%
	Age, years	50.9 (5.4)	51.2 (5.5)	50.6 (5.1)	50.7 (5.3)	51.0 (5.6)	52.6 (6.3)
	Body Mass Index, kg^2/m	23.5 (2.7)	23.3 (2.7)	23.7 (2.6)	23.8 (2.6)	23.8 (2.5)	23.7 (2.6)
	Systolic blood pressure, mm Hg	121.4 (13.3)	120.9 (13.5)	121.7 (13.0)	121.5 (12.8)	121.5 (13.1)	122.7 (13.6)
	Diastolic blood pressure, mm Hg	76.6 (9.2)	76.2 (9.3)	77.0 (9.2)	76.8 (8.9)	76.8 (9.4)	77.2 (9.5)
	Fasting glucose levels, mg/dL	93.9 (17.8)	93.3 (17.9)	94.4 (17.9)	94.3 (17.1)	94.0 (16.0)	94.9 (18.8)
	Total cholesterol, mg/dL	196.8 (35.0)	197.2 (35.5)	196.7 (34.6)	196.6 (34.2)	195.0 (34.3)	196.5 (34.9)
	Family history of heart disease, stroke or hypertension, %	12.3%	10.4%	13.7%	14.2%	16.3%	11.6%
	Family history of cancer, %	13.8%	12.6%	14.7%	15.0%	16.3%	13.5%
	Family history of diabetes, %	5.7%	4.8%	6.2%	6.9%	7.5%	6.7%
	Smoking status, %						
	Never	60.6%	69.5%	51.9%	55.5%	57.3%	62.0%
	Previously	12.2%	6.9%	15.1%	18.7%	18.7%	15.7%
	Currently	27.2%	23.7%	33.0%	25.8%	24.0%	22.4%
	Alcohol Consumption, %						
	Never	48.1%	60.0%	38.0%	39.1%	39.9%	45.4%
	2-3 times/month	20.5%	15.5%	24.9%	24.3%	24.0%	19.7%
	1-2 times/week	22.4%	16.2%	28.4%	26.3%	22.6%	22.9%
	≥3 times/week	9.0%	8.2%	8.7%	10.3%	13.6%	12.0%

Supplementary Table 2. Participants' characteristics at 1st, 2nd, 3rd, 4th, 5th and 6th repeat-visit (cont.)

Repeat visit	Variables	All (n= 26,356)	Exercise frequency				
5th			None (n=10,771)	1-2 times/week (n=9,807)	3-4 times/week (n=3,884)	5-6 times/week (n=724)	Almost everyday (n=1,170)
	Sex, %						
	Men	73.7%	62.6%	81.6%	82.4%	81.2%	76.9%
	Women	26.3%	37.4%	18.4%	17.6%	18.8%	23.0%
	Age, years	51.1 (4.9)	51.4 (5.1)	50.8 (4.7)	51.0 (4.7)	51.4 (5.0)	52.1 (5.6)
	Body Mass Index, kg^2/m	23.5 (2.6)	23.3 (2.7)	23.7 (2.6)	23.8 (2.4)	23.7 (2.5)	23.8 (2.6)
	Systolic blood pressure, mm Hg	121.4 (13.0)	121.0 (13.4)	121.8 (12.9)	121.6 (12.6)	120.8 (12.9)	121.5 (12.7)
	Diastolic blood pressure, mm Hg	76.6 (9.1)	76.2 (9.1)	77.1 (9.0)	76.8 (8.8)	76.7 (8.6)	76.4 (9.0)
	Fasting glucose levels, mg/dL	94.3 (17.6)	93.7 (18.1)	94.6 (17.3)	94.7 (16.6)	94.6 (15.9)	95.4 (18.7)
	Total cholesterol, mg/dL	197.1 (34.5)	197.7 (34.8)	196.7 (34.2)	196.4 (33.8)	195.2 (34.2)	198.2 (37.0)
	Family history of heart disease, stroke or hypertension, %	12.6%	11.3%	13.4%	14.3%	13.8%	10.9%
	Family history of cancer, %	14.3%	13.2%	15.1%	14.9%	17.8%	13.5%
	Family history of diabetes, %	5.9%	4.9%	6.4%	6.7%	7.2%	8.6%
	Smoking status, %						
	Never	58.9%	68.1%	50.8%	54.4%	57.0%	58.5%
	Previously	13.1%	7.1%	16.0%	19.3%	19.8%	19.7%
	Currently	28.0%	24.8%	33.2%	26.2%	23.2%	21.9%
	Alcohol Consumption, %						
	Never	46.3%	57.9%	37.6%	38.3%	41.3%	42.9%
	2-3 times/month	21.7%	16.7%	25.8%	24.7%	22.2%	22.3%
	1-2 times/week	23.4%	17.4%	28.0%	27.3%	26.0%	25.0%
	≥3 times/week	8.6%	8.1%	8.5%	9.8%	10.5%	9.7%

Supplementary Table 2. Participants' characteristics at 1st, 2nd, 3rd, 4th, 5th and 6th repeat-visit (cont.)

Repeat visit	Variables	All (n= 12,302)	Exercise frequency				
6th			None (n= 4,684)	1-2 times/week (n=4,754)	3-4 times/week (n=1,929)	5-6 times/week (n=358)	Almost everyday (n=577)
	Sex, %						
	Men	76.2%	64.2%	83.8%	84.7%	80.7%	80.4%
	Women	23.8%	35.8%	16.2%	15.3%	19.3%	19.6%
	Age, years	51.3 (4.5)	51.6 (4.7)	51.0 (4.2)	51.0 (4.4)	51.2 (4.3)	52.0 (5.1)
	Body Mass Index, kg^2/m	23.6 (2.6)	23.3 (2.7)	23.7 (2.6)	23.8 (2.4)	23.6 (2.2)	23.7 (2.5)
	Systolic blood pressure, mm Hg	121.5 (12.8)	121.2 (13.0)	121.6 (12.6)	121.7 (12.6)	121.0 (12.5)	122.1 (12.3)
	Diastolic blood pressure, mm Hg	76.7 (9.0)	76.4 (9.0)	77.0 (8.9)	77.0 (9.0)	76.0 (8.7)	76.3 (9.0)
	Fasting glucose levels, mg/dL	94.6 (18.5)	93.7 (18.8)	95.1 (18.7)	94.9 (16.6)	95.1 (18.4)	95.7 (20.1)
	Total cholesterol, mg/dL	197.1 (34.5)	197.9 (35.0)	197.1 (34.2)	195.8 (34.3)	192.0 (32.2)	197.4 (34.3)
	Family history of heart disease, stroke or hypertension, %	13.2%	11.7%	13.7%	15.5%	14.3%	12.7%
	Family history of cancer, %	14.5%	12.9%	15.5%	15.7%	15.1%	14.9%
	Family history of diabetes, %	6.4%	5.5%	6.4%	7.3%	7.3%	9.5%
	Smoking status, %						
	Never	56.9%	66.6%	48.7%	53.6%	57.3%	57.4%
	Previously	14.0%	7.0%	17.3%	20.4%	16.8%	21.1%
	Currently	29.1%	26.5%	34.1%	26.0%	26.0%	21.5%
	Alcohol Consumption, %						
	Never	43.7%	56.5%	35.0%	36.4%	40.5%	37.8%
	2-3 times/month	22.8%	17.0%	27.2%	24.9%	24.6%	24.8%
	1-2 times/week	24.8%	17.6%	30.0%	28.3%	26.5%	27.4%
	≥3 times/week	8.8%	8.8%	7.9%	10.4%	8.4%	10.1%

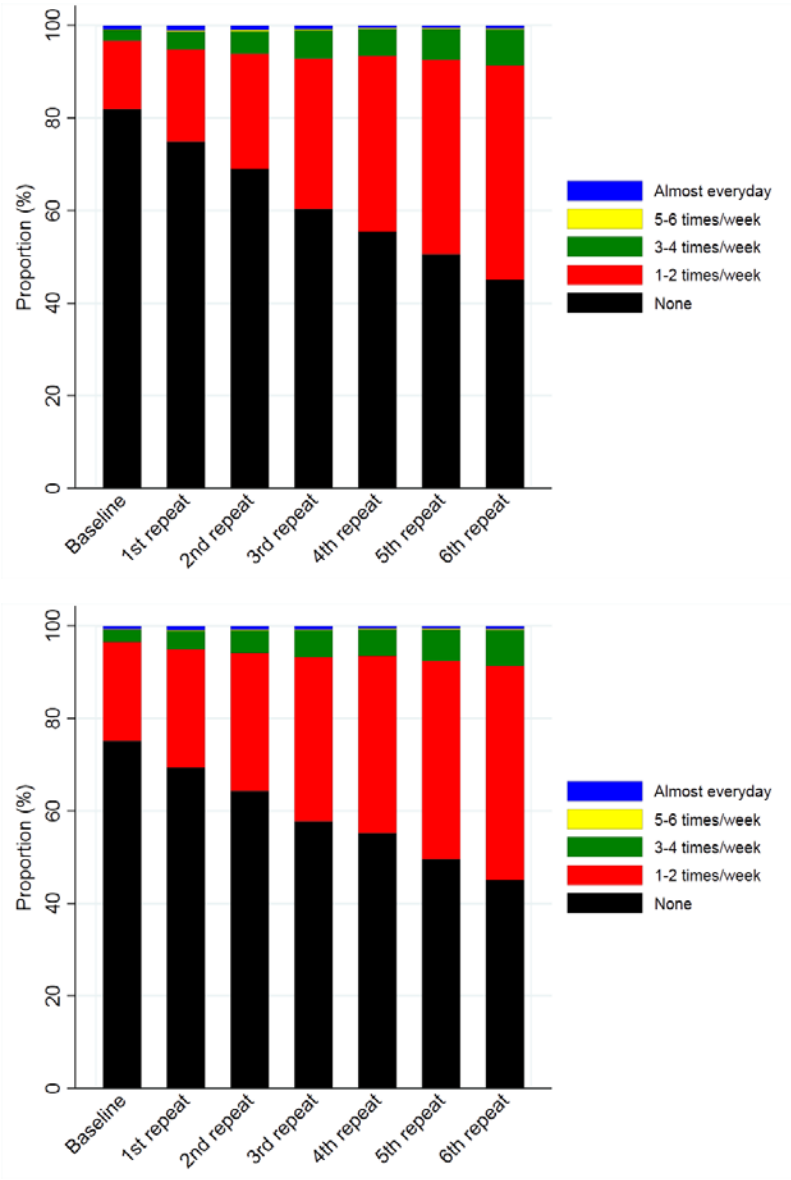

Supplementary Figure 1. Changes in proportions of exercise frequency categories across 7 time points in all 257,854 individuals who provided data at each respective assessment visit (top panel), and 12,302 individuals who provided data from all 7 assessment visits (bottom panel).

Group	HR (95% CI)	Total cases(N)	Unique participants(n)	Events(n)	Crude rate
**Myocardial infarction**					
Exercise frequency: None (Reference)	1.00 (1.00, 1.00)	403356	148284	1741	40.6
Exercise frequency: 1-2 times/week	0.98 (0.90, 1.07)	220601	62923	723	31.7
Exercise frequency: 3-4 times/week	0.83 (0.73, 0.94)	92749	24836	276	29.2
Exercise frequency: 5-6 times/week	0.98 (0.79, 1.22)	22457	6676	88	38.2
Exercise frequency: Almost everyday	0.97 (0.84, 1.12)	45038	15135	219	46.4
**Stroke**					
Exercise frequency: None (Reference)	1.00 (1.00, 1.00)	403356	148284	9689	232.9
Exercise frequency: 1-2 times/week	0.86 (0.82, 0.89)	220601	62923	3333	149.1
Exercise frequency: 3-4 times/week	0.83 (0.79, 0.88)	92749	24836	1482	160.2
Exercise frequency: 5-6 times/week	0.80 (0.72, 0.88)	22457	6676	390	173.1
Exercise frequency: Almost everyday	0.95 (0.90, 1.01)	45038	15135	1240	272.7
**Hypertension**					
Exercise frequency: None (Reference)	1.00 (1.00, 1.00)	403356	148284	65964	2383
Exercise frequency: 1-2 times/week	0.98 (0.96, 0.99)	220601	62923	30623	2114.3
Exercise frequency: 3-4 times/week	0.90 (0.89, 0.92)	92749	24836	12617	2079.8
Exercise frequency: 5-6 times/week	0.91 (0.88, 0.94)	22457	6676	3294	2199.5
Exercise frequency: Almost everyday	1.00 (0.97, 1.02)	45038	15135	7705	2653.7
**Type 2 diabetes**					
Exercise frequency: None (Reference)	1.00 (1.00, 1.00)	403356	148284	27128	1457.6
Exercise frequency: 1-2 times/week	1.00 (0.98, 1.02)	220601	62923	13226	1671.1
Exercise frequency: 3-4 times/week	0.91 (0.89, 0.94)	92749	24836	5421	1745
Exercise frequency: 5-6 times/week	0.92 (0.88, 0.97)	22457	6676	1399	1681.1
Exercise frequency: Almost everyday	1.01 (0.97, 1.04)	45038	15135	3285	1737.5

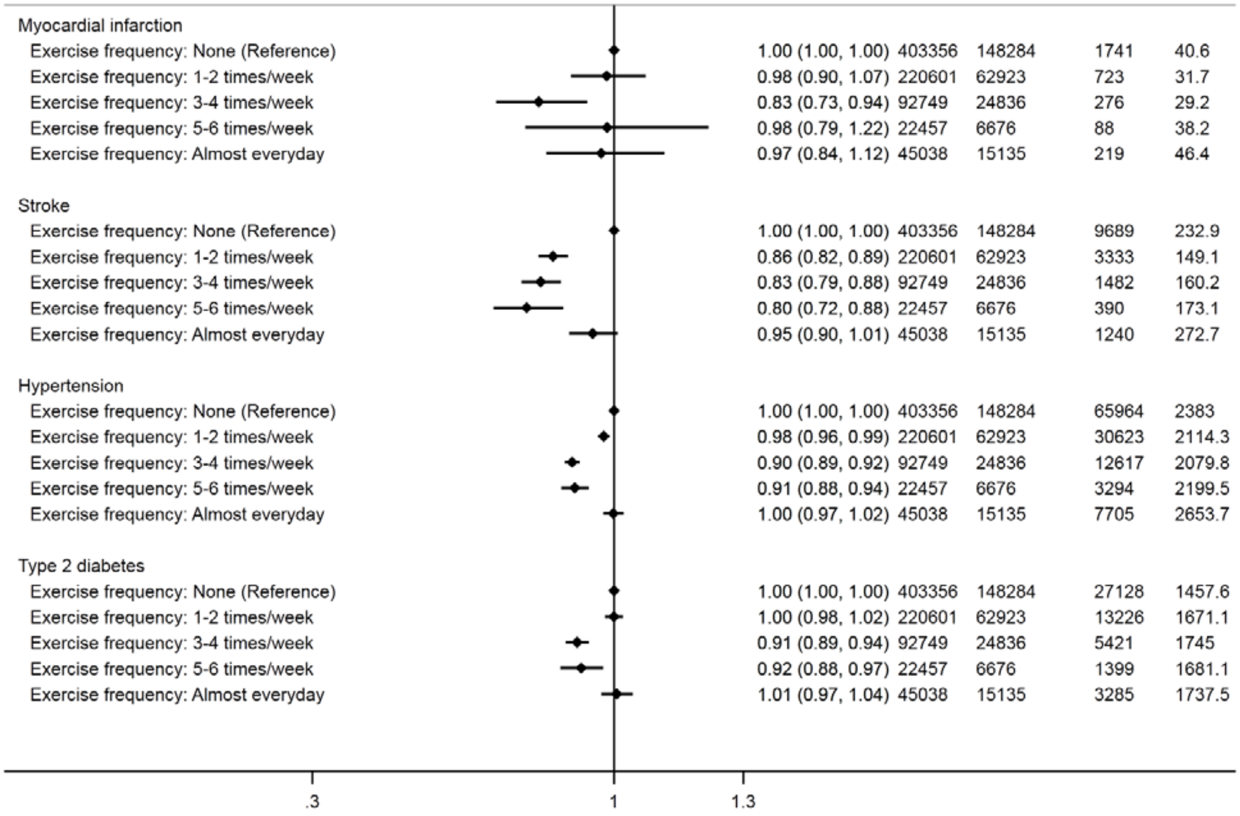

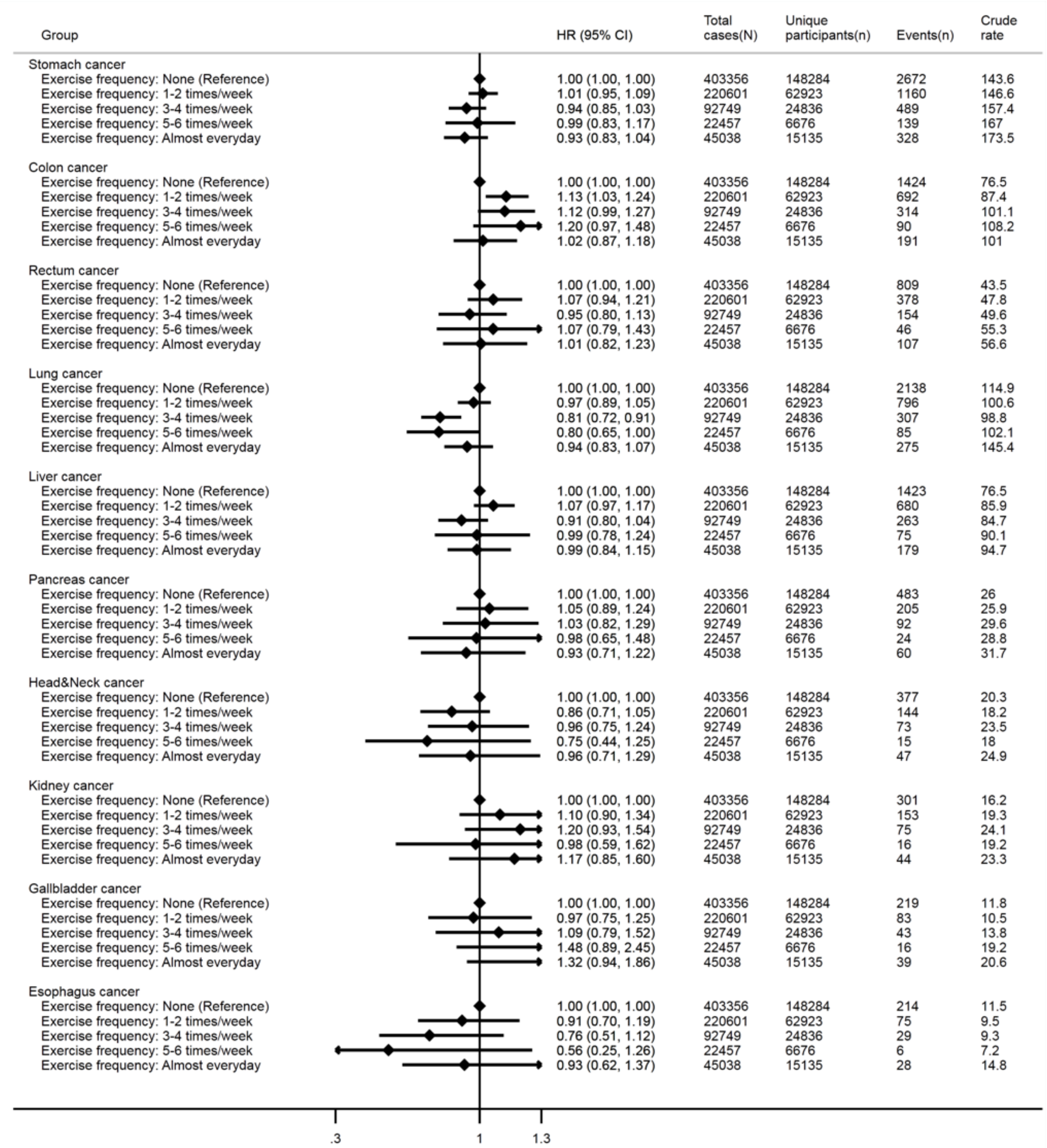

Supplementary Figure 2. Associations of exercise with incident myocardial infarction, stroke, hypertension and Type 2 diabetes (top panel) and various incident cancer outcomes (bottom panel). Note: Cox regression models using age as the underlying timescale were not adjusted for any confounders. Crude rates are per 100,000 person-years. “N” indicates numbers of total observations (i.e. participants who provided repeated measures are treated as separate observations) and “n” indicates numbers of unique participants at baseline. Abbreviations: HR – Hazard Ratio; CI – Confidence Intervals

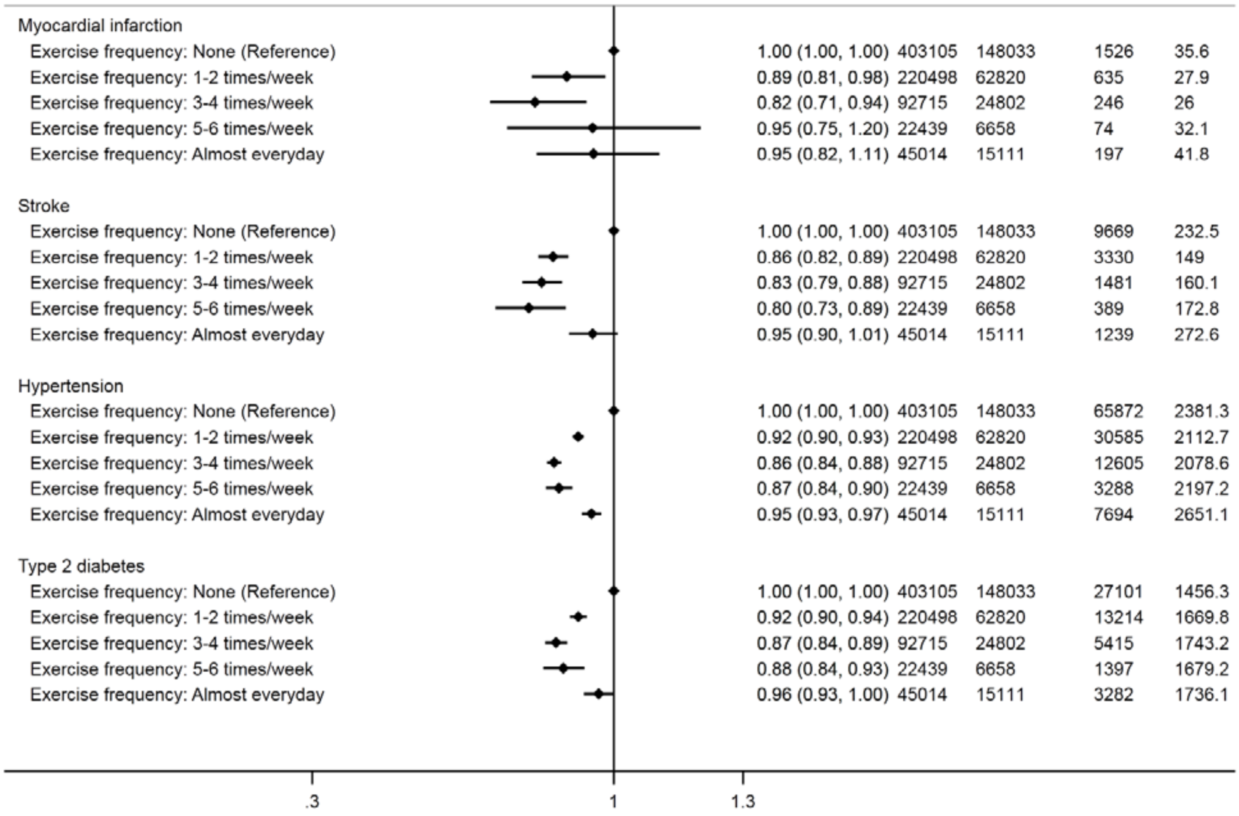

Group	HR (95% CI)	Total cases(N)	Unique participants(n)	Events(n)	Crude rate
**Myocardial infarction**					
Exercise frequency: None (Reference)	1.00 (1.00, 1.00)	403105	148033	1526	35.6
Exercise frequency: 1-2 times/week	0.89 (0.81, 0.98)	220498	62820	635	27.9
Exercise frequency: 3-4 times/week	0.82 (0.71, 0.94)	92715	24802	246	26
Exercise frequency: 5-6 times/week	0.95 (0.75, 1.20)	22439	6658	74	32.1
Exercise frequency: Almost everyday	0.95 (0.82, 1.11)	45014	15111	197	41.8
**Stroke**					
Exercise frequency: None (Reference)	1.00 (1.00, 1.00)	403105	148033	9669	232.5
Exercise frequency: 1-2 times/week	0.86 (0.82, 0.89)	220498	62820	3330	149
Exercise frequency: 3-4 times/week	0.83 (0.79, 0.88)	92715	24802	1481	160.1
Exercise frequency: 5-6 times/week	0.80 (0.73, 0.89)	22439	6658	389	172.8
Exercise frequency: Almost everyday	0.95 (0.90, 1.01)	45014	15111	1239	272.6
**Hypertension**					
Exercise frequency: None (Reference)	1.00 (1.00, 1.00)	403105	148033	65872	2381.3
Exercise frequency: 1-2 times/week	0.92 (0.90, 0.93)	220498	62820	30585	2112.7
Exercise frequency: 3-4 times/week	0.86 (0.84, 0.88)	92715	24802	12605	2078.6
Exercise frequency: 5-6 times/week	0.87 (0.84, 0.90)	22439	6658	3288	2197.2
Exercise frequency: Almost everyday	0.95 (0.93, 0.97)	45014	15111	7694	2651.1
**Type 2 diabetes**					
Exercise frequency: None (Reference)	1.00 (1.00, 1.00)	403105	148033	27101	1456.3
Exercise frequency: 1-2 times/week	0.92 (0.90, 0.94)	220498	62820	13214	1669.8
Exercise frequency: 3-4 times/week	0.87 (0.84, 0.89)	92715	24802	5415	1743.2
Exercise frequency: 5-6 times/week	0.88 (0.84, 0.93)	22439	6658	1397	1679.2
Exercise frequency: Almost everyday	0.96 (0.93, 1.00)	45014	15111	3282	1736.1

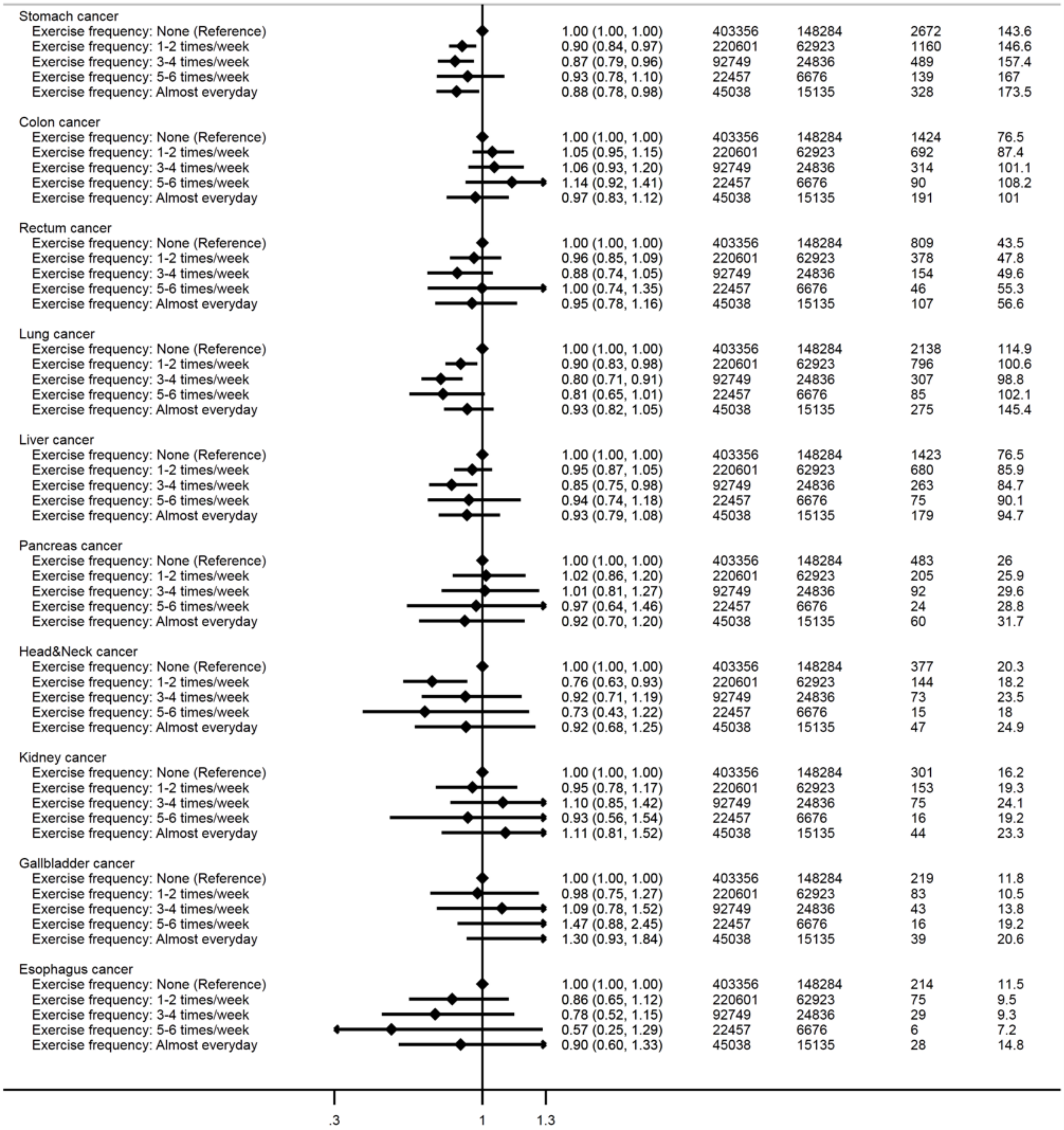

Group	HR (95% CI)	Total cases(N)	Unique participants(n)	Events(n)	Crude rate
**Stomach cancer**					
Exercise frequency: None (Reference)	1.00 (1.00, 1.00)	403356	148284	2672	143.6
Exercise frequency: 1-2 times/week	0.90 (0.84, 0.97)	220601	62923	1160	146.6
Exercise frequency: 3-4 times/week	0.87 (0.79, 0.96)	92749	24836	489	157.4
Exercise frequency: 5-6 times/week	0.93 (0.78, 1.10)	22457	6676	139	167
Exercise frequency: Almost everyday	0.88 (0.78, 0.98)	45038	15135	328	173.5
**Colon cancer**					
Exercise frequency: None (Reference)	1.00 (1.00, 1.00)	403356	148284	1424	76.5
Exercise frequency: 1-2 times/week	1.05 (0.95, 1.15)	220601	62923	692	87.4
Exercise frequency: 3-4 times/week	1.06 (0.93, 1.20)	92749	24836	314	101.1
Exercise frequency: 5-6 times/week	1.14 (0.92, 1.41)	22457	6676	90	108.2
Exercise frequency: Almost everyday	0.97 (0.83, 1.12)	45038	15135	191	101
**Rectum cancer**					
Exercise frequency: None (Reference)	1.00 (1.00, 1.00)	403356	148284	809	43.5
Exercise frequency: 1-2 times/week	0.96 (0.85, 1.09)	220601	62923	378	47.8
Exercise frequency: 3-4 times/week	0.88 (0.74, 1.05)	92749	24836	154	49.6
Exercise frequency: 5-6 times/week	1.00 (0.74, 1.35)	22457	6676	46	55.3
Exercise frequency: Almost everyday	0.95 (0.78, 1.16)	45038	15135	107	56.6
**Lung cancer**					
Exercise frequency: None (Reference)	1.00 (1.00, 1.00)	403356	148284	2138	114.9
Exercise frequency: 1-2 times/week	0.90 (0.83, 0.98)	220601	62923	796	100.6
Exercise frequency: 3-4 times/week	0.80 (0.71, 0.91)	92749	24836	307	98.8
Exercise frequency: 5-6 times/week	0.81 (0.65, 1.01)	22457	6676	85	102.1
Exercise frequency: Almost everyday	0.93 (0.82, 1.05)	45038	15135	275	145.4
**Liver cancer**					
Exercise frequency: None (Reference)	1.00 (1.00, 1.00)	403356	148284	1423	76.5
Exercise frequency: 1-2 times/week	0.95 (0.87, 1.05)	220601	62923	680	85.9
Exercise frequency: 3-4 times/week	0.85 (0.75, 0.98)	92749	24836	263	84.7
Exercise frequency: 5-6 times/week	0.94 (0.74, 1.18)	22457	6676	75	90.1
Exercise frequency: Almost everyday	0.93 (0.79, 1.08)	45038	15135	179	94.7
**Pancreas cancer**					
Exercise frequency: None (Reference)	1.00 (1.00, 1.00)	403356	148284	483	26
Exercise frequency: 1-2 times/week	1.02 (0.86, 1.20)	220601	62923	205	25.9
Exercise frequency: 3-4 times/week	1.01 (0.81, 1.27)	92749	24836	92	29.6
Exercise frequency: 5-6 times/week	0.97 (0.64, 1.46)	22457	6676	24	28.8
Exercise frequency: Almost everyday	0.92 (0.70, 1.20)	45038	15135	60	31.7
**Head&Neck cancer**					
Exercise frequency: None (Reference)	1.00 (1.00, 1.00)	403356	148284	377	20.3
Exercise frequency: 1-2 times/week	0.76 (0.63, 0.93)	220601	62923	144	18.2
Exercise frequency: 3-4 times/week	0.92 (0.71, 1.19)	92749	24836	73	23.5
Exercise frequency: 5-6 times/week	0.73 (0.43, 1.22)	22457	6676	15	18
Exercise frequency: Almost everyday	0.92 (0.68, 1.25)	45038	15135	47	24.9
**Kidney cancer**					
Exercise frequency: None (Reference)	1.00 (1.00, 1.00)	403356	148284	301	16.2
Exercise frequency: 1-2 times/week	0.95 (0.78, 1.17)	220601	62923	153	19.3
Exercise frequency: 3-4 times/week	1.10 (0.85, 1.42)	92749	24836	75	24.1
Exercise frequency: 5-6 times/week	0.93 (0.56, 1.54)	22457	6676	16	19.2
Exercise frequency: Almost everyday	1.11 (0.81, 1.52)	45038	15135	44	23.3
**Gallbladder cancer**					
Exercise frequency: None (Reference)	1.00 (1.00, 1.00)	403356	148284	219	11.8
Exercise frequency: 1-2 times/week	0.98 (0.75, 1.27)	220601	62923	83	10.5
Exercise frequency: 3-4 times/week	1.09 (0.78, 1.52)	92749	24836	43	13.8
Exercise frequency: 5-6 times/week	1.47 (0.88, 2.45)	22457	6676	16	19.2
Exercise frequency: Almost everyday	1.30 (0.93, 1.84)	45038	15135	39	20.6
**Esophagus cancer**					
Exercise frequency: None (Reference)	1.00 (1.00, 1.00)	403356	148284	214	11.5
Exercise frequency: 1-2 times/week	0.86 (0.65, 1.12)	220601	62923	75	9.5
Exercise frequency: 3-4 times/week	0.78 (0.52, 1.15)	92749	24836	29	9.3
Exercise frequency: 5-6 times/week	0.57 (0.25, 1.29)	22457	6676	6	7.2
Exercise frequency: Almost everyday	0.90 (0.60, 1.33)	45038	15135	28	14.8

Supplementary Figure 3. Associations of exercise with incident myocardial infarction, stroke, hypertension and Type 2 diabetes (top panel) and various incident cancer outcomes (bottom panel) after excluding data from the first 2-year follow-up period. Note: Cox regression models using age as the underlying timescale were adjusted for sex, body mass index, systolic blood pressure, fasting glucose levels, total cholesterol levels, family history of heart disease, stroke or hypertension (in models for incident myocardial infarction, stroke and hypertension), diabetes (in models for incident Type 2 diabetes) or cancer (in models for incident cancer outcomes), smoking status and alcohol consumption. Crude rates are per 100,000 person-years. “N”

indicates numbers of total observations (i.e. participants who provided repeated measures are treated as separate observations) and “n” indicates numbers of unique participants at baseline. Abbreviations: HR – Hazard Ratio; CI – Confidence Intervals

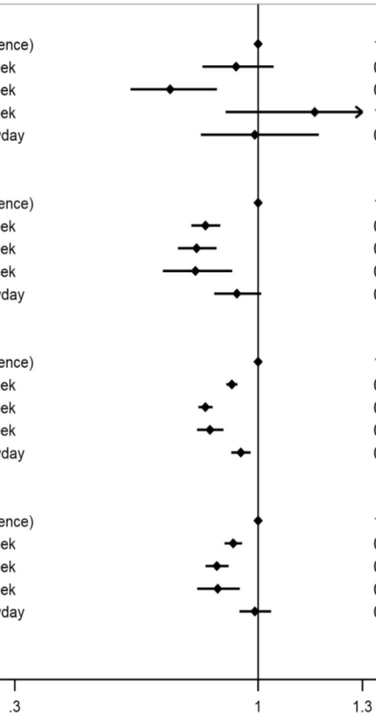

Body Mass Index <25 kg/m²

Group	HR (95% CI)	Total cases(N)	Unique participants(n)	Events(n)	Crude rate
**Myocardial infarction**					
Exercise frequency: None (Reference)	1.00 (1.00, 1.00)	295683	108755	1181	37.5
Exercise frequency: 1-2 times/week	0.94 (0.84, 1.04)	156729	44670	487	30
Exercise frequency: 3-4 times/week	0.75 (0.63, 0.88)	65633	17543	163	24.4
Exercise frequency: 5-6 times/week	1.16 (0.91, 1.49)	16000	4704	66	40.3
Exercise frequency: Almost everyday	0.99 (0.84, 1.17)	31821	10658	150	45
**Stroke**					
Exercise frequency: None (Reference)	1.00 (1.00, 1.00)	295683	108755	6877	225.1
Exercise frequency: 1-2 times/week	0.85 (0.81, 0.89)	156729	44670	2227	139.9
Exercise frequency: 3-4 times/week	0.82 (0.77, 0.88)	65633	17543	990	151.2
Exercise frequency: 5-6 times/week	0.82 (0.73, 0.93)	16000	4704	272	169.6
Exercise frequency: Almost everyday	0.94 (0.87, 1.01)	31821	10658	842	262.2
**Hypertension**					
Exercise frequency: None (Reference)	1.00 (1.00, 1.00)	295683	108755	44055	2061
Exercise frequency: 1-2 times/week	0.92 (0.91, 0.94)	156729	44670	19638	1796.3
Exercise frequency: 3-4 times/week	0.85 (0.83, 0.87)	65633	17543	7967	1745
Exercise frequency: 5-6 times/week	0.86 (0.82, 0.90)	16000	4704	2079	1838.9
Exercise frequency: Almost everyday	0.95 (0.92, 0.98)	31821	10658	4984	2296.9
**Type 2 diabetes**					
Exercise frequency: None (Reference)	1.00 (1.00, 1.00)	295683	108755	17229	613.2
Exercise frequency: 1-2 times/week	0.93 (0.90, 0.95)	156729	44670	7975	552.4
Exercise frequency: 3-4 times/week	0.88 (0.85, 0.92)	65633	17543	3291	548.3
Exercise frequency: 5-6 times/week	0.88 (0.82, 0.95)	16000	4704	844	575.7
Exercise frequency: Almost everyday	0.99 (0.95, 1.04)	31821	10658	2044	697.4

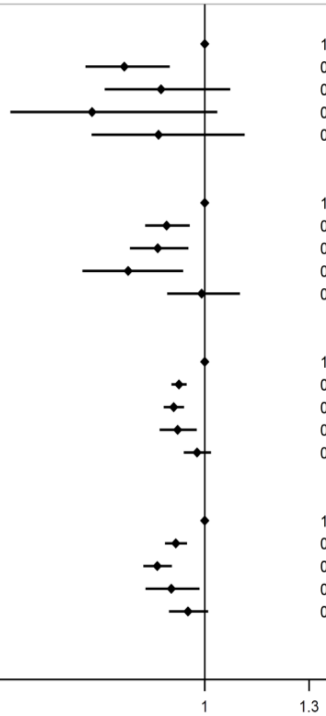

Body Mass Index ≥25 kg/m²

Group	HR (95% CI)	Total cases(N)	Unique participants(n)	Events(n)	Crude rate
**Myocardial infarction**					
Exercise frequency: None (Reference)	1.00 (1.00, 1.00)	107673	39529	560	49
Exercise frequency: 1-2 times/week	0.77 (0.66, 0.90)	63872	18253	236	35.8
Exercise frequency: 3-4 times/week	0.87 (0.71, 1.07)	27116	7293	113	40.9
Exercise frequency: 5-6 times/week	0.68 (0.44, 1.04)	6457	1972	22	33
Exercise frequency: Almost everyday	0.87 (0.67, 1.11)	13217	4477	69	49.8
**Stroke**					
Exercise frequency: None (Reference)	1.00 (1.00, 1.00)	107673	39529	2812	254.4
Exercise frequency: 1-2 times/week	0.89 (0.83, 0.96)	63872	18253	1106	171.8
Exercise frequency: 3-4 times/week	0.86 (0.78, 0.95)	27116	7293	492	182
Exercise frequency: 5-6 times/week	0.78 (0.65, 0.94)	6457	1972	118	181.7
Exercise frequency: Almost everyday	0.99 (0.89, 1.10)	13217	4477	398	298.1
**Hypertension**					
Exercise frequency: None (Reference)	1.00 (1.00, 1.00)	107673	39529	21909	3474.6
Exercise frequency: 1-2 times/week	0.93 (0.90, 0.95)	63872	18253	10985	3093.3
Exercise frequency: 3-4 times/week	0.91 (0.88, 0.94)	27116	7293	4650	3098.2
Exercise frequency: 5-6 times/week	0.92 (0.87, 0.98)	6457	1972	1215	3310.1
Exercise frequency: Almost everyday	0.98 (0.94, 1.02)	13217	4477	2721	3709.4
**Type 2 diabetes**					
Exercise frequency: None (Reference)	1.00 (1.00, 1.00)	107673	39529	9899	1031.5
Exercise frequency: 1-2 times/week	0.92 (0.89, 0.95)	63872	18253	5251	947.9
Exercise frequency: 3-4 times/week	0.86 (0.82, 0.91)	27116	7293	2130	910.9
Exercise frequency: 5-6 times/week	0.90 (0.83, 0.98)	6457	1972	555	986.6
Exercise frequency: Almost everyday	0.95 (0.90, 1.01)	13217	4477	1241	1069.5

Smoking: never

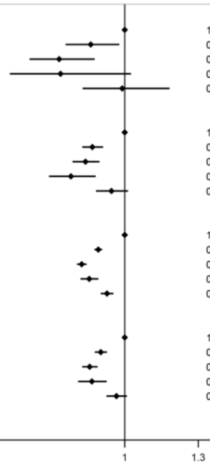

Group	HR (95% CI)	Total cases(N)	Unique participants(n)	Events(n)	Crude rate
Myocardial infarction					
Exercise frequency: None (Reference)	1.00 (1.00, 1.00)	296255	106647	1011	32.3
Exercise frequency: 1-2 times/week	0.86 (0.76, 0.98)	130907	37173	333	24.8
Exercise frequency: 3-4 times/week	0.73 (0.61, 0.88)	60735	16199	137	22.3
Exercise frequency: 5-6 times/week	0.74 (0.53, 1.03)	15420	4593	37	23.5
Exercise frequency: Almost everyday	0.99 (0.83, 1.18)	32380	10849	139	41.2
Stroke					
Exercise frequency: None (Reference)	1.00 (1.00, 1.00)	296255	106647	7174	236.7
Exercise frequency: 1-2 times/week	0.87 (0.83, 0.91)	130907	37173	2171	165.6
Exercise frequency: 3-4 times/week	0.84 (0.79, 0.90)	60735	16199	1050	175.3
Exercise frequency: 5-6 times/week	0.78 (0.69, 0.88)	15420	4593	271	176.5
Exercise frequency: Almost everyday	0.95 (0.88, 1.01)	32380	10849	909	280.4
Hypertension					
Exercise frequency: None (Reference)	1.00 (1.00, 1.00)	296255	106647	47918	2346.4
Exercise frequency: 1-2 times/week	0.89 (0.88, 0.91)	130907	37173	17968	2035.9
Exercise frequency: 3-4 times/week	0.82 (0.81, 0.84)	60735	16199	8086	1979.9
Exercise frequency: 5-6 times/week	0.86 (0.82, 0.89)	15420	4593	2223	2110.5
Exercise frequency: Almost everyday	0.93 (0.90, 0.95)	32380	10849	5556	2625.7
Type 2 diabetes					
Exercise frequency: None (Reference)	1.00 (1.00, 1.00)	296255	106647	18858	677.3
Exercise frequency: 1-2 times/week	0.90 (0.88, 0.93)	130907	37173	7336	612.3
Exercise frequency: 3-4 times/week	0.86 (0.83, 0.89)	60735	16199	3377	614.8
Exercise frequency: 5-6 times/week	0.87 (0.81, 0.93)	15420	4593	889	633.9
Exercise frequency: Almost everyday	0.97 (0.93, 1.01)	32380	10849	2313	782.6

Smoking: previous

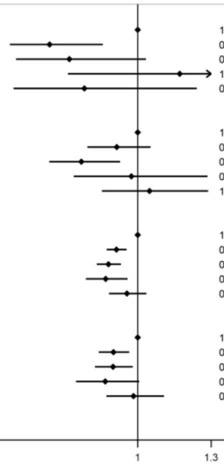

Group	HR (95% CI)	Total cases(N)	Unique participants(n)	Events(n)	Crude rate
Myocardial infarction					
Exercise frequency: None (Reference)	1.00 (1.00, 1.00)	23702	8659	132	52.1
Exercise frequency: 1-2 times/week	0.64 (0.48, 0.86)	28268	7571	75	25.7
Exercise frequency: 3-4 times/week	0.72 (0.50, 1.03)	12863	3156	40	30.5
Exercise frequency: 5-6 times/week	1.17 (0.71, 1.92)	3027	831	18	58.2
Exercise frequency: Almost everyday	0.78 (0.49, 1.24)	4496	1337	21	44.5
Stroke					
Exercise frequency: None (Reference)	1.00 (1.00, 1.00)	23702	8659	507	205.3
Exercise frequency: 1-2 times/week	0.91 (0.79, 1.05)	28268	7571	363	126.4
Exercise frequency: 3-4 times/week	0.77 (0.64, 0.93)	12863	3156	148	114.8
Exercise frequency: 5-6 times/week	0.97 (0.74, 1.28)	3027	831	56	184.8
Exercise frequency: Almost everyday	1.05 (0.85, 1.29)	4496	1337	112	245.4
Hypertension					
Exercise frequency: None (Reference)	1.00 (1.00, 1.00)	23702	8659	4168	2733.5
Exercise frequency: 1-2 times/week	0.91 (0.87, 0.95)	28268	7571	3967	2282.3
Exercise frequency: 3-4 times/week	0.88 (0.83, 0.93)	12863	3156	1791	2339.4
Exercise frequency: 5-6 times/week	0.87 (0.79, 0.96)	3027	831	446	2396.9
Exercise frequency: Almost everyday	0.96 (0.88, 1.04)	4496	1337	708	2629.5
Type 2 diabetes					
Exercise frequency: None (Reference)	1.00 (1.00, 1.00)	23702	8659	1691	776.7
Exercise frequency: 1-2 times/week	0.90 (0.84, 0.97)	28268	7571	1685	669.5
Exercise frequency: 3-4 times/week	0.90 (0.83, 0.98)	12863	3156	787	693.5
Exercise frequency: 5-6 times/week	0.87 (0.75, 1.01)	3027	831	196	733
Exercise frequency: Almost everyday	0.98 (0.87, 1.11)	4496	1337	322	793

Smoking: current

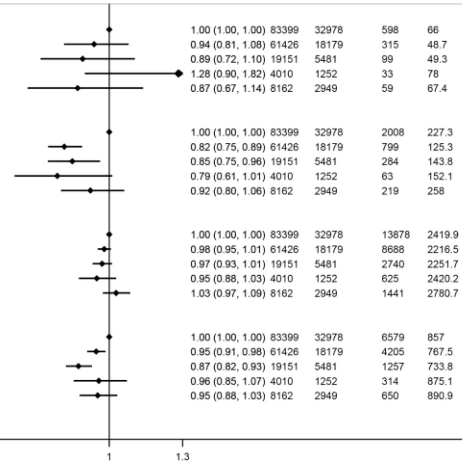

Group	HR (95% CI)	Total cases(N)	Unique participants(n)	Events(n)	Crude rate
Myocardial infarction					
Exercise frequency: None (Reference)	1.00 (1.00, 1.00)	83399	32978	598	66
Exercise frequency: 1-2 times/week	0.94 (0.81, 1.08)	61426	18179	315	48.7
Exercise frequency: 3-4 times/week	0.89 (0.72, 1.10)	19151	5481	99	49.3
Exercise frequency: 5-6 times/week	1.28 (0.90, 1.82)	4010	1252	33	78
Exercise frequency: Almost everyday	0.87 (0.67, 1.14)	8162	2949	59	67.4
Stroke					
Exercise frequency: None (Reference)	1.00 (1.00, 1.00)	83399	32978	2008	227.3
Exercise frequency: 1-2 times/week	0.82 (0.75, 0.89)	61426	18179	799	125.3
Exercise frequency: 3-4 times/week	0.85 (0.75, 0.96)	19151	5481	284	143.8
Exercise frequency: 5-6 times/week	0.79 (0.61, 1.01)	4010	1252	63	152.1
Exercise frequency: Almost everyday	0.92 (0.80, 1.06)	8162	2949	219	258
Hypertension					
Exercise frequency: None (Reference)	1.00 (1.00, 1.00)	83399	32978	13878	2419.9
Exercise frequency: 1-2 times/week	0.98 (0.95, 1.01)	61426	18179	8688	2216.5
Exercise frequency: 3-4 times/week	0.97 (0.93, 1.01)	19151	5481	2740	2251.7
Exercise frequency: 5-6 times/week	0.95 (0.88, 1.03)	4010	1252	625	2420.2
Exercise frequency: Almost everyday	1.03 (0.97, 1.09)	8162	2949	1441	2780.7
Type 2 diabetes					
Exercise frequency: None (Reference)	1.00 (1.00, 1.00)	83399	32978	6579	857
Exercise frequency: 1-2 times/week	0.95 (0.91, 0.98)	61426	18179	4205	767.5
Exercise frequency: 3-4 times/week	0.87 (0.82, 0.93)	19151	5481	1257	733.8
Exercise frequency: 5-6 times/week	0.96 (0.85, 1.07)	4010	1252	314	875.1
Exercise frequency: Almost everyday	0.95 (0.88, 1.03)	8162	2949	650	890.9

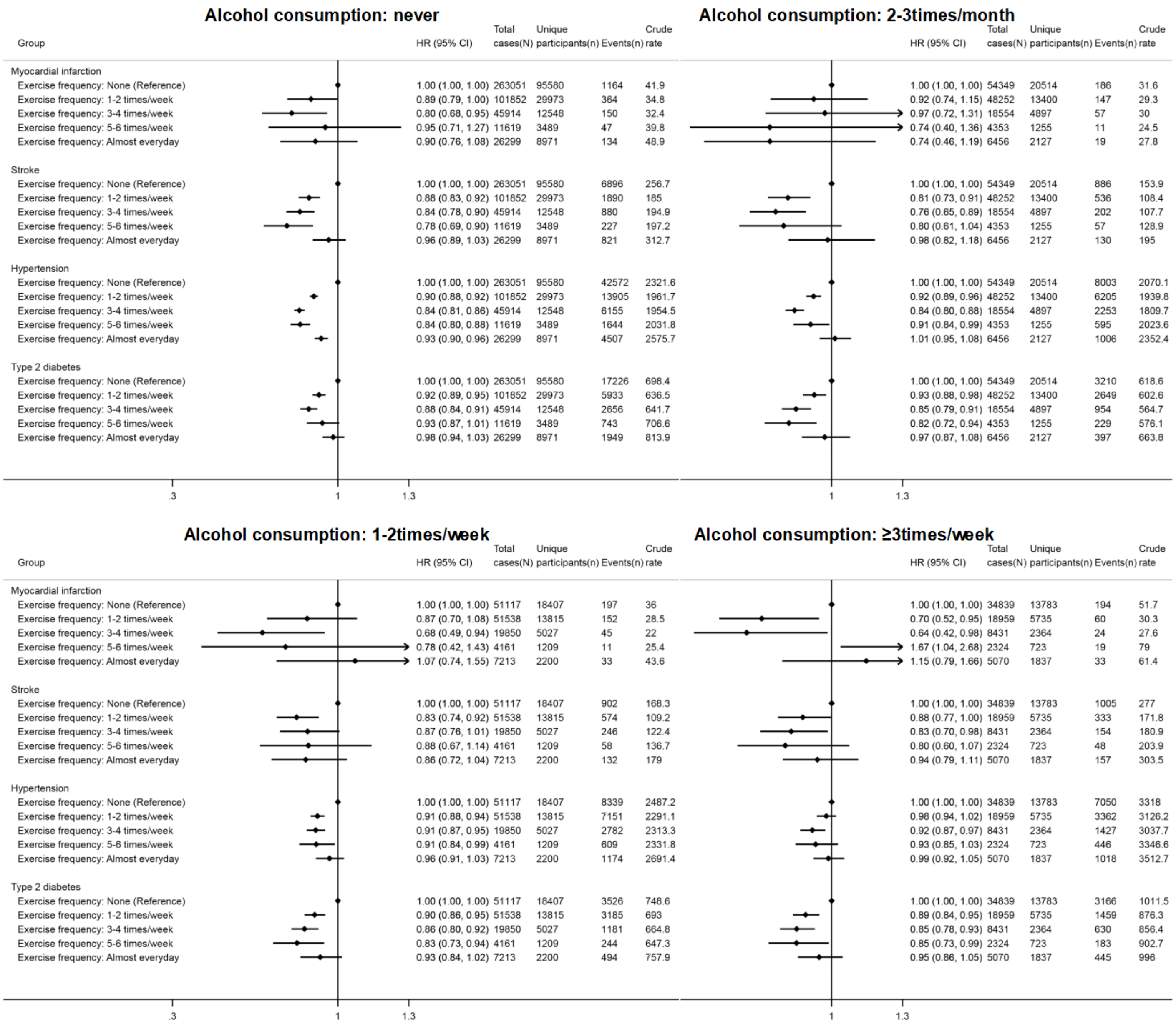

Alcohol consumption: never

Group	HR (95% CI)	Total cases(N)	Unique participants(n)	Events(n)	Crude rate
Myocardial infarction					
Exercise frequency: None (Reference)	1.00 (1.00, 1.00)	263051	95580	1164	41.9
Exercise frequency: 1-2 times/week	0.89 (0.79, 1.00)	101852	29973	364	34.8
Exercise frequency: 3-4 times/week	0.80 (0.68, 0.95)	45914	12548	150	32.4
Exercise frequency: 5-6 times/week	0.95 (0.71, 1.27)	11619	3489	47	39.8
Exercise frequency: Almost everyday	0.90 (0.76, 1.08)	26299	8971	134	48.9
Stroke					
Exercise frequency: None (Reference)	1.00 (1.00, 1.00)	263051	95580	6896	256.7
Exercise frequency: 1-2 times/week	0.88 (0.83, 0.92)	101852	29973	1890	185
Exercise frequency: 3-4 times/week	0.84 (0.78, 0.90)	45914	12548	880	194.9
Exercise frequency: 5-6 times/week	0.78 (0.69, 0.90)	11619	3489	227	197.2
Exercise frequency: Almost everyday	0.96 (0.89, 1.03)	26299	8971	821	312.7
Hypertension					
Exercise frequency: None (Reference)	1.00 (1.00, 1.00)	263051	95580	42572	2321.6
Exercise frequency: 1-2 times/week	0.90 (0.88, 0.92)	101852	29973	13905	1961.7
Exercise frequency: 3-4 times/week	0.84 (0.81, 0.86)	45914	12548	6155	1954.5
Exercise frequency: 5-6 times/week	0.84 (0.80, 0.88)	11619	3489	1644	2031.8
Exercise frequency: Almost everyday	0.93 (0.90, 0.96)	26299	8971	4507	2575.7
Type 2 diabetes					
Exercise frequency: None (Reference)	1.00 (1.00, 1.00)	263051	95580	17226	698.4
Exercise frequency: 1-2 times/week	0.92 (0.89, 0.95)	101852	29973	5933	636.5
Exercise frequency: 3-4 times/week	0.88 (0.84, 0.91)	45914	12548	2656	641.7
Exercise frequency: 5-6 times/week	0.93 (0.87, 1.01)	11619	3489	743	706.6
Exercise frequency: Almost everyday	0.98 (0.94, 1.03)	26299	8971	1949	813.9

Alcohol consumption: 2-3times/month

Group	HR (95% CI)	Total cases(N)	Unique participants(n)	Events(n)	Crude rate
Myocardial infarction					
Exercise frequency: None (Reference)	1.00 (1.00, 1.00)	54349	20514	186	31.6
Exercise frequency: 1-2 times/week	0.92 (0.74, 1.15)	48252	13400	147	29.3
Exercise frequency: 3-4 times/week	0.97 (0.72, 1.31)	18554	4897	57	30
Exercise frequency: 5-6 times/week	0.74 (0.40, 1.36)	4353	1255	11	24.5
Exercise frequency: Almost everyday	0.74 (0.46, 1.19)	6456	2127	19	27.8
Stroke					
Exercise frequency: None (Reference)	1.00 (1.00, 1.00)	54349	20514	886	153.9
Exercise frequency: 1-2 times/week	0.81 (0.73, 0.91)	48252	13400	536	108.4
Exercise frequency: 3-4 times/week	0.76 (0.65, 0.89)	18554	4897	202	107.7
Exercise frequency: 5-6 times/week	0.80 (0.61, 1.04)	4353	1255	57	128.9
Exercise frequency: Almost everyday	0.98 (0.82, 1.18)	6456	2127	130	195
Hypertension					
Exercise frequency: None (Reference)	1.00 (1.00, 1.00)	54349	20514	8003	2070.1
Exercise frequency: 1-2 times/week	0.92 (0.89, 0.96)	48252	13400	6205	1939.8
Exercise frequency: 3-4 times/week	0.84 (0.80, 0.88)	18554	4897	2253	1809.7
Exercise frequency: 5-6 times/week	0.91 (0.84, 0.99)	4353	1255	595	2023.6
Exercise frequency: Almost everyday	1.01 (0.95, 1.08)	6456	2127	1006	2352.4
Type 2 diabetes					
Exercise frequency: None (Reference)	1.00 (1.00, 1.00)	54349	20514	3210	618.6
Exercise frequency: 1-2 times/week	0.93 (0.88, 0.98)	48252	13400	2649	602.6
Exercise frequency: 3-4 times/week	0.85 (0.79, 0.91)	18554	4897	954	564.7
Exercise frequency: 5-6 times/week	0.82 (0.72, 0.94)	4353	1255	229	576.1
Exercise frequency: Almost everyday	0.97 (0.87, 1.08)	6456	2127	397	663.8

Alcohol consumption: 1-2times/week

Group	HR (95% CI)	Total cases(N)	Unique participants(n)	Events(n)	Crude rate
Myocardial infarction					
Exercise frequency: None (Reference)	1.00 (1.00, 1.00)	51117	18407	197	36
Exercise frequency: 1-2 times/week	0.87 (0.70, 1.08)	51538	13815	152	28.5
Exercise frequency: 3-4 times/week	0.68 (0.49, 0.94)	19850	5027	45	22
Exercise frequency: 5-6 times/week	0.78 (0.42, 1.43)	4161	1209	11	25.4
Exercise frequency: Almost everyday	1.07 (0.74, 1.55)	7213	2200	33	43.6
Stroke					
Exercise frequency: None (Reference)	1.00 (1.00, 1.00)	51117	18407	902	168.3
Exercise frequency: 1-2 times/week	0.83 (0.74, 0.92)	51538	13815	574	109.2
Exercise frequency: 3-4 times/week	0.87 (0.76, 1.01)	19850	5027	246	122.4
Exercise frequency: 5-6 times/week	0.88 (0.67, 1.14)	4161	1209	58	136.7
Exercise frequency: Almost everyday	0.86 (0.72, 1.04)	7213	2200	132	179
Hypertension					
Exercise frequency: None (Reference)	1.00 (1.00, 1.00)	51117	18407	8339	2487.2
Exercise frequency: 1-2 times/week	0.91 (0.88, 0.94)	51538	13815	7151	2291.1
Exercise frequency: 3-4 times/week	0.91 (0.87, 0.95)	19850	5027	2782	2313.3
Exercise frequency: 5-6 times/week	0.91 (0.84, 0.99)	4161	1209	609	2331.8
Exercise frequency: Almost everyday	0.96 (0.91, 1.03)	7213	2200	1174	2691.4
Type 2 diabetes					
Exercise frequency: None (Reference)	1.00 (1.00, 1.00)	51117	18407	3526	748.6
Exercise frequency: 1-2 times/week	0.90 (0.86, 0.95)	51538	13815	3185	693
Exercise frequency: 3-4 times/week	0.86 (0.80, 0.92)	19850	5027	1181	664.8
Exercise frequency: 5-6 times/week	0.83 (0.73, 0.94)	4161	1209	244	647.3
Exercise frequency: Almost everyday	0.93 (0.84, 1.02)	7213	2200	494	757.9

Alcohol consumption: ≥3times/week

Group	HR (95% CI)	Total cases(N)	Unique participants(n)	Events(n)	Crude rate
Myocardial infarction					
Exercise frequency: None (Reference)	1.00 (1.00, 1.00)	34839	13783	194	51.7
Exercise frequency: 1-2 times/week	0.70 (0.52, 0.95)	18959	5735	60	30.3
Exercise frequency: 3-4 times/week	0.64 (0.42, 0.98)	8431	2364	24	27.6
Exercise frequency: 5-6 times/week	1.67 (1.04, 2.68)	2324	723	19	79
Exercise frequency: Almost everyday	1.15 (0.79, 1.66)	5070	1837	33	61.4
Stroke					
Exercise frequency: None (Reference)	1.00 (1.00, 1.00)	34839	13783	1005	277
Exercise frequency: 1-2 times/week	0.88 (0.77, 1.00)	18959	5735	333	171.8
Exercise frequency: 3-4 times/week	0.83 (0.70, 0.98)	8431	2364	154	180.9
Exercise frequency: 5-6 times/week	0.80 (0.60, 1.07)	2324	723	48	203.9
Exercise frequency: Almost everyday	0.94 (0.79, 1.11)	5070	1837	157	303.5
Hypertension					
Exercise frequency: None (Reference)	1.00 (1.00, 1.00)	34839	13783	7050	3318
Exercise frequency: 1-2 times/week	0.98 (0.94, 1.02)	18959	5735	3362	3126.2
Exercise frequency: 3-4 times/week	0.92 (0.87, 0.97)	8431	2364	1427	3037.7
Exercise frequency: 5-6 times/week	0.93 (0.85, 1.03)	2324	723	446	3346.6
Exercise frequency: Almost everyday	0.99 (0.92, 1.05)	5070	1837	1018	3512.7
Type 2 diabetes					
Exercise frequency: None (Reference)	1.00 (1.00, 1.00)	34839	13783	3166	1011.5
Exercise frequency: 1-2 times/week	0.89 (0.84, 0.95)	18959	5735	1459	876.3
Exercise frequency: 3-4 times/week	0.85 (0.78, 0.93)	8431	2364	630	856.4
Exercise frequency: 5-6 times/week	0.85 (0.73, 0.99)	2324	723	183	902.7
Exercise frequency: Almost everyday	0.95 (0.86, 1.05)	5070	1837	445	996

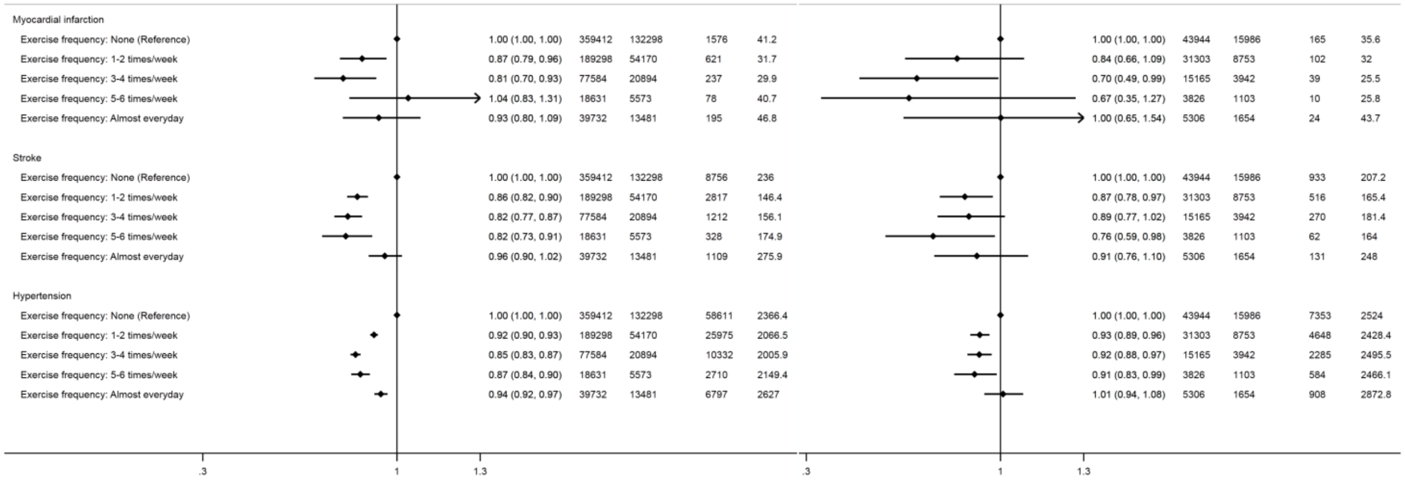

No family history of CVD

Group	HR (95% CI)	Total cases(N)	Unique participants(n)	Events(n)	Crude rate
Myocardial infarction					
Exercise frequency: None (Reference)	1.00 (1.00, 1.00)	359412	132298	1576	41.2
Exercise frequency: 1-2 times/week	0.87 (0.79, 0.96)	189298	54170	621	31.7
Exercise frequency: 3-4 times/week	0.81 (0.70, 0.93)	77584	20894	237	29.9
Exercise frequency: 5-6 times/week	1.04 (0.83, 1.31)	18631	5573	78	40.7
Exercise frequency: Almost everyday	0.93 (0.80, 1.09)	39732	13481	195	46.8
Stroke					
Exercise frequency: None (Reference)	1.00 (1.00, 1.00)	359412	132298	8756	236
Exercise frequency: 1-2 times/week	0.86 (0.82, 0.90)	189298	54170	2817	146.4
Exercise frequency: 3-4 times/week	0.82 (0.77, 0.87)	77584	20894	1212	156.1
Exercise frequency: 5-6 times/week	0.82 (0.73, 0.91)	18631	5573	328	174.9
Exercise frequency: Almost everyday	0.96 (0.90, 1.02)	39732	13481	1109	275.9
Hypertension					
Exercise frequency: None (Reference)	1.00 (1.00, 1.00)	359412	132298	58611	2366.4
Exercise frequency: 1-2 times/week	0.92 (0.90, 0.93)	189298	54170	25975	2066.5
Exercise frequency: 3-4 times/week	0.85 (0.83, 0.87)	77584	20894	10332	2005.9
Exercise frequency: 5-6 times/week	0.87 (0.84, 0.90)	18631	5573	2710	2149.4
Exercise frequency: Almost everyday	0.94 (0.92, 0.97)	39732	13481	6797	2627

Family history of CVD

Group	HR (95% CI)	Total cases(N)	Unique participants(n)	Events(n)	Crude rate
Myocardial infarction					
Exercise frequency: None (Reference)	1.00 (1.00, 1.00)	43944	15986	165	35.6
Exercise frequency: 1-2 times/week	0.84 (0.66, 1.09)	31303	8753	102	32
Exercise frequency: 3-4 times/week	0.70 (0.49, 0.99)	15165	3942	39	25.5
Exercise frequency: 5-6 times/week	0.67 (0.35, 1.27)	3826	1103	10	25.8
Exercise frequency: Almost everyday	1.00 (0.65, 1.54)	5306	1654	24	43.7
Stroke					
Exercise frequency: None (Reference)	1.00 (1.00, 1.00)	43944	15986	933	207.2
Exercise frequency: 1-2 times/week	0.87 (0.78, 0.97)	31303	8753	516	165.4
Exercise frequency: 3-4 times/week	0.89 (0.77, 1.02)	15165	3942	270	181.4
Exercise frequency: 5-6 times/week	0.76 (0.59, 0.98)	3826	1103	62	164
Exercise frequency: Almost everyday	0.91 (0.76, 1.10)	5306	1654	131	248
Hypertension					
Exercise frequency: None (Reference)	1.00 (1.00, 1.00)	43944	15986	7353	2524
Exercise frequency: 1-2 times/week	0.93 (0.89, 0.96)	31303	8753	4648	2428.4
Exercise frequency: 3-4 times/week	0.92 (0.88, 0.97)	15165	3942	2285	2495.5
Exercise frequency: 5-6 times/week	0.91 (0.83, 0.99)	3826	1103	584	2466.1
Exercise frequency: Almost everyday	1.01 (0.94, 1.08)	5306	1654	908	2872.8

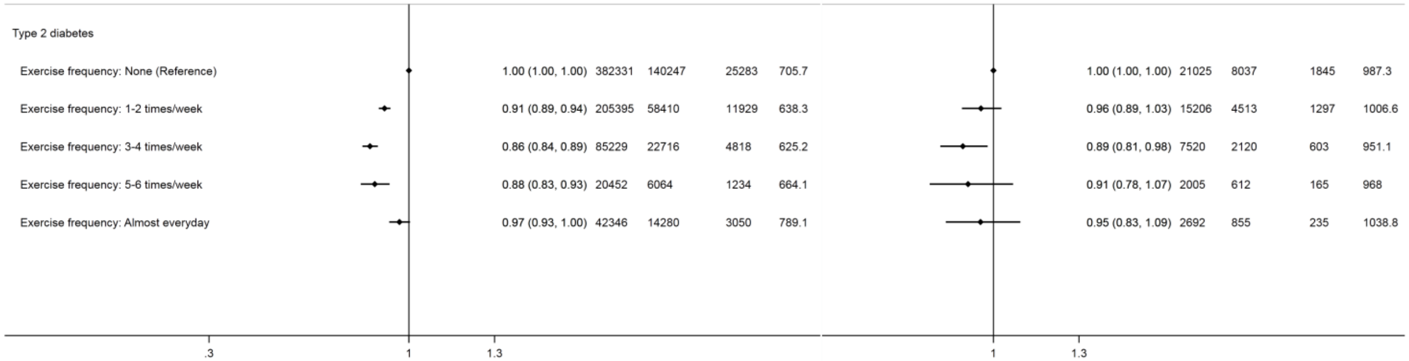

No family history of diabetes

Group	HR (95% CI)	Total cases(N)	Unique participants(n)	Events(n)	Crude rate
Type 2 diabetes					
Exercise frequency: None (Reference)	1.00 (1.00, 1.00)	382331	140247	25283	705.7
Exercise frequency: 1-2 times/week	0.91 (0.89, 0.94)	205395	58410	11929	638.3
Exercise frequency: 3-4 times/week	0.86 (0.84, 0.89)	85229	22716	4818	625.2
Exercise frequency: 5-6 times/week	0.88 (0.83, 0.93)	20452	6064	1234	664.1
Exercise frequency: Almost everyday	0.97 (0.93, 1.00)	42346	14280	3050	789.1

Family history of diabetes

Group	HR (95% CI)	Total cases(N)	Unique participants(n)	Events(n)	Crude rate
Type 2 diabetes					
Exercise frequency: None (Reference)	1.00 (1.00, 1.00)	21025	8037	1845	987.3
Exercise frequency: 1-2 times/week	0.96 (0.89, 1.03)	15206	4513	1297	1006.6
Exercise frequency: 3-4 times/week	0.89 (0.81, 0.98)	7520	2120	603	951.1
Exercise frequency: 5-6 times/week	0.91 (0.78, 1.07)	2005	612	165	968
Exercise frequency: Almost everyday	0.95 (0.83, 1.09)	2692	855	235	1038.8

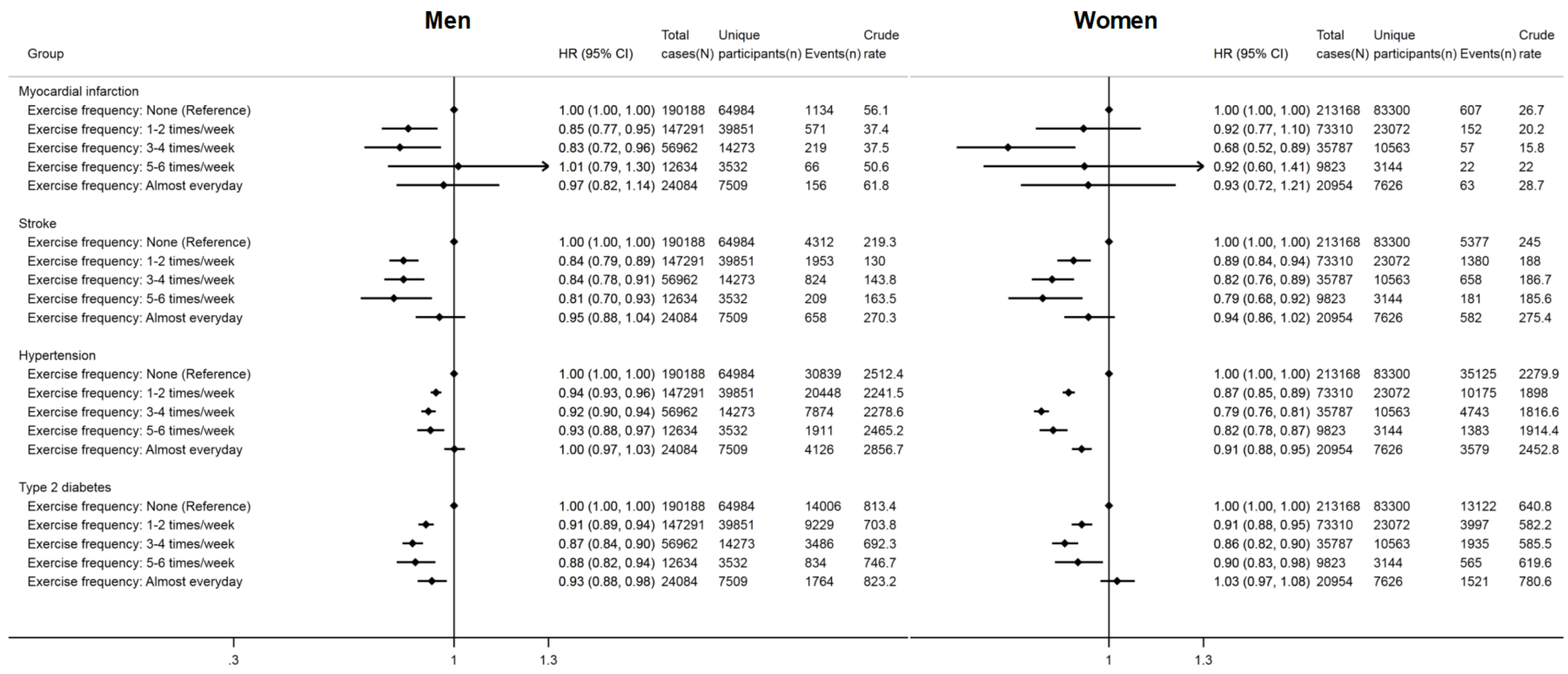

Supplementary Figure 4. Results from running Cox regression models examining effect modification of sex, body mass index, smoking, alcohol consumption, and family history of disease in the associations between exercise frequency and incident myocardial infarction, stroke, hypertension and type 2 diabetes. Note: Cox regression models using age as the underlying timescale were adjusted for sex [not in models for effect modification by sex], body mass index [not in models for effect modification by body mass index], systolic blood pressure, fasting glucose levels, total cholesterol levels, family history [not in models for effect modification by family history of respective disease] of heart disease/stroke/hypertension (in models for myocardial infarction, stroke and hypertension), or diabetes (in models for Type 2 diabetes), smoking status [not in models for effect modification by smoking status] and alcohol consumption [not in models for effect modification by alcohol consumption]. Crude rates are per 100,000 person-years. “N” indicates numbers of total observations (i.e. participants who provided repeated measures are treated as separate observations) and “n” indicates numbers of unique participants at baseline. P-values for multiplicative terms – myocardial infarction (p-value = 0.235), stroke (p-value = 0.363), hypertension (p-value = 0.050) and type 2 diabetes (p-value = 0.196) by body mass index;

myocardial infarction (p-value = 0.643), stroke (p-value = 0.661), hypertension (p-value <0.001) and type 2 diabetes (p-value = 0.980) by smoking; myocardial infarction (p-value = 0.300), stroke (p-value = 0.607), hypertension (p-value <0.001) and type 2 diabetes (p-value = 0.081) by alcohol consumption; myocardial infarction (p-value = 0.590), stroke (p-value = 0.505), and hypertension (p-value = 0.029) by family history of cardiovascular disease; type 2 diabetes (p-value = 0.952) by family history of diabetes; and myocardial infarction (p-value = 0.334), stroke (p-value = 0.818), hypertension (p-value <0.001) and type 2 diabetes (p-value = 0.012) by sex.
Abbreviations: HR – Hazard Ratio; CI – Confidence Intervals

Body Mass Index <25 kg/m²

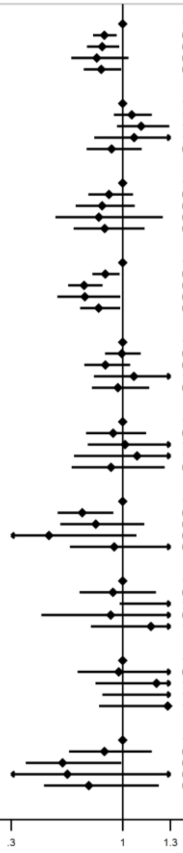

Group	HR (95% CI)	Total cases(N)	Unique participants(n)	Events(n)	Crude rate
Stomach cancer					
Exercise frequency: None (Reference)	1.00 (1.00, 1.00)	295683	108755	2005	64
Exercise frequency: 1-2 times/week	0.88 (0.81, 0.96)	156729	44670	802	49.6
Exercise frequency: 3-4 times/week	0.87 (0.78, 0.98)	65633	17543	343	51.5
Exercise frequency: 5-6 times/week	0.84 (0.68, 1.04)	16000	4704	89	54.4
Exercise frequency: Almost everyday	0.87 (0.76, 0.99)	31821	10658	235	70.9
Colon cancer					
Exercise frequency: None (Reference)	1.00 (1.00, 1.00)	295683	108755	1019	32.4
Exercise frequency: 1-2 times/week	1.06 (0.94, 1.18)	156729	44670	471	29
Exercise frequency: 3-4 times/week	1.12 (0.96, 1.29)	65633	17543	224	33.5
Exercise frequency: 5-6 times/week	1.07 (0.82, 1.40)	16000	4704	58	35.4
Exercise frequency: Almost everyday	0.93 (0.77, 1.12)	31821	10658	127	38.1
Rectum cancer					
Exercise frequency: None (Reference)	1.00 (1.00, 1.00)	295683	108755	591	18.7
Exercise frequency: 1-2 times/week	0.91 (0.78, 1.06)	156729	44670	248	15.3
Exercise frequency: 3-4 times/week	0.87 (0.71, 1.08)	65633	17543	104	15.5
Exercise frequency: 5-6 times/week	0.85 (0.58, 1.25)	16000	4704	27	16.4
Exercise frequency: Almost everyday	0.89 (0.69, 1.14)	31821	10658	70	21
Lung cancer					
Exercise frequency: None (Reference)	1.00 (1.00, 1.00)	295683	108755	1708	54.4
Exercise frequency: 1-2 times/week	0.89 (0.81, 0.98)	156729	44670	605	37.3
Exercise frequency: 3-4 times/week	0.76 (0.66, 0.87)	65633	17543	220	32.9
Exercise frequency: 5-6 times/week	0.76 (0.59, 0.99)	16000	4704	61	37.2
Exercise frequency: Almost everyday	0.85 (0.73, 0.98)	31821	10658	198	59.6
Liver cancer					
Exercise frequency: None (Reference)	1.00 (1.00, 1.00)	295683	108755	1019	32.3
Exercise frequency: 1-2 times/week	0.99 (0.89, 1.11)	156729	44670	470	29
Exercise frequency: 3-4 times/week	0.89 (0.76, 1.05)	65633	17543	179	26.8
Exercise frequency: 5-6 times/week	1.07 (0.82, 1.40)	16000	4704	57	34.8
Exercise frequency: Almost everyday	0.97 (0.81, 1.17)	31821	10658	128	38.4
Pancreas cancer					
Exercise frequency: None (Reference)	1.00 (1.00, 1.00)	295683	108755	373	11.8
Exercise frequency: 1-2 times/week	0.94 (0.77, 1.15)	156729	44670	142	8.7
Exercise frequency: 3-4 times/week	1.01 (0.78, 1.32)	65633	17543	68	10.1
Exercise frequency: 5-6 times/week	1.09 (0.69, 1.71)	16000	4704	20	12.2
Exercise frequency: Almost everyday	0.93 (0.68, 1.26)	31821	10658	45	13.5
Head&Neck cancer					
Exercise frequency: None (Reference)	1.00 (1.00, 1.00)	295683	108755	288	9.1
Exercise frequency: 1-2 times/week	0.75 (0.59, 0.94)	156729	44670	101	6.2
Exercise frequency: 3-4 times/week	0.83 (0.61, 1.14)	65633	17543	47	7
Exercise frequency: 5-6 times/week	0.54 (0.27, 1.09)	16000	4704	8	4.9
Exercise frequency: Almost everyday	0.95 (0.67, 1.34)	31821	10658	36	10.8
Kidney cancer					
Exercise frequency: None (Reference)	1.00 (1.00, 1.00)	295683	108755	201	6.4
Exercise frequency: 1-2 times/week	0.94 (0.73, 1.21)	156729	44670	93	5.7
Exercise frequency: 3-4 times/week	1.33 (0.98, 1.79)	65633	17543	56	8.4
Exercise frequency: 5-6 times/week	0.93 (0.49, 1.75)	16000	4704	10	6.1
Exercise frequency: Almost everyday	1.18 (0.80, 1.73)	31821	10658	30	9
Gallbladder cancer					
Exercise frequency: None (Reference)	1.00 (1.00, 1.00)	295683	108755	162	5.1
Exercise frequency: 1-2 times/week	0.98 (0.72, 1.33)	156729	44670	58	3.6
Exercise frequency: 3-4 times/week	1.21 (0.83, 1.77)	65633	17543	33	4.9
Exercise frequency: 5-6 times/week	1.57 (0.87, 2.84)	16000	4704	12	7.3
Exercise frequency: Almost everyday	1.28 (0.85, 1.93)	31821	10658	27	8.1
Esophagus cancer					
Exercise frequency: None (Reference)	1.00 (1.00, 1.00)	295683	108755	188	5.9
Exercise frequency: 1-2 times/week	0.89 (0.66, 1.18)	156729	44670	66	4.1
Exercise frequency: 3-4 times/week	0.62 (0.39, 0.99)	65633	17543	20	3
Exercise frequency: 5-6 times/week	0.65 (0.29, 1.48)	16000	4704	6	3.6
Exercise frequency: Almost everyday	0.79 (0.51, 1.23)	31821	10658	22	6.6

Body Mass Index ≥25 kg/m²

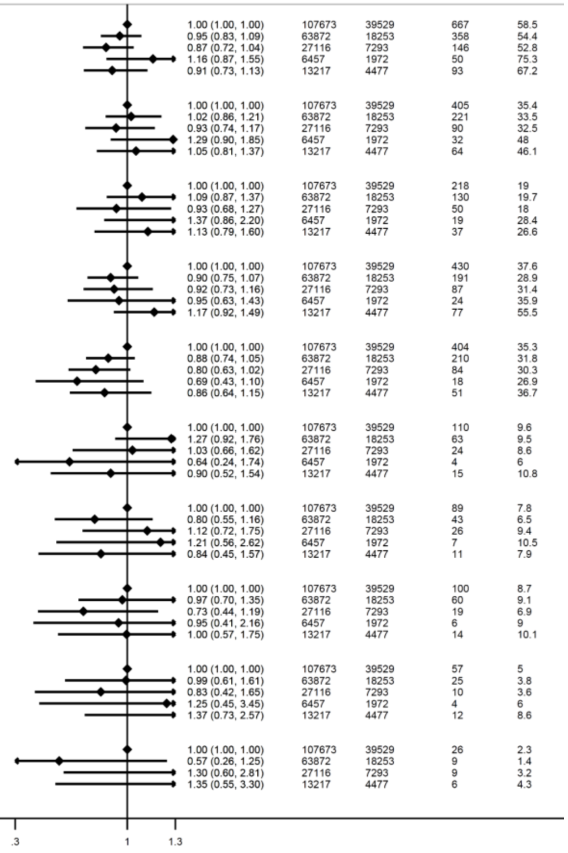

HR (95% CI)	Total cases(N)	Unique participants(n)	Events(n)	Crude rate
1.00 (1.00, 1.00)	107673	39529	667	58.5
0.95 (0.83, 1.09)	63872	18253	358	54.4
0.87 (0.72, 1.04)	27116	7293	146	52.8
1.16 (0.87, 1.55)	6457	1972	50	75.3
0.91 (0.73, 1.13)	13217	4477	93	67.2
1.00 (1.00, 1.00)	107673	39529	405	35.4
1.02 (0.86, 1.21)	63872	18253	221	33.5
0.93 (0.74, 1.17)	27116	7293	90	32.5
1.29 (0.90, 1.85)	6457	1972	32	48
1.05 (0.81, 1.37)	13217	4477	64	46.1
1.00 (1.00, 1.00)	107673	39529	218	19
1.09 (0.87, 1.37)	63872	18253	130	19.7
0.93 (0.68, 1.27)	27116	7293	50	18
1.37 (0.86, 2.20)	6457	1972	19	28.4
1.13 (0.79, 1.60)	13217	4477	37	26.6
1.00 (1.00, 1.00)	107673	39529	430	37.6
0.90 (0.75, 1.07)	63872	18253	191	28.9
0.92 (0.73, 1.16)	27116	7293	87	31.4
0.95 (0.63, 1.43)	6457	1972	24	35.9
1.17 (0.92, 1.49)	13217	4477	77	55.5
1.00 (1.00, 1.00)	107673	39529	404	35.3
0.88 (0.74, 1.05)	63872	18253	210	31.8
0.80 (0.63, 1.02)	27116	7293	84	30.3
0.69 (0.43, 1.10)	6457	1972	18	26.9
0.86 (0.64, 1.15)	13217	4477	51	36.7
1.00 (1.00, 1.00)	107673	39529	110	9.6
1.27 (0.92, 1.76)	63872	18253	63	9.5
1.03 (0.66, 1.62)	27116	7293	24	8.6
0.64 (0.24, 1.74)	6457	1972	4	6
0.90 (0.52, 1.54)	13217	4477	15	10.8
1.00 (1.00, 1.00)	107673	39529	89	7.8
0.80 (0.55, 1.16)	63872	18253	43	6.5
1.12 (0.72, 1.75)	27116	7293	26	9.4
1.21 (0.56, 2.62)	6457	1972	7	10.5
0.84 (0.45, 1.57)	13217	4477	11	7.9
1.00 (1.00, 1.00)	107673	39529	100	8.7
0.97 (0.70, 1.35)	63872	18253	60	9.1
0.73 (0.44, 1.19)	27116	7293	19	6.9
0.95 (0.41, 2.16)	6457	1972	6	9
1.00 (0.57, 1.75)	13217	4477	14	10.1
1.00 (1.00, 1.00)	107673	39529	57	5
0.99 (0.61, 1.61)	63872	18253	25	3.8
0.83 (0.42, 1.65)	27116	7293	10	3.6
1.25 (0.45, 3.45)	6457	1972	4	6
1.37 (0.73, 2.57)	13217	4477	12	8.6
1.00 (1.00, 1.00)	107673	39529	26	2.3
0.57 (0.26, 1.25)	63872	18253	9	1.4
1.30 (0.60, 2.81)	27116	7293	9	3.2
1.35 (0.55, 3.30)	13217	4477	6	4.3

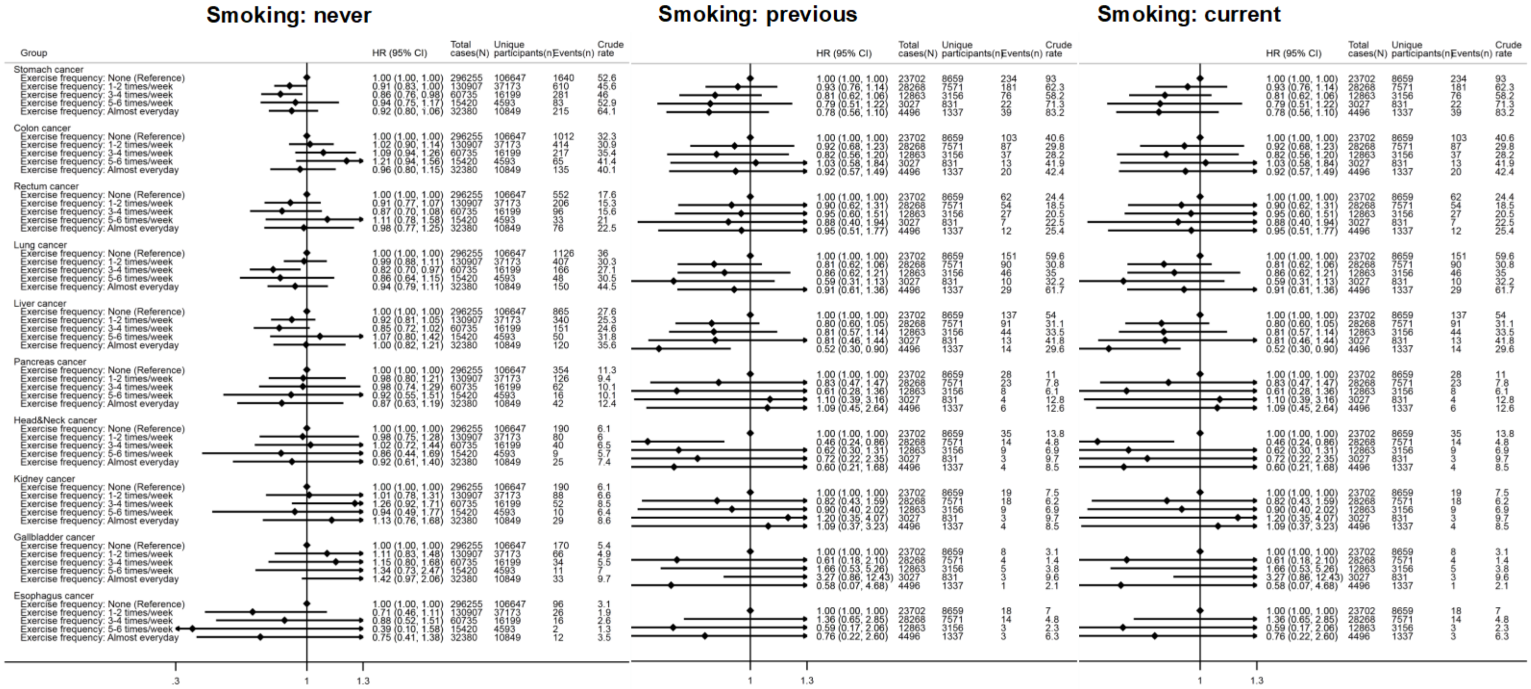

Smoking: never

Group	HR (95% CI)	Total cases(N)	Unique participants(n)	Events(n)	Crude rate
Stomach cancer					
Exercise frequency: None (Reference)	1.00 (1.00, 1.00)	296255	106647	1640	52.6
Exercise frequency: 1-2 times/week	0.91 (0.83, 1.00)	130907	37173	610	45.6
Exercise frequency: 3-4 times/week	0.86 (0.76, 0.98)	60735	16199	281	46
Exercise frequency: 5-6 times/week	0.94 (0.75, 1.17)	15420	4593	83	52.9
Exercise frequency: Almost everyday	0.92 (0.80, 1.06)	32380	10849	215	64.1
Colon cancer					
Exercise frequency: None (Reference)	1.00 (1.00, 1.00)	296255	106647	1012	32.3
Exercise frequency: 1-2 times/week	1.02 (0.90, 1.14)	130907	37173	414	30.9
Exercise frequency: 3-4 times/week	1.09 (0.94, 1.26)	60735	16199	217	35.4
Exercise frequency: 5-6 times/week	1.21 (0.94, 1.56)	15420	4593	65	41.4
Exercise frequency: Almost everyday	0.96 (0.80, 1.15)	32380	10849	135	40.1
Rectum cancer					
Exercise frequency: None (Reference)	1.00 (1.00, 1.00)	296255	106647	552	17.6
Exercise frequency: 1-2 times/week	0.91 (0.77, 1.07)	130907	37173	206	15.3
Exercise frequency: 3-4 times/week	0.87 (0.70, 1.08)	60735	16199	96	15.6
Exercise frequency: 5-6 times/week	1.11 (0.78, 1.58)	15420	4593	33	21
Exercise frequency: Almost everyday	0.98 (0.77, 1.25)	32380	10849	76	22.5
Lung cancer					
Exercise frequency: None (Reference)	1.00 (1.00, 1.00)	296255	106647	1126	36
Exercise frequency: 1-2 times/week	0.99 (0.88, 1.11)	130907	37173	407	30.3
Exercise frequency: 3-4 times/week	0.82 (0.70, 0.97)	60735	16199	166	27.1
Exercise frequency: 5-6 times/week	0.86 (0.64, 1.15)	15420	4593	48	30.5
Exercise frequency: Almost everyday	0.94 (0.79, 1.11)	32380	10849	150	44.5
Liver cancer					
Exercise frequency: None (Reference)	1.00 (1.00, 1.00)	296255	106647	865	27.6
Exercise frequency: 1-2 times/week	0.92 (0.81, 1.05)	130907	37173	340	25.3
Exercise frequency: 3-4 times/week	0.85 (0.72, 1.02)	60735	16199	151	24.6
Exercise frequency: 5-6 times/week	1.07 (0.80, 1.42)	15420	4593	50	31.8
Exercise frequency: Almost everyday	1.00 (0.82, 1.21)	32380	10849	120	35.6
Pancreas cancer					
Exercise frequency: None (Reference)	1.00 (1.00, 1.00)	296255	106647	354	11.3
Exercise frequency: 1-2 times/week	0.98 (0.80, 1.21)	130907	37173	126	9.4
Exercise frequency: 3-4 times/week	0.98 (0.74, 1.29)	60735	16199	62	10.1
Exercise frequency: 5-6 times/week	0.92 (0.55, 1.51)	15420	4593	16	10.1
Exercise frequency: Almost everyday	0.87 (0.63, 1.19)	32380	10849	42	12.4
Head&Neck cancer					
Exercise frequency: None (Reference)	1.00 (1.00, 1.00)	296255	106647	190	6.1
Exercise frequency: 1-2 times/week	0.98 (0.75, 1.28)	130907	37173	80	6
Exercise frequency: 3-4 times/week	1.02 (0.72, 1.44)	60735	16199	40	6.5
Exercise frequency: 5-6 times/week	0.86 (0.44, 1.69)	15420	4593	9	5.7
Exercise frequency: Almost everyday	0.92 (0.61, 1.40)	32380	10849	25	7.4
Kidney cancer					
Exercise frequency: None (Reference)	1.00 (1.00, 1.00)	296255	106647	190	6.1
Exercise frequency: 1-2 times/week	1.01 (0.78, 1.31)	130907	37173	88	6.6
Exercise frequency: 3-4 times/week	1.26 (0.92, 1.71)	60735	16199	52	8.5
Exercise frequency: 5-6 times/week	0.94 (0.49, 1.77)	15420	4593	10	6.4
Exercise frequency: Almost everyday	1.13 (0.76, 1.68)	32380	10849	29	8.6
Gallbladder cancer					
Exercise frequency: None (Reference)	1.00 (1.00, 1.00)	296255	106647	170	5.4
Exercise frequency: 1-2 times/week	1.11 (0.83, 1.48)	130907	37173	66	4.9
Exercise frequency: 3-4 times/week	1.15 (0.80, 1.68)	60735	16199	34	5.5
Exercise frequency: 5-6 times/week	1.34 (0.73, 2.47)	15420	4593	11	7
Exercise frequency: Almost everyday	1.42 (0.97, 2.06)	32380	10849	33	9.7
Esophagus cancer					
Exercise frequency: None (Reference)	1.00 (1.00, 1.00)	296255	106647	96	3.1
Exercise frequency: 1-2 times/week	0.71 (0.46, 1.11)	130907	37173	26	1.9
Exercise frequency: 3-4 times/week	0.88 (0.52, 1.51)	60735	16199	16	2.6
Exercise frequency: 5-6 times/week	0.39 (0.10, 1.58)	15420	4593	2	1.3
Exercise frequency: Almost everyday	0.75 (0.41, 1.38)	32380	10849	12	3.5

Smoking: previous

HR (95% CI)	Total cases(N)	Unique participants(n)	Events(n)	Crude rate
1.00 (1.00, 1.00)	23702	8659	234	93
0.93 (0.76, 1.14)	28268	7571	181	62.3
0.81 (0.62, 1.06)	12863	3156	76	58.2
0.79 (0.51, 1.22)	3027	831	22	71.3
0.78 (0.56, 1.10)	4496	1337	39	83.2
1.00 (1.00, 1.00)	23702	8659	103	40.6
0.92 (0.68, 1.23)	28268	7571	87	29.8
0.82 (0.56, 1.20)	12863	3156	37	28.2
1.03 (0.58, 1.84)	3027	831	13	41.9
0.92 (0.57, 1.49)	4496	1337	20	42.4
1.00 (1.00, 1.00)	23702	8659	62	24.4
0.90 (0.62, 1.31)	28268	7571	54	18.5
0.95 (0.60, 1.51)	12863	3156	27	20.5
0.88 (0.40, 1.94)	3027	831	7	22.5
0.95 (0.51, 1.77)	4496	1337	12	25.4
1.00 (1.00, 1.00)	23702	8659	151	59.6
0.81 (0.62, 1.06)	28268	7571	90	30.8
0.86 (0.62, 1.21)	12863	3156	46	35
0.59 (0.31, 1.13)	3027	831	10	32.2
0.91 (0.61, 1.36)	4496	1337	29	61.7
1.00 (1.00, 1.00)	23702	8659	137	54
0.80 (0.60, 1.05)	28268	7571	91	31.1
0.81 (0.57, 1.14)	12863	3156	44	33.5
0.81 (0.46, 1.44)	3027	831	13	41.8
0.52 (0.30, 0.90)	4496	1337	14	29.6
1.00 (1.00, 1.00)	23702	8659	28	11
0.83 (0.47, 1.47)	28268	7571	23	7.8
0.61 (0.28, 1.36)	12863	3156	8	6.1
1.10 (0.39, 3.16)	3027	831	4	12.8
1.09 (0.45, 2.64)	4496	1337	6	12.6
1.00 (1.00, 1.00)	23702	8659	35	13.8
0.46 (0.24, 0.86)	28268	7571	14	4.8
0.62 (0.30, 1.31)	12863	3156	9	6.9
0.72 (0.22, 2.35)	3027	831	3	9.7
0.60 (0.21, 1.68)	4496	1337	4	8.5
1.00 (1.00, 1.00)	23702	8659	19	7.5
0.82 (0.43, 1.59)	28268	7571	18	6.2
0.90 (0.40, 2.02)	12863	3156	9	6.9
1.20 (0.35, 4.07)	3027	831	3	9.7
1.09 (0.37, 3.23)	4496	1337	4	8.5
1.00 (1.00, 1.00)	23702	8659	8	3.1
0.61 (0.18, 2.10)	28268	7571	4	1.4
1.66 (0.53, 5.26)	12863	3156	5	3.8
3.27 (0.86, 12.43)	3027	831	3	9.6
0.58 (0.07, 4.68)	4496	1337	1	2.1
1.00 (1.00, 1.00)	23702	8659	18	7
1.36 (0.65, 2.85)	28268	7571	14	4.8
0.59 (0.17, 2.06)	12863	3156	3	2.3
0.76 (0.22, 2.60)	4496	1337	3	6.3

Smoking: current

HR (95% CI)	Total cases(N)	Unique participants(n)	Events(n)	Crude rate
1.00 (1.00, 1.00)	23702	8659	234	93
0.93 (0.76, 1.14)	28268	7571	181	62.3
0.81 (0.62, 1.06)	12863	3156	76	58.2
0.79 (0.51, 1.22)	3027	831	22	71.3
0.78 (0.56, 1.10)	4496	1337	39	83.2
1.00 (1.00, 1.00)	23702	8659	103	40.6
0.92 (0.68, 1.23)	28268	7571	87	29.8
0.82 (0.56, 1.20)	12863	3156	37	28.2
1.03 (0.58, 1.84)	3027	831	13	41.9
0.92 (0.57, 1.49)	4496	1337	20	42.4
1.00 (1.00, 1.00)	23702	8659	62	24.4
0.90 (0.62, 1.31)	28268	7571	54	18.5
0.95 (0.60, 1.51)	12863	3156	27	20.5
0.88 (0.40, 1.94)	3027	831	7	22.5
0.95 (0.51, 1.77)	4496	1337	12	25.4
1.00 (1.00, 1.00)	23702	8659	151	59.6
0.81 (0.62, 1.06)	28268	7571	90	30.8
0.86 (0.62, 1.21)	12863	3156	46	35
0.59 (0.31, 1.13)	3027	831	10	32.2
0.91 (0.61, 1.36)	4496	1337	29	61.7
1.00 (1.00, 1.00)	23702	8659	137	54
0.80 (0.60, 1.05)	28268	7571	91	31.1
0.81 (0.57, 1.14)	12863	3156	44	33.5
0.81 (0.46, 1.44)	3027	831	13	41.8
0.52 (0.30, 0.90)	4496	1337	14	29.6
1.00 (1.00, 1.00)	23702	8659	28	11
0.83 (0.47, 1.47)	28268	7571	23	7.8
0.61 (0.28, 1.36)	12863	3156	8	6.1
1.10 (0.39, 3.16)	3027	831	4	12.8
1.09 (0.45, 2.64)	4496	1337	6	12.6
1.00 (1.00, 1.00)	23702	8659	35	13.8
0.46 (0.24, 0.86)	28268	7571	14	4.8
0.62 (0.30, 1.31)	12863	3156	9	6.9
0.72 (0.22, 2.35)	3027	831	3	9.7
0.60 (0.21, 1.68)	4496	1337	4	8.5
1.00 (1.00, 1.00)	23702	8659	19	7.5
0.82 (0.43, 1.59)	28268	7571	18	6.2
0.90 (0.40, 2.02)	12863	3156	9	6.9
1.20 (0.35, 4.07)	3027	831	3	9.7
1.09 (0.37, 3.23)	4496	1337	4	8.5
1.00 (1.00, 1.00)	23702	8659	8	3.1
0.61 (0.18, 2.10)	28268	7571	4	1.4
1.66 (0.53, 5.26)	12863	3156	5	3.8
3.27 (0.86, 12.43)	3027	831	3	9.6
0.58 (0.07, 4.68)	4496	1337	1	2.1
1.00 (1.00, 1.00)	23702	8659	18	7
1.36 (0.65, 2.85)	28268	7571	14	4.8
0.59 (0.17, 2.06)	12863	3156	3	2.3
0.76 (0.22, 2.60)	4496	1337	3	6.3

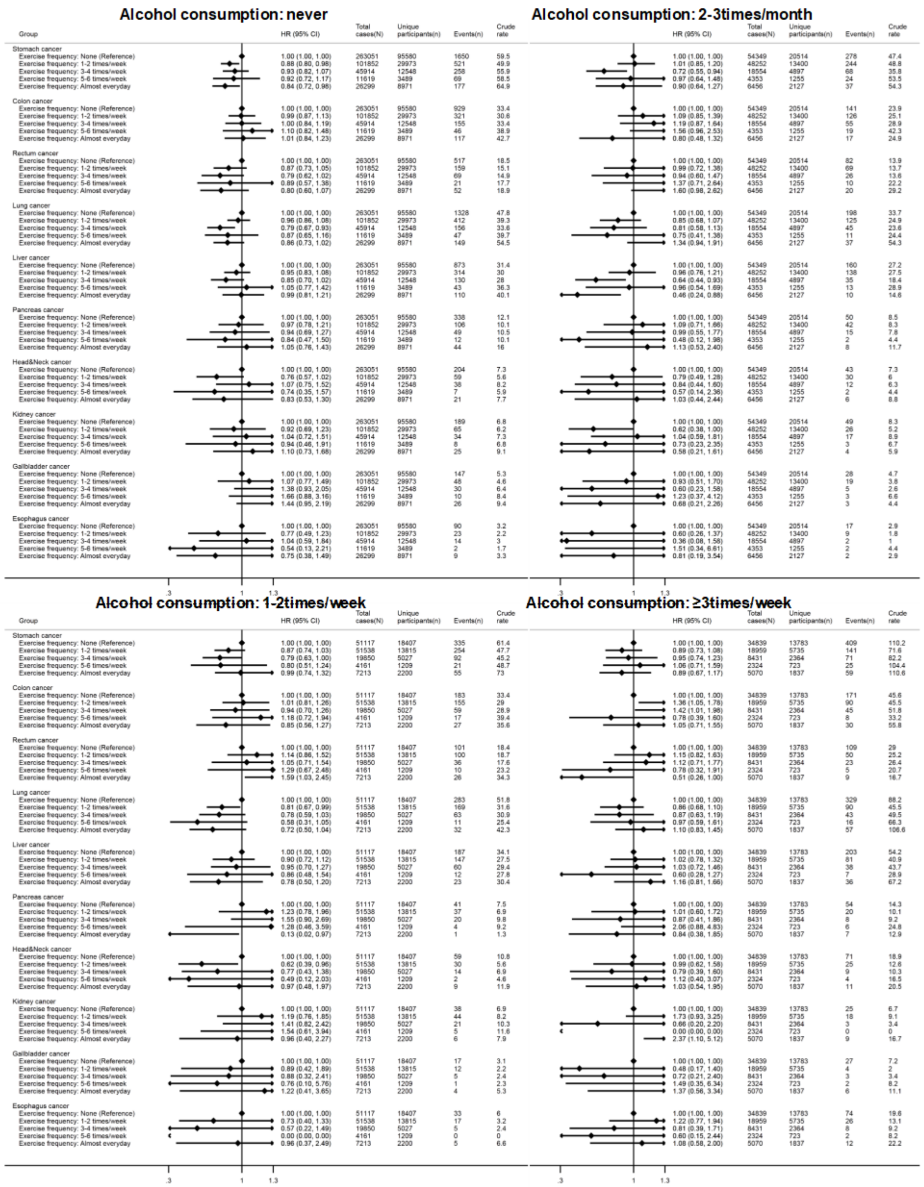
Alcohol consumption: never
Group HR (95% CI) Total cases(N) Unique participants(n) Events(n) Crude rate
Stomach cancer
Exercise frequency: None (Reference) 1.00 (1.00, 1.00) 263051 95580 1650 59.5
Exercise frequency: 1-2 times/week 0.88 (0.80, 0.98) 101852 29973 521 49.9
Exercise frequency: 3-4 times/week 0.93 (0.82, 1.07) 45914 12548 258 55.9
Exercise frequency: 5-6 times/week 0.92 (0.72, 1.17) 11619 3489 69 58.5
Exercise frequency: Almost everyday 0.84 (0.72, 0.98) 26299 8971 177 64.9
Colon cancer
Exercise frequency: None (Reference) 1.00 (1.00, 1.00) 263051 95580 929 33.4
Exercise frequency: 1-2 times/week 0.99 (0.87, 1.13) 101852 29973 321 30.6
Exercise frequency: 3-4 times/week 1.00 (0.84, 1.19) 45914 12548 155 33.4
Exercise frequency: 5-6 times/week 1.10 (0.82, 1.48) 11619 3489 46 38.9
Exercise frequency: Almost everyday 1.01 (0.84, 1.23) 26299 8971 117 42.7
Rectum cancer
Exercise frequency: None (Reference) 1.00 (1.00, 1.00) 263051 95580 517 18.5
Exercise frequency: 1-2 times/week 0.87 (0.73, 1.05) 101852 29973 159 15.1
Exercise frequency: 3-4 times/week 0.79 (0.62, 1.02) 45914 12548 69 14.9
Exercise frequency: 5-6 times/week 0.89 (0.57, 1.38) 11619 3489 21 17.7
Exercise frequency: Almost everyday 0.80 (0.60, 1.07) 26299 8971 52 18.9
Lung cancer
Exercise frequency: None (Reference) 1.00 (1.00, 1.00) 263051 95580 1328 47.8
Exercise frequency: 1-2 times/week 0.96 (0.86, 1.08) 101852 29973 412 39.3
Exercise frequency: 3-4 times/week 0.79 (0.67, 0.93) 45914 12548 156 33.6
Exercise frequency: 5-6 times/week 0.87 (0.65, 1.16) 11619 3489 47 39.7
Exercise frequency: Almost everyday 0.86 (0.73, 1.02) 26299 8971 149 54.5
Liver cancer
Exercise frequency: None (Reference) 1.00 (1.00, 1.00) 263051 95580 873 31.4
Exercise frequency: 1-2 times/week 0.95 (0.83, 1.08) 101852 29973 314 30
Exercise frequency: 3-4 times/week 0.85 (0.70, 1.02) 45914 12548 130 28
Exercise frequency: 5-6 times/week 1.05 (0.77, 1.42) 11619 3489 43 36.3
Exercise frequency: Almost everyday 0.99 (0.81, 1.21) 26299 8971 110 40.1
Pancreas cancer
Exercise frequency: None (Reference) 1.00 (1.00, 1.00) 263051 95580 338 12.1
Exercise frequency: 1-2 times/week 0.97 (0.78, 1.21) 101852 29973 106 10.1
Exercise frequency: 3-4 times/week 0.94 (0.69, 1.27) 45914 12548 49 10.5
Exercise frequency: 5-6 times/week 0.84 (0.47, 1.50) 11619 3489 12 10.1
Exercise frequency: Almost everyday 1.05 (0.76, 1.43) 26299 8971 44 16
Head&Neck cancer
Exercise frequency: None (Reference) 1.00 (1.00, 1.00) 263051 95580 204 7.3
Exercise frequency: 1-2 times/week 0.76 (0.57, 1.02) 101852 29973 59 5.6
Exercise frequency: 3-4 times/week 1.07 (0.75, 1.52) 45914 12548 38 8.2
Exercise frequency: 5-6 times/week 0.74 (0.35, 1.57) 11619 3489 7 5.9
Exercise frequency: Almost everyday 0.83 (0.53, 1.30) 26299 8971 21 7.7
Kidney cancer
Exercise frequency: None (Reference) 1.00 (1.00, 1.00) 263051 95580 189 6.8
Exercise frequency: 1-2 times/week 0.92 (0.69, 1.23) 101852 29973 65 6.2
Exercise frequency: 3-4 times/week 1.04 (0.72, 1.51) 45914 12548 34 7.3
Exercise frequency: 5-6 times/week 0.94 (0.46, 1.91) 11619 3489 8 6.8
Exercise frequency: Almost everyday 1.10 (0.73, 1.68) 26299 8971 25 9.1
Gallbladder cancer
Exercise frequency: None (Reference) 1.00 (1.00, 1.00) 263051 95580 147 5.3
Exercise frequency: 1-2 times/week 1.07 (0.77, 1.49) 101852 29973 48 4.6
Exercise frequency: 3-4 times/week 1.38 (0.93, 2.05) 45914 12548 30 6.4
Exercise frequency: 5-6 times/week 1.66 (0.88, 3.16) 11619 3489 10 8.4
Exercise frequency: Almost everyday 1.44 (0.95, 2.19) 26299 8971 26 9.4
Esophagus cancer
Exercise frequency: None (Reference) 1.00 (1.00, 1.00) 263051 95580 90 3.2
Exercise frequency: 1-2 times/week 0.77 (0.49, 1.23) 101852 29973 23 2.2
Exercise frequency: 3-4 times/week 1.04 (0.59, 1.84) 45914 12548 14 3
Exercise frequency: 5-6 times/week 0.54 (0.13, 2.21) 11619 3489 2 1.7
Exercise frequency: Almost everyday 0.75 (0.38, 1.49) 26299 8971 9 3.3
.3 1 1.3
Alcohol consumption: 2-3times/month
HR (95% CI) Total cases(N) Unique participants(n) Events(n) Crude rate
1.00 (1.00, 1.00) 54349 20514 278 47.4
1.01 (0.85, 1.20) 48252 13400 244 48.8
0.72 (0.55, 0.94) 18554 4897 68 35.8
0.97 (0.64, 1.48) 4353 1255 24 53.5
0.90 (0.64, 1.27) 6456 2127 37 54.3
1.00 (1.00, 1.00) 54349 20514 141 23.9
1.09 (0.85, 1.39) 48252 13400 126 25.1
1.19 (0.87, 1.64) 18554 4897 55 28.9
1.56 (0.96, 2.53) 4353 1255 19 42.3
0.80 (0.48, 1.32) 6456 2127 17 24.9
1.00 (1.00, 1.00) 54349 20514 82 13.9
0.99 (0.72, 1.38) 48252 13400 69 13.7
0.94 (0.60, 1.47) 18554 4897 26 13.6
1.37 (0.71, 2.64) 4353 1255 10 22.2
1.60 (0.98, 2.62) 6456 2127 20 29.2
1.00 (1.00, 1.00) 54349 20514 198 33.7
0.85 (0.68, 1.07) 48252 13400 125 24.9
0.81 (0.58, 1.13) 18554 4897 45 23.6
0.75 (0.41, 1.38) 4353 1255 11 24.4
1.34 (0.94, 1.91) 6456 2127 37 54.3
1.00 (1.00, 1.00) 54349 20514 160 27.2
0.96 (0.76, 1.21) 48252 13400 138 27.5
0.64 (0.44, 0.93) 18554 4897 35 18.4
0.96 (0.54, 1.69) 4353 1255 13 28.9
0.46 (0.24, 0.88) 6456 2127 10 14.6
1.00 (1.00, 1.00) 54349 20514 50 8.5
1.09 (0.71, 1.66) 48252 13400 42 8.3
0.99 (0.55, 1.77) 18554 4897 15 7.8
0.48 (0.12, 1.98) 4353 1255 2 4.4
1.13 (0.53, 2.40) 6456 2127 8 11.7
1.00 (1.00, 1.00) 54349 20514 43 7.3
0.79 (0.49, 1.28) 48252 13400 30 6
0.84 (0.44, 1.60) 18554 4897 12 6.3
0.57 (0.14, 2.36) 4353 1255 2 4.4
1.03 (0.44, 2.44) 6456 2127 6 8.8
1.00 (1.00, 1.00) 54349 20514 49 8.3
0.62 (0.38, 1.00) 48252 13400 26 5.2
1.04 (0.59, 1.81) 18554 4897 17 8.9
0.73 (0.23, 2.35) 4353 1255 3 6.7
0.58 (0.21, 1.61) 6456 2127 4 5.9
1.00 (1.00, 1.00) 54349 20514 28 4.7
0.93 (0.51, 1.70) 48252 13400 19 3.8
0.60 (0.23, 1.58) 18554 4897 5 2.6
1.23 (0.37, 4.12) 4353 1255 3 6.6
0.68 (0.21, 2.26) 6456 2127 3 4.4
1.00 (1.00, 1.00) 54349 20514 17 2.9
0.60 (0.26, 1.37) 48252 13400 9 1.8
0.36 (0.08, 1.58) 18554 4897 2 1
1.51 (0.34, 6.61) 4353 1255 2 4.4
0.81 (0.19, 3.54) 6456 2127 2 2.9
.3 1 1.3
Alcohol consumption: 1-2times/week
Group HR (95% CI) Total cases(N) Unique participants(n) Events(n) Crude rate
Stomach cancer
Exercise frequency: None (Reference) 1.00 (1.00, 1.00) 51117 18407 335 61.4
Exercise frequency: 1-2 times/week 0.87 (0.74, 1.03) 51538 13815 254 47.7
Exercise frequency: 3-4 times/week 0.79 (0.63, 1.00) 19850 5027 92 45.2
Exercise frequency: 5-6 times/week 0.80 (0.51, 1.24) 4161 1209 21 48.7
Exercise frequency: Almost everyday 0.99 (0.74, 1.32) 7213 2200 55 73
Colon cancer
Exercise frequency: None (Reference) 1.00 (1.00, 1.00) 51117 18407 183 33.4
Exercise frequency: 1-2 times/week 1.01 (0.81, 1.26) 51538 13815 155 29
Exercise frequency: 3-4 times/week 0.94 (0.70, 1.26) 19850 5027 59 28.9
Exercise frequency: 5-6 times/week 1.18 (0.72, 1.94) 4161 1209 17 39.4
Exercise frequency: Almost everyday 0.85 (0.56, 1.27) 7213 2200 27 35.6
Rectum cancer
Exercise frequency: None (Reference) 1.00 (1.00, 1.00) 51117 18407 101 18.4
Exercise frequency: 1-2 times/week 1.14 (0.86, 1.52) 51538 13815 100 18.7
Exercise frequency: 3-4 times/week 1.05 (0.71, 1.54) 19850 5027 36 17.6
Exercise frequency: 5-6 times/week 1.29 (0.67, 2.48) 4161 1209 10 23.2
Exercise frequency: Almost everyday 1.59 (1.03, 2.45) 7213 2200 26 34.3
Lung cancer
Exercise frequency: None (Reference) 1.00 (1.00, 1.00) 51117 18407 283 51.8
Exercise frequency: 1-2 times/week 0.81 (0.67, 0.99) 51538 13815 169 31.6
Exercise frequency: 3-4 times/week 0.78 (0.59, 1.03) 19850 5027 63 30.9
Exercise frequency: 5-6 times/week 0.58 (0.31, 1.05) 4161 1209 11 25.4
Exercise frequency: Almost everyday 0.72 (0.50, 1.04) 7213 2200 32 42.3
Liver cancer
Exercise frequency: None (Reference) 1.00 (1.00, 1.00) 51117 18407 187 34.1
Exercise frequency: 1-2 times/week 0.90 (0.72, 1.12) 51538 13815 147 27.5
Exercise frequency: 3-4 times/week 0.95 (0.70, 1.27) 19850 5027 60 29.4
Exercise frequency: 5-6 times/week 0.86 (0.48, 1.54) 4161 1209 12 27.8
Exercise frequency: Almost everyday 0.78 (0.50, 1.20) 7213 2200 23 30.4
Pancreas cancer
Exercise frequency: None (Reference) 1.00 (1.00, 1.00) 51117 18407 41 7.5
Exercise frequency: 1-2 times/week 1.23 (0.78, 1.96) 51538 13815 37 6.9
Exercise frequency: 3-4 times/week 1.55 (0.90, 2.69) 19850 5027 20 9.8
Exercise frequency: 5-6 times/week 1.28 (0.46, 3.59) 4161 1209 4 9.2
Exercise frequency: Almost everyday 0.13 (0.02, 0.97) 7213 2200 1 1.3
Head&Neck cancer
Exercise frequency: None (Reference) 1.00 (1.00, 1.00) 51117 18407 59 10.8
Exercise frequency: 1-2 times/week 0.62 (0.39, 0.96) 51538 13815 30 5.6
Exercise frequency: 3-4 times/week 0.77 (0.43, 1.38) 19850 5027 14 6.9
Exercise frequency: 5-6 times/week 0.49 (0.12, 2.03) 4161 1209 2 4.6
Exercise frequency: Almost everyday 0.97 (0.48, 1.97) 7213 2200 9 11.9
Kidney cancer
Exercise frequency: None (Reference) 1.00 (1.00, 1.00) 51117 18407 38 6.9
Exercise frequency: 1-2 times/week 1.19 (0.76, 1.85) 51538 13815 44 8.2
Exercise frequency: 3-4 times/week 1.41 (0.82, 2.42) 19850 5027 21 10.3
Exercise frequency: 5-6 times/week 1.54 (0.61, 3.94) 4161 1209 5 11.6
Exercise frequency: Almost everyday 0.96 (0.40, 2.27) 7213 2200 6 7.9
Gallbladder cancer
Exercise frequency: None (Reference) 1.00 (1.00, 1.00) 51117 18407 17 3.1
Exercise frequency: 1-2 times/week 0.89 (0.42, 1.89) 51538 13815 12 2.2
Exercise frequency: 3-4 times/week 0.88 (0.32, 2.41) 19850 5027 5 2.4
Exercise frequency: 5-6 times/week 0.76 (0.10, 5.76) 4161 1209 1 2.3
Exercise frequency: Almost everyday 1.22 (0.41, 3.65) 7213 2200 4 5.3
Esophagus cancer
Exercise frequency: None (Reference) 1.00 (1.00, 1.00) 51117 18407 33 6
Exercise frequency: 1-2 times/week 0.73 (0.40, 1.33) 51538 13815 17 3.2
Exercise frequency: 3-4 times/week 0.57 (0.22, 1.49) 19850 5027 5 2.4
Exercise frequency: 5-6 times/week 0.00 (0.00, 0.00) 4161 1209 0 0
Exercise frequency: Almost everyday 0.96 (0.37, 2.49) 7213 2200 5 6.6
.3 1 1.3
Alcohol consumption: ≥3times/week
HR (95% CI) Total cases(N) Unique participants(n) Events(n) Crude rate
1.00 (1.00, 1.00) 34839 13783 409 110.2
0.89 (0.73, 1.08) 18959 5735 141 71.6
0.95 (0.74, 1.23) 8431 2364 71 82.2
1.06 (0.71, 1.59) 2324 723 25 104.4
0.89 (0.67, 1.17) 5070 1837 59 110.6
1.00 (1.00, 1.00) 34839 13783 171 45.6
1.36 (1.05, 1.78) 18959 5735 90 45.5
1.42 (1.01, 1.98) 8431 2364 45 51.8
0.78 (0.39, 1.60) 2324 723 8 33.2
1.05 (0.71, 1.55) 5070 1837 30 55.8
1.00 (1.00, 1.00) 34839 13783 109 29
1.15 (0.82, 1.63) 18959 5735 50 25.2
1.12 (0.71, 1.77) 8431 2364 23 26.4
0.78 (0.32, 1.91) 2324 723 5 20.7
0.51 (0.26, 1.00) 5070 1837 9 16.7
1.00 (1.00, 1.00) 34839 13783 329 88.2
0.86 (0.68, 1.10) 18959 5735 90 45.5
0.87 (0.63, 1.19) 8431 2364 43 49.5
0.97 (0.59, 1.61) 2324 723 16 66.3
1.10 (0.83, 1.45) 5070 1837 57 106.6
1.00 (1.00, 1.00) 34839 13783 203 54.2
1.02 (0.78, 1.32) 18959 5735 81 40.9
1.03 (0.72, 1.46) 8431 2364 38 43.7
0.60 (0.28, 1.27) 2324 723 7 28.9
1.16 (0.81, 1.66) 5070 1837 36 67.2
1.00 (1.00, 1.00) 34839 13783 54 14.3
1.01 (0.60, 1.72) 18959 5735 20 10.1
0.87 (0.41, 1.86) 8431 2364 8 9.2
2.06 (0.88, 4.83) 2324 723 6 24.8
0.84 (0.38, 1.85) 5070 1837 7 12.9
1.00 (1.00, 1.00) 34839 13783 71 18.9
0.99 (0.62, 1.58) 18959 5735 25 12.6
0.79 (0.39, 1.60) 8431 2364 9 10.3
1.12 (0.40, 3.07) 2324 723 4 16.5
1.03 (0.54, 1.95) 5070 1837 11 20.5
1.00 (1.00, 1.00) 34839 13783 25 6.7
1.73 (0.93, 3.25) 18959 5735 18 9.1
0.66 (0.20, 2.20) 8431 2364 3 3.4
0.00 (0.00, 0.00) 2324 723 0 0
2.37 (1.10, 5.12) 5070 1837 9 16.7
1.00 (1.00, 1.00) 34839 13783 27 7.2
0.48 (0.17, 1.40) 18959 5735 4 2
0.72 (0.21, 2.40) 8431 2364 3 3.4
1.49 (0.35, 6.34) 2324 723 2 8.2
1.37 (0.56, 3.34) 5070 1837 6 11.1
1.00 (1.00, 1.00) 34839 13783 74 19.6
1.22 (0.77, 1.94) 18959 5735 26 13.1
0.81 (0.39, 1.71) 8431 2364 8 9.2
0.60 (0.15, 2.44) 2324 723 2 8.2
1.08 (0.58, 2.00) 5070 1837 12 22.2
.3 1 1.3

No family history of cancer

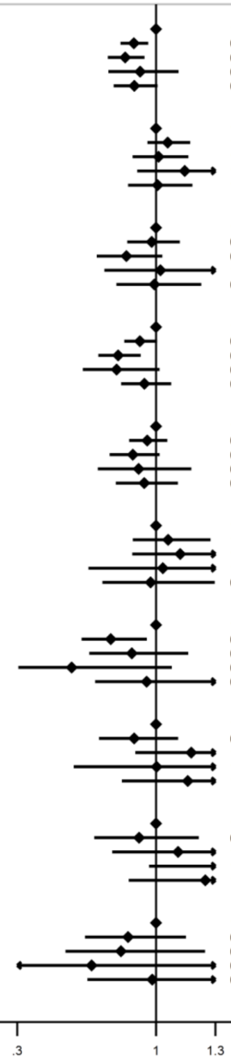

Group	HR (95% CI)	Total cases(N)	Unique participants(n)	Events(n)	Crude rate
Stomach cancer					
Exercise frequency: None (Reference)	1.00 (1.00, 1.00)	351171	128853	2325	62.4
Exercise frequency: 1-2 times/week	0.89 (0.82, 0.96)	186251	53323	937	48.7
Exercise frequency: 3-4 times/week	0.84 (0.76, 0.94)	76877	20575	384	49.1
Exercise frequency: 5-6 times/week	0.92 (0.76, 1.11)	18364	5571	111	58.8
Exercise frequency: Almost everyday	0.89 (0.79, 1.01)	38451	12944	282	70.2
Colon cancer					
Exercise frequency: None (Reference)	1.00 (1.00, 1.00)	351171	128853	1247	33.3
Exercise frequency: 1-2 times/week	1.06 (0.96, 1.17)	186251	53323	583	30.2
Exercise frequency: 3-4 times/week	1.01 (0.88, 1.16)	76877	20575	247	31.5
Exercise frequency: 5-6 times/week	1.14 (0.90, 1.45)	18364	5571	74	39.1
Exercise frequency: Almost everyday	1.01 (0.86, 1.18)	38451	12944	171	42.4
Rectum cancer					
Exercise frequency: None (Reference)	1.00 (1.00, 1.00)	351171	128853	715	19.1
Exercise frequency: 1-2 times/week	0.98 (0.85, 1.12)	186251	53323	327	16.9
Exercise frequency: 3-4 times/week	0.85 (0.70, 1.03)	76877	20575	124	15.8
Exercise frequency: 5-6 times/week	1.02 (0.74, 1.41)	18364	5571	39	20.6
Exercise frequency: Almost everyday	0.99 (0.80, 1.23)	38451	12944	96	23.7
Lung cancer					
Exercise frequency: None (Reference)	1.00 (1.00, 1.00)	351171	128853	1908	51.1
Exercise frequency: 1-2 times/week	0.92 (0.84, 1.01)	186251	53323	689	35.7
Exercise frequency: 3-4 times/week	0.81 (0.71, 0.92)	76877	20575	258	32.9
Exercise frequency: 5-6 times/week	0.80 (0.63, 1.02)	18364	5571	70	36.9
Exercise frequency: Almost everyday	0.94 (0.82, 1.08)	38451	12944	243	60.3
Liver cancer					
Exercise frequency: None (Reference)	1.00 (1.00, 1.00)	351171	128853	1271	34
Exercise frequency: 1-2 times/week	0.96 (0.86, 1.06)	186251	53323	588	30.4
Exercise frequency: 3-4 times/week	0.88 (0.77, 1.02)	76877	20575	231	29.4
Exercise frequency: 5-6 times/week	0.91 (0.71, 1.18)	18364	5571	62	32.7
Exercise frequency: Almost everyday	0.94 (0.80, 1.11)	38451	12944	159	39.4
Pancreas cancer					
Exercise frequency: None (Reference)	1.00 (1.00, 1.00)	351171	128853	415	11.1
Exercise frequency: 1-2 times/week	1.06 (0.88, 1.28)	186251	53323	171	8.8
Exercise frequency: 3-4 times/week	1.12 (0.88, 1.43)	76877	20575	80	10.2
Exercise frequency: 5-6 times/week	1.03 (0.66, 1.62)	18364	5571	20	10.5
Exercise frequency: Almost everyday	0.97 (0.73, 1.30)	38451	12944	53	13.1
Head&Neck cancer					
Exercise frequency: None (Reference)	1.00 (1.00, 1.00)	351171	128853	338	9
Exercise frequency: 1-2 times/week	0.77 (0.62, 0.95)	186251	53323	125	6.5
Exercise frequency: 3-4 times/week	0.88 (0.66, 1.16)	76877	20575	59	7.5
Exercise frequency: 5-6 times/week	0.57 (0.31, 1.08)	18364	5571	10	5.3
Exercise frequency: Almost everyday	0.95 (0.69, 1.31)	38451	12944	43	10.6
Kidney cancer					
Exercise frequency: None (Reference)	1.00 (1.00, 1.00)	351171	128853	264	7.1
Exercise frequency: 1-2 times/week	0.89 (0.71, 1.11)	186251	53323	120	6.2
Exercise frequency: 3-4 times/week	1.18 (0.90, 1.55)	76877	20575	66	8.4
Exercise frequency: 5-6 times/week	1.00 (0.59, 1.72)	18364	5571	14	7.4
Exercise frequency: Almost everyday	1.16 (0.83, 1.63)	38451	12944	39	9.7
Gallbladder cancer					
Exercise frequency: None (Reference)	1.00 (1.00, 1.00)	351171	128853	200	5.3
Exercise frequency: 1-2 times/week	0.91 (0.69, 1.22)	186251	53323	67	3.5
Exercise frequency: 3-4 times/week	1.11 (0.78, 1.59)	76877	20575	37	4.7
Exercise frequency: 5-6 times/week	1.63 (0.96, 2.77)	18364	5571	15	7.9
Exercise frequency: Almost everyday	1.25 (0.86, 1.81)	38451	12944	33	8.1
Esophagus cancer					
Exercise frequency: None (Reference)	1.00 (1.00, 1.00)	351171	128853	193	5.1
Exercise frequency: 1-2 times/week	0.86 (0.64, 1.15)	186251	53323	64	3.3
Exercise frequency: 3-4 times/week	0.82 (0.54, 1.25)	76877	20575	26	3.3
Exercise frequency: 5-6 times/week	0.68 (0.30, 1.53)	18364	5571	6	3.2
Exercise frequency: Almost everyday	0.98 (0.65, 1.47)	38451	12944	27	6.7

Family history of cancer

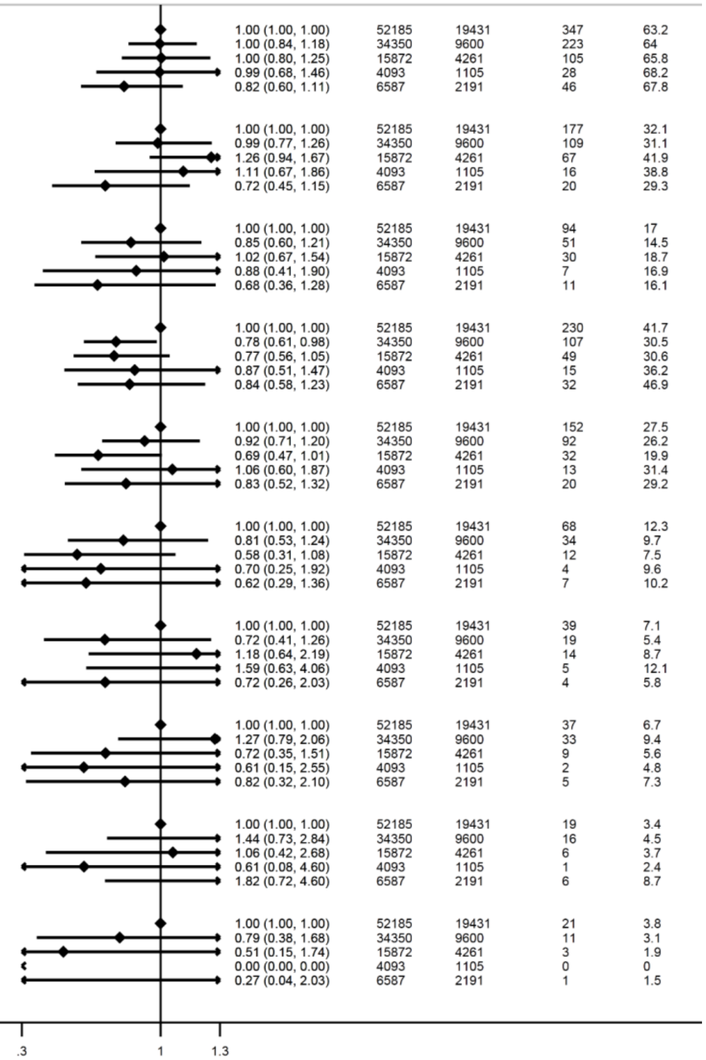

Group	HR (95% CI)	Total cases(N)	Unique participants(n)	Events(n)	Crude rate
Stomach cancer					
Exercise frequency: None (Reference)	1.00 (1.00, 1.00)	52185	19431	347	63.2
Exercise frequency: 1-2 times/week	1.00 (0.84, 1.18)	34350	9600	223	64
Exercise frequency: 3-4 times/week	1.00 (0.80, 1.25)	15872	4261	105	65.8
Exercise frequency: 5-6 times/week	0.99 (0.68, 1.46)	4093	1105	28	68.2
Exercise frequency: Almost everyday	0.82 (0.60, 1.11)	6587	2191	46	67.8
Colon cancer					
Exercise frequency: None (Reference)	1.00 (1.00, 1.00)	52185	19431	177	32.1
Exercise frequency: 1-2 times/week	0.99 (0.77, 1.26)	34350	9600	109	31.1
Exercise frequency: 3-4 times/week	1.26 (0.94, 1.67)	15872	4261	67	41.9
Exercise frequency: 5-6 times/week	1.11 (0.67, 1.86)	4093	1105	16	38.8
Exercise frequency: Almost everyday	0.72 (0.45, 1.15)	6587	2191	20	29.3
Rectum cancer					
Exercise frequency: None (Reference)	1.00 (1.00, 1.00)	52185	19431	94	17
Exercise frequency: 1-2 times/week	0.85 (0.60, 1.21)	34350	9600	51	14.5
Exercise frequency: 3-4 times/week	1.02 (0.67, 1.54)	15872	4261	30	18.7
Exercise frequency: 5-6 times/week	0.88 (0.41, 1.90)	4093	1105	7	16.9
Exercise frequency: Almost everyday	0.68 (0.36, 1.28)	6587	2191	11	16.1
Lung cancer					
Exercise frequency: None (Reference)	1.00 (1.00, 1.00)	52185	19431	230	41.7
Exercise frequency: 1-2 times/week	0.78 (0.61, 0.98)	34350	9600	107	30.5
Exercise frequency: 3-4 times/week	0.77 (0.56, 1.05)	15872	4261	49	30.6
Exercise frequency: 5-6 times/week	0.87 (0.51, 1.47)	4093	1105	15	36.2
Exercise frequency: Almost everyday	0.84 (0.58, 1.23)	6587	2191	32	46.9
Liver cancer					
Exercise frequency: None (Reference)	1.00 (1.00, 1.00)	52185	19431	152	27.5
Exercise frequency: 1-2 times/week	0.92 (0.71, 1.20)	34350	9600	92	26.2
Exercise frequency: 3-4 times/week	0.69 (0.47, 1.01)	15872	4261	32	19.9
Exercise frequency: 5-6 times/week	1.06 (0.60, 1.87)	4093	1105	13	31.4
Exercise frequency: Almost everyday	0.83 (0.52, 1.32)	6587	2191	20	29.2
Pancreas cancer					
Exercise frequency: None (Reference)	1.00 (1.00, 1.00)	52185	19431	68	12.3
Exercise frequency: 1-2 times/week	0.81 (0.53, 1.24)	34350	9600	34	9.7
Exercise frequency: 3-4 times/week	0.58 (0.31, 1.08)	15872	4261	12	7.5
Exercise frequency: 5-6 times/week	0.70 (0.25, 1.92)	4093	1105	4	9.6
Exercise frequency: Almost everyday	0.62 (0.29, 1.36)	6587	2191	7	10.2
Head&Neck cancer					
Exercise frequency: None (Reference)	1.00 (1.00, 1.00)	52185	19431	39	7.1
Exercise frequency: 1-2 times/week	0.72 (0.41, 1.26)	34350	9600	19	5.4
Exercise frequency: 3-4 times/week	1.18 (0.64, 2.19)	15872	4261	14	8.7
Exercise frequency: 5-6 times/week	1.59 (0.63, 4.06)	4093	1105	5	12.1
Exercise frequency: Almost everyday	0.72 (0.26, 2.03)	6587	2191	4	5.8
Kidney cancer					
Exercise frequency: None (Reference)	1.00 (1.00, 1.00)	52185	19431	37	6.7
Exercise frequency: 1-2 times/week	1.27 (0.79, 2.06)	34350	9600	33	9.4
Exercise frequency: 3-4 times/week	0.72 (0.35, 1.51)	15872	4261	9	5.6
Exercise frequency: 5-6 times/week	0.61 (0.15, 2.55)	4093	1105	2	4.8
Exercise frequency: Almost everyday	0.82 (0.32, 2.10)	6587	2191	5	7.3
Gallbladder cancer					
Exercise frequency: None (Reference)	1.00 (1.00, 1.00)	52185	19431	19	3.4
Exercise frequency: 1-2 times/week	1.44 (0.73, 2.84)	34350	9600	16	4.5
Exercise frequency: 3-4 times/week	1.06 (0.42, 2.68)	15872	4261	6	3.7
Exercise frequency: 5-6 times/week	0.61 (0.08, 4.60)	4093	1105	1	2.4
Exercise frequency: Almost everyday	1.82 (0.72, 4.60)	6587	2191	6	8.7
Esophagus cancer					
Exercise frequency: None (Reference)	1.00 (1.00, 1.00)	52185	19431	21	3.8
Exercise frequency: 1-2 times/week	0.79 (0.38, 1.68)	34350	9600	11	3.1
Exercise frequency: 3-4 times/week	0.51 (0.15, 1.74)	15872	4261	3	1.9
Exercise frequency: 5-6 times/week	0.00 (0.00, 0.00)	4093	1105	0	0
Exercise frequency: Almost everyday	0.27 (0.04, 2.03)	6587	2191	1	1.5

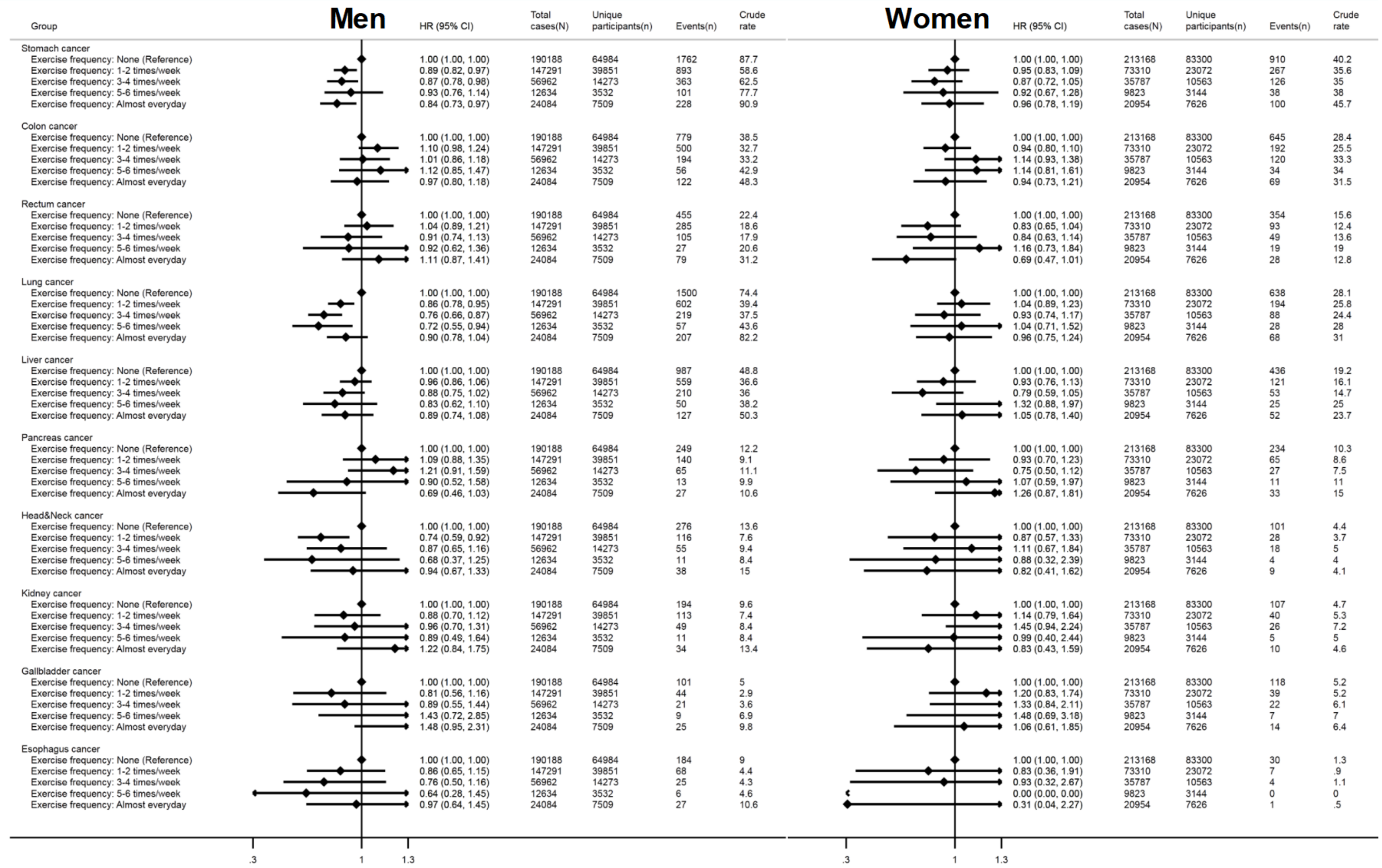

Men

Group	HR (95% CI)	Total cases(N)	Unique participants(n)	Events(n)	Crude rate
Stomach cancer					
Exercise frequency: None (Reference)	1.00 (1.00, 1.00)	190188	64984	1762	87.7
Exercise frequency: 1-2 times/week	0.89 (0.82, 0.97)	147291	39851	893	58.6
Exercise frequency: 3-4 times/week	0.87 (0.78, 0.98)	56962	14273	363	62.5
Exercise frequency: 5-6 times/week	0.93 (0.76, 1.14)	12634	3532	101	77.7
Exercise frequency: Almost everyday	0.84 (0.73, 0.97)	24084	7509	228	90.9
Colon cancer					
Exercise frequency: None (Reference)	1.00 (1.00, 1.00)	190188	64984	779	38.5
Exercise frequency: 1-2 times/week	1.10 (0.98, 1.24)	147291	39851	500	32.7
Exercise frequency: 3-4 times/week	1.01 (0.86, 1.18)	56962	14273	194	33.2
Exercise frequency: 5-6 times/week	1.12 (0.85, 1.47)	12634	3532	56	42.9
Exercise frequency: Almost everyday	0.97 (0.80, 1.18)	24084	7509	122	48.3
Rectum cancer					
Exercise frequency: None (Reference)	1.00 (1.00, 1.00)	190188	64984	455	22.4
Exercise frequency: 1-2 times/week	1.04 (0.89, 1.21)	147291	39851	285	18.6
Exercise frequency: 3-4 times/week	0.91 (0.74, 1.13)	56962	14273	105	17.9
Exercise frequency: 5-6 times/week	0.92 (0.62, 1.36)	12634	3532	27	20.6
Exercise frequency: Almost everyday	1.11 (0.87, 1.41)	24084	7509	79	31.2
Lung cancer					
Exercise frequency: None (Reference)	1.00 (1.00, 1.00)	190188	64984	1500	74.4
Exercise frequency: 1-2 times/week	0.86 (0.78, 0.95)	147291	39851	602	39.4
Exercise frequency: 3-4 times/week	0.76 (0.66, 0.87)	56962	14273	219	37.5
Exercise frequency: 5-6 times/week	0.72 (0.55, 0.94)	12634	3532	57	43.6
Exercise frequency: Almost everyday	0.90 (0.78, 1.04)	24084	7509	207	82.2
Liver cancer					
Exercise frequency: None (Reference)	1.00 (1.00, 1.00)	190188	64984	987	48.8
Exercise frequency: 1-2 times/week	0.96 (0.86, 1.06)	147291	39851	559	36.6
Exercise frequency: 3-4 times/week	0.88 (0.75, 1.02)	56962	14273	210	36
Exercise frequency: 5-6 times/week	0.83 (0.62, 1.10)	12634	3532	50	38.2
Exercise frequency: Almost everyday	0.89 (0.74, 1.08)	24084	7509	127	50.3
Pancreas cancer					
Exercise frequency: None (Reference)	1.00 (1.00, 1.00)	190188	64984	249	12.2
Exercise frequency: 1-2 times/week	1.09 (0.88, 1.35)	147291	39851	140	9.1
Exercise frequency: 3-4 times/week	1.21 (0.91, 1.59)	56962	14273	65	11.1
Exercise frequency: 5-6 times/week	0.90 (0.52, 1.58)	12634	3532	13	9.9
Exercise frequency: Almost everyday	0.69 (0.46, 1.03)	24084	7509	27	10.6
Head&Neck cancer					
Exercise frequency: None (Reference)	1.00 (1.00, 1.00)	190188	64984	276	13.6
Exercise frequency: 1-2 times/week	0.74 (0.59, 0.92)	147291	39851	116	7.6
Exercise frequency: 3-4 times/week	0.87 (0.65, 1.16)	56962	14273	55	9.4
Exercise frequency: 5-6 times/week	0.68 (0.37, 1.25)	12634	3532	11	8.4
Exercise frequency: Almost everyday	0.94 (0.67, 1.33)	24084	7509	38	15
Kidney cancer					
Exercise frequency: None (Reference)	1.00 (1.00, 1.00)	190188	64984	194	9.6
Exercise frequency: 1-2 times/week	0.88 (0.70, 1.12)	147291	39851	113	7.4
Exercise frequency: 3-4 times/week	0.96 (0.70, 1.31)	56962	14273	49	8.4
Exercise frequency: 5-6 times/week	0.89 (0.49, 1.64)	12634	3532	11	8.4
Exercise frequency: Almost everyday	1.22 (0.84, 1.75)	24084	7509	34	13.4
Gallbladder cancer					
Exercise frequency: None (Reference)	1.00 (1.00, 1.00)	190188	64984	101	5
Exercise frequency: 1-2 times/week	0.81 (0.56, 1.16)	147291	39851	44	2.9
Exercise frequency: 3-4 times/week	0.89 (0.55, 1.44)	56962	14273	21	3.6
Exercise frequency: 5-6 times/week	1.43 (0.72, 2.85)	12634	3532	9	6.9
Exercise frequency: Almost everyday	1.48 (0.95, 2.31)	24084	7509	25	9.8
Esophagus cancer					
Exercise frequency: None (Reference)	1.00 (1.00, 1.00)	190188	64984	184	9
Exercise frequency: 1-2 times/week	0.86 (0.65, 1.15)	147291	39851	68	4.4
Exercise frequency: 3-4 times/week	0.76 (0.50, 1.16)	56962	14273	25	4.3
Exercise frequency: 5-6 times/week	0.64 (0.28, 1.45)	12634	3532	6	4.6
Exercise frequency: Almost everyday	0.97 (0.64, 1.45)	24084	7509	27	10.6

Women

Group	HR (95% CI)	Total cases(N)	Unique participants(n)	Events(n)	Crude rate
Stomach cancer					
Exercise frequency: None (Reference)	1.00 (1.00, 1.00)	213168	83300	910	40.2
Exercise frequency: 1-2 times/week	0.95 (0.83, 1.09)	73310	23072	267	35.6
Exercise frequency: 3-4 times/week	0.87 (0.72, 1.05)	35787	10563	126	35
Exercise frequency: 5-6 times/week	0.92 (0.67, 1.28)	9823	3144	38	38
Exercise frequency: Almost everyday	0.96 (0.78, 1.19)	20954	7626	100	45.7
Colon cancer					
Exercise frequency: None (Reference)	1.00 (1.00, 1.00)	213168	83300	645	28.4
Exercise frequency: 1-2 times/week	0.94 (0.80, 1.10)	73310	23072	192	25.5
Exercise frequency: 3-4 times/week	1.14 (0.93, 1.38)	35787	10563	120	33.3
Exercise frequency: 5-6 times/week	1.14 (0.81, 1.61)	9823	3144	34	34
Exercise frequency: Almost everyday	0.94 (0.73, 1.21)	20954	7626	69	31.5
Rectum cancer					
Exercise frequency: None (Reference)	1.00 (1.00, 1.00)	213168	83300	354	15.6
Exercise frequency: 1-2 times/week	0.83 (0.65, 1.04)	73310	23072	93	12.4
Exercise frequency: 3-4 times/week	0.84 (0.63, 1.14)	35787	10563	49	13.6
Exercise frequency: 5-6 times/week	1.16 (0.73, 1.84)	9823	3144	19	19
Exercise frequency: Almost everyday	0.69 (0.47, 1.01)	20954	7626	28	12.8
Lung cancer					
Exercise frequency: None (Reference)	1.00 (1.00, 1.00)	213168	83300	638	28.1
Exercise frequency: 1-2 times/week	1.04 (0.89, 1.23)	73310	23072	194	25.8
Exercise frequency: 3-4 times/week	0.93 (0.74, 1.17)	35787	10563	88	24.4
Exercise frequency: 5-6 times/week	1.04 (0.71, 1.52)	9823	3144	28	28
Exercise frequency: Almost everyday	0.96 (0.75, 1.24)	20954	7626	68	31
Liver cancer					
Exercise frequency: None (Reference)	1.00 (1.00, 1.00)	213168	83300	436	19.2
Exercise frequency: 1-2 times/week	0.93 (0.76, 1.13)	73310	23072	121	16.1
Exercise frequency: 3-4 times/week	0.79 (0.59, 1.05)	35787	10563	53	14.7
Exercise frequency: 5-6 times/week	1.32 (0.88, 1.97)	9823	3144	25	25
Exercise frequency: Almost everyday	1.05 (0.78, 1.40)	20954	7626	52	23.7
Pancreas cancer					
Exercise frequency: None (Reference)	1.00 (1.00, 1.00)	213168	83300	234	10.3
Exercise frequency: 1-2 times/week	0.93 (0.70, 1.23)	73310	23072	65	8.6
Exercise frequency: 3-4 times/week	0.75 (0.50, 1.12)	35787	10563	27	7.5
Exercise frequency: 5-6 times/week	1.07 (0.59, 1.97)	9823	3144	11	11
Exercise frequency: Almost everyday	1.26 (0.87, 1.81)	20954	7626	33	15
Head&Neck cancer					
Exercise frequency: None (Reference)	1.00 (1.00, 1.00)	213168	83300	101	4.4
Exercise frequency: 1-2 times/week	0.87 (0.57, 1.33)	73310	23072	28	3.7
Exercise frequency: 3-4 times/week	1.11 (0.67, 1.84)	35787	10563	18	5
Exercise frequency: 5-6 times/week	0.88 (0.32, 2.39)	9823	3144	4	4
Exercise frequency: Almost everyday	0.82 (0.41, 1.62)	20954	7626	9	4.1
Kidney cancer					
Exercise frequency: None (Reference)	1.00 (1.00, 1.00)	213168	83300	107	4.7
Exercise frequency: 1-2 times/week	1.14 (0.79, 1.64)	73310	23072	40	5.3
Exercise frequency: 3-4 times/week	1.45 (0.94, 2.24)	35787	10563	26	7.2
Exercise frequency: 5-6 times/week	0.99 (0.40, 2.44)	9823	3144	5	5
Exercise frequency: Almost everyday	0.83 (0.43, 1.59)	20954	7626	10	4.6
Gallbladder cancer					
Exercise frequency: None (Reference)	1.00 (1.00, 1.00)	213168	83300	118	5.2
Exercise frequency: 1-2 times/week	1.20 (0.83, 1.74)	73310	23072	39	5.2
Exercise frequency: 3-4 times/week	1.33 (0.84, 2.11)	35787	10563	22	6.1
Exercise frequency: 5-6 times/week	1.48 (0.69, 3.18)	9823	3144	7	7
Exercise frequency: Almost everyday	1.06 (0.61, 1.85)	20954	7626	14	6.4
Esophagus cancer					
Exercise frequency: None (Reference)	1.00 (1.00, 1.00)	213168	83300	30	1.3
Exercise frequency: 1-2 times/week	0.83 (0.36, 1.91)	73310	23072	7	.9
Exercise frequency: 3-4 times/week	0.93 (0.32, 2.67)	35787	10563	4	1.1
Exercise frequency: 5-6 times/week	0.00 (0.00, 0.00)	9823	3144	0	0
Exercise frequency: Almost everyday	0.31 (0.04, 2.27)	20954	7626	1	.5

Supplementary Figure 5. Results from running Cox regression models examining effect modification of sex, body mass index, smoking, alcohol consumption, and family history of disease in the associations between exercise frequency and various incident cancer outcomes. Note: Cox regression models using age as the underlying timescale were adjusted for sex [not in models for effect

modification by sex], body mass index [not in models for effect modification by body mass index], systolic blood pressure, fasting glucose levels, total cholesterol levels, family history of cancer [not in models for effect modification by family history of cancer], smoking status [not in models for effect modification by smoking status] and alcohol consumption [not in models for effect modification by alcohol consumption]. Crude rates are per 100,000 person-years. “N” indicates numbers of total observations (i.e. participants who provided repeated measures are treated as separate observations) and “n” indicates numbers of unique participants at baseline. P-values for multiplicative terms – stomach cancer (p-value = 0.274), colon cancer (p-value = 0.640), rectum cancer (p-value = 0.107), lung cancer (p-value = 0.016), liver cancer (p-value = 0.129), pancreas cancer (p-value = 0.860), head & neck cancer (p-value = 0.488), kidney cancer (p-value = 0.285), gallbladder cancer (p-value = 0.970), and esophagus cancer (p-value = 0.263) by body mass index; stomach cancer (p-value = 0.699), colon cancer (p-value = 0.932), rectum cancer (p-value = 0.610), lung cancer (p-value = 0.492), liver cancer (p-value = 0.405), pancreas cancer (p-value = 0.338), head & neck cancer (p-value = 0.562), kidney cancer (p-value = 0.280), gallbladder cancer (p-value = 0.295), and esophagus cancer (p-value = 0.292) by smoking ; stomach cancer (p-value = 0.655), colon cancer (p-value = 0.977), rectum cancer (p-value = 0.433), lung cancer (p-value = 0.387), liver cancer (p-value = 0.704), pancreas cancer (p-value = 0.711), head & neck cancer (p-value = 1.000), kidney cancer (p-value = 0.336), gallbladder cancer (p-value = 0.350), and esophagus cancer (p-value = 0.550) by alcohol consumption ; stomach cancer (p-value = 0.996), colon cancer (p-value = 0.399), rectum cancer (p-value = 0.478), lung cancer (p-value = 0.427), liver cancer (p-value = 0.337), pancreas cancer (p-value = 0.086), head & neck cancer (p-value = 0.712), kidney cancer (p-value = 0.319), gallbladder cancer (p-value = 0.766), and esophagus cancer (p-value = 0.098) by family history of cancer; and stomach cancer (p-value = 0.405), colon cancer (p-value = 0.957), rectum cancer (p-value = 0.106), lung cancer (p-value = 0.063), liver cancer (p-value = 0.278), pancreas cancer (p-value = 0.265), head & neck cancer (p-value = 0.907), kidney cancer (p-value = 0.967), gallbladder cancer (p-value = 0.548), and esophagus cancer (p-value = 0.256) by sex. Abbreviations: HR – Hazard Ratio; CI – Confidence Intervals
