# Supplementary material for: Exercise and incidence of myocardial infarction, stroke, hypertension, type 2 diabetes and site-specific cancers: prospective cohort study of 257 854 adults in South Korea
Source: BMJ Open. 2019 Mar 13;9(3):e025590. doi: 10.1136/bmjopen-2018-025590 (PMC6430026; doi:10.1136/bmjopen-2018-025590)
Supplement: Author's manuscript [file bmjopen-2018-025590.draft_revisions.pdf]

BMJ Open

BMJ Open is committed to open peer review. As part of this commitment we make the peer review history of every article we publish publicly available.

When an article is published we post the peer reviewers' comments and the authors' responses online. We also post the versions of the paper that were used during peer review. These are the versions that the peer review comments apply to.

The versions of the paper that follow are the versions that were submitted during the peer review process. They are not the versions of record or the final published versions. They should not be cited or distributed as the published version of this manuscript.

BMJ Open is an open access journal and the full, final, typeset and author-corrected version of record of the manuscript is available on our site with no access controls, subscription charges or pay-per-view fees (http://bmjopen.bmj.com).

If you have any questions on BMJ Open's open peer review process please email info.bmjopen@bmj.com

BMJ Open

Exercise and incidence of myocardial infarction, stroke, hypertension, type 2 diabetes and site-specific cancers

Journal:	*BMJ Open*
Manuscript ID	bmjopen-2018-025590
Article Type:	Research
Date Submitted by the Author:	23-Jul-2018
Complete List of Authors:	Kim, Youngwon; University of Utah, Department of Health, Kinesiology, and Recreation; University of Cambridge School of Clinical Medicine, MRC Epidemiology Unit<br>Sharp, Stephen; University of Cambridge, MRC Epidemiology Unit<br>Hwang, Se-mi; Yonsei University, Department of Epidemiology and Health Promotion, Graduate School of Public Health<br>Jee, Sun Ha; Graduate School of Public Health, Yonsei University, Epidemiology
Keywords:	exercise, non-communicable disease, cohort, Epidemiology < TROPICAL MEDICINE, cardiovascular disease, Hypertension < CARDIOLOGY

SCHOLARONE™
Manuscripts

Title: Exercise and incidence of myocardial infarction, stroke, hypertension, type 2 diabetes and site-specific cancers

Authors: Youngwon Kim,[1,2] Stephen J. Sharp,[2] Se-mi Hwang,[3] Sun Ha Jee[3]

Affiliations:

[1] Department of Health, Kinesiology, and Recreation, College of Health, University of Utah, UT, USA

[2] MRC Epidemiology Unit, University of Cambridge School of Clinical Medicine, Cambridge, UK

[3] Institute for Health Promotion, Department of Epidemiology and Health Promotion, Graduate School of Public Health, Yonsei University, Seoul, Republic of Korea.

Full Addresses:

[1] Department of Health, Kinesiology, and Recreation, College of Health, University of Utah. 250 South 1850 East Room 204, Salt Lake City, UT, USA, 84112

[2] MRC Epidemiology Unit, University of Cambridge School of Clinical Medicine, Box 285 Institute of Metabolic Science, Cambridge Biomedical Campus, Cambridge, Cambridgeshire, UK CB2 0QQ

[3] Department of Epidemiology and Health Promotion, Graduate School of Public Health, Yonsei University, 50-1 Yonsei-ro, Seodaemun-gu, Seoul, Korea 03722

Corresponding Author:

Name: Sun Ha Jee, Ph.D

Mailing Address: Department of Epidemiology and Health Promotion, Graduate School of Public Health, Yonsei University, 50-1 Yonsei-ro, Seodaemun-gu, Seoul, Korea 03722

E-mail Information: jsunha@yuhs.ac

Telephone: +82-2-2228-1523

Fax: +82-2-365-5118

Word count for Abstract: 250

Word count for Main text: 2787

For peer review only

Abstract

Objective: Little is known about the dose-response relationships between exercise and non-communicable diseases in East Asians. The objective of this study was to examine longitudinal associations of exercise frequency with the incidence of myocardial infarction, stroke, hypertension, type 2 diabetes and 10 different cancer outcomes.

Design: A prospective cohort study.

Setting: Physical examination data linked with the entire Korean population's health insurance system.

Participants: 257,854 Korean adults who provided up to 7 repeat-measures of exercise and confounders.

Primary outcome measures: Each disease incidence was defined using both fatal and non-fatal health records (a median follow-up period of 13 years).

Results: Compared with no exercise category, the middle categories of exercise frequency (1-2, 3-4 or 5-6 times/week) showed the lowest risk of myocardial infarction (hazard ratio[HR]: 0.79; 95% confidence interval[CI]: 0.70-0.90), stroke (HR: 0.80; 95%CI: 0.73-0.89), hypertension (HR: 0.86; 95%CI: 0.85-0.88), type 2 diabetes (HR: 0.87; 95%CI: 0.84-0.89), stomach (HR: 0.87; 95%CI: 0.79-0.96), lung (HR: 0.80; 95%CI: 0.71-0.91), liver (HR: 0.85; 95%CI: 0.75-0.98) and head & neck cancer (HR: 0.76; 95%CI: 0.63-0.93), exhibiting J-shaped associations. There was, in general, little evidence of effect modification by body mass index, smoking, alcohol consumption, family history of disease, and sex in these associations.

Conclusions: Moderate levels of exercise showed the lowest risk of myocardial infarction, stroke, hypertension, type 2 diabetes, stomach, lung, liver and head & neck cancer. Public health and lifestyle interventions should promote moderate levels of exercise as a behavioral prevention strategy for non-communicable diseases in a wider population of East Asians.

For peer review only

Keywords: exercise, non-communicable disease, cohort, epidemiology, cardiovascular disease, hypertension, cancer

For peer review only

Article Summary

Strengths and limitations of this study

- This study is the first to investigate the longitudinal associations of exercise with various cardiovascular and cancer incident outcomes in an East Asian population.
- Another strength is the use of a large-scale cohort dataset of adults (n=257,854) who provided up to 7 repeated measures of exercise frequency and all confounders in order to minimize the risk of regression dilution.
- A limitation is that no strong inference can be drawn about the exercise-incident disease relationships.

For peer review only

Introduction

Prevention and control of non-communicable diseases is a contemporary global public health priority. At present, 40 million deaths per year, which accounts for nearly 70% of total deaths globally, are attributable to non-communicable diseases.[1,2] Moreover, the number of deaths due to non-communicable diseases, such as cardiovascular disease,[3] hypertension,[4] diabetes[5] and cancer,[6] has increased dramatically over the past few decades, although age-standardized cardiovascular disease and cancer rates as well as systolic blood pressure levels[7] have declined.[8,9] However, trends in these disease traits have varied across different populations, particularly with less favorable changes observed in East Asian populations compared with Western populations. For example, diabetes rates[10] have increased more rapidly, while age-standardized cardiovascular disease rates[3] and systolic blood pressure levels[7] have fallen less steeply in East Asians in comparison with Westerners.

In addition, adults in East Asia tend to have higher prevalence of physical inactivity,[11] which is one of the four target behaviors (including unhealthy diet, tobacco use and harmful use of alcohol) that have been set as the global focus to reduce the risk of non-communicable diseases.[12] The beneficial impacts of increased physical activity on various non-communicable outcomes have been demonstrated by numerous previous investigations. However, the majority of previous research has been predicated on evidence from Western populations, thereby limiting its application to other populations including East Asians. As such, little is currently known about levels of physical activity including exercise in relation to non-communicable diseases in East Asian populations as compared with Western populations.[13] Another critical gap in the existing literature is the use of data measured only at a single point in time (i.e. baseline), in which case physical activity or exercise levels are assumed to remain constant over time. This methodology, therefore, does not allow for the fact that individuals' physical activity or exercise levels change with time, and hence may increase the potential for regression dilution.[14] Furthermore, it is well-known that temporal changes occur in other traditional behavioral and metabolic risk factors for non-

For peer review only

communicable diseases, such as adiposity levels,[15,16] smoking,[17] glucose levels[18] and total cholesterol levels,[19] showing different patterns of changes between East Asian and Western populations. Nevertheless, no previous research of East Asians or Westerners took into account changes in these risk markers in understanding the relationships between physical activity and non-communicable diseases. Moreover, the dose-response relationship between physical activity and various non-communicable disease outcomes has remained unclear in East Asians. Therefore, the purpose of this research was to explore the dose-response relationships between exercise frequency and various types of incident non-communicable diseases, such as myocardial infarction, stroke, hypertension, type 2 diabetes and site-specific cancers, using a large-scale prospective cohort of Korean adults with multiple repeated measures of exercise frequency and other risk markers.

Methods

Study design and participants

This study is based on data from the National Health Insurance Service - Health Screening (NHIS-HEALS) cohort dataset,[20] which is a nationally representative random sample (stratified by sex, age, employment status and income) of >500,000 South Korean adults aged 40-79 years between 2002 and 2003 made available by the NHIS. The NHIS is a single health insurance system in South Korea, which manages and maintains information on the entire South Korean population's healthcare utilization; it is mandatory for all South Koreans to take part in the national health insurance system. The NHIS is also responsible for maintaining national health examination programs involving data from general health examinations of all insured employees or self-employed individuals aged >40 years; it is recommended for them to perform the health examination at least every two years. The health examination involves collection of information on body composition, blood profiles, blood pressure, self-reported lifestyles, self-reported physician-diagnosed disease, and self-reported family history of disease.

For peer review only

The NHIS-HEALS cohort includes a wide variety of information collected between 2002 and 2015: health examination data and demographic and eligibility data (e.g., in-patient and out-patient hospital records, medical bill, health insurance and medical aid beneficiaries, etc.). In the present analysis, we utilized health examination data collected between 2002 and 2008 to define the exercise frequency and all confounders. There was a change in the type of self-report methods in 2009; hence, health examination data collected in or after 2009 were not considered in the analysis due to the inability to harmonize variables. However, we used full follow-up data accrued from 2002 until 2015. This research was approved by the Institutional Review Board (4-2017-0051) of the Yonsei University's Severance Hospital in Republic of Korea.

Exposure

The primary exposure variable of this study was exercise frequency, assessed using questionnaires administered during the health examinations. The specific question asked was "How many times per week do you engage in exercise that causes sweating?" Participants were asked to choose only one of the following 5 possible answers: None, 1-2 times/week, 3-4 times/week, 5-6 times/week and almost every day.

Outcomes

We evaluated 17 different incident disease outcomes in the present study: myocardial infarction, stroke, hypertension, type 2 diabetes, stomach, colon, rectum, lung, liver, head & neck, pancreas, kidney, gallbladder and esophagus cancer. Participants' in-patient and out-patient hospital records (i.e. non-fatal status) and death records (i.e. fatal status) obtained through linkage with Statistics Korea were both classified according to the International Classification of Disease (ICD)-10 codes to classify different incidence types (Supplementary Table 1). Additionally, blood pressure (e.g., systolic ≥140 mmHg, diastolic ≥90 mmHg) and fasting glucose levels (e.g., ≥126 mg/dL), both of which were measured during physical examinations, were used in conjunction with physician diagnosis information and ICD-10

For peer review only

codes to define incident hypertension and type 2 diabetes, respectively. Each incident disease outcome was defined as the first occurrence of either non-fatal or fatal respective disease cases. Follow-up was censored at occurrence of an incident disease outcome or the end date of hospital and death records (December 31st, 2015). The median follow-up was 13.0 years (interquartile range: 10.2-11.3 years)

Confounders

The following variables were included as confounders in the analyses: sex, body mass index, systolic blood pressure, fasting glucose, total cholesterol, family history of heart disease, stroke or hypertension [only in models for incident myocardial infarction, stroke or hypertension], family history of diabetes [only in models for type 2 diabetes], family history of cancer [only in models for incident cancer outcomes], smoking status (never, previously, currently) and alcohol consumption (never, 2-3 times/month, 1-2 times/week, ≥3 times/week).

Statistical analysis

Cox regression with age as the underlying time scale was used to estimate the associations of exercise frequency with each incident disease outcome, with adjustment for all the above-mentioned confounders as well as without any adjustment. Data were structured to enable the inclusion of exercise frequency and all confounders from both baseline and up to 6 repeated measures as time-updated covariates. This approach takes into account changes in exercise frequency as well as each confounder over time in relation to disease incidence. Individuals who reported no exercise served as a reference group for all comparisons. Effect modification by body mass index (<25, ≥25kg^2/m), smoking status, alcohol consumption, family history of disease and sex was also examined for each incident disease outcome. Visual inspections of log-log plots provided support for the assumptions of proportional hazards for all covariates. A sensitivity analysis where incident disease cases occurring during the first 2 years of follow-up were removed was performed to address reverse

For peer review only

causality. Analyses were performed in Stata/SE Version 14 (StataCorp LP, College Station, TX).

Patient and Public Involvement.

Neither patients nor members of the public were involved in this study.

Results

Of an initial sample of 512,190 individuals, 74,931 had missing data on at least one of the model covariates, and 179,405 had self-reported physician-diagnosed heart attack, stroke, hypertension (additionally, systolic ≥140 mmHg or diastolic ≥90 mmHg), diabetes (additionally, fasting glucose levels ≥126 mg/dL) or cancer at baseline, respectively. Excluding these individuals resulted in a final sample for analysis of 257,854 individuals (Figure 1).

Individuals provided up to 7 measures of exercise frequency and each confounder (i.e. baseline plus 6 repeated measures). Participants' characteristics at baseline are summarized in Table 1. Supplementary Table 2 summarizes participants' characteristics at each repeat assessment. Individuals in the categories of 1-2, 3-4 or 5-6 times/week of exercise were slightly younger, but showed higher proportions of family history of disease and lower proportions of never smoking or drinking alcohol, compared with those in the categories of none or almost every day of exercise. Across the seven time points (Supplementary Figure 2), the proportion of individuals who reported no exercise decreased while the proportion who reported 1-2 or 3-4 times/week of exercise increased; there were no noticeable changes for the categories of 5-6 times/week or almost every day of exercise.

Overall, J-shaped associations were found between exercise frequency and incident myocardial infarction, stroke, hypertension and type 2 diabetes. Hazard ratios for these diseases were lowest in the middle categories of exercise frequency (e.g., 3-4 or 5-6 times/week) (Figure 2). There were no associations for the most frequent exercise category

For peer review only

(e.g., almost every day) with the incidence of myocardial infarction, stroke and type 2 diabetes.

J-shaped associations were also found for incident stomach, lung, liver and head & neck cancer (Figure 3). Higher exercise frequencies (e.g., 1-2, 3-4 times/week and almost every day) were associated with lower hazards of incident stomach cancer. No statistical significance was observed for incident colon, rectum, pancreas, kidney, gallbladder and esophagus cancer. Crude event rates per 100,000 person-years in the middle categories of exercise frequency were relatively lower for incident rectum, and esophagus cancer, but higher for incident pancreas, kidney and gallbladder cancer. Cox regression models with no adjustment for confounders (Supplementary Figure 2) and a sensitivity analysis (Supplementary Figure 3) in which incident cases occurring in the first 2 years of follow-up were removed both revealed nearly identical patterns of associations as the main analyses.

Figure 4 shows comparisons of results that showed statistical significance for multiplicative interaction terms between exercise frequency and each incident disease outcome. Strong J-shaped associations for incident hypertension were identified at each level of body mass index. J-shaped associations of exercise frequency with incident hypertension were strong only in the more favorable levels of smoking (e.g., never, previously) and alcohol consumption (e.g., never, 2-3times/month, 1-2times/week); no or weak associations were identified in the most harmful level of smoking (e.g., current smokers) and alcohol consumption (e.g., ≥3times/week). J-shaped associations were evident at all levels of family history of CVD and sex for incident hypertension, and sex for incident type 2 diabetes. Exercise frequency was associated with incident lung cancer in non-obese individuals, but there was no evidence of association in obese individuals. All comparisons stratified by each potential effect modifier are presented in Supplementary Figures 4 and 5.

Discussion

For peer review only

This is the first investigation examining the prospective associations of exercise with various incident non-communicable disease outcomes using multiple repeated measures of covariates in East Asian populations. We identified J-shaped associations of exercise frequency with incident myocardial infarction, stroke, hypertension, type 2 diabetes, stomach, lung, liver and head & neck cancer, with the greatest benefits being observed in the middle categories of exercise frequency (e.g., 1-2, 3-4 or 5-6 times/week). These findings provide two important clinical and public health implications. First, prevention and management of non-communicable diseases in East Asians may benefit considerably from employing an exercise promotion approach in the context of combined non-communicable disease prevention. Mechanism research indicates that cardiovascular disease and type 2 diabetes have similar biological pathways relating to exercise,[21,22] so an integrated prevention approach can be applied to control and manage these two diseases at a minimum.[5] Moreover, regular participation in exercise can induce favorable changes in intermediate cardiometabolic risk markers,[23] which are important predictors of typical non-communicable diseases. Hence, promoting exercise has great potential to act as an integrative behavioral strategy for preventing and controlling various non-communicable diseases simultaneously in East Asian populations.

Second, individuals who engage in exercise 1-2, 3-4 or 5-6 times/week, rather than every day, may be able to reduce their risk of developing myocardial infarction, stroke, hypertension, type 2 diabetes, stomach, lung, liver and head & neck cancer. Similar J-shaped associations between high intensity exercise (e.g., running) and cardiovascular disease risk have also been reported in previous cohort studies of Western[24,25] and Japanese adults.[26] Nevertheless, the present study as well as previous research[24-26] found that the risk of developing cardiovascular events in individuals who had the highest level of exercise was not noticeably higher compared with those who had the lowest level of exercise. No previous research in East Asians has found such J-shaped relationships between exercise or physical activity and other incident disease outcomes such as hypertension,[27-31] diabetes[32-39] and different type of cancers.[40-46] However, previous meta-

For peer review only

analyses of cohort studies comprising predominantly Westerners found leisure-time physical activity to have curvilinear (but not J-shaped) associations with the incidence of type 2 diabetes,[47] and linear associations with the incidence of hypertension[48] and various site-specific cancers (liver, lung, head & neck, kidney, colon, rectal, bladder, gastric cardia, breast, endometrial, myeloid leukemia, myeloma, esophageal adenocarcinoma).[49] While additional research is needed to confirm the J-shaped associations of exercise with various incident diseases in other samples of East Asians, findings of this research provide a strong rationale for development and implementation of public health policies and clinical trials aimed at promoting a moderate level of exercise to minimize the risk of myocardial infarction, stroke, hypertension, type 2 diabetes, stomach, lung, liver and head & neck cancer.

Another finding of this research is that associations of exercise frequency with hypertension were modified by body mass index, smoking, alcohol consumption, family history of cardiovascular disease and sex: lung cancer by body mass index and type 2 diabetes by sex. Notably, exercise frequency was not associated with hypertension in individuals who are smokers or drinking alcohol ≥3 times/week (except for 3-4 times/week of exercise). This observation provides some evidence that the harmful impacts of smoking or binge drinking on hypertension[50-52] may not be offset completely by exercise. This, in turn, appears to advocate for the need for implementing a combined hypertension prevention strategy targeting promotion of exercise in conjunction with smoking cessation and reductions in alcohol consumption in East Asians.[13] For lung cancer, the null associations in individuals with body mass index ≥25 may be indicative of potential residual confounding through reported bias in smoking behaviors. Nonetheless, there was little evidence for effect modification for other disease comparisons, highlighting the importance of promoting exercise for the prevention of various non-communicable diseases in individuals at different categories of body mass index, smoking, alcohol consumption, family history of disease and sex.

For peer review only

This study has several notable strengths. First, we used data from a large prospective cohort study in which exercise and other risk markers were assessed on multiple occasions (up to 7 times). Nearly 84% and 5% of the full participants provided 1 and 6 repeated measures of all covariates, respectively. Compelling evidence indicates that the risk of regression dilution can be reduced using repeated measures of exposure and confounders.[14] Moreover, we examined the dose-response-relationship of exercise frequency with a wide variety of specific types of incident non-communicable disease outcomes simultaneously using in-patient and out-patient diagnosis data as well as mortality data. The large sample size (n=257,854) is another strength.

This study has some limitations. Findings of this study may not be generalizable to adult populations of other countries. Due to the observational nature of this research, no strong inference can be drawn about the exercise-incident disease relationships. In addition, the accuracy of hospital admission records is uncertain, although the accuracy of death records from Statistics Korea was found to be 92% in previous research.[53] No information about medication use was available in the cohort data, so we could not use it as a potential confounder and another condition when defining disease status (e.g., hypertension, type 2 diabetes) at both baseline and follow-up. Furthermore, no exercise duration was assessed; hence, inference was made purely based on exercise frequency. Moreover, ICD-10 codes for sex-specific cancers (e.g., prostate and breast cancer) were masked due to the data management policy set forth by the NHIS, so it was not possible to examine such cancers in the present study. The lack of data on diet, which is another behavioral risk marker for non-communicable diseases[12] is another limitation.

Conclusion

Exercise frequency showed J-shaped associations with the risk of developing myocardial infarction, stroke, hypertension, type 2 diabetes, stomach, lung, liver and head & neck cancer, with the lowest risks found in individuals who engaged in exercise 1-2, 3-4 or 5-6 times/week as opposed to every day. These findings were generally applicable to different

For peer review only

sub-populations as stratified by body mass index, smoking, alcohol consumption, family history of disease, and sex. Public health and lifestyle interventions should promote a moderate level of exercise as a behavioral strategy for prevention and control of non-communicable diseases in a wider population of East Asians.

Author Contributions

YK designed this study, performed statistical analysis, and drafted an initial version of the manuscript. SJS, SMH and SHJ all contributed to conceptualizing the study idea and developing the analytical plans, and provided assistance with statistical analysis. All authors critically reviewed, approved of the final version of the manuscript, and agreed to be responsible for all facets of this work.

Acknowledgements

None.

Funding

This work was supported by the National R&D Program for Cancer Control, Ministry of Health & Welfare, Republic of Korea (Grant 1631020 to S.H.J.), The Korean Health Technology R&D Project, Ministry of Health & Welfare, Republic of Korea (Grant HI14C2686 to S.H.J.), and the Medical Research Council (Grant MC_UU_12015/1 to S.J.S.). The funders had no role in study design, data collection and analysis, decision to publish, or preparation of the manuscript.

Conflicts of interest

None declared.

Patient consent

Not required.

Ethics approval

For peer review only

This research was approved by the Institutional Review Board (4-2017-0051) of the Yonsei University's Severance Hospital in Republic of Korea.

Data sharing statement

Data sharing is not applicable because no informed consent for data sharing was obtained from the participants.

For peer review only

References

1 Mortality, G. B. D. & Causes of Death, C. Global, regional, and national life expectancy, all-cause mortality, and cause-specific mortality for 249 causes of death, 1980-2015: a systematic analysis for the Global Burden of Disease Study 2015. *Lancet* **388**, 1459-1544, doi:10.1016/S0140-6736(16)31012-1 (2016).

2 Lakerveld, J., Bot, S. D., van der Ploeg, H. P. & Nijpels, G. The effects of a lifestyle intervention on leisure-time sedentary behaviors in adults at risk: The Hoorn Prevention Study, a randomized controlled trial. *Prev Med* **57**, 351-356, doi:10.1016/j.ypmed.2013.06.011 (2013).

3 Roth, G. A. *et al.* Global, Regional, and National Burden of Cardiovascular Diseases for 10 Causes, 1990 to 2015. *J Am Coll Cardiol* **70**, 1-25, doi:10.1016/j.jacc.2017.04.052 (2017).

4 Forouzanfar, M. H. *et al.* Global Burden of Hypertension and Systolic Blood Pressure of at Least 110 to 115 mm Hg, 1990-2015. *Jama* **317**, 165-182, doi:10.1001/jama.2016.19043 (2017).

5 *World Health Organization Global report on diabetes*. (World Health Organization, 2016).

6 Global Burden of Disease Cancer, C. *et al.* Global, Regional, and National Cancer Incidence, Mortality, Years of Life Lost, Years Lived With Disability, and Disability-Adjusted Life-years for 32 Cancer Groups, 1990 to 2015: A Systematic Analysis for the Global Burden of Disease Study. *JAMA Oncol* **3**, 524-548, doi:10.1001/jamaoncol.2016.5688 (2017).

7 Danaei, G. *et al.* National, regional, and global trends in systolic blood pressure since 1980: systematic analysis of health examination surveys and epidemiological studies with 786 country-years and 5.4 million participants. *Lancet* **377**, 568-577, doi:10.1016/S0140-6736(10)62036-3 (2011).

8 Roth, G. A. *et al.* Global and regional patterns in cardiovascular mortality from 1990 to 2013. *Circulation* **132**, 1667-1678, doi:10.1161/CIRCULATIONAHA.114.008720 (2015).

9 Death, G. C. Global, regional, and national age-sex specific mortality for 264 causes of death, 1980-2016: a systematic analysis for the Global Burden of Disease Study 2016 (vol 390, pg 1151, 2017). *Lancet* **390**, E38-E38 (2017).

10 NCD-, N. R. F. C. NCD Risk Factor Collaboration (NCD-RisC). Worldwide trends in diabetes since 1980: a pooled analysis of 751 population-based studies with 4.4 million participants (vol 387, pg 1513, 2016). *Lancet* **389**, E2-E2, doi:10.1016/S0140-6736(16)32060-8 (2017).

11 Ku, P. W., Fox, K. R., McKenna, J. & Peng, T. L. Prevalence of leisure-time physical activity in Taiwanese adults: results of four national surveys, 2000-2004. *Preventive medicine* **43**, 454-457, doi:10.1016/j.ypmed.2006.04.011 (2006).

12 World Health Organization. Global status report on noncommunicable diseases 2010. Geneva, World Health Organization, 2011.

13 Kokubo, Y. Prevention of hypertension and cardiovascular diseases: a comparison of lifestyle factors in Westerners and East Asians. *Hypertension* **63**, 655-660, doi:10.1161/HYPERTENSIONAHA.113.00543 (2014).

14 Hutcheon, J. A., Chiolero, A. & Hanley, J. A. Random measurement error and regression dilution bias. *BMJ* **340**, c2289, doi:10.1136/bmj.c2289 (2010).

15 Ng, M. *et al.* Global, regional, and national prevalence of overweight and obesity in children and adults during 1980-2013: a systematic analysis for the Global Burden of Disease Study 2013. *Lancet* **384**, 766-781, doi:10.1016/S0140-6736(14)60460-8 (2014).

16 Lear, S. A. *et al.* Visceral adipose tissue accumulation differs according to ethnic background: results of the Multicultural Community Health Assessment Trial (M-CHAT). *Am J Clin Nutr* **86**, 353-359 (2007).

17 (ed Geneva: World Health Organization) (2015).

18 Chan, J. C. *et al.* Diabetes in Asia: epidemiology, risk factors, and pathophysiology. *Jama* **301**, 2129-2140, doi:10.1001/jama.2009.726 (2009).

For peer review only

19 Nguyen, H. N., Fujiyoshi, A., Abbott, R. D. & Miura, K. Epidemiology of cardiovascular risk factors in Asian countries. *Circulation journal : official journal of the Japanese Circulation Society* **77**, 2851-2859 (2013).

20 Seong, S. C. *et al.* Cohort profile: the National Health Insurance Service-National Health Screening Cohort (NHIS-HEALS) in Korea. *BMJ open* **7**, e016640, doi:10.1136/bmjopen-2017-016640 (2017).

21 Pedersen, B. K. Anti-inflammatory effects of exercise: role in diabetes and cardiovascular disease. *Eur J Clin Invest* **47**, 600-611, doi:10.1111/eci.12781 (2017).

22 Stern, M. P. Diabetes and cardiovascular disease. The "common soil" hypothesis. *Diabetes* **44**, 369-374 (1995).

23 *Global Recommendations on Physical Activity for Health*. (2010).

24 Lee, D. C. *et al.* Leisure-Time Running Reduces All-Cause and Cardiovascular Mortality Risk. *J. Am. Coll. Cardiol.* **64**, 472-481, doi:10.1016/j.jacc.2014.04.058 (2014).

25 Tanasescu, M. *et al.* Exercise type and intensity in relation to coronary heart disease in men. *Jama* **288**, 1994-2000 (2002).

26 Kubota, Y. *et al.* Daily Total Physical Activity and Incident Stroke: The Japan Public Health Center-Based Prospective Study. *Stroke* **48**, 1730-1736, doi:10.1161/STROKEAHA.117.017560 (2017).

27 Lu, Y. *et al.* Lifestyle and Risk of Hypertension: Follow-Up of a Young Pre-Hypertensive Cohort. *Int J Med Sci* **12**, 605-612, doi:10.7150/ijms.12446 (2015).

28 Sun, Z. *et al.* Incidence and predictors of hypertension among rural Chinese adults: results from Liaoning province. *Ann Fam Med* **8**, 19-24, doi:10.1370/afm.1018 (2010).

29 Gu, D. *et al.* Incidence and predictors of hypertension over 8 years among Chinese men and women. *J Hypertens* **25**, 517-523, doi:10.1097/HJH.0b013e328013e7f4 (2007).

30 Lee, S. *et al.* Cardiorespiratory fitness attenuates metabolic risk independent of abdominal subcutaneous and visceral fat in men. *Diabetes Care* **28**, 895-901, doi:DOI 10.2337/diacare.28.4.895 (2005).

31 Nakanishi, N. & Suzuki, K. Daily life activity and the risk of developing hypertension in middle-aged Japanese men. *Archives of internal medicine* **165**, 214-220, doi:10.1001/archinte.165.2.214 (2005).

32 Fan, S. *et al.* Physical Activity Level and Incident Type 2 Diabetes among Chinese Adults. *Med Sci Sport Exer* **47**, 751-756, doi:10.1249/Mss.0000000000000471 (2015).

33 Joseph, J. J. *et al.* Physical activity, sedentary behaviors and the incidence of type 2 diabetes mellitus: the Multi-Ethnic Study of Atherosclerosis (MESA). *Bmj Open Diab Res Ca* **4**, doi:ARTN e00018510.1136/bmjdrc-2015-000185 (2016).

34 Xu, F. *et al.* Joint Associations of Physical Activity and Hypertension with the Development of Type 2 Diabetes among Urban Men and Women in Mainland China. *Plos One* **9**, doi:ARTN e8871910.1371/journal.pone.0088719 (2014).

35 Xu, F. *et al.* Joint impact of physical activity and family history on the development of diabetes among urban adults in Mainland China: a pooled analysis of community-based prospective cohort studies. *Asia Pac J Public Health* **27**, NP372-381, doi:10.1177/1010539512443700 (2015).

36 Shi, L. *et al.* Physical activity, smoking, and alcohol consumption in association with incidence of type 2 diabetes among middle-aged and elderly Chinese men. *Plos One* **8**, e77919, doi:10.1371/journal.pone.0077919 (2013).

37 Villegas, R. *et al.* Physical activity and the incidence of type 2 diabetes in the Shanghai women's health study. *Int J Epidemiol* **35**, 1553-1562, doi:10.1093/ije/dyl209 (2006).

38 Lee, D. C. *et al.* Physical Activity and Body Mass Index and Their Associations With the Development of Type 2 Diabetes in Korean Men. *Am J Epidemiol* **176**, 43-51, doi:10.1093/aje/kwr471 (2012).

39 Honda, T. *et al.* Leisure-time, occupational, and commuting physical activity and risk of type 2 diabetes in Japanese workers: a cohort study. *Bmc Public Health* **15**, doi:ARTN 1004

10.1186/s12889-015-2362-5 (2015).

40 Inoue, M. *et al.* Daily total physical activity level and total cancer risk in men and women: results from a large-scale population-based cohort study in Japan. *Am J Epidemiol* **168**, 391-403, doi:10.1093/aje/kwn146 (2008).

41 Lee, K. J. *et al.* Physical activity and risk of colorectal cancer in Japanese men and women: the Japan Public Health Center-based prospective study. *Cancer causes & control : CCC* **18**, 199-209, doi:10.1007/s10552-006-0098-3 (2007).

42 Luo, J. *et al.* Body mass index, physical activity and the risk of pancreatic cancer in relation to smoking status and history of diabetes: a large-scale population-based cohort study in Japan--the JPHC study. *Cancer causes & control : CCC* **18**, 603-612, doi:10.1007/s10552-007-9002-z (2007).

43 Suzuki, S. *et al.* Effect of physical activity on breast cancer risk: findings of the Japan collaborative cohort study. *Cancer epidemiology, biomarkers & prevention : a publication of the American Association for Cancer Research, cosponsored by the American Society of Preventive Oncology* **17**, 3396-3401, doi:10.1158/1055-9965.EPI-08-0497 (2008).

44 Eaglehouse, Y. L. *et al.* Physical activity, sedentary time, and risk of colorectal cancer: the Singapore Chinese Health Study. *Eur J Cancer Prev* **26**, 469-475, doi:10.1097/CEJ.0000000000000369 (2017).

45 Mok, Y., Jeon, C., Lee, G. J. & Jee, S. H. Physical Activity Level and Colorectal Cancer Mortality. *Asia Pac J Public Health* **28**, 638-647, doi:10.1177/1010539516661761 (2016).

46 Yun, Y. H. *et al.* Dietary preference, physical activity, and cancer risk in men: national health insurance corporation study. *Bmc Cancer* **8**, 366, doi:10.1186/1471-2407-8-366 (2008).

47 Smith, A. D., Crippa, A., Woodcock, J. & Brage, S. Physical activity and incident type 2 diabetes mellitus: a systematic review and dose-response meta-analysis of prospective cohort studies. *Diabetologia* **59**, 2527-2545, doi:10.1007/s00125-016-4079-0 (2016).

48 Liu, X. J. *et al.* Dose-Response Association Between Physical Activity and Incident Hypertension A Systematic Review and Meta-Analysis of Cohort Studies. *Hypertension* **69**, 813-+, doi:10.1161/Hypertensionaha.116.08994 (2017).

49 Moore, S. C. *et al.* Association of Leisure-Time Physical Activity With Risk of 26 Types of Cancer in 1.44 Million Adults. *JAMA Intern Med* **176**, 816-825, doi:10.1001/jamainternmed.2016.1548 (2016).

50 Stranges, S. *et al.* Relationship of alcohol drinking pattern to risk of hypertension: a population-based study. *Hypertension* **44**, 813-819, doi:10.1161/01.HYP.0000146537.03103.f2 (2004).

51 Niskanen, L. *et al.* Inflammation, abdominal obesity, and smoking as predictors of hypertension. *Hypertension* **44**, 859-865, doi:10.1161/01.HYP.0000146691.51307.84 (2004).

52 Halperin, R. O., Gaziano, J. M. & Sesso, H. D. Smoking and the risk of incident hypertension in middle-aged and older men. *Am J Hypertens* **21**, 148-152, doi:10.1038/ajh.2007.36 (2008).

53 Won, T., Kang, B., Im, T. & Choi, H. The study of accuracy of death statistics. *J Korean Soc Emerg Med* **18**, 256–262 (2007).

Figure Legends

Figure 1. A flow diagram. Note: “N” indicates numbers of total observations (i.e. participants who provided repeated measures are treated as separate observations) and “n” indicates numbers of unique participants at baseline. Data without missingness and prevalence of major diseases were used to create final analysis datasets for different incident disease outcomes; while the number of unique participants at baseline is the same for all incident disease outcomes, the total number of observations varied due to the nature of time-updated covariate analyses (i.e. censoring of subsequent time-updated covariates when an incident

For peer review only

disease case occurs before the end date of repeated measures). Data were obtained from a prospective cohort, which has been established by linking physical examination data of over half a million Korean adults (2002-2015) with the entire Korean population's health insurance system.

Figure 2. Associations of exercise frequency with various incident cardiovascular disease outcomes. Cox regression models with age as the underlying timescale were adjusted for sex, body mass index, systolic blood pressure, fasting glucose levels, total cholesterol levels, family history of heart disease, stroke or hypertension (in models for myocardial infarction, stroke and hypertension) or diabetes (in models for Type 2 diabetes), smoking status and alcohol consumption. Crude rates are per 100,000 person-years. "N" indicates numbers of total observations (i.e. participants who provided repeated measures are treated as separate observations) and "n" indicates numbers of unique participants at baseline. Data were obtained from a prospective cohort, which has been established by linking physical examination data of over half a million Korean adults (2002-2015) with the entire Korean population's health insurance system. Abbreviations: HR – Hazard Ratio; CI – Confidence Intervals

Figure 3. Associations of exercise frequency with various incident cancer outcomes. Cox regression models with age as the underlying timescale were adjusted for sex, body mass index, systolic blood pressure, fasting glucose levels, total cholesterol levels, family history of cancer, smoking status and alcohol consumption. Crude rates are per 100,000 person-years. "N" indicates numbers of total observations (i.e. participants who provided repeated measures are treated as separate observations) and "n" indicates numbers of unique participants at baseline. Data were obtained from a prospective cohort, which has been established by linking physical examination data of over half a million Korean adults (2002-2015) with the entire Korean population's health insurance system. Abbreviations: HR – Hazard Ratio; CI – Confidence Intervals

Figure 4. Results from assessment of effect modification of sex, body mass index, smoking, alcohol consumption, and family history of disease in the associations between exercise frequency and each incident disease outcome. Only associations for which the multiplicative interaction terms were statistically significant are presented. Cox regression models with age as the underlying timescale were adjusted for sex [not in models for effect modification by sex], body mass index [not in models for effect modification by body mass index], systolic blood pressure, fasting glucose levels, total cholesterol levels, family history [not in models for effect modification by family history of respective disease] of heart disease/stroke/hypertension (in models for myocardial infarction, stroke and hypertension), diabetes (in models for Type 2 diabetes) or cancer (in models for each cancer), smoking status [not in models for effect modification by smoking status] and alcohol consumption [not in models for effect modification by alcohol consumption]. Crude rates are per 100,000 person-years. "N" indicates numbers of total observations (i.e. participants who provided repeated measures are treated as separate observations) and "n" indicates numbers of unique participants at baseline. P-values for multiplicative interactions with exercise frequency are as follows; Outcome – hypertension: body mass index (0.049), smoking (<0.001), alcohol consumption (<0.001), family history of cardiovascular disease (0.029) and sex (<0.001); Outcome - lung cancer: body mass index (0.016), and; Outcome - type 2 diabetes: sex (0.012). Data were obtained from a prospective cohort, which has been established by linking physical examination data of over half a million Korean adults (2002-2015) with the entire Korean population's health insurance system. Abbreviations: HR – Hazard Ratio; CI – Confidence Intervals

For peer review only

Table 1. Characteristics of the participants at baseline.

Variables	All (n=257,854)	Exercise frequency				
		None (n=148,284)	1-2 times/week (n=62,923)	3-4 times/week (n=24,836)	5-6 times/week (n=6,676)	Almost everyday (n=15,135)
Sex, %						
Men	50.5%	43.8%	63.3%	57.5%	52.9%	49.6%
Women	49.5%	56.2%	36.7%	42.5%	47.1%	50.4%
Age, years	50.7 (8.7)	51.5 (9.2)	49.0 (7.5)	49.3 (7.5)	49.9 (7.8)	53.5 (9.3)
Body Mass Index, kg^2/m	23.5 (2.8)	23.3 (2.9)	23.6 (2.7)	23.7 (2.6)	23.7 (2.6)	23.7 (2.7)
Systolic blood pressure, mmHg	116.8 (11.2)	116.6 (11.3)	117.1 (11.0)	116.8 (11.1)	116.8 (11.2)	117.3 (11.3)
Diastolic blood pressure, mmHg	73.4 (7.9)	73.1 (8.0)	73.8 (7.8)	73.5 (7.9)	73.4 (8.0)	73.4 (7.9)
Fasting glucose levels, mg/dL	90.2 (12.3)	90.1 (12.4)	90.4 (12.2)	89.9 (11.9)	90.2 (12.3)	90.2 (12.4)
Total cholesterol, mg/dL	197.0 (36.7)	196.5 (37.1)	197.7 (36.2)	197.8 (35.9)	197.8 (36.1)	197.4 (36.8)
Family history of heart disease, stroke or hypertension, %	12.2%	10.8%	13.9%	15.9%	16.5%	10.9%
Family history of cancer, %	14.2%	13.1%	15.3%	17.2%	16.6%	14.5%
Family history of diabetes, %	6.3%	5.4%	7.2%	8.5%	9.2%	5.7%
Smoking status, %						
Never	68.1%	71.9%	59.1%	65.2%	68.8%	71.7%
Previously	8.4%	5.8%	12.0%	12.7%	12.5%	8.8%
Currently	23.6%	22.2%	28.9%	22.1%	18.8%	19.5%
Alcohol Consumption, %						
Never	58.4%	64.5%	47.6%	50.5%	52.2%	59.3%
2-3 times/month	16.4%	13.8%	21.3%	19.7%	18.8%	14.1%
1-2 times/week	15.8%	12.4%	22.0%	20.2%	18.1%	14.5%
≥3 times/week	9.5%	9.3%	9.1%	9.5%	10.8%	12.1%
Incident myocardial infarction, n (%)	3,047 (1.2)	1,741 (1.2)	723 (1.1)	276 (1.1)	88 (1.3)	219 (1.4)
Incident stroke, n (%)	16,134 (6.3)	9,689 (6.5)	3,333 (5.3)	1,482 (6.0)	390 (5.8)	1,240 (8.2)
Incident hypertension, n (%)	120,203 (46.6)	65,964 (44.5)	30,623 (48.7)	12,617 (50.8)	3,294 (49.3)	7,705 (50.9)
Incident Type 2 diabetes, n (%)	50,459 (19.6)	27,128 (18.3)	10,666 (6.5)	5,421 (21.8)	1,399 (21.0)	3,285 (21.7)
Incident stomach cancer, n (%)	4,788 (1.9)	2,672 (1.8)	13,226 (21.0)	489 (2.0)	139 (2.1)	328 (2.2)
Incident colon cancer, n (%)	2,711 (1.1)	1,424 (1.0)	1,160 (1.8)	314 (1.3)	90 (1.3)	191 (1.3)
Incident rectum cancer, n (%)	1,494 (0.6)	809 (0.6)	692 (1.1)	154 (0.6)	46 (0.6)	107 (0.7)
Incident lung cancer, n (%)	3,601 (1.4)	2,138 (1.4)	796 (1.3)	307 (1.2)	85 (1.3)	275 (1.8)
Incident liver cancer, n (%)	2,620 (1.0)	1,423 (1.0)	680 (1.1)	263 (1.1)	75 (1.1)	179 (1.2)
Incident pancreas cancer, n (%)	864 (0.3)	483 (0.3)	205 (0.3)	92 (0.4)	24 (0.4)	60 (0.4)
Incident head & neck cancer, n (%)	656 (0.3)	377 (0.3)	144 (0.2)	73 (0.3)	15 (0.2)	47 (0.3)
Incident kidney cancer, n (%)	589 (0.2)	301 (0.2)	153 (0.2)	75 (0.3)	16 (0.2)	44 (0.3)
Incident gallbladder cancer, n (%)	400 (0.2)	219 (0.1)	83 (0.1)	43 (0.2)	16 (0.2)	39 (0.3)
Incident esophagus cancer, n (%)	352 (0.1)	214 (0.1)	75 (0.1)	29 (0.1)	6 (0.09)	28 (0.2)
Median follow-up period, years (interquartile range)	13.0 (12.2, 13.3)	13.0 (12.2, 13.3)	13.0 (12.2, 13.3)	13.0 (12.2, 13.3)	12.7 (12.2, 13.3)	12.6 (12.2, 13.3)

For peer review only

Note: Values presented are means unless indicated as an ‘n’. Values in parentheses are standard deviations unless otherwise indicated. Data were obtained from a prospective cohort, which has been established by linking physical examination data of over half a million Korean adults (2002-2015) with the entire Korean population’s health insurance system.

For peer review only

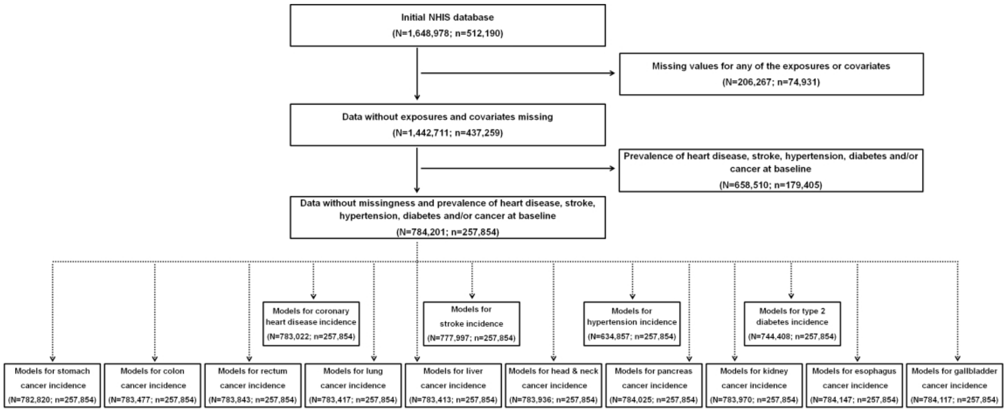

A flow diagram. Note: “N” indicates numbers of total observations (i.e. participants who provided repeated measures are treated as separate observations) and “n” indicates numbers of unique participants at baseline. Data without missingness and prevalence of major diseases were used to create final analysis datasets for different incident disease outcomes; while the number of unique participants at baseline is the same for all incident disease outcomes, the total number of observations varied due to the nature of time-updated covariate analyses (i.e. censoring of subsequent time-updated covariates when an incident disease case occurs before the end date of repeated measures). Data were obtained from a prospective cohort, which has been established by linking physical examination data of over half a million Korean adults (2002-2015) with the entire Korean population’s health insurance system.

1412x571mm (72 x 72 DPI)

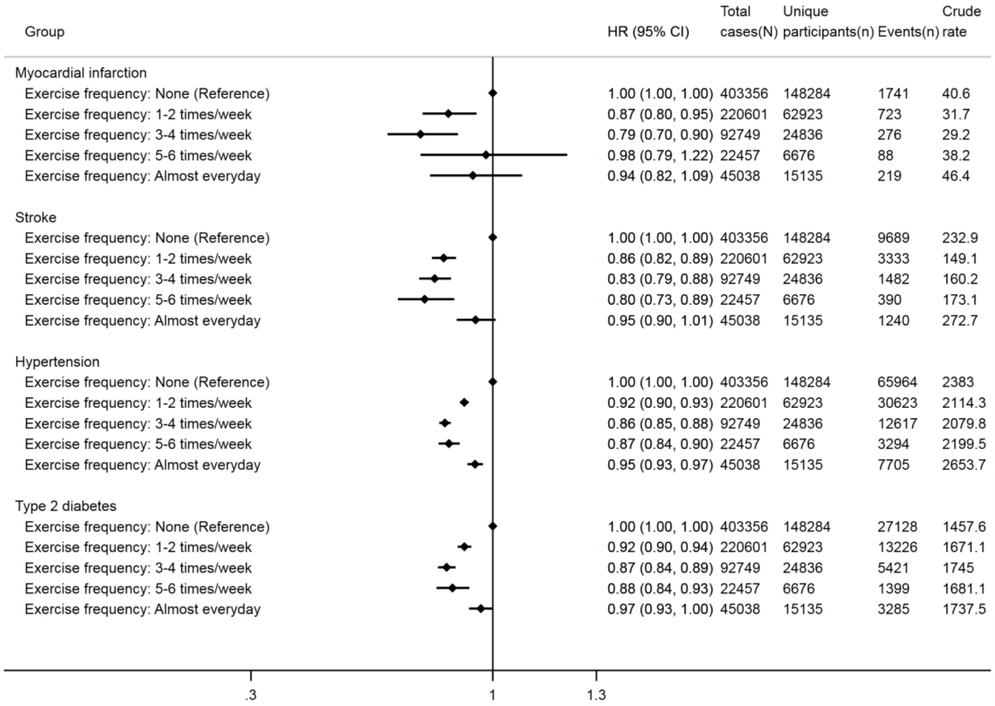

Associations of exercise frequency with various incident cardiovascular disease outcomes. Cox regression models with age as the underlying timescale were adjusted for sex, body mass index, systolic blood pressure, fasting glucose levels, total cholesterol levels, family history of heart disease, stroke or hypertension (in models for myocardial infarction, stroke and hypertension) or diabetes (in models for Type 2 diabetes), smoking status and alcohol consumption. Crude rates are per 100,000 person-years. "N" indicates numbers of total observations (i.e. participants who provided repeated measures are treated as separate observations) and "n" indicates numbers of unique participants at baseline. Data were obtained from a prospective cohort, which has been established by linking physical examination data of over half a million Korean adults (2002-2015) with the entire Korean population's health insurance system.
Abbreviations: HR – Hazard Ratio; CI – Confidence Intervals

881x671mm (72 x 72 DPI)

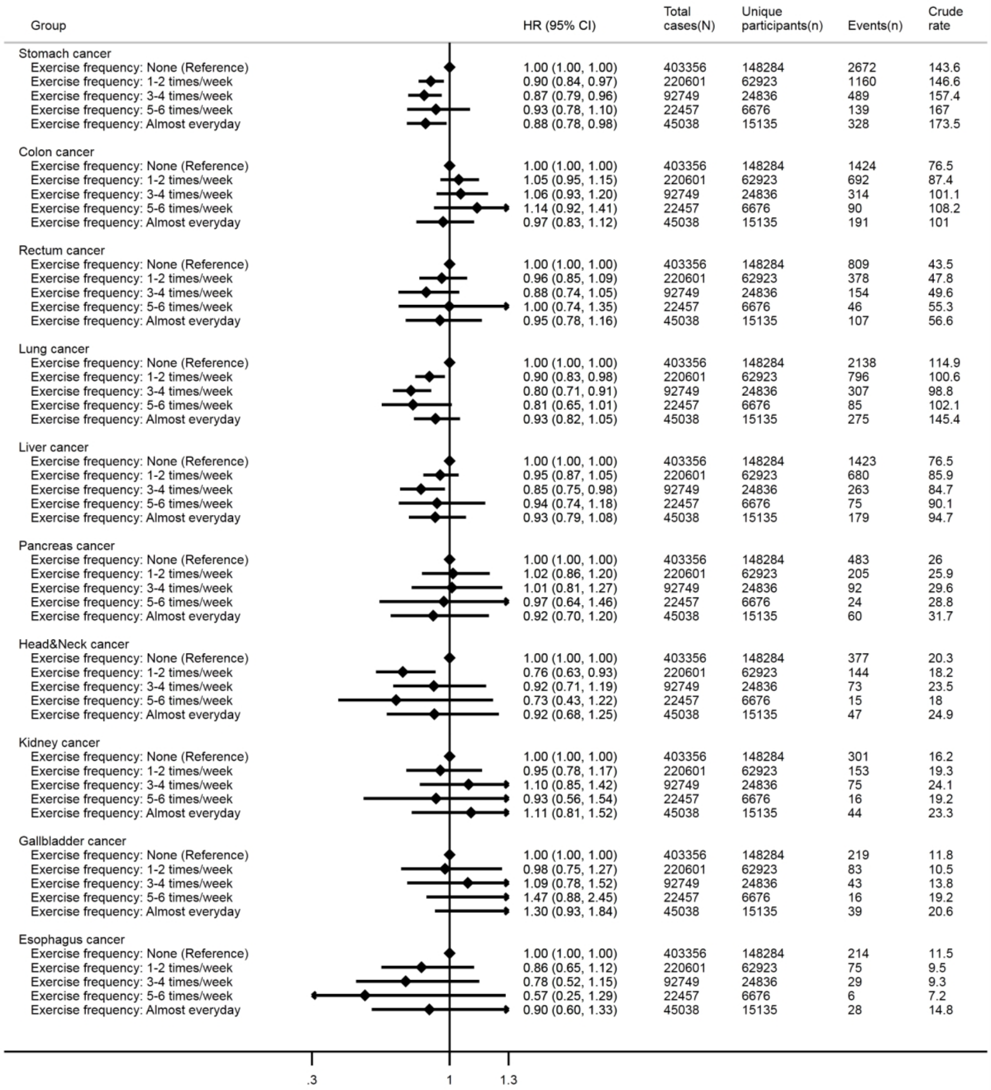

Associations of exercise frequency with various incident cancer outcomes. Cox regression models with age as the underlying timescale were adjusted for sex, body mass index, systolic blood pressure, fasting glucose levels, total cholesterol levels, family history of cancer, smoking status and alcohol consumption. Crude rates are per 100,000 person-years. “N” indicates numbers of total observations (i.e. participants who provided repeated measures are treated as separate observations) and “n” indicates numbers of unique participants at baseline. Data were obtained from a prospective cohort, which has been established by linking physical examination data of over half a million Korean adults (2002-2015) with the entire Korean population’s health insurance system. Abbreviations: HR – Hazard Ratio; CI – Confidence Intervals

881x975mm (72 x 72 DPI)

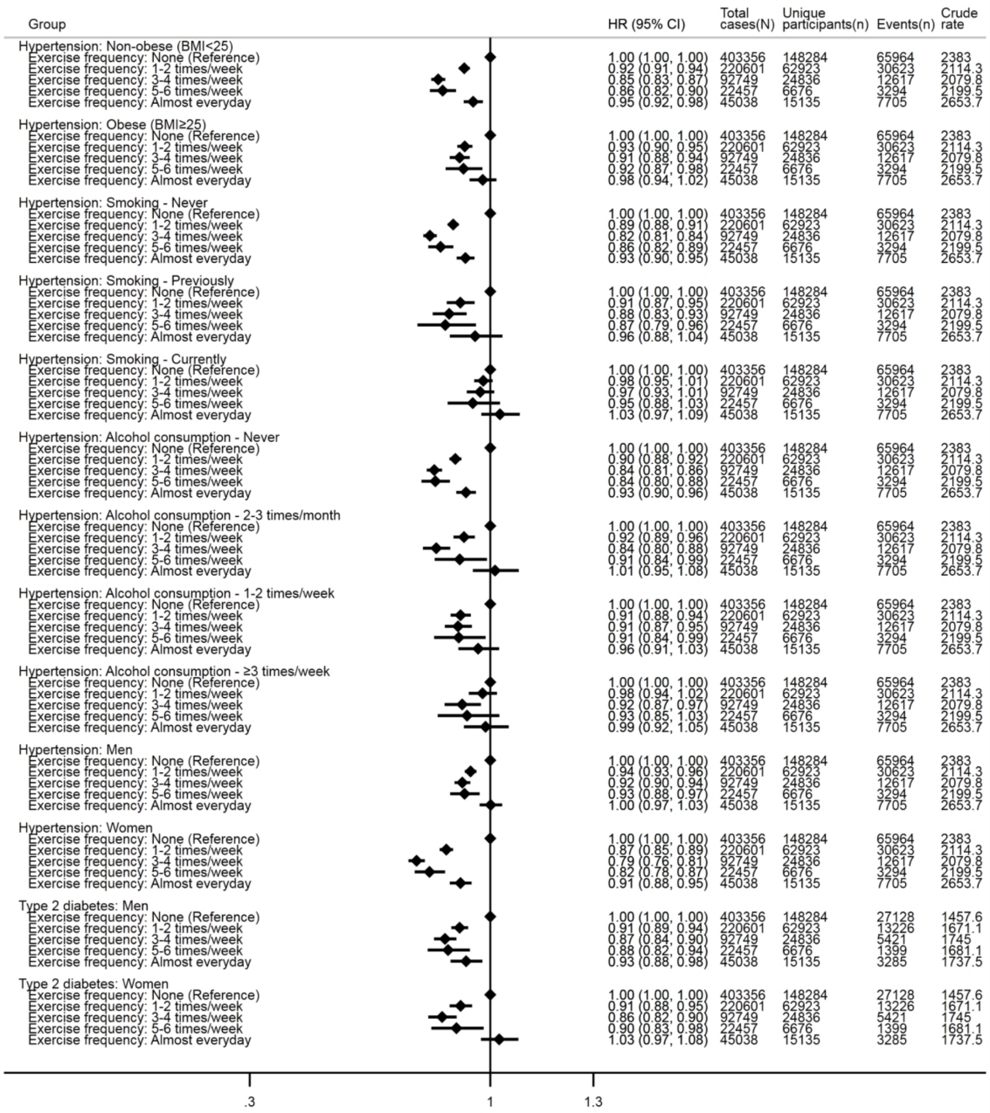

Results from assessment of effect modification of sex, body mass index, smoking, alcohol consumption, and family history of disease in the associations between exercise frequency and each incident disease outcome. Only associations for which the multiplicative interaction terms were statistically significant are presented. Cox regression models with age as the underlying timescale were adjusted for sex [not in models for effect modification by sex], body mass index [not in models for effect modification by body mass index], systolic blood pressure, fasting glucose levels, total cholesterol levels, family history [not in models for effect modification by family history of respective disease] of heart disease/stroke/hypertension (in models for myocardial infarction, stroke and hypertension), diabetes (in models for Type 2 diabetes) or cancer (in models for each cancer), smoking status [not in models for effect modification by smoking status] and alcohol consumption [not in models for effect modification by alcohol consumption]. Crude rates are per 100,000 person-years. "N" indicates numbers of total observations (i.e. participants who provided repeated measures are treated as separate observations) and "n" indicates numbers of unique participants at baseline. P-values for multiplicative interactions with exercise frequency are as follows; Outcome – hypertension: body mass index (0.049), smoking (<0.001), alcohol consumption (<0.001), family history of cardiovascular disease (0.029) and sex (<0.001); Outcome - lung cancer: body mass index (0.016), and; Outcome - type 2 diabetes: sex (0.012)¬¬¬¬¬¬¬. Data were obtained from a prospective cohort, which has been established by linking physical examination data of over half a million Korean adults (2002-2015) with

the entire Korean population’s health insurance system. Abbreviations: HR – Hazard Ratio; CI – Confidence Intervals

Supplementary Table 1. Definitions for classification of incident disease outcomes

Incident disease outcome	Definition
Myocardial infarction	ICD 10 codes I21-I23
Stroke	ICD 10 codes I60-I64
Hypertension	ICD 10 codes I10, I11 and I15, systolic blood pressure ≥140 mmHg, diastolic blood pressure ≥90 mmHg, or physician diagnosis
Type 2 diabetes	ICD 10 codes E11, fasting glucose ≥126 mg/dL, or physician diagnosis
Stomach cancer	ICD 10 codes C16
Colon cancer	ICD 10 codes C18
Rectum cancer	ICD 10 codes C20
Lung cancer	ICD 10 codes C34
Liver cancer	ICD 10 codes C22
Pancreas cancer	ICD 10 codes C25
Head & neck cancer	ICD 10 codes C00-C06, C09-C14 and C32
Kidney cancer	ICD 10 codes C64
Gallbladder cancer	ICD 10 codes C23
Esophagus cancer	ICD 10 codes C15

Abbreviations: ICD – International Classification of Disease

For peer review only

Supplementary Table 2. Participants' characteristics at 1st, 2nd, 3rd, 4th, 5th and 6th repeat-visit

Repeat visit	Variables	All (n=215,295)	Exercise frequency				
1st			None (n=112,156)	1-2 times/week (n=57,777)	3-4 times/week (n=25,390)	5-6 times/week (n= 6,443)	Almost everyday (n=13,529)
	Sex, %						
	Men	51.1%	45.0%	61.8%	55.3%	50.6%	48.8%
	Women	48.9%	55.0%	38.2%	44.7%	49.4%	51.2%
	Age, years	52.7 (8.6)	53.6 (9.2)	50.9 (7.4)	51.3 (7.5)	52.3 (8.0)	55.6 (9.1)
	Body Mass Index, kg^2/m	23.5 (2.8)	23.4 (2.9)	23.6 (2.7)	23.7 (2.6)	23.6 (2.6)	23.7 (2.7)
	Systolic blood pressure, mmHg	120.5 (14.4)	120.6 (14.7)	120.4 (13.9)	120.0 (13.9)	119.6 (13.9)	121.4 (14.6)
	Diastolic blood pressure, mmHg	75.7 (9.9)	75.6 (9.9)	75.9 (9.8)	75.5 (9.8)	75.2 (9.8)	75.7 (9.8)
	Fasting glucose levels, mg/dL	92.6 (18.5)	92.6 (18.8)	92.7 (17.4)	92.4 (17.8)	92.8 (21.0)	93.3 (20.2)
	Total cholesterol, mg/dL	197.3 (36.2)	197.0 (36.8)	197.3 (35.6)	197.9 (35.1)	197.4 (35.9)	197.8 (36.7)
	Family history of heart disease, stroke or hypertension, %	12.6%	10.7%	14.2%	16.7%	16.7%	12.0%
	Family history of cancer, %	14.3%	12.7%	15.5%	17.4%	19.2%	14.5%
	Family history of diabetes, %	6.0%	5.0%	6.7%	8.2%	9.0%	5.8%
	Smoking status, %						
	Never	70.7%	74.8%	62.4%	68.9%	71.7%	74.8%
	Previously	8.6%	5.8%	11.9%	12.4%	11.9%	8.8%
	Currently	20.8%	19.4%	25.7%	18.7%	16.3%	16.4%
	Alcohol Consumption, %						
	Never	59.8%	66.9%	49.2%	52.7%	54.9%	61.8%
	2-3 times/month	15.6%	12.7%	20.5%	18.6%	18.2%	12.8%
	1-2 times/week	15.8%	11.8%	21.7%	19.9%	17.0%	14.8%
	≥3 times/week	8.8%	8.7%	8.6%	8.7%	10.0%	10.6%

For peer review only

Supplementary Table 2. Participants' characteristics at 1st, 2nd, 3rd, 4th, 5th and 6th repeat-visit (cont.)

Repeat visit 2nd	Variables	All (n=150,845)	Exercise frequency				
			None (n=73,660)	1-2 times/week (n=44,211)	3-4 times/week (n=19,593)	5-6 times/week (n=4,771)	Almost everyday (n= 8,610)
	Sex, %						
	Men	54.2%	47.0%	64.6%	57.9%	53.8%	53.5%
	Women	45.8%	53.0%	35.4%	42.1%	46.2%	46.5%
	Age, years	53.3 (8.3)	54.2 (8.9)	51.7 (7.2)	52.3 (7.4)	53.4 (7.8)	56.5 (9.1)
	Body Mass Index, kg^2/m	23.5 (2.7)	23.4 (2.8)	23.6 (2.6)	23.7 (2.6)	23.6 (2.6)	23.6 (2.7)
	Systolic blood pressure, mmHg	120.9 (14.1)	121.0 (14.5)	120.7 (13.7)	120.4 (13.6)	120.3 (13.7)	122.0 (14.5)
	Diastolic blood pressure, mmHg	75.8 (9.6)	75.8 (9.7)	76.0 (9.5)	75.5 (9.4)	75.4 (9.4)	76.0 (9.8)
	Fasting glucose levels, mg/dL	93.2 (16.9)	93.1 (17.4)	93.5 (17.0)	93.1 (15.5)	93.5 (15.9)	93.7 (16.2)
	Total cholesterol, mg/dL	197.6 (35.9)	197.7 (36.5)	197.4 (35.4)	197.8 (35.2)	196.7 (34.9)	198.1 (36.5)
	Family history of heart disease, stroke or hypertension, %	13.4%	11.2%	14.6%	17.5%	18.3%	13.1%
	Family history of cancer, %	14.9%	12.9%	16.4%	17.4%	19.7%	15.4%
	Family history of diabetes, %	6.3%	5.2%	7.1%	8.2%	9.0%	6.1%
	Smoking status, %						
	Never	71.0%	76.5%	62.3%	68.7%	71.2%	74.2%
	Previously	9.0%	5.5%	12.5%	13.1%	13.1%	9.4%
	Currently	20.0%	18.0%	25.3%	18.2%	15.8%	16.5%
	Alcohol Consumption, %						
	Never	59.0%	67.6%	48.1%	51.9%	54.1%	60.0%
	2-3 times/month	16.1%	12.6%	20.9%	19.0%	18.9%	13.6%
	1-2 times/week	16.7%	12.1%	22.8%	20.7%	17.5%	15.8%
	≥3 times/week	8.1%	7.8%	8.2%	8.3%	9.5%	10.7%

For peer review only

Supplementary Table 2. Participants' characteristics at 1st, 2nd, 3rd, 4th, 5th and 6th repeat-visit (cont.)

Repeat visit	Variables	All (n=80,639)	Exercise frequency				
3rd			None (n= 36,123)	1-2 times/week (n=26,505)	3-4 times/week (n=11,432)	5-6 times/week (n=2,391)	Almost everyday (n=4,188)
	Sex, %						
	Men	63.1%	53.9%	73.0%	68.6%	64.5%	63.4%
	Women	36.9%	46.1%	27.0%	31.4%	35.6%	36.7%
	Age, years	52.3 (7.2)	52.9 (7.8)	51.2 (6.2)	51.9 (6.6)	52.8 (7.0)	55.2 (8.4)
	Body Mass Index, kg^2/m	23.5 (2.7)	23.3 (2.8)	23.6 (2.6)	23.7 (2.5)	23.7 (2.6)	23.7 (2.7)
	Systolic blood pressure, mmHg	121.1 (13.6)	120.9 (13.9)	120.9 (13.4)	121.0 (13.2)	120.6 (13.3)	122.0 (14.0)
	Diastolic blood pressure, mmHg	76.1 (9.4)	76.0 (9.5)	76.4 (9.4)	76.1 (9.1)	75.6 (9.5)	76.3 (9.6)
	Fasting glucose levels, mg/dL	93.7 (16.8)	93.3 (17.1)	94.1 (16.7)	93.9 (15.9)	94.1 (16.5)	94.5 (17.0)
	Total cholesterol, mg/dL	197.1 (35.2)	197.1 (35.5)	197.4 (34.8)	196.9 (34.7)	196.5 (35.2)	196.7 (35.4)
	Family history of heart disease, stroke or hypertension, %	13.5%	11.4%	14.8%	16.7%	18.6%	11.7%
	Family history of cancer, %	14.8%	13.0%	15.8%	17.8%	18.9%	14.7%
	Family history of diabetes, %	6.4%	5.3%	7.0%	8.0%	9.5%	6.2%
	Smoking status, %						
	Never	66.6%	73.8%	57.6%	63.9%	65.5%	68.8%
	Previously	10.7%	6.1%	14.0%	15.5%	16.6%	12.4%
	Currently	22.8%	20.2%	28.4%	20.6%	17.9%	18.7%
	Alcohol Consumption, %						
	Never	53.6%	64.2%	42.5%	47.2%	47.4%	53.9%
	2-3 times/month	18.5%	14.0%	23.7%	20.8%	21.4%	15.8%
	1-2 times/week	19.5%	14.0%	25.6 %	22.8%	20.7%	18.8%
	≥3 times/week	8.4%	7.8%	8.3%	9.2%	10.5%	11.5%

For peer review only

Supplementary Table 2. Participants' characteristics at 1st, 2nd, 3rd, 4th, 5th and 6th repeat-visit (cont.)

Repeat visit	Variables	All (n=40,910)	Exercise frequency				
4th			None (n=17,678)	1-2 times/week (n=14,624)	3-4 times/week (n= 5,685)	5-6 times/week (n=1,094)	Almost everyday (n=1,829)
	Sex, %						
	Men	72.3%	61.6%	81.1%	81.5%	78.2%	73.9%
	Women	27.7%	38.4%	18.9%	18.5%	21.9%	26.1%
	Age, years	50.9 (5.4)	51.2 (5.5)	50.6 (5.1)	50.7 (5.3)	51.0 (5.6)	52.6 (6.3)
	Body Mass Index, kg^2/m	23.5 (2.7)	23.3 (2.7)	23.7 (2.6)	23.8 (2.6)	23.8 (2.5)	23.7 (2.6)
	Systolic blood pressure, mmHg	121.4 (13.3)	120.9 (13.5)	121.7 (13.0)	121.5 (12.8)	121.5 (13.1)	122.7 (13.6)
	Diastolic blood pressure, mmHg	76.6 (9.2)	76.2 (9.3)	77.0 (9.2)	76.8 (8.9)	76.8 (9.4)	77.2 (9.5)
	Fasting glucose levels, mg/dL	93.9 (17.8)	93.3 (17.9)	94.4 (17.9)	94.3 (17.1)	94.0 (16.0)	94.9 (18.8)
	Total cholesterol, mg/dL	196.8 (35.0)	197.2 (35.5)	196.7 (34.6)	196.6 (34.2)	195.0 (34.3)	196.5 (34.9)
	Family history of heart disease, stroke or hypertension, %	12.3%	10.4%	13.7%	14.2%	16.3%	11.6%
	Family history of cancer, %	13.8%	12.6%	14.7%	15.0%	16.3%	13.5%
	Family history of diabetes, %	5.7%	4.8%	6.2%	6.9%	7.5%	6.7%
	Smoking status, %						
	Never	60.6%	69.5%	51.9%	55.5%	57.3%	62.0%
	Previously	12.2%	6.9%	15.1%	18.7%	18.7%	15.7%
	Currently	27.2%	23.7%	33.0%	25.8%	24.0%	22.4%
	Alcohol Consumption, %						
	Never	48.1%	60.0%	38.0%	39.1%	39.9%	45.4%
	2-3 times/month	20.5%	15.5%	24.9%	24.3%	24.0%	19.7%
	1-2 times/week	22.4%	16.2%	28.4%	26.3%	22.6%	22.9%
	≥3 times/week	9.0%	8.2%	8.7%	10.3%	13.6%	12.0%

For peer review only

Supplementary Table 2. Participants' characteristics at 1st, 2nd, 3rd, 4th, 5th and 6th repeat-visit (cont.)

Repeat visit 5th	Variables	All (n= 26,356)	Exercise frequency				
			None (n=10,771)	1-2 times/week (n=9,807)	3-4 times/week (n=3,884)	5-6 times/week (n=724)	Almost everyday (n=1,170)
	Sex, %						
	Men	73.7%	62.6%	81.6%	82.4%	81.2%	76.9%
	Women	26.3%	37.4%	18.4%	17.6%	18.8%	23.0%
	Age, years	51.1 (4.9)	51.4 (5.1)	50.8 (4.7)	51.0 (4.7)	51.4 (5.0)	52.1 (5.6)
	Body Mass Index, kg^2/m	23.5 (2.6)	23.3 (2.7)	23.7 (2.6)	23.8 (2.4)	23.7 (2.5)	23.8 (2.6)
	Systolic blood pressure, mmHg	121.4 (13.0)	121.0 (13.4)	121.8 (12.9)	121.6 (12.6)	120.8 (12.9)	121.5 (12.7)
	Diastolic blood pressure, mmHg	76.6 (9.1)	76.2 (9.1)	77.1 (9.0)	76.8 (8.8)	76.7 (8.6)	76.4 (9.0)
	Fasting glucose levels, mg/dL	94.3 (17.6)	93.7 (18.1)	94.6 (17.3)	94.7 (16.6)	94.6 (15.9)	95.4 (18.7)
	Total cholesterol, mg/dL	197.1 (34.5)	197.7 (34.8)	196.7 (34.2)	196.4 (33.8)	195.2 (34.2)	198.2 (37.0)
	Family history of heart disease, stroke or hypertension, %	12.6%	11.3%	13.4%	14.3%	13.8%	10.9%
	Family history of cancer, %	14.3%	13.2%	15.1%	14.9%	17.8%	13.5%
	Family history of diabetes, %	5.9%	4.9%	6.4%	6.7%	7.2%	8.6%
	Smoking status, %						
	Never	58.9%	68.1%	50.8%	54.4%	57.0%	58.5%
	Previously	13.1%	7.1%	16.0%	19.3%	19.8%	19.7%
	Currently	28.0%	24.8%	33.2%	26.2%	23.2%	21.9%
	Alcohol Consumption, %						
	Never	46.3%	57.9%	37.6%	38.3%	41.3%	42.9%
	2-3 times/month	21.7%	16.7%	25.8%	24.7%	22.2%	22.3%
	1-2 times/week	23.4%	17.4%	28.0%	27.3%	26.0%	25.0%
	≥3 times/week	8.6%	8.1%	8.5%	9.8%	10.5%	9.7%

For peer review only

Supplementary Table 2. Participants' characteristics at 1st, 2nd, 3rd, 4th, 5th and 6th repeat-visit (cont.)

Repeat visit	Variables	All (n= 12,302)	Exercise frequency				
6th			None (n= 4,684)	1-2 times/week (n=4,754)	3-4 times/week (n=1,929)	5-6 times/week (n=358)	Almost everyday (n=577)
	Sex, %						
	Men	76.2%	64.2%	83.8%	84.7%	80.7%	80.4%
	Women	23.8%	35.8%	16.2%	15.3%	19.3%	19.6%
	Age, years	51.3 (4.5)	51.6 (4.7)	51.0 (4.2)	51.0 (4.4)	51.2 (4.3)	52.0 (5.1)
	Body Mass Index, kg^2/m	23.6 (2.6)	23.3 (2.7)	23.7 (2.6)	23.8 (2.4)	23.6 (2.2)	23.7 (2.5)
	Systolic blood pressure, mmHg	121.5 (12.8)	121.2 (13.0)	121.6 (12.6)	121.7 (12.6)	121.0 (12.5)	122.1 (12.3)
	Diastolic blood pressure, mmHg	76.7 (9.0)	76.4 (9.0)	77.0 (8.9)	77.0 (9.0)	76.0 (8.7)	76.3 (9.0)
	Fasting glucose levels, mg/dL	94.6 (18.5)	93.7 (18.8)	95.1 (18.7)	94.9 (16.6)	95.1 (18.4)	95.7 (20.1)
	Total cholesterol, mg/dL	197.1 (34.5)	197.9 (35.0)	197.1 (34.2)	195.8 (34.3)	192.0 (32.2)	197.4 (34.3)
	Family history of heart disease, stroke or hypertension, %	13.2%	11.7%	13.7%	15.5%	14.3%	12.7%
	Family history of cancer, %	14.5%	12.9%	15.5%	15.7%	15.1%	14.9%
	Family history of diabetes, %	6.4%	5.5%	6.4%	7.3%	7.3%	9.5%
	Smoking status, %						
	Never	56.9%	66.6%	48.7%	53.6%	57.3%	57.4%
	Previously	14.0%	7.0%	17.3%	20.4%	16.8%	21.1%
	Currently	29.1%	26.5%	34.1%	26.0%	26.0%	21.5%
	Alcohol Consumption, %						
	Never	43.7%	56.5%	35.0%	36.4%	40.5%	37.8%
	2-3 times/month	22.8%	17.0%	27.2%	24.9%	24.6%	24.8%
	1-2 times/week	24.8%	17.6%	30.0%	28.3%	26.5%	27.4%
	≥3 times/week	8.8%	8.8%	7.9%	10.4%	8.4%	10.1%

For peer review only

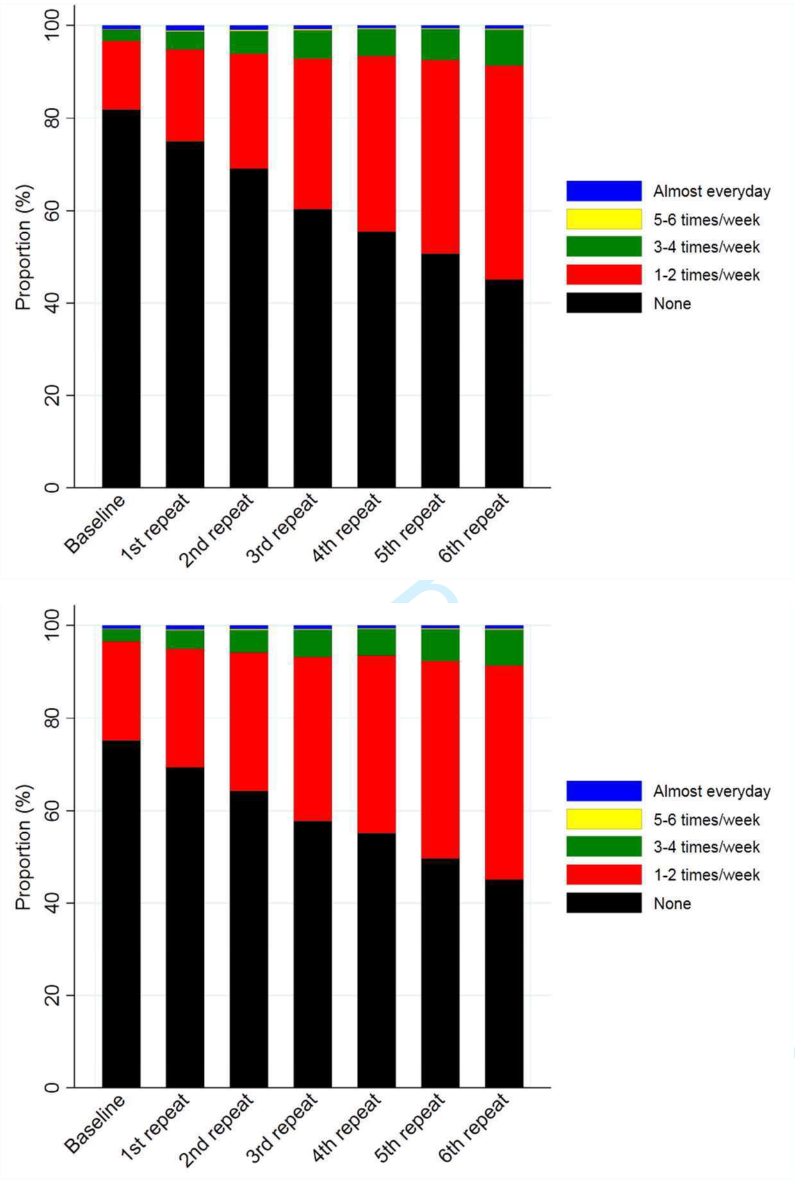

Supplementary Figure 1. Changes in proportions of exercise frequency categories across 7 time points in all 257,854 individuals who provided data at each respective assessment visit (top panel), and 12,302 individuals who provided data from all 7 assessment visits (bottom panel).

Group	HR (95% CI)	Total cases(N)	Unique participants(n)	Events(n)	Crude rate
Myocardial infarction					
Exercise frequency: None (Reference)	1.00 (1.00, 1.00)	403356	148284	1741	40.6
Exercise frequency: 1-2 times/week	0.98 (0.90, 1.07)	220601	62923	723	31.7
Exercise frequency: 3-4 times/week	0.83 (0.73, 0.94)	92749	24836	276	29.2
Exercise frequency: 5-6 times/week	0.98 (0.79, 1.22)	22457	6676	88	38.2
Exercise frequency: Almost everyday	0.97 (0.84, 1.12)	45038	15135	219	46.4
Stroke					
Exercise frequency: None (Reference)	1.00 (1.00, 1.00)	403356	148284	9689	232.9
Exercise frequency: 1-2 times/week	0.86 (0.82, 0.89)	220601	62923	3333	149.1
Exercise frequency: 3-4 times/week	0.83 (0.79, 0.88)	92749	24836	1482	160.2
Exercise frequency: 5-6 times/week	0.80 (0.72, 0.88)	22457	6676	390	173.1
Exercise frequency: Almost everyday	0.95 (0.90, 1.01)	45038	15135	1240	272.7
Hypertension					
Exercise frequency: None (Reference)	1.00 (1.00, 1.00)	403356	148284	65964	2383
Exercise frequency: 1-2 times/week	0.98 (0.96, 0.99)	220601	62923	30623	2114.3
Exercise frequency: 3-4 times/week	0.90 (0.89, 0.92)	92749	24836	12617	2079.8
Exercise frequency: 5-6 times/week	0.91 (0.88, 0.94)	22457	6676	3294	2199.5
Exercise frequency: Almost everyday	1.00 (0.97, 1.02)	45038	15135	7705	2653.7
Type 2 diabetes					
Exercise frequency: None (Reference)	1.00 (1.00, 1.00)	403356	148284	27128	1457.6
Exercise frequency: 1-2 times/week	1.00 (0.98, 1.02)	220601	62923	13226	1671.1
Exercise frequency: 3-4 times/week	0.91 (0.89, 0.94)	92749	24836	5421	1745
Exercise frequency: 5-6 times/week	0.92 (0.88, 0.97)	22457	6676	1399	1681.1
Exercise frequency: Almost everyday	1.01 (0.97, 1.04)	45038	15135	3285	1737.5

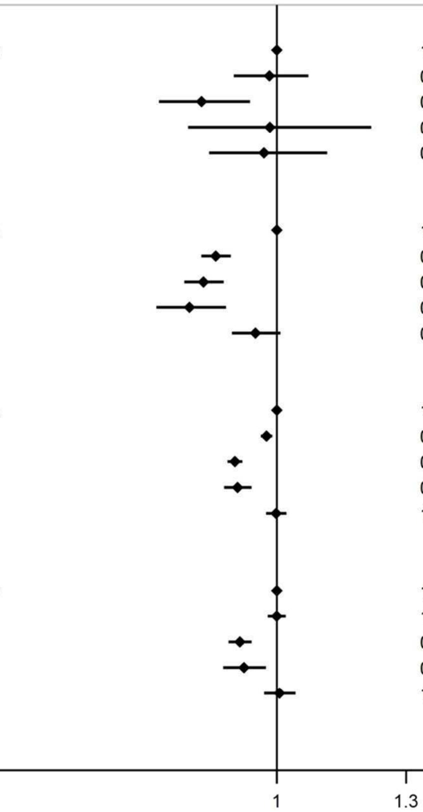

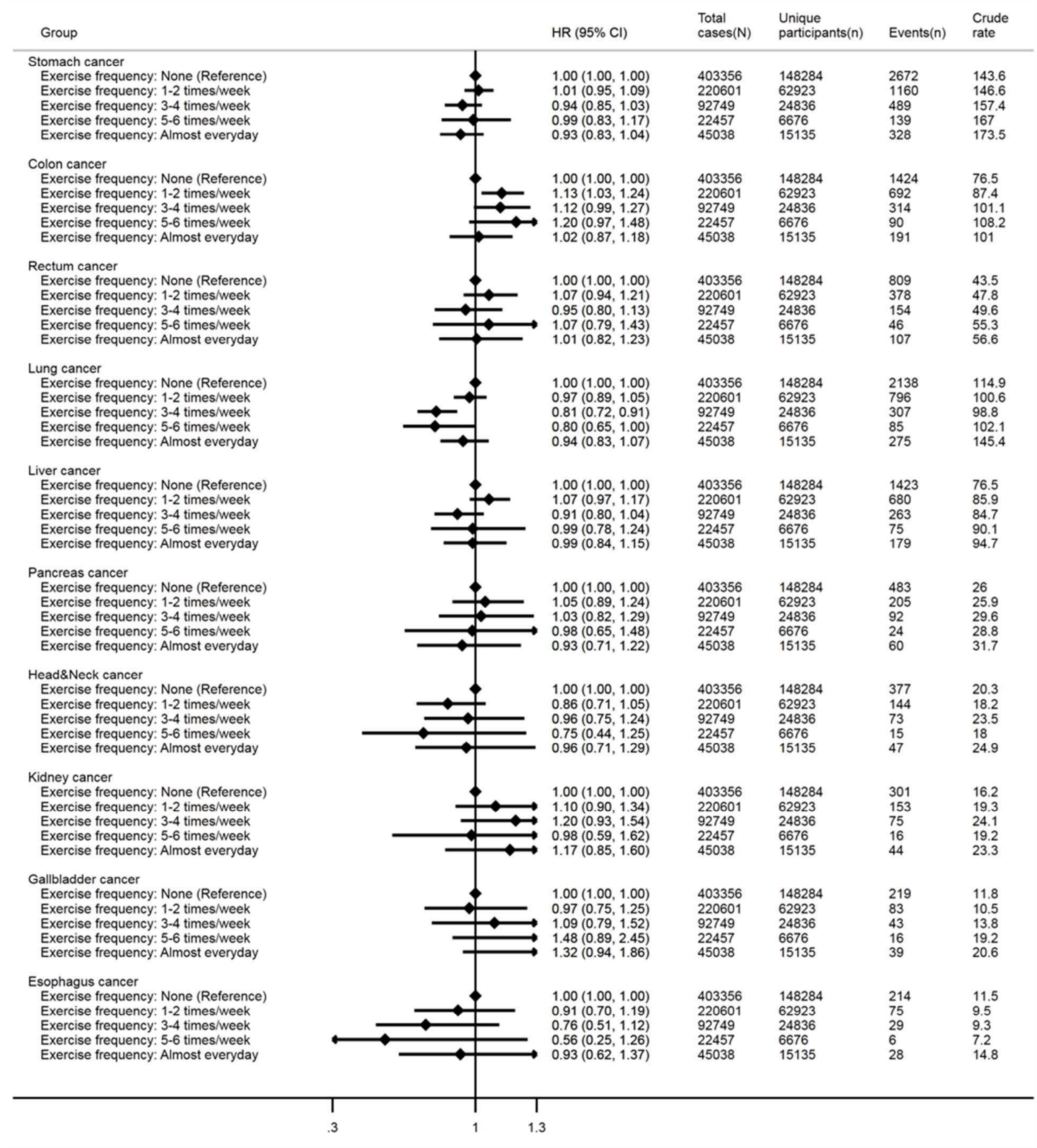

Group	HR (95% CI)	Total cases(N)	Unique participants(n)	Events(n)	Crude rate
**Stomach cancer**					
Exercise frequency: None (Reference)	1.00 (1.00, 1.00)	403356	148284	2672	143.6
Exercise frequency: 1-2 times/week	1.01 (0.95, 1.09)	220601	62923	1160	146.6
Exercise frequency: 3-4 times/week	0.94 (0.85, 1.03)	92749	24836	489	157.4
Exercise frequency: 5-6 times/week	0.99 (0.83, 1.17)	22457	6676	139	167
Exercise frequency: Almost everyday	0.93 (0.83, 1.04)	45038	15135	328	173.5
**Colon cancer**					
Exercise frequency: None (Reference)	1.00 (1.00, 1.00)	403356	148284	1424	76.5
Exercise frequency: 1-2 times/week	1.13 (1.03, 1.24)	220601	62923	692	87.4
Exercise frequency: 3-4 times/week	1.12 (0.99, 1.27)	92749	24836	314	101.1
Exercise frequency: 5-6 times/week	1.20 (0.97, 1.48)	22457	6676	90	108.2
Exercise frequency: Almost everyday	1.02 (0.87, 1.18)	45038	15135	191	101
**Rectum cancer**					
Exercise frequency: None (Reference)	1.00 (1.00, 1.00)	403356	148284	809	43.5
Exercise frequency: 1-2 times/week	1.07 (0.94, 1.21)	220601	62923	378	47.8
Exercise frequency: 3-4 times/week	0.95 (0.80, 1.13)	92749	24836	154	49.6
Exercise frequency: 5-6 times/week	1.07 (0.79, 1.43)	22457	6676	46	55.3
Exercise frequency: Almost everyday	1.01 (0.82, 1.23)	45038	15135	107	56.6
**Lung cancer**					
Exercise frequency: None (Reference)	1.00 (1.00, 1.00)	403356	148284	2138	114.9
Exercise frequency: 1-2 times/week	0.97 (0.89, 1.05)	220601	62923	796	100.6
Exercise frequency: 3-4 times/week	0.81 (0.72, 0.91)	92749	24836	307	98.8
Exercise frequency: 5-6 times/week	0.80 (0.65, 1.00)	22457	6676	85	102.1
Exercise frequency: Almost everyday	0.94 (0.83, 1.07)	45038	15135	275	145.4
**Liver cancer**					
Exercise frequency: None (Reference)	1.00 (1.00, 1.00)	403356	148284	1423	76.5
Exercise frequency: 1-2 times/week	1.07 (0.97, 1.17)	220601	62923	680	85.9
Exercise frequency: 3-4 times/week	0.91 (0.80, 1.04)	92749	24836	263	84.7
Exercise frequency: 5-6 times/week	0.99 (0.78, 1.24)	22457	6676	75	90.1
Exercise frequency: Almost everyday	0.99 (0.84, 1.15)	45038	15135	179	94.7
**Pancreas cancer**					
Exercise frequency: None (Reference)	1.00 (1.00, 1.00)	403356	148284	483	26
Exercise frequency: 1-2 times/week	1.05 (0.89, 1.24)	220601	62923	205	25.9
Exercise frequency: 3-4 times/week	1.03 (0.82, 1.29)	92749	24836	92	29.6
Exercise frequency: 5-6 times/week	0.98 (0.65, 1.48)	22457	6676	24	28.8
Exercise frequency: Almost everyday	0.93 (0.71, 1.22)	45038	15135	60	31.7
**Head&Neck cancer**					
Exercise frequency: None (Reference)	1.00 (1.00, 1.00)	403356	148284	377	20.3
Exercise frequency: 1-2 times/week	0.86 (0.71, 1.05)	220601	62923	144	18.2
Exercise frequency: 3-4 times/week	0.96 (0.75, 1.24)	92749	24836	73	23.5
Exercise frequency: 5-6 times/week	0.75 (0.44, 1.25)	22457	6676	15	18
Exercise frequency: Almost everyday	0.96 (0.71, 1.29)	45038	15135	47	24.9
**Kidney cancer**					
Exercise frequency: None (Reference)	1.00 (1.00, 1.00)	403356	148284	301	16.2
Exercise frequency: 1-2 times/week	1.10 (0.90, 1.34)	220601	62923	153	19.3
Exercise frequency: 3-4 times/week	1.20 (0.93, 1.54)	92749	24836	75	24.1
Exercise frequency: 5-6 times/week	0.98 (0.59, 1.62)	22457	6676	16	19.2
Exercise frequency: Almost everyday	1.17 (0.85, 1.60)	45038	15135	44	23.3
**Gallbladder cancer**					
Exercise frequency: None (Reference)	1.00 (1.00, 1.00)	403356	148284	219	11.8
Exercise frequency: 1-2 times/week	0.97 (0.75, 1.25)	220601	62923	83	10.5
Exercise frequency: 3-4 times/week	1.09 (0.79, 1.52)	92749	24836	43	13.8
Exercise frequency: 5-6 times/week	1.48 (0.89, 2.45)	22457	6676	16	19.2
Exercise frequency: Almost everyday	1.32 (0.94, 1.86)	45038	15135	39	20.6
**Esophagus cancer**					
Exercise frequency: None (Reference)	1.00 (1.00, 1.00)	403356	148284	214	11.5
Exercise frequency: 1-2 times/week	0.91 (0.70, 1.19)	220601	62923	75	9.5
Exercise frequency: 3-4 times/week	0.76 (0.51, 1.12)	92749	24836	29	9.3
Exercise frequency: 5-6 times/week	0.56 (0.25, 1.26)	22457	6676	6	7.2
Exercise frequency: Almost everyday	0.93 (0.62, 1.37)	45038	15135	28	14.8

Supplementary Figure 2. Associations of exercise with incident myocardial infarction, stroke, hypertension and Type 2 diabetes (top panel) and various incident cancer outcomes (bottom panel). Note: Cox regression models using age as the underlying timescale were not adjusted for any confounders. Crude rates are per 100,000 person-years. “N” indicates numbers of total observations (i.e. participants who provided repeated measures are treated as separate observations) and “n” indicates numbers of unique participants at baseline. Abbreviations: HR – Hazard Ratio; CI – Confidence Intervals

BMJ Open

Exercise and incidence of myocardial infarction, stroke, hypertension, type 2 diabetes and site-specific cancers: A prospective cohort study.

Journal:	*BMJ Open*
Manuscript ID	bmjopen-2018-025590.R1
Article Type:	Research
Date Submitted by the Author:	25-Sep-2018
Complete List of Authors:	Kim, Youngwon; University of Utah, Department of Health, Kinesiology, and Recreation; University of Cambridge School of Clinical Medicine, MRC Epidemiology Unit<br>Sharp, Stephen; University of Cambridge, MRC Epidemiology Unit<br>Hwang, Se-mi; Yonsei University, Department of Epidemiology and Health Promotion, Graduate School of Public Health<br>Jee, Sun Ha; Graduate School of Public Health, Yonsei University, Epidemiology
**Primary Subject Heading**:	Epidemiology
Secondary Subject Heading:	Public health
Keywords:	exercise, non-communicable disease, cohort, Epidemiology < TROPICAL MEDICINE, cardiovascular disease, Hypertension < CARDIOLOGY

SCHOLARONE™
Manuscripts

Title: Exercise and incidence of myocardial infarction, stroke, hypertension, type 2 diabetes and site-specific cancers: A prospective cohort study.

Authors: Youngwon Kim,[1,2] Stephen J. Sharp,[2] Se-mi Hwang,[3] Sun Ha Jee[3]

Affiliations:

[1] Department of Health, Kinesiology, and Recreation, College of Health, University of Utah, UT, USA

[2] MRC Epidemiology Unit, University of Cambridge School of Clinical Medicine, Cambridge, UK

[3] Institute for Health Promotion, Department of Epidemiology and Health Promotion, Graduate School of Public Health, Yonsei University, Seoul, Republic of Korea.

Full Addresses:

[1] Department of Health, Kinesiology, and Recreation, College of Health, University of Utah. 250 South 1850 East Room 204, Salt Lake City, UT, USA, 84112

[2] MRC Epidemiology Unit, University of Cambridge School of Clinical Medicine, Box 285 Institute of Metabolic Science, Cambridge Biomedical Campus, Cambridge, Cambridgeshire, UK CB2 0QQ

[3] Department of Epidemiology and Health Promotion, Graduate School of Public Health, Yonsei University, 50-1 Yonsei-ro, Seodaemun-gu, Seoul, Korea 03722

Corresponding Author:

Name: Sun Ha Jee, Ph.D

Mailing Address: Department of Epidemiology and Health Promotion,
Graduate School of Public Health, Yonsei University,
50-1 Yonsei-ro, Seodaemun-gu,
Seoul, Korea 03722

E-mail Information: jsunha@yuhs.ac

Telephone: +82-2-2228-1523

Fax: +82-2-365-5118

Word count for Abstract: 250

Word count for Main text: 2787

For peer review only

Abstract

Objective: The objective of this study was to examine the longitudinal associations of exercise frequency with the incidence of myocardial infarction, stroke, hypertension, type 2 diabetes and 10 different cancer outcomes.

Design: A prospective cohort study.

Setting: Physical examination data linked with the entire South Korean population's health insurance system: from 2002 to 2015

Participants: 257,854 South Korean adults who provided up to 7 repeat-measures of exercise (defined as exercises causing sweat) and confounders.

Primary outcome measures: Each disease incidence was defined using both fatal and non-fatal health records (a median follow-up period of 13 years).

Results: Compared with no exercise category, the middle categories of exercise frequency (3-4 or 5-6 times/week) showed the lowest risk of myocardial infarction (hazard ratio[HR]: 0.79; 95% confidence interval[CI]: 0.70-0.90), stroke (HR: 0.80; 95%CI: 0.73-0.89), hypertension (HR: 0.86; 95%CI: 0.85-0.88), type 2 diabetes (HR: 0.87; 95%CI: 0.84-0.89), stomach (HR: 0.87; 95%CI: 0.79-0.96), lung (HR: 0.80; 95%CI: 0.71-0.91), liver (HR: 0.85; 95%CI: 0.75-0.98) and head & neck cancers (HR: 0.76; 95%CI: 0.63-0.93; for 1-2 times/week), exhibiting J-shaped associations. There was, in general, little evidence of effect modification by body mass index, smoking, alcohol consumption, family history of disease, and sex in these associations.

Conclusions: Moderate levels of sweat-inducing exercise showed the lowest risk of myocardial infarction, stroke, hypertension, type 2 diabetes, stomach, lung, liver and head & neck cancers. Public health and lifestyle interventions should, therefore, promote moderate levels of sweat-causing exercise as a behavioural prevention strategy for non-communicable diseases in a wider population of East Asians.

For peer review only

Keywords: exercise, non-communicable disease, cohort, epidemiology, cardiovascular disease, hypertension, cancer

Article Summary

Strengths and limitations of this study

- This study is the first to investigate the longitudinal associations of exercise with various cardiovascular disease and cancer incidence using a large-scale cohort dataset of South Korean adults (n=257,854) who provided up to 7 repeated measures of exercise and all confounders in order to minimise the risk of regression dilution.
- A limitation is that no strong inference can be drawn about the exercise-incident disease relationships.
- Findings of this study may not be generalizable to adult populations of other ethnic origins.

Introduction

Prevention and control of non-communicable diseases is a contemporary global public health priority. At present, 40 million deaths per year, which accounts for nearly 70% of total deaths globally, are attributable to non-communicable diseases.[1,2] Moreover, the number of deaths due to non-communicable diseases, such as cardiovascular disease,[3] hypertension,[4] diabetes[5] and cancer,[6] has increased dramatically over the past few decades, although age-standardised cardiovascular disease and cancer rates as well as systolic blood pressure levels[7] have declined.[8,9] However, trends in these disease traits have varied across different populations, particularly with less favourable changes observed in East Asian populations compared with Western populations. For example, the prevalence of diabetes[10] has increased more rapidly, while the age-standardised prevalence of cardiovascular disease[3]

For peer review only - http://bmjopen.bmj.com/site/about/guidelines.xhtml

and systolic blood pressure levels[7] have fallen less steeply in East Asians in comparison with Westerners.

In addition, adults in East Asia tend to have higher prevalence of physical inactivity,[11] which is one of the four target behaviours (including unhealthy diet, tobacco use and harmful use of alcohol) that have been set as the global focus to reduce the risk of non-communicable diseases.[12] The beneficial impacts of increased physical activity on various non-communicable outcomes have been demonstrated by numerous previous investigations. However, the majority of previous research has been predicated on evidence from Western populations, thereby limiting its application to other populations including East Asians. As such, little is currently known about levels of physical activity including exercise in relation to non-communicable diseases in East Asian populations as compared with Western populations.[13] Another critical gap in the existing literature is the use of data measured only at a single point in time (i.e., baseline), in which case physical activity or exercise levels are assumed to remain constant over time. This methodology, therefore, precludes the fact that individuals' physical activity or exercise levels change with time, and hence may increase the potential for regression dilution.[14] Furthermore, it is well-known that temporal changes occur in other traditional behavioural and metabolic risk factors for non-communicable diseases, such as adiposity levels,[15,16] smoking,[17] glucose levels[18] and total cholesterol levels,[19] exhibiting different patterns of changes between East Asian and Western populations. Nevertheless, no previous research of East Asians or Westerners took into account changes in these risk markers in understanding the relationships between physical activity and non-communicable diseases. Moreover, the dose-response relationship between physical activity and various non-communicable disease outcomes has remained unclear in East Asians. Therefore, the purpose of this research was to explore the dose-response relationships between exercise frequency and various types of incident non-communicable diseases, such as myocardial infarction, stroke, hypertension, type 2 diabetes and site-specific cancers,

For peer review only

using a large-scale prospective cohort of South Korean adults with multiple repeated measures of exercise frequency and other risk markers.

Methods

Study design and participants

This study is based on data from the National Health Insurance Service - Health Screening (NHIS-HEALS) cohort dataset,[20] which is a nationally representative random sample (stratified by 2 groups of sex [males and females], 18 groups of age ranges [less than 1 year, 1-4 years, every 5 years between 5-79 years, and more than 80 years], 3 groups of employment status [insured employees, self-employed individuals and medical aid beneficiaries] and 41 groups of income levels [upper 20% for insured employees, lower 20% for insured self-employed individuals and the lowest level for medical aid beneficiaries][21]) of over 500,000 South Korean adults aged 40-79 years between 2002 and 2003 made available by the NHIS. The NHIS is a single health insurance system in South Korea, which manages and maintains information on the entire South Korean population's healthcare utilization; it is mandatory for all South Koreans to take part in the national health insurance system. The NHIS is also responsible for maintaining national health examination programs involving data from general health examinations of all insured employees, self-employed individuals and medical aid beneficiaries aged over 40 years; it is recommended for them to perform the health examination at least every two years. The health examination involves collection of information on body composition, blood profiles, blood pressure, self-reported lifestyles, self-reported physician-diagnosed disease, and self-reported family history of disease.

The NHIS-HEALS cohort includes a wide variety of information collected between 2002 and 2015: health examination data and demographic and eligibility data (e.g., in-patient and out-patient hospital records, medical bill, health insurance and medical aid beneficiaries). In the present analysis, we utilised health examination data collected between 2002 and 2008 to define the exercise frequency and all confounders. There was a change in the type of self-

report methods in 2009; hence, health examination data collected in or after 2009 were not considered in the analysis due to the inability to harmonise variables. However, we used full follow-up data accrued from 2002 until 2015. This research was approved by the Institutional Review Board (4-2017-0051) of the Yonsei University's Severance Hospital in Republic of Korea.

Exposure

The primary exposure variable of this study was exercise frequency, assessed using questionnaires administered during the health examinations. The specific question asked was "How many times per week do you engage in exercise that causes sweating?" Participants were asked to choose only one of the following 5 possible answers: None, 1-2 times/week, 3-4 times/week, 5-6 times/week and almost every day.

Outcomes

In the present study, we evaluated 14 incident disease outcomes, namely, myocardial infarction; stroke, hypertension; type 2 diabetes mellitus; and stomach, colon, rectum, lung, liver, head & neck, pancreatic, kidney, gall bladder and esophagus cancers. Participants' in-patient and out-patient hospital records (i.e., non-fatal status) and death records (i.e., fatal status) obtained through linkage with Statistics Korea were both classified according to the International Classification of Disease (ICD)-10 codes to classify different incidence types (Supplementary Table 1). Additionally, blood pressure (e.g., systolic ≥140 mm Hg, diastolic ≥90 mm Hg) and fasting glucose levels (e.g., ≥126 mg/dL), both of which were measured during physical examinations, were used in conjunction with physicians' diagnosis information and ICD-10 codes to define incident hypertension and type 2 diabetes, respectively. Each incident disease outcome was defined as the first occurrence of either non-fatal or fatal respective disease cases. Incident disease cases were adjudicated using hospital and death records collected through December 31st, 2015. The median follow-up was 13.0 years (interquartile range: 10.2-11.3 years)

For peer review only

Other covariates

The following covariates were included as confounders in the analyses: sex, body mass index (weight in kilograms (kg) divided by height in meters squared (m^2)), systolic blood pressure, fasting glucose, total cholesterol, family history of heart disease, stroke or hypertension [only in models for incident myocardial infarction, stroke or hypertension], family history of diabetes [only in models for type 2 diabetes], family history of cancer [only in models for incident cancer outcomes], smoking status (never, previously, currently) and alcohol consumption (never, 2-3 times/month, 1-2 times/week, ≥3 times/week).

Statistical analysis

Analyses were performed to summarise descriptive statistics (e.g., means, standard deviations, frequency, and proportions) of each covariate and incident disease outcome for all participants and by exercise frequency category. Cox regression with age as the underlying time scale was used to estimate the associations of exercise frequency with each incident disease outcome, with adjustment for all the above-mentioned confounders as well as without any adjustment. Hazard ratios along with corresponding 95% confidence intervals were calculated to evaluate relative risk of each incident disease outcome. Data were structured to enable the inclusion of exercise frequency and all confounders from both baseline and up to 6 repeated measures as time-updated covariates. This approach takes into account changes in exercise frequency as well as each confounder over time in relation to disease incidence. Individuals who reported no exercise served as a reference group for all comparisons. Effect modification by body mass index (<25, ≥25kg^2/m), smoking status, alcohol consumption, family history of disease and sex was also examined based on Wald tests of interaction terms in the fully adjusted models for each incident disease outcome. Visual inspections of log-log plots provided support for the assumptions of proportional hazards for all covariates. A sensitivity analysis where incident disease cases occurring during the first 2 years of follow-up were removed was performed to address reverse

For peer review only

causality. Analyses were performed in Stata/SE Version 14 (StataCorp LP, College Station, TX).

Patient and Public Involvement.

Neither patients nor members of the public were involved in this study.

Results

Of an initial sample of 512,190 individuals, 74,931 had missing data on at least one of the model covariates, and 179,405 had self-reported physician-diagnosed heart attack, stroke, hypertension (additionally, systolic ≥140 mm Hg or diastolic ≥90 mm Hg), diabetes (additionally, fasting glucose levels ≥126 mg/dL) or cancer at baseline, respectively. Excluding these individuals resulted in a final sample for analysis of 257,854 individuals (Figure 1).

Individuals provided up to 7 measures of exercise frequency and each confounder (i.e., baseline plus 6 repeated measures). Participants' characteristics at baseline are summarised in Table 1. Supplementary Table 2 summarises participants' characteristics at each repeat assessment. Individuals in the categories of 1-2, 3-4 or 5-6 times/week of exercise were slightly younger, but showed higher proportions of family history of disease and lower proportions of never smoking or drinking alcohol, compared with those in the categories of none or almost every day of exercise. Across the seven time points (Supplementary Figure 1), the proportion of individuals who reported no exercise decreased while the proportion who reported 1-2 or 3-4 times/week of exercise increased; there were no noticeable changes for the categories of 5-6 times/week or almost every day of exercise.

Overall, J-shaped associations were found between exercise frequency and incident myocardial infarction, stroke, hypertension and type 2 diabetes. Hazard ratios for these diseases were lowest in the middle categories of exercise frequency (e.g., 3-4 or 5-6 times/week) (Figure 2). There were no associations for the most frequent exercise category

For peer review only

(e.g., almost every day) with the incidence of myocardial infarction, stroke and type 2 diabetes.

J-shaped associations were also found for incident stomach, lung, liver and head & neck cancers (Figure 3). Higher exercise frequencies (e.g., 1-2, 3-4 times/week and almost every day) were associated with lower hazards of incident stomach cancer. No statistical significance was observed for incident colon, rectum, pancreas, kidney, gallbladder and esophagus cancers. Crude event rates per 100,000 person-years in the middle categories of exercise frequency were relatively lower for incident rectum, and esophagus cancers, but higher for incident pancreas, kidney and gallbladder cancers. Cox regression models with no adjustment for confounders (Supplementary Figure 2) and a sensitivity analysis (Supplementary Figure 3) in which incident cases occurring in the first 2 years of follow-up were removed both revealed nearly identical patterns of associations as the main analyses.

Figure 4 shows comparisons of results that showed statistical significance for multiplicative interaction terms between exercise frequency and each incident disease outcome. Strong J-shaped associations for incident hypertension were identified at each level of body mass index. J-shaped associations of exercise frequency with incident hypertension were strong only in the more favourable levels of smoking (e.g., never, previously) and alcohol consumption (e.g., never, 2-3 times/month, 1-2 times/week); no or weak associations were identified in the most harmful level of smoking (e.g., current smokers) and alcohol consumption (e.g., ≥3 times/week). J-shaped associations were evident at all levels of family history of CVD and sex for incident hypertension, and sex for incident type 2 diabetes. Exercise frequency was associated with incident lung cancer in non-obese individuals, but there was no evidence of association in obese individuals. All comparisons stratified by each potential effect modifier are presented in Supplementary Figures 4 and 5.

Discussion

For peer review only

This is the first investigation examining the prospective associations of exercise with various incident non-communicable disease outcomes using multiple repeated measures of covariates in East Asian populations. We identified J-shaped associations of sweat-inducing exercise with incident myocardial infarction, stroke, hypertension, type 2 diabetes, stomach, lung, liver and head & neck cancers, with the greatest benefits being observed in the middle categories of exercise frequency (e.g., 3-4 or 5-6 times/week): 1-2 times/week for head & neck cancer. These findings provide two important clinical and public health implications. First, prevention and management of non-communicable diseases in East Asians may benefit considerably from employing an exercise promotion approach in the context of combined non-communicable disease prevention. Mechanism research indicates that cardiovascular disease and type 2 diabetes have similar biological pathways relating to exercise,[22,23] so an integrated prevention approach can be applied to control and manage these two diseases at a minimum.[5] Moreover, regular participation in exercise can induce favourable changes in intermediate cardiometabolic risk markers,[24] which are important predictors of typical non-communicable diseases. Hence, promoting exercise has great potential to act as an integrative behavioural strategy for preventing and controlling various non-communicable diseases simultaneously in East Asian populations.

Second, individuals who engage in exercise 3-4 or 5-6 times/week, rather than every day, may be able to reduce their risk of developing myocardial infarction, stroke, hypertension, type 2 diabetes, stomach, lung and liver cancers: 1-2 times/week for head & neck cancer. Similar J-shaped associations between high intensity exercise (e.g., running) and cardiovascular disease risk have also been reported in previous cohort studies of Western[25,26] and Japanese adults.[27] Nevertheless, the present study as well as previous research[25-27] found that the risk of developing cardiovascular events in individuals who had the highest level of exercise was not noticeably higher compared with those who had the lowest level of exercise. No previous research in East Asians has found such J-shaped relationships between exercise or physical activity and other incident disease outcomes such as hypertension,[28-32] diabetes[33-40] and different type of cancers.[41-47] However, previous meta-

For peer review only

analyses of cohort studies comprising predominantly Westerners found leisure-time physical activity to have curvilinear (but not J-shaped) associations with the incidence of type 2 diabetes,[48] and linear associations with the incidence of hypertension[49] and various site-specific cancers (liver, lung, head & neck, kidney, colon, rectal, bladder, gastric cardia, breast, endometrial, myeloid leukemia, myeloma, esophageal adenocarcinoma).[50] While additional research is needed to confirm the J-shaped associations of exercise with various incident diseases in other samples of East Asians, findings of this research provide a strong rationale for development and implementation of public health policies and clinical trials aimed at promoting a moderate level of sweat-causing exercise to minimise the risk of myocardial infarction, stroke, hypertension, type 2 diabetes, stomach, lung, liver and head & neck cancers.

Another finding of this research is that associations of sweat-inducing exercise with hypertension were modified by body mass index, smoking, alcohol consumption, family history of cardiovascular disease and sex: lung cancer by body mass index and type 2 diabetes by sex. Notably, exercise frequency was not associated with hypertension in individuals who are smokers or drinking alcohol ≥3 times/week (except for 3-4 times/week of exercise). This observation provides some evidence that the harmful impacts of smoking or binge drinking on hypertension[51-53] may not be offset completely by exercise. This, in turn, appears to advocate for the need for implementing a combined hypertension prevention strategy targeting promotion of exercise in conjunction with smoking cessation and reductions in alcohol consumption in East Asians.[13] For lung cancer, the null associations in individuals with body mass index ≥25 may be indicative of potential residual confounding through reported bias in smoking behaviours. Nonetheless, there was little evidence for effect modification for other disease comparisons, highlighting the importance of promoting exercise for the prevention of various non-communicable diseases in individuals at different levels of body mass index, smoking, alcohol consumption, family history of disease and sex.

For peer review only

This study has several notable strengths. First, we used data from a large prospective cohort study in which exercise and other risk markers were assessed on multiple occasions (up to 7 times). Nearly 84% and 5% of the full participants provided 1 and 6 repeated measures of all covariates, respectively. Compelling evidence indicates that the risk of regression dilution can be reduced using repeated measures of exposure and confounders.[14] Moreover, we examined the dose-response relationship of exercise frequency with a wide variety of specific types of incident non-communicable disease outcomes simultaneously using in-patient and out-patient diagnosis data as well as mortality data. The large sample size (n=257,854) is another strength.

This study has some limitations. Findings of this study may not be generalizable to adult populations of other ethnic origins. Due to the observational nature of this research, no strong inference can be drawn about the exercise-incident disease relationships. In addition, the accuracy of hospital admission records is uncertain, although the accuracy of death records from Statistics Korea was found to be 92% in previous research.[54] No information about medication use was available in the cohort data, so we could not use it as a potential confounder and another condition when defining disease status (e.g., hypertension, type 2 diabetes) at both baseline and follow-up. Furthermore, no exercise duration was assessed; hence, inference was made purely based on exercise frequency. Moreover, ICD-10 codes for sex-specific cancers (e.g., prostate and breast cancers) were masked due to the data management policy set forth by the NHIS, so it was not possible to examine such cancers in the present study. The lack of data on diet, which is another behavioural risk marker for non-communicable diseases[12] is another limitation. Moreover, a sizeable proportion (n=74,931; 14.6%) of individuals were excluded due to the missing information on the covariates. Another limitation is that the measurement methods to assess the covariates were not standardized across the different medical institutes participating in the NHIS-HEALS cohort.

Conclusion

For peer review only

Individuals who engaged in sweat-inducing exercise around 3-6 times/week (as opposed to every day) generally had the lowest risk of developing myocardial infarction, stroke, hypertension, type 2 diabetes, stomach, lung, liver and head & neck cancers. These findings were generally applicable to different sub-populations as stratified by body mass index, smoking, alcohol consumption, family history of disease, and sex. Public health and lifestyle interventions should promote a moderate level of sweat-inducing exercise as a behavioural strategy for prevention and control of non-communicable diseases in a wider population of East Asians.

Author Contributions

YK designed this study, performed statistical analysis, and drafted an initial version of the manuscript. SJS, SMH and SHJ all contributed to conceptualizing the study idea and developing the analytical plans, and provided assistance with statistical analysis. All authors critically reviewed, approved of the final version of the manuscript, and agreed to be responsible for all facets of this work.

Acknowledgements

None.

Funding

This work was supported by the National R&D Program for Cancer Control, Ministry of Health & Welfare, Republic of Korea (Grant 1631020 to S.H.J.), The Korean Health Technology R&D Project, Ministry of Health & Welfare, Republic of Korea (Grant HI14C2686 to S.H.J.), and the Medical Research Council (Grant MC_UU_12015/1 to S.J.S.). The funders had no role in study design, data collection and analysis, decision to publish, or preparation of the manuscript.

Conflicts of interest

None declared.

For peer review only

Patient consent

Not required.

Ethics approval

This research was approved by the Institutional Review Board (4-2017-0051) of the Yonsei University's Severance Hospital in Republic of Korea.

Data sharing statement

Data sharing is not applicable because no informed consent for data sharing was obtained from the participants.

For peer review only

References

1 Mortality, G. B. D. & Causes of Death, C. Global, regional, and national life expectancy, all-cause mortality, and cause-specific mortality for 249 causes of death, 1980-2015: a systematic analysis for the Global Burden of Disease Study 2015. *Lancet* **388**, 1459-1544, doi:10.1016/S0140-6736(16)31012-1 (2016).

2 Lakerveld, J., Bot, S. D., van der Ploeg, H. P. & Nijpels, G. The effects of a lifestyle intervention on leisure-time sedentary behaviors in adults at risk: The Hoorn Prevention Study, a randomized controlled trial. *Prev Med* **57**, 351-356, doi:10.1016/j.ypmed.2013.06.011 (2013).

3 Roth, G. A. *et al.* Global, Regional, and National Burden of Cardiovascular Diseases for 10 Causes, 1990 to 2015. *J Am Coll Cardiol* **70**, 1-25, doi:10.1016/j.jacc.2017.04.052 (2017).

4 Forouzanfar, M. H. *et al.* Global Burden of Hypertension and Systolic Blood Pressure of at Least 110 to 115 mm Hg, 1990-2015. *Jama* **317**, 165-182, doi:10.1001/jama.2016.19043 (2017).

5 *World Health Organization Global report on diabetes*. (World Health Organization, 2016).

6 Global Burden of Disease Cancer, C. *et al.* Global, Regional, and National Cancer Incidence, Mortality, Years of Life Lost, Years Lived With Disability, and Disability-Adjusted Life-years for 32 Cancer Groups, 1990 to 2015: A Systematic Analysis for the Global Burden of Disease Study. *JAMA Oncol* **3**, 524-548, doi:10.1001/jamaoncol.2016.5688 (2017).

7 Danaei, G. *et al.* National, regional, and global trends in systolic blood pressure since 1980: systematic analysis of health examination surveys and epidemiological studies with 786 country-years and 5.4 million participants. *Lancet* **377**, 568-577, doi:10.1016/S0140-6736(10)62036-3 (2011).

8 Roth, G. A. *et al.* Global and regional patterns in cardiovascular mortality from 1990 to 2013. *Circulation* **132**, 1667-1678, doi:10.1161/CIRCULATIONAHA.114.008720 (2015).

9 Death, G. C. Global, regional, and national age-sex specific mortality for 264 causes of death, 1980-2016: a systematic analysis for the Global Burden of Disease Study 2016 (vol 390, pg 1151, 2017). *Lancet* **390**, E38-E38 (2017).

10 NCD-, N. R. F. C. NCD Risk Factor Collaboration (NCD-RisC). Worldwide trends in diabetes since 1980: a pooled analysis of 751 population-based studies with 4.4 million participants (vol 387, pg 1513, 2016). *Lancet* **389**, E2-E2, doi:10.1016/S0140-6736(16)32060-8 (2017).

11 Ku, P. W., Fox, K. R., McKenna, J. & Peng, T. L. Prevalence of leisure-time physical activity in Taiwanese adults: results of four national surveys, 2000-2004. *Preventive medicine* **43**, 454-457, doi:10.1016/j.ypmed.2006.04.011 (2006).

12 World Health Organization. Global status report on noncommunicable diseases 2010. Geneva, World Health Organization, 2011.

13 Kokubo, Y. Prevention of hypertension and cardiovascular diseases: a comparison of lifestyle factors in Westerners and East Asians. *Hypertension* **63**, 655-660, doi:10.1161/HYPERTENSIONAHA.113.00543 (2014).

14 Hutcheon, J. A., Chiolero, A. & Hanley, J. A. Random measurement error and regression dilution bias. *Bmj* **340**, c2289, doi:10.1136/bmj.c2289 (2010).

15 Ng, M. *et al.* Global, regional, and national prevalence of overweight and obesity in children and adults during 1980-2013: a systematic analysis for the Global Burden of Disease Study 2013. *Lancet* **384**, 766-781, doi:10.1016/S0140-6736(14)60460-8 (2014).

16 Lear, S. A. *et al.* Visceral adipose tissue accumulation differs according to ethnic background: results of the Multicultural Community Health Assessment Trial (M-CHAT). *Am J Clin Nutr* **86**, 353-359 (2007).

17 (ed Geneva: World Health Organization) (2015).

18 Chan, J. C. *et al.* Diabetes in Asia: epidemiology, risk factors, and pathophysiology. *Jama* **301**, 2129-2140, doi:10.1001/jama.2009.726 (2009).

For peer review only

19 Nguyen, H. N., Fujiyoshi, A., Abbott, R. D. & Miura, K. Epidemiology of cardiovascular risk factors in Asian countries. *Circulation journal : official journal of the Japanese Circulation Society* **77**, 2851-2859 (2013).
20 Seong, S. C. *et al.* Cohort profile: the National Health Insurance Service-National Health Screening Cohort (NHIS-HEALS) in Korea. *BMJ open* **7**, e016640, doi:10.1136/bmjopen-2017-016640 (2017).
21 Lee, J., Lee, J. S., Park, S. H., Shin, S. A. & Kim, K. Cohort Profile: The National Health Insurance Service-National Sample Cohort (NHIS-NSC), South Korea. *Int J Epidemiol* **46**, e15, doi:10.1093/ije/dyv319 (2017).
22 Pedersen, B. K. Anti-inflammatory effects of exercise: role in diabetes and cardiovascular disease. *Eur J Clin Invest* **47**, 600-611, doi:10.1111/eci.12781 (2017).
23 Stern, M. P. Diabetes and cardiovascular disease. The "common soil" hypothesis. *Diabetes* **44**, 369-374 (1995).
24 *Global Recommendations on Physical Activity for Health*. (2010).
25 Lee, D. C. *et al.* Leisure-Time Running Reduces All-Cause and Cardiovascular Mortality Risk. *J Am Coll Cardiol* **64**, 472-481, doi:10.1016/j.jacc.2014.04.058 (2014).
26 Tanasescu, M. *et al.* Exercise type and intensity in relation to coronary heart disease in men. *Jama* **288**, 1994-2000 (2002).
27 Kubota, Y. *et al.* Daily Total Physical Activity and Incident Stroke: The Japan Public Health Center-Based Prospective Study. *Stroke* **48**, 1730-1736, doi:10.1161/STROKEAHA.117.017560 (2017).
28 Lu, Y. *et al.* Lifestyle and Risk of Hypertension: Follow-Up of a Young Pre-Hypertensive Cohort. *Int J Med Sci* **12**, 605-612, doi:10.7150/ijms.12446 (2015).
29 Sun, Z. *et al.* Incidence and predictors of hypertension among rural Chinese adults: results from Liaoning province. *Ann Fam Med* **8**, 19-24, doi:10.1370/afm.1018 (2010).
30 Gu, D. *et al.* Incidence and predictors of hypertension over 8 years among Chinese men and women. *J Hypertens* **25**, 517-523, doi:10.1097/HJH.0b013e328013e7f4 (2007).
31 Lee, S. *et al.* Cardiorespiratory fitness attenuates metabolic risk independent of abdominal subcutaneous and visceral fat in men. *Diabetes Care* **28**, 895-901, doi:DOI 10.2337/diacare.28.4.895 (2005).
32 Nakanishi, N. & Suzuki, K. Daily life activity and the risk of developing hypertension in middle-aged Japanese men. *Archives of internal medicine* **165**, 214-220, doi:10.1001/archinte.165.2.214 (2005).
33 Fan, S. *et al.* Physical Activity Level and Incident Type 2 Diabetes among Chinese Adults. *Med Sci Sport Exer* **47**, 751-756, doi:10.1249/Mss.0000000000000471 (2015).
34 Joseph, J. J. *et al.* Physical activity, sedentary behaviors and the incidence of type 2 diabetes mellitus: the Multi-Ethnic Study of Atherosclerosis (MESA). *Bmj Open Diab Res Ca* **4**, doi:ARTN e00018510.1136/bmjdrc-2015-000185 (2016).

35 Xu, F. *et al.* Joint Associations of Physical Activity and Hypertension with the Development of Type 2 Diabetes among Urban Men and Women in Mainland China. *Plos One* **9**, doi:ARTN e8871910.1371/journal.pone.0088719 (2014).

36 Xu, F. *et al.* Joint impact of physical activity and family history on the development of diabetes among urban adults in Mainland China: a pooled analysis of community-based prospective cohort studies. *Asia Pac J Public Health* **27**, NP372-381, doi:10.1177/1010539512443700 (2015).
37 Shi, L. *et al.* Physical activity, smoking, and alcohol consumption in association with incidence of type 2 diabetes among middle-aged and elderly Chinese men. *Plos One* **8**, e77919, doi:10.1371/journal.pone.0077919 (2013).
38 Villegas, R. *et al.* Physical activity and the incidence of type 2 diabetes in the Shanghai women's health study. *Int J Epidemiol* **35**, 1553-1562, doi:10.1093/ije/dyl209 (2006).

For peer review only

39 Lee, D. C. *et al.* Physical Activity and Body Mass Index and Their Associations With the Development of Type 2 Diabetes in Korean Men. *Am J Epidemiol* **176**, 43-51, doi:10.1093/aje/kwr471 (2012).
40 Honda, T. *et al.* Leisure-time, occupational, and commuting physical activity and risk of type 2 diabetes in Japanese workers: a cohort study. *Bmc Public Health* **15**, doi:ARTN 100410.1186/s12889-015-2362-5 (2015).
41 Inoue, M. *et al.* Daily total physical activity level and total cancer risk in men and women: results from a large-scale population-based cohort study in Japan. *Am J Epidemiol* **168**, 391-403, doi:10.1093/aje/kwn146 (2008).
42 Lee, K. J. *et al.* Physical activity and risk of colorectal cancer in Japanese men and women: the Japan Public Health Center-based prospective study. *Cancer causes & control : CCC* **18**, 199-209, doi:10.1007/s10552-006-0098-3 (2007).
43 Luo, J. *et al.* Body mass index, physical activity and the risk of pancreatic cancer in relation to smoking status and history of diabetes: a large-scale population-based cohort study in Japan--the JPHC study. *Cancer causes & control : CCC* **18**, 603-612, doi:10.1007/s10552-007-9002-z (2007).
44 Suzuki, S. *et al.* Effect of physical activity on breast cancer risk: findings of the Japan collaborative cohort study. *Cancer epidemiology, biomarkers & prevention : a publication of the American Association for Cancer Research, cosponsored by the American Society of Preventive Oncology* **17**, 3396-3401, doi:10.1158/1055-9965.EPI-08-0497 (2008).
45 Eaglehouse, Y. L. *et al.* Physical activity, sedentary time, and risk of colorectal cancer: the Singapore Chinese Health Study. *Eur J Cancer Prev* **26**, 469-475, doi:10.1097/CEJ.0000000000000369 (2017).
46 Mok, Y., Jeon, C., Lee, G. J. & Jee, S. H. Physical Activity Level and Colorectal Cancer Mortality. *Asia Pac J Public Health* **28**, 638-647, doi:10.1177/1010539516661761 (2016).
47 Yun, Y. H. *et al.* Dietary preference, physical activity, and cancer risk in men: national health insurance corporation study. *Bmc Cancer* **8**, 366, doi:10.1186/1471-2407-8-366 (2008).
48 Smith, A. D., Crippa, A., Woodcock, J. & Brage, S. Physical activity and incident type 2 diabetes mellitus: a systematic review and dose-response meta-analysis of prospective cohort studies. *Diabetologia* **59**, 2527-2545, doi:10.1007/s00125-016-4079-0 (2016).
49 Liu, X. J. *et al.* Dose-Response Association Between Physical Activity and Incident Hypertension A Systematic Review and Meta-Analysis of Cohort Studies. *Hypertension* **69**, 813-+, doi:10.1161/Hypertensionaha.116.08994 (2017).
50 Moore, S. C. *et al.* Association of Leisure-Time Physical Activity With Risk of 26 Types of Cancer in 1.44 Million Adults. *JAMA internal medicine* **176**, 816-825, doi:10.1001/jamainternmed.2016.1548 (2016).
51 Stranges, S. *et al.* Relationship of alcohol drinking pattern to risk of hypertension: a population-based study. *Hypertension* **44**, 813-819, doi:10.1161/01.HYP.0000146537.03103.f2 (2004).
52 Niskanen, L. *et al.* Inflammation, abdominal obesity, and smoking as predictors of hypertension. *Hypertension* **44**, 859-865, doi:10.1161/01.HYP.0000146691.51307.84 (2004).
53 Halperin, R. O., Gaziano, J. M. & Sesso, H. D. Smoking and the risk of incident hypertension in middle-aged and older men. *Am J Hypertens* **21**, 148-152, doi:10.1038/ajh.2007.36 (2008).
54 Won, T., Kang, B., Im, T. & Choi, H. The study of accuracy of death statistics. *J Korean Soc Emerg Med* **18**, 256–262 (2007).

Figure Legends

Figure 1. A flow diagram. Note: "N" indicates numbers of total observations (i.e. participants who provided repeated measures are treated as separate observations) and "n" indicates numbers of unique participants at baseline. Data without missingness and prevalence of major diseases were used to create final analysis datasets for different incident disease

For peer review only

outcomes; while the number of unique participants at baseline is the same for all incident disease outcomes, the total number of observations varied due to the nature of time-updated covariate analyses (i.e. censoring of subsequent time-updated covariates when an incident disease case occurs before the end date of repeated measures). Data were obtained from a prospective cohort, which has been established by linking physical examination data of over half a million South Korean adults (2002-2015) with the entire South Korean population's health insurance system.

Figure 2. Associations of exercise frequency with various incident cardiovascular disease outcomes. Cox regression models with age as the underlying timescale were adjusted for sex, body mass index, systolic blood pressure, fasting glucose levels, total cholesterol levels, family history of heart disease, stroke or hypertension (in models for myocardial infarction, stroke and hypertension) or diabetes (in models for Type 2 diabetes), smoking status and alcohol consumption. Crude rates are per 100,000 person-years. "N" indicates numbers of total observations (i.e. participants who provided repeated measures are treated as separate observations) and "n" indicates numbers of unique participants at baseline. Data were obtained from a prospective cohort, which has been established by linking physical examination data of over half a million Korean adults (2002-2015) with the entire South Korean population's health insurance system. Abbreviations: HR – Hazard Ratio; CI – Confidence Intervals

Figure 3. Associations of exercise frequency with various incident cancer outcomes. Cox regression models with age as the underlying timescale were adjusted for sex, body mass index, systolic blood pressure, fasting glucose levels, total cholesterol levels, family history of cancer, smoking status and alcohol consumption. Crude rates are per 100,000 person-years. "N" indicates numbers of total observations (i.e. participants who provided repeated measures are treated as separate observations) and "n" indicates numbers of unique participants at baseline. Data were obtained from a prospective cohort, which has been established by linking physical examination data of over half a million South Korean adults (2002-2015) with the entire South Korean population's health insurance system. Abbreviations: HR – Hazard Ratio; CI – Confidence Intervals

Figure 4. Results from assessment of effect modification of sex, body mass index, smoking, alcohol consumption, and family history of disease in the associations between exercise frequency and each incident disease outcome. Only associations for which the multiplicative interaction terms were statistically significant are presented. Cox regression models with age as the underlying timescale were adjusted for sex [not in models for effect modification by sex], body mass index [not in models for effect modification by body mass index], systolic blood pressure, fasting glucose levels, total cholesterol levels, family history [not in models for effect modification by family history of respective disease] of heart disease/stroke/hypertension (in models for myocardial infarction, stroke and hypertension), diabetes (in models for Type 2 diabetes) or cancer (in models for each cancer), smoking status [not in models for effect modification by smoking status] and alcohol consumption [not in models for effect modification by alcohol consumption]. Crude rates are per 100,000 person-years. "N" indicates numbers of total observations (i.e. participants who provided repeated measures are treated as separate observations) and "n" indicates numbers of unique participants at baseline. P-values for multiplicative interactions with exercise frequency are as follows; Outcome – hypertension: body mass index (0.049), smoking (<0.001), alcohol consumption (<0.001), family history of cardiovascular disease (0.029) and sex (<0.001); Outcome - lung cancer: body mass index (0.016), and; Outcome - type 2 diabetes: sex (0.012). Data were obtained from a prospective cohort, which has been established by linking physical examination data of over half a million South Korean adults

For peer review only - http://bmjopen.bmj.com/site/about/guidelines.xhtml

(2002-2015) with the entire South Korean population's health insurance system. Abbreviations: HR – Hazard Ratio; CI – Confidence Intervals

For peer review only

Table 1. Characteristics of the participants at baseline.

Variables	All (n=257,854)	Exercise frequency				
		None (n=148,284)	1-2 times/week (n=62,923)	3-4 times/week (n=24,836)	5-6 times/week (n=6,676)	Almost everyday (n=15,135)
Sex, %						
Men	50.5%	43.8%	63.3%	57.5%	52.9%	49.6%
Women	49.5%	56.2%	36.7%	42.5%	47.1%	50.4%
Age, years	50.7 (8.7)	51.5 (9.2)	49.0 (7.5)	49.3 (7.5)	49.9 (7.8)	53.5 (9.3)
Body Mass Index, kg^2/m	23.5 (2.8)	23.3 (2.9)	23.6 (2.7)	23.7 (2.6)	23.7 (2.6)	23.7 (2.7)
Systolic blood pressure, mm Hg	116.8 (11.2)	116.6 (11.3)	117.1 (11.0)	116.8 (11.1)	116.8 (11.2)	117.3 (11.3)
Diastolic blood pressure, mm Hg	73.4 (7.9)	73.1 (8.0)	73.8 (7.8)	73.5 (7.9)	73.4 (8.0)	73.4 (7.9)
Fasting glucose levels, mg/dL	90.2 (12.3)	90.1 (12.4)	90.4 (12.2)	89.9 (11.9)	90.2 (12.3)	90.2 (12.4)
Total cholesterol, mg/dL	197.0 (36.7)	196.5 (37.1)	197.7 (36.2)	197.8 (35.9)	197.8 (36.1)	197.4 (36.8)
Family history of heart disease, stroke or hypertension, %	12.2%	10.8%	13.9%	15.9%	16.5%	10.9%
Family history of cancer, %	14.2%	13.1%	15.3%	17.2%	16.6%	14.5%
Family history of diabetes, %	6.3%	5.4%	7.2%	8.5%	9.2%	5.7%
Smoking status, %						
Never	68.1%	71.9%	59.1%	65.2%	68.8%	71.7%
Previously	8.4%	5.8%	12.0%	12.7%	12.5%	8.8%
Currently	23.6%	22.2%	28.9%	22.1%	18.8%	19.5%
Alcohol Consumption, %						
Never	58.4%	64.5%	47.6%	50.5%	52.2%	59.3%
2-3 times/month	16.4%	13.8%	21.3%	19.7%	18.8%	14.1%
1-2 times/week	15.8%	12.4%	22.0%	20.2%	18.1%	14.5%
≥3 times/week	9.5%	9.3%	9.1%	9.5%	10.8%	12.1%
Incident myocardial infarction, n (%)	3,047 (1.2)	1,741 (1.2)	723 (1.1)	276 (1.1)	88 (1.3)	219 (1.4)
Incident stroke, n (%)	16,134 (6.3)	9,689 (6.5)	3,333 (5.3)	1,482 (6.0)	390 (5.8)	1,240 (8.2)
Incident hypertension, n (%)	120,203 (46.6)	65,964 (44.5)	30,623 (48.7)	12,617 (50.8)	3,294 (49.3)	7,705 (50.9)
Incident Type 2 diabetes, n (%)	50,459 (19.6)	27,128 (18.3)	10,666 (6.5)	5,421 (21.8)	1,399 (21.0)	3,285 (21.7)
Incident stomach cancer, n (%)	4,788 (1.9)	2,672 (1.8)	13,226 (21.0)	489 (2.0)	139 (2.1)	328 (2.2)
Incident colon cancer, n (%)	2,711 (1.1)	1,424 (1.0)	1,160 (1.8)	314 (1.3)	90 (1.3)	191 (1.3)
Incident rectum cancer, n (%)	1,494 (0.6)	809 (0.6)	692 (1.1)	154 (0.6)	46 (0.6)	107 (0.7)
Incident lung cancer, n (%)	3,601 (1.4)	2,138 (1.4)	796 (1.3)	307 (1.2)	85 (1.3)	275 (1.8)
Incident liver cancer, n (%)	2,620 (1.0)	1,423 (1.0)	680 (1.1)	263 (1.1)	75 (1.1)	179 (1.2)
Incident pancreas cancer, n (%)	864 (0.3)	483 (0.3)	205 (0.3)	92 (0.4)	24 (0.4)	60 (0.4)
Incident head & neck cancer, n (%)	656 (0.3)	377 (0.3)	144 (0.2)	73 (0.3)	15 (0.2)	47 (0.3)
Incident kidney cancer, n (%)	589 (0.2)	301 (0.2)	153 (0.2)	75 (0.3)	16 (0.2)	44 (0.3)
Incident gallbladder cancer, n (%)	400 (0.2)	219 (0.1)	83 (0.1)	43 (0.2)	16 (0.2)	39 (0.3)
Incident esophagus cancer, n (%)	352 (0.1)	214 (0.1)	75 (0.1)	29 (0.1)	6 (0.09)	28 (0.2)
Median follow-up period, years (interquartile range)	13.0 (12.2, 13.3)	13.0 (12.2, 13.3)	13.0 (12.2, 13.3)	13.0 (12.2, 13.3)	12.7 (12.2, 13.3)	12.6 (12.2, 13.3)

For peer review only

Note: Values presented are means unless indicated as an 'n'. Values in parentheses are standard deviations unless otherwise indicated. Data were obtained from a prospective cohort, which has been established by linking physical examination data of over half a million South Korean adults (2002-2015) with the entire South Korean population's health insurance system.

For peer review only

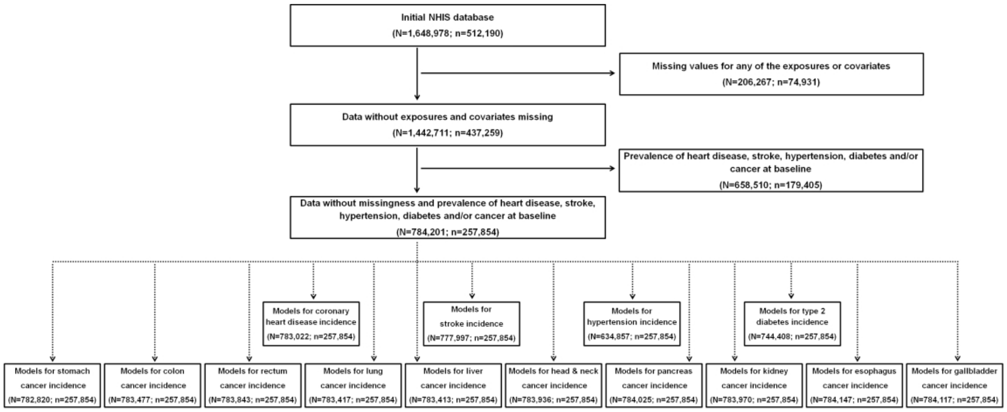

Figure 1. A flow diagram. Note: “N” indicates numbers of total observations (i.e. participants who provided repeated measures are treated as separate observations) and “n” indicates numbers of unique participants at baseline. Data without missingness and prevalence of major diseases were used to create final analysis datasets for different incident disease outcomes; while the number of unique participants at baseline is the same for all incident disease outcomes, the total number of observations varied due to the nature of time-updated covariate analyses (i.e. censoring of subsequent time-updated covariates when an incident disease case occurs before the end date of repeated measures). Data were obtained from a prospective cohort, which has been established by linking physical examination data of over half a million South Korean adults (2002-2015) with the entire South Korean population’s health insurance system.

1412x571mm (72 x 72 DPI)

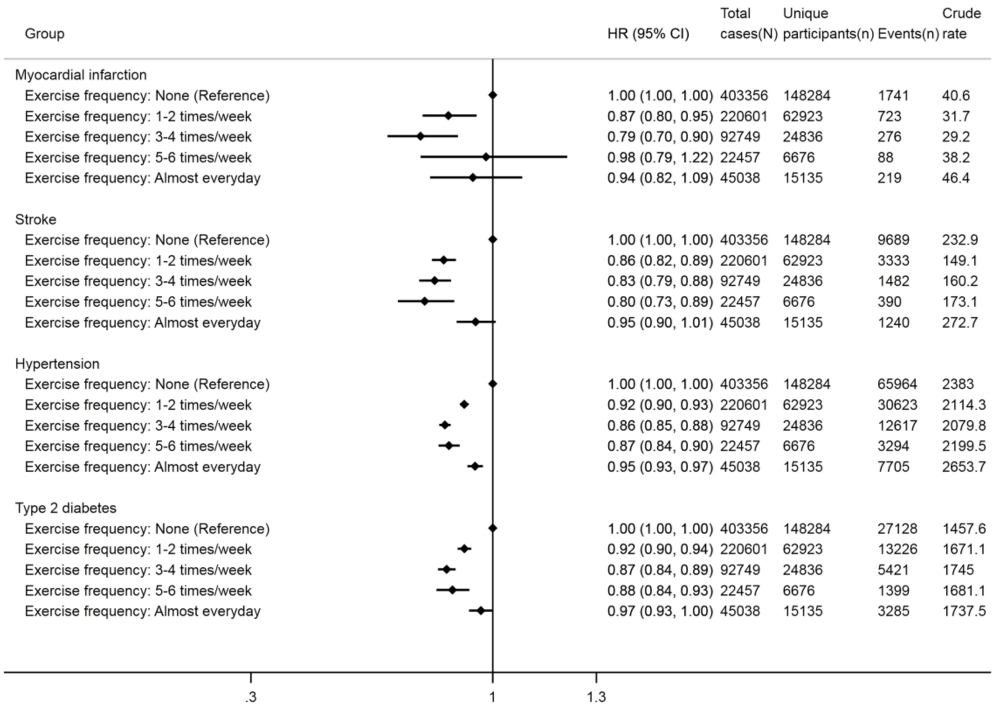

Figure 2. Associations of exercise frequency with various incident cardiovascular disease outcomes. Cox regression models with age as the underlying timescale were adjusted for sex, body mass index, systolic blood pressure, fasting glucose levels, total cholesterol levels, family history of heart disease, stroke or hypertension (in models for myocardial infarction, stroke and hypertension) or diabetes (in models for Type 2 diabetes), smoking status and alcohol consumption. Crude rates are per 100,000 person-years. “N” indicates numbers of total observations (i.e. participants who provided repeated measures are treated as separate observations) and “n” indicates numbers of unique participants at baseline. Data were obtained from a prospective cohort, which has been established by linking physical examination data of over half a million Korean adults (2002-2015) with the entire South Korean population’s health insurance system.
Abbreviations: HR – Hazard Ratio; CI – Confidence Intervals

209x159mm (300 x 300 DPI)

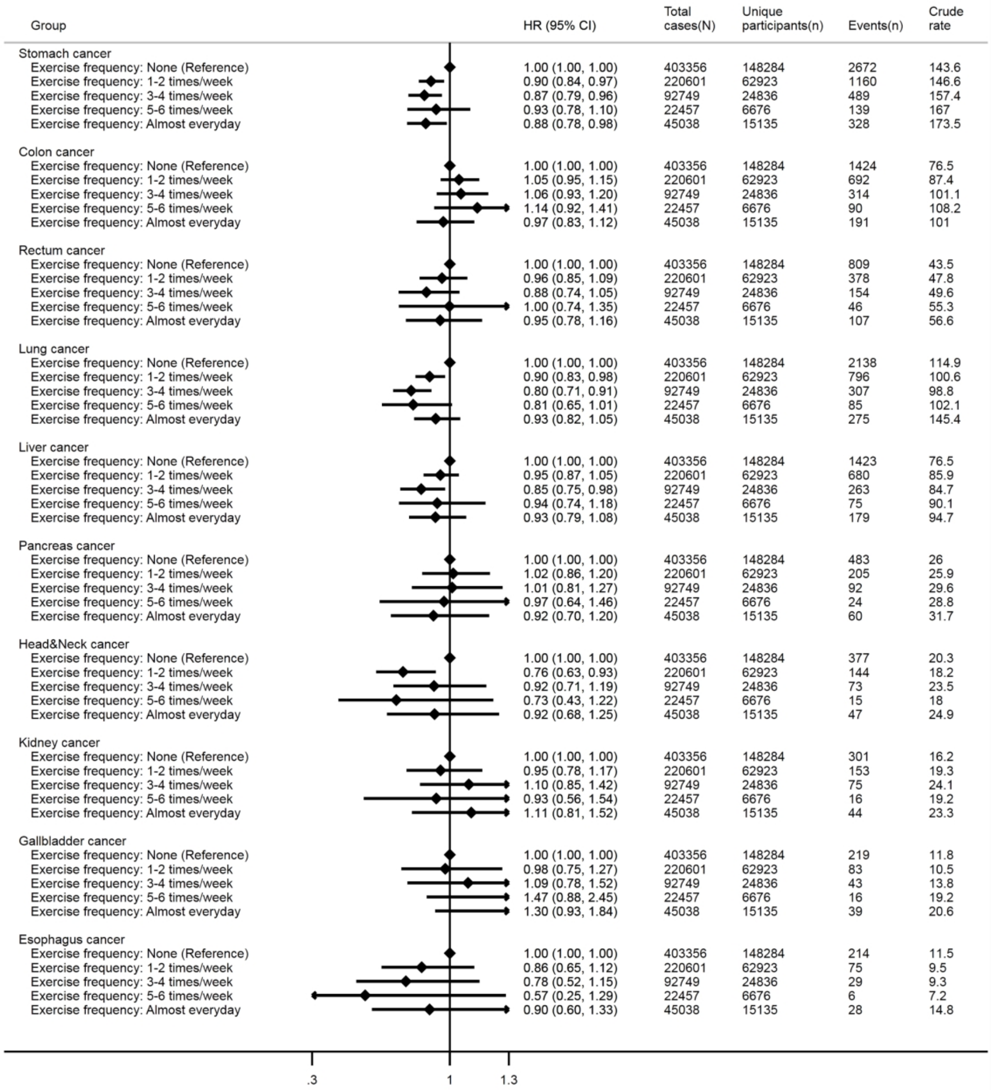

Figure 3. Associations of exercise frequency with various incident cancer outcomes. Cox regression models with age as the underlying timescale were adjusted for sex, body mass index, systolic blood pressure, fasting glucose levels, total cholesterol levels, family history of cancer, smoking status and alcohol consumption. Crude rates are per 100,000 person-years. “N” indicates numbers of total observations (i.e. participants who provided repeated measures are treated as separate observations) and “n” indicates numbers of unique participants at baseline. Data were obtained from a prospective cohort, which has been established by linking physical examination data of over half a million South Korean adults (2002-2015) with the entire South Korean population’s health insurance system. Abbreviations: HR – Hazard Ratio; CI – Confidence Intervals

268x297mm (300 x 300 DPI)

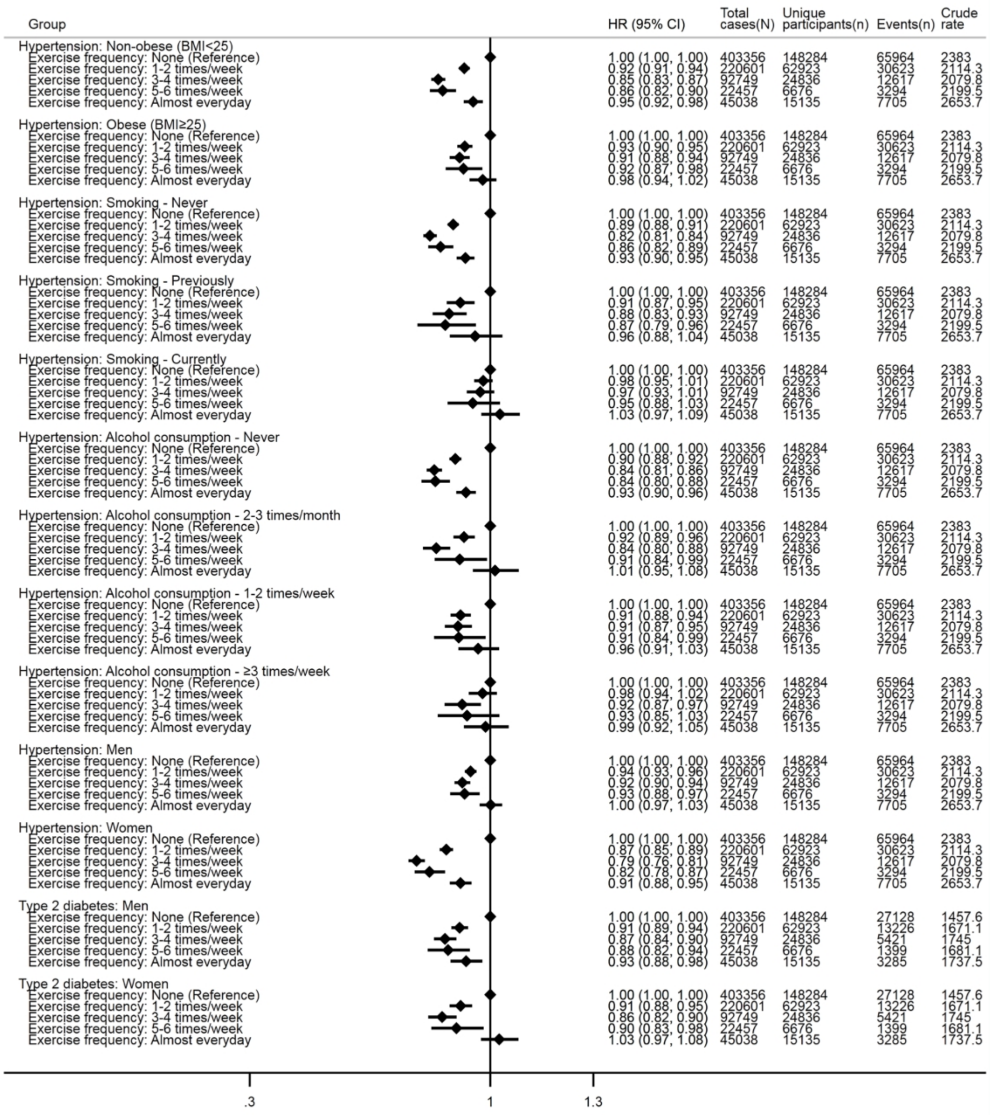

Figure 4. Results from assessment of effect modification of sex, body mass index, smoking, alcohol consumption, and family history of disease in the associations between exercise frequency and each incident disease outcome. Only associations for which the multiplicative interaction terms were statistically significant are presented. Cox regression models with age as the underlying timescale were adjusted for sex [not in models for effect modification by sex], body mass index [not in models for effect modification by body mass index], systolic blood pressure, fasting glucose levels, total cholesterol levels, family history [not in models for effect modification by family history of respective disease] of heart disease/stroke/hypertension (in models for myocardial infarction, stroke and hypertension), diabetes (in models for Type 2 diabetes) or cancer (in models for each cancer), smoking status [not in models for effect modification by smoking status] and alcohol consumption [not in models for effect modification by alcohol consumption]. Crude rates are per 100,000 person-years. "N" indicates numbers of total observations (i.e. participants who provided repeated measures are treated as separate observations) and "n" indicates numbers of unique participants at baseline. P-values for multiplicative interactions with exercise frequency are as follows; Outcome – hypertension: body mass index (0.049), smoking (<0.001), alcohol consumption (<0.001), family history of cardiovascular disease (0.029) and sex (<0.001); Outcome - lung cancer: body mass index (0.016), and; Outcome - type 2 diabetes: sex (0.012)¬¬¬¬¬¬¬. Data were obtained from a prospective cohort, which has been established by linking physical examination data of over half a million South Korean adults (2002-

2015) with the entire South Korean population's health insurance system. Abbreviations: HR – Hazard Ratio; CI – Confidence Intervals

Supplementary Table 1. Definitions for classification of incident disease outcomes

Incident disease outcome	Definition
Myocardial infarction	ICD 10 codes I21-I23
Stroke	ICD 10 codes I60-I64
Hypertension	ICD 10 codes I10, I11 and I15, systolic blood pressure ≥140 mm Hg, diastolic blood pressure ≥90 mm Hg, or physician diagnosis
Type 2 diabetes	ICD 10 codes E11, fasting glucose ≥126 mg/dL, or physician diagnosis
Stomach cancer	ICD 10 codes C16
Colon cancer	ICD 10 codes C18
Rectum cancer	ICD 10 codes C20
Lung cancer	ICD 10 codes C34
Liver cancer	ICD 10 codes C22
Pancreas cancer	ICD 10 codes C25
Head & neck cancer	ICD 10 codes C00-C06, C09-C14 and C32
Kidney cancer	ICD 10 codes C64
Gallbladder cancer	ICD 10 codes C23
Esophagus cancer	ICD 10 codes C15

Abbreviations: ICD – International Classification of Disease

For peer review only

Supplementary Table 2. Participants' characteristics at 1st, 2nd, 3rd, 4th, 5th and 6th repeat-visit

Repeat visit	Variables	All (n=215,295)	Exercise frequency				
1st			None (n=112,156)	1-2 times/week (n=57,777)	3-4 times/week (n=25,390)	5-6 times/week (n= 6,443)	Almost everyday (n=13,529)
	Sex, %						
	Men	51.1%	45.0%	61.8%	55.3%	50.6%	48.8%
	Women	48.9%	55.0%	38.2%	44.7%	49.4%	51.2%
	Age, years	52.7 (8.6)	53.6 (9.2)	50.9 (7.4)	51.3 (7.5)	52.3 (8.0)	55.6 (9.1)
	Body Mass Index, kg^2/m	23.5 (2.8)	23.4 (2.9)	23.6 (2.7)	23.7 (2.6)	23.6 (2.6)	23.7 (2.7)
	Systolic blood pressure, mm Hg	120.5 (14.4)	120.6 (14.7)	120.4 (13.9)	120.0 (13.9)	119.6 (13.9)	121.4 (14.6)
	Diastolic blood pressure, mm Hg	75.7 (9.9)	75.6 (9.9)	75.9 (9.8)	75.5 (9.8)	75.2 (9.8)	75.7 (9.8)
	Fasting glucose levels, mg/dL	92.6 (18.5)	92.6 (18.8)	92.7 (17.4)	92.4 (17.8)	92.8 (21.0)	93.3 (20.2)
	Total cholesterol, mg/dL	197.3 (36.2)	197.0 (36.8)	197.3 (35.6)	197.9 (35.1)	197.4 (35.9)	197.8 (36.7)
	Family history of heart disease, stroke or hypertension, %	12.6%	10.7%	14.2%	16.7%	16.7%	12.0%
	Family history of cancer, %	14.3%	12.7%	15.5%	17.4%	19.2%	14.5%
	Family history of diabetes, %	6.0%	5.0%	6.7%	8.2%	9.0%	5.8%
	Smoking status, %						
	Never	70.7%	74.8%	62.4%	68.9%	71.7%	74.8%
	Previously	8.6%	5.8%	11.9%	12.4%	11.9%	8.8%
	Currently	20.8%	19.4%	25.7%	18.7%	16.3%	16.4%
	Alcohol Consumption, %						
	Never	59.8%	66.9%	49.2%	52.7%	54.9%	61.8%
	2-3 times/month	15.6%	12.7%	20.5%	18.6%	18.2%	12.8%
	1-2 times/week	15.8%	11.8%	21.7%	19.9%	17.0%	14.8%
	≥3 times/week	8.8%	8.7%	8.6%	8.7%	10.0%	10.6%

For peer review only

Supplementary Table 2. Participants' characteristics at 1st, 2nd, 3rd, 4th, 5th and 6th repeat-visit (cont.)

Repeat visit	Variables	All (n=150,845)	Exercise frequency				
2nd			None (n=73,660)	1-2 times/week (n=44,211)	3-4 times/week (n=19,593)	5-6 times/week (n=4,771)	Almost everyday (n= 8,610)
	Sex, %						
	Men	54.2%	47.0%	64.6%	57.9%	53.8%	53.5%
	Women	45.8%	53.0%	35.4%	42.1%	46.2%	46.5%
	Age, years	53.3 (8.3)	54.2 (8.9)	51.7 (7.2)	52.3 (7.4)	53.4 (7.8)	56.5 (9.1)
	Body Mass Index, kg^2/m	23.5 (2.7)	23.4 (2.8)	23.6 (2.6)	23.7 (2.6)	23.6 (2.6)	23.6 (2.7)
	Systolic blood pressure, mm Hg	120.9 (14.1)	121.0 (14.5)	120.7 (13.7)	120.4 (13.6)	120.3 (13.7)	122.0 (14.5)
	Diastolic blood pressure, mm Hg	75.8 (9.6)	75.8 (9.7)	76.0 (9.5)	75.5 (9.4)	75.4 (9.4)	76.0 (9.8)
	Fasting glucose levels, mg/dL	93.2 (16.9)	93.1 (17.4)	93.5 (17.0)	93.1 (15.5)	93.5 (15.9)	93.7 (16.2)
	Total cholesterol, mg/dL	197.6 (35.9)	197.7 (36.5)	197.4 (35.4)	197.8 (35.2)	196.7 (34.9)	198.1 (36.5)
	Family history of heart disease, stroke or hypertension, %	13.4%	11.2%	14.6%	17.5%	18.3%	13.1%
	Family history of cancer, %	14.9%	12.9%	16.4%	17.4%	19.7%	15.4%
	Family history of diabetes, %	6.3%	5.2%	7.1%	8.2%	9.0%	6.1%
	Smoking status, %						
	Never	71.0%	76.5%	62.3%	68.7%	71.2%	74.2%
	Previously	9.0%	5.5%	12.5%	13.1%	13.1%	9.4%
	Currently	20.0%	18.0%	25.3%	18.2%	15.8%	16.5%
	Alcohol Consumption, %						
	Never	59.0%	67.6%	48.1%	51.9%	54.1%	60.0%
	2-3 times/month	16.1%	12.6%	20.9%	19.0%	18.9%	13.6%
	1-2 times/week	16.7%	12.1%	22.8%	20.7%	17.5%	15.8%
	≥3 times/week	8.1%	7.8%	8.2%	8.3%	9.5%	10.7%

For peer review only

Supplementary Table 2. Participants' characteristics at 1st, 2nd, 3rd, 4th, 5th and 6th repeat-visit (cont.)

Repeat visit	Variables	All (n=80,639)	Exercise frequency				
3rd			None (n= 36,123)	1-2 times/week (n=26,505)	3-4 times/week (n=11,432)	5-6 times/week (n=2,391)	Almost everyday (n=4,188)
	Sex, %						
	Men	63.1%	53.9%	73.0%	68.6%	64.5%	63.4%
	Women	36.9%	46.1%	27.0%	31.4%	35.6%	36.7%
	Age, years	52.3 (7.2)	52.9 (7.8)	51.2 (6.2)	51.9 (6.6)	52.8 (7.0)	55.2 (8.4)
	Body Mass Index, kg^2/m	23.5 (2.7)	23.3 (2.8)	23.6 (2.6)	23.7 (2.5)	23.7 (2.6)	23.7 (2.7)
	Systolic blood pressure, mm Hg	121.1 (13.6)	120.9 (13.9)	120.9 (13.4)	121.0 (13.2)	120.6 (13.3)	122.0 (14.0)
	Diastolic blood pressure, mm Hg	76.1 (9.4)	76.0 (9.5)	76.4 (9.4)	76.1 (9.1)	75.6 (9.5)	76.3 (9.6)
	Fasting glucose levels, mg/dL	93.7 (16.8)	93.3 (17.1)	94.1 (16.7)	93.9 (15.9)	94.1 (16.5)	94.5 (17.0)
	Total cholesterol, mg/dL	197.1 (35.2)	197.1 (35.5)	197.4 (34.8)	196.9 (34.7)	196.5 (35.2)	196.7 (35.4)
	Family history of heart disease, stroke or hypertension, %	13.5%	11.4%	14.8%	16.7%	18.6%	11.7%
	Family history of cancer, %	14.8%	13.0%	15.8%	17.8%	18.9%	14.7%
	Family history of diabetes, %	6.4%	5.3%	7.0%	8.0%	9.5%	6.2%
	Smoking status, %						
	Never	66.6%	73.8%	57.6%	63.9%	65.5%	68.8%
	Previously	10.7%	6.1%	14.0%	15.5%	16.6%	12.4%
	Currently	22.8%	20.2%	28.4%	20.6%	17.9%	18.7%
	Alcohol Consumption, %						
	Never	53.6%	64.2%	42.5%	47.2%	47.4%	53.9%
	2-3 times/month	18.5%	14.0%	23.7%	20.8%	21.4%	15.8%
	1-2 times/week	19.5%	14.0%	25.6 %	22.8%	20.7%	18.8%
	≥3 times/week	8.4%	7.8%	8.3%	9.2%	10.5%	11.5%

For peer review only

Supplementary Table 2. Participants' characteristics at 1st, 2nd, 3rd, 4th, 5th and 6th repeat-visit (cont.)

Repeat visit	Variables	All (n=40,910)	Exercise frequency				
4th			None (n=17,678)	1-2 times/week (n=14,624)	3-4 times/week (n= 5,685)	5-6 times/week (n=1,094)	Almost everyday (n=1,829)
	Sex, %						
	Men	72.3%	61.6%	81.1%	81.5%	78.2%	73.9%
	Women	27.7%	38.4%	18.9%	18.5%	21.9%	26.1%
	Age, years	50.9 (5.4)	51.2 (5.5)	50.6 (5.1)	50.7 (5.3)	51.0 (5.6)	52.6 (6.3)
	Body Mass Index, kg^2/m	23.5 (2.7)	23.3 (2.7)	23.7 (2.6)	23.8 (2.6)	23.8 (2.5)	23.7 (2.6)
	Systolic blood pressure, mm Hg	121.4 (13.3)	120.9 (13.5)	121.7 (13.0)	121.5 (12.8)	121.5 (13.1)	122.7 (13.6)
	Diastolic blood pressure, mm Hg	76.6 (9.2)	76.2 (9.3)	77.0 (9.2)	76.8 (8.9)	76.8 (9.4)	77.2 (9.5)
	Fasting glucose levels, mg/dL	93.9 (17.8)	93.3 (17.9)	94.4 (17.9)	94.3 (17.1)	94.0 (16.0)	94.9 (18.8)
	Total cholesterol, mg/dL	196.8 (35.0)	197.2 (35.5)	196.7 (34.6)	196.6 (34.2)	195.0 (34.3)	196.5 (34.9)
	Family history of heart disease, stroke or hypertension, %	12.3%	10.4%	13.7%	14.2%	16.3%	11.6%
	Family history of cancer, %	13.8%	12.6%	14.7%	15.0%	16.3%	13.5%
	Family history of diabetes, %	5.7%	4.8%	6.2%	6.9%	7.5%	6.7%
	Smoking status, %						
	Never	60.6%	69.5%	51.9%	55.5%	57.3%	62.0%
	Previously	12.2%	6.9%	15.1%	18.7%	18.7%	15.7%
	Currently	27.2%	23.7%	33.0%	25.8%	24.0%	22.4%
	Alcohol Consumption, %						
	Never	48.1%	60.0%	38.0%	39.1%	39.9%	45.4%
	2-3 times/month	20.5%	15.5%	24.9%	24.3%	24.0%	19.7%
	1-2 times/week	22.4%	16.2%	28.4%	26.3%	22.6%	22.9%
	≥3 times/week	9.0%	8.2%	8.7%	10.3%	13.6%	12.0%

For peer review only

Supplementary Table 2. Participants' characteristics at 1st, 2nd, 3rd, 4th, 5th and 6th repeat-visit (cont.)

Repeat visit	Variables	All (n= 26,356)	Exercise frequency				
5th			None (n=10,771)	1-2 times/week (n=9,807)	3-4 times/week (n=3,884)	5-6 times/week (n=724)	Almost everyday (n=1,170)
	Sex, %						
	Men	73.7%	62.6%	81.6%	82.4%	81.2%	76.9%
	Women	26.3%	37.4%	18.4%	17.6%	18.8%	23.0%
	Age, years	51.1 (4.9)	51.4 (5.1)	50.8 (4.7)	51.0 (4.7)	51.4 (5.0)	52.1 (5.6)
	Body Mass Index, kg^2/m	23.5 (2.6)	23.3 (2.7)	23.7 (2.6)	23.8 (2.4)	23.7 (2.5)	23.8 (2.6)
	Systolic blood pressure, mm Hg	121.4 (13.0)	121.0 (13.4)	121.8 (12.9)	121.6 (12.6)	120.8 (12.9)	121.5 (12.7)
	Diastolic blood pressure, mm Hg	76.6 (9.1)	76.2 (9.1)	77.1 (9.0)	76.8 (8.8)	76.7 (8.6)	76.4 (9.0)
	Fasting glucose levels, mg/dL	94.3 (17.6)	93.7 (18.1)	94.6 (17.3)	94.7 (16.6)	94.6 (15.9)	95.4 (18.7)
	Total cholesterol, mg/dL	197.1 (34.5)	197.7 (34.8)	196.7 (34.2)	196.4 (33.8)	195.2 (34.2)	198.2 (37.0)
	Family history of heart disease, stroke or hypertension, %	12.6%	11.3%	13.4%	14.3%	13.8%	10.9%
	Family history of cancer, %	14.3%	13.2%	15.1%	14.9%	17.8%	13.5%
	Family history of diabetes, %	5.9%	4.9%	6.4%	6.7%	7.2%	8.6%
	Smoking status, %						
	Never	58.9%	68.1%	50.8%	54.4%	57.0%	58.5%
	Previously	13.1%	7.1%	16.0%	19.3%	19.8%	19.7%
	Currently	28.0%	24.8%	33.2%	26.2%	23.2%	21.9%
	Alcohol Consumption, %						
	Never	46.3%	57.9%	37.6%	38.3%	41.3%	42.9%
	2-3 times/month	21.7%	16.7%	25.8%	24.7%	22.2%	22.3%
	1-2 times/week	23.4%	17.4%	28.0%	27.3%	26.0%	25.0%
	≥3 times/week	8.6%	8.1%	8.5%	9.8%	10.5%	9.7%

For peer review only

Supplementary Table 2. Participants' characteristics at 1st, 2nd, 3rd, 4th, 5th and 6th repeat-visit (cont.)

Repeat visit	Variables	All (n= 12,302)	Exercise frequency				
6th			None (n= 4,684)	1-2 times/week (n=4,754)	3-4 times/week (n=1,929)	5-6 times/week (n=358)	Almost everyday (n=577)
	Sex, %						
	Men	76.2%	64.2%	83.8%	84.7%	80.7%	80.4%
	Women	23.8%	35.8%	16.2%	15.3%	19.3%	19.6%
	Age, years	51.3 (4.5)	51.6 (4.7)	51.0 (4.2)	51.0 (4.4)	51.2 (4.3)	52.0 (5.1)
	Body Mass Index, kg^2/m	23.6 (2.6)	23.3 (2.7)	23.7 (2.6)	23.8 (2.4)	23.6 (2.2)	23.7 (2.5)
	Systolic blood pressure, mm Hg	121.5 (12.8)	121.2 (13.0)	121.6 (12.6)	121.7 (12.6)	121.0 (12.5)	122.1 (12.3)
	Diastolic blood pressure, mm Hg	76.7 (9.0)	76.4 (9.0)	77.0 (8.9)	77.0 (9.0)	76.0 (8.7)	76.3 (9.0)
	Fasting glucose levels, mg/dL	94.6 (18.5)	93.7 (18.8)	95.1 (18.7)	94.9 (16.6)	95.1 (18.4)	95.7 (20.1)
	Total cholesterol, mg/dL	197.1 (34.5)	197.9 (35.0)	197.1 (34.2)	195.8 (34.3)	192.0 (32.2)	197.4 (34.3)
	Family history of heart disease, stroke or hypertension, %	13.2%	11.7%	13.7%	15.5%	14.3%	12.7%
	Family history of cancer, %	14.5%	12.9%	15.5%	15.7%	15.1%	14.9%
	Family history of diabetes, %	6.4%	5.5%	6.4%	7.3%	7.3%	9.5%
	Smoking status, %						
	Never	56.9%	66.6%	48.7%	53.6%	57.3%	57.4%
	Previously	14.0%	7.0%	17.3%	20.4%	16.8%	21.1%
	Currently	29.1%	26.5%	34.1%	26.0%	26.0%	21.5%
	Alcohol Consumption, %						
	Never	43.7%	56.5%	35.0%	36.4%	40.5%	37.8%
	2-3 times/month	22.8%	17.0%	27.2%	24.9%	24.6%	24.8%
	1-2 times/week	24.8%	17.6%	30.0%	28.3%	26.5%	27.4%
	≥3 times/week	8.8%	8.8%	7.9%	10.4%	8.4%	10.1%

For peer review only

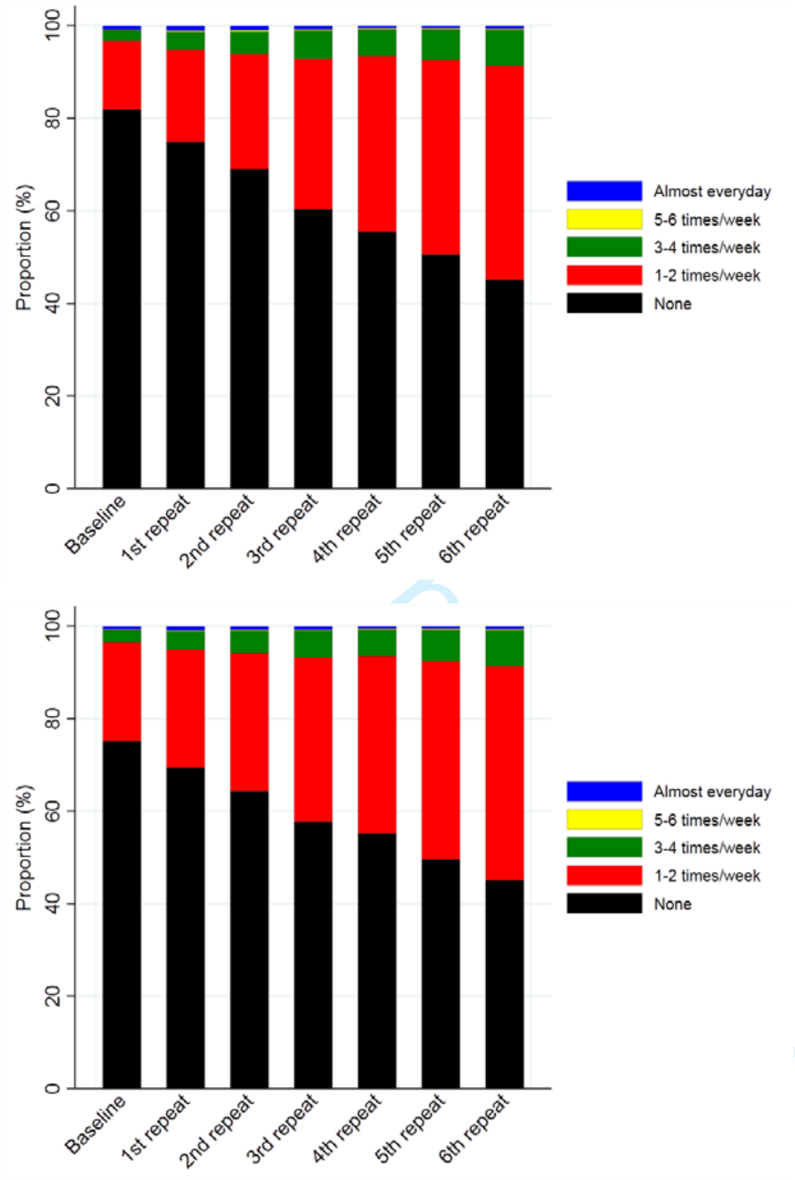

Supplementary Figure 1. Changes in proportions of exercise frequency categories across 7 time points in all 257,854 individuals who provided data at each respective assessment visit (top panel), and 12,302 individuals who provided data from all 7 assessment visits (bottom panel).

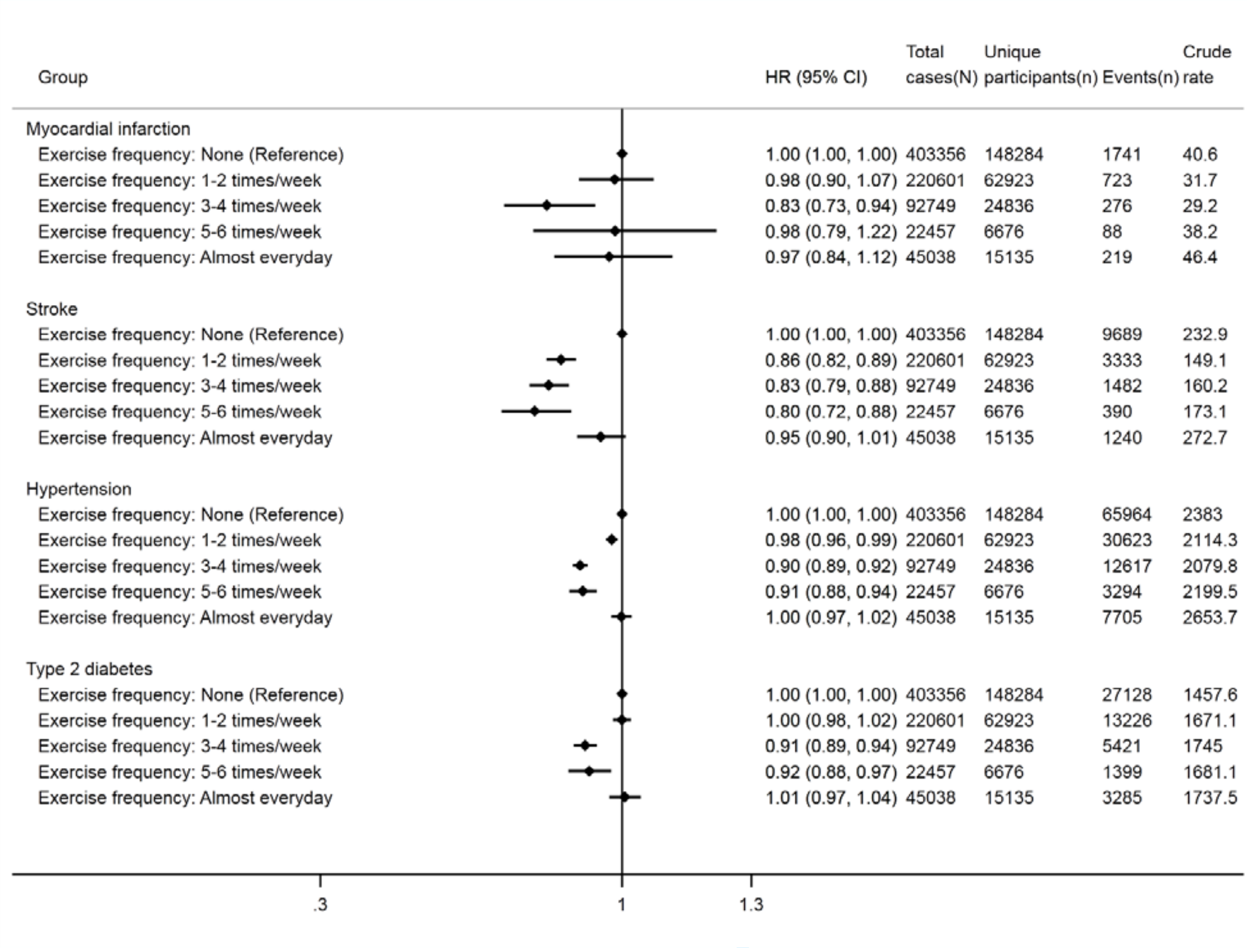
Group
HR (95% CI)
Total cases(N)
Unique participants(n)
Events(n)
Crude rate
Myocardial infarction
Exercise frequency: None (Reference) 1.00 (1.00, 1.00) 403356 148284 1741 40.6
Exercise frequency: 1-2 times/week 0.98 (0.90, 1.07) 220601 62923 723 31.7
Exercise frequency: 3-4 times/week 0.83 (0.73, 0.94) 92749 24836 276 29.2
Exercise frequency: 5-6 times/week 0.98 (0.79, 1.22) 22457 6676 88 38.2
Exercise frequency: Almost everyday 0.97 (0.84, 1.12) 45038 15135 219 46.4
Stroke
Exercise frequency: None (Reference) 1.00 (1.00, 1.00) 403356 148284 9689 232.9
Exercise frequency: 1-2 times/week 0.86 (0.82, 0.89) 220601 62923 3333 149.1
Exercise frequency: 3-4 times/week 0.83 (0.79, 0.88) 92749 24836 1482 160.2
Exercise frequency: 5-6 times/week 0.80 (0.72, 0.88) 22457 6676 390 173.1
Exercise frequency: Almost everyday 0.95 (0.90, 1.01) 45038 15135 1240 272.7
Hypertension
Exercise frequency: None (Reference) 1.00 (1.00, 1.00) 403356 148284 65964 2383
Exercise frequency: 1-2 times/week 0.98 (0.96, 0.99) 220601 62923 30623 2114.3
Exercise frequency: 3-4 times/week 0.90 (0.89, 0.92) 92749 24836 12617 2079.8
Exercise frequency: 5-6 times/week 0.91 (0.88, 0.94) 22457 6676 3294 2199.5
Exercise frequency: Almost everyday 1.00 (0.97, 1.02) 45038 15135 7705 2653.7
Type 2 diabetes
Exercise frequency: None (Reference) 1.00 (1.00, 1.00) 403356 148284 27128 1457.6
Exercise frequency: 1-2 times/week 1.00 (0.98, 1.02) 220601 62923 13226 1671.1
Exercise frequency: 3-4 times/week 0.91 (0.89, 0.94) 92749 24836 5421 1745
Exercise frequency: 5-6 times/week 0.92 (0.88, 0.97) 22457 6676 1399 1681.1
Exercise frequency: Almost everyday 1.01 (0.97, 1.04) 45038 15135 3285 1737.5
.3
1
1.3

w only

Group	HR (95% CI)	Total cases(N)	Unique participants(n)	Events(n)	Crude rate
Stomach cancer					
Exercise frequency: None (Reference)	1.00 (1.00, 1.00)	403356	148284	2672	143.6
Exercise frequency: 1-2 times/week	1.01 (0.95, 1.09)	220601	62923	1160	146.6
Exercise frequency: 3-4 times/week	0.94 (0.85, 1.03)	92749	24836	489	157.4
Exercise frequency: 5-6 times/week	0.99 (0.83, 1.17)	22457	6676	139	167
Exercise frequency: Almost everyday	0.93 (0.83, 1.04)	45038	15135	328	173.5
Colon cancer					
Exercise frequency: None (Reference)	1.00 (1.00, 1.00)	403356	148284	1424	76.5
Exercise frequency: 1-2 times/week	1.13 (1.03, 1.24)	220601	62923	692	87.4
Exercise frequency: 3-4 times/week	1.12 (0.99, 1.27)	92749	24836	314	101.1
Exercise frequency: 5-6 times/week	1.20 (0.97, 1.48)	22457	6676	90	108.2
Exercise frequency: Almost everyday	1.02 (0.87, 1.18)	45038	15135	191	101
Rectum cancer					
Exercise frequency: None (Reference)	1.00 (1.00, 1.00)	403356	148284	809	43.5
Exercise frequency: 1-2 times/week	1.07 (0.94, 1.21)	220601	62923	378	47.8
Exercise frequency: 3-4 times/week	0.95 (0.80, 1.13)	92749	24836	154	49.6
Exercise frequency: 5-6 times/week	1.07 (0.79, 1.43)	22457	6676	46	55.3
Exercise frequency: Almost everyday	1.01 (0.82, 1.23)	45038	15135	107	56.6
Lung cancer					
Exercise frequency: None (Reference)	1.00 (1.00, 1.00)	403356	148284	2138	114.9
Exercise frequency: 1-2 times/week	0.97 (0.89, 1.05)	220601	62923	796	100.6
Exercise frequency: 3-4 times/week	0.81 (0.72, 0.91)	92749	24836	307	98.8
Exercise frequency: 5-6 times/week	0.80 (0.65, 1.00)	22457	6676	85	102.1
Exercise frequency: Almost everyday	0.94 (0.83, 1.07)	45038	15135	275	145.4
Liver cancer					
Exercise frequency: None (Reference)	1.00 (1.00, 1.00)	403356	148284	1423	76.5
Exercise frequency: 1-2 times/week	1.07 (0.97, 1.17)	220601	62923	680	85.9
Exercise frequency: 3-4 times/week	0.91 (0.80, 1.04)	92749	24836	263	84.7
Exercise frequency: 5-6 times/week	0.99 (0.78, 1.24)	22457	6676	75	90.1
Exercise frequency: Almost everyday	0.99 (0.84, 1.15)	45038	15135	179	94.7
Pancreas cancer					
Exercise frequency: None (Reference)	1.00 (1.00, 1.00)	403356	148284	483	26
Exercise frequency: 1-2 times/week	1.05 (0.89, 1.24)	220601	62923	205	25.9
Exercise frequency: 3-4 times/week	1.03 (0.82, 1.29)	92749	24836	92	29.6
Exercise frequency: 5-6 times/week	0.98 (0.65, 1.48)	22457	6676	24	28.8
Exercise frequency: Almost everyday	0.93 (0.71, 1.22)	45038	15135	60	31.7
Head&Neck cancer					
Exercise frequency: None (Reference)	1.00 (1.00, 1.00)	403356	148284	377	20.3
Exercise frequency: 1-2 times/week	0.86 (0.71, 1.05)	220601	62923	144	18.2
Exercise frequency: 3-4 times/week	0.96 (0.75, 1.24)	92749	24836	73	23.5
Exercise frequency: 5-6 times/week	0.75 (0.44, 1.25)	22457	6676	15	18
Exercise frequency: Almost everyday	0.96 (0.71, 1.29)	45038	15135	47	24.9
Kidney cancer					
Exercise frequency: None (Reference)	1.00 (1.00, 1.00)	403356	148284	301	16.2
Exercise frequency: 1-2 times/week	1.10 (0.90, 1.34)	220601	62923	153	19.3
Exercise frequency: 3-4 times/week	1.20 (0.93, 1.54)	92749	24836	75	24.1
Exercise frequency: 5-6 times/week	0.98 (0.59, 1.62)	22457	6676	16	19.2
Exercise frequency: Almost everyday	1.17 (0.85, 1.60)	45038	15135	44	23.3
Gallbladder cancer					
Exercise frequency: None (Reference)	1.00 (1.00, 1.00)	403356	148284	219	11.8
Exercise frequency: 1-2 times/week	0.97 (0.75, 1.25)	220601	62923	83	10.5
Exercise frequency: 3-4 times/week	1.09 (0.79, 1.52)	92749	24836	43	13.8
Exercise frequency: 5-6 times/week	1.48 (0.89, 2.45)	22457	6676	16	19.2
Exercise frequency: Almost everyday	1.32 (0.94, 1.86)	45038	15135	39	20.6
Esophagus cancer					
Exercise frequency: None (Reference)	1.00 (1.00, 1.00)	403356	148284	214	11.5
Exercise frequency: 1-2 times/week	0.91 (0.70, 1.19)	220601	62923	75	9.5
Exercise frequency: 3-4 times/week	0.76 (0.51, 1.12)	92749	24836	29	9.3
Exercise frequency: 5-6 times/week	0.56 (0.25, 1.26)	22457	6676	6	7.2
Exercise frequency: Almost everyday	0.93 (0.62, 1.37)	45038	15135	28	14.8

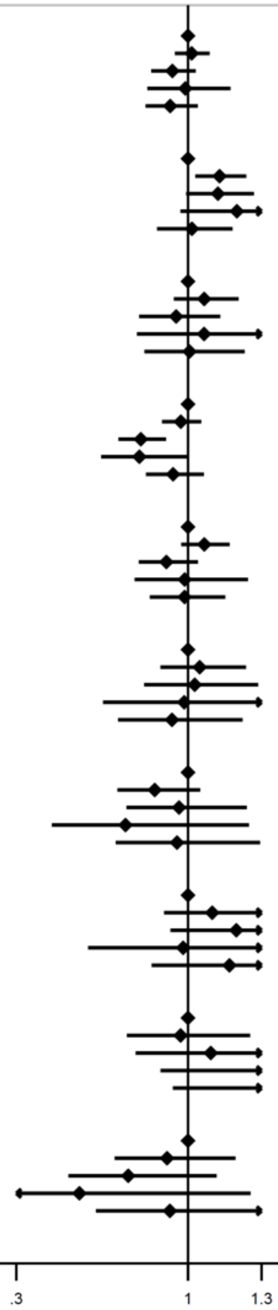

Supplementary Figure 2. Associations of exercise with incident myocardial infarction, stroke, hypertension and Type 2 diabetes (top panel) and various incident cancer outcomes (bottom panel). Note: Cox regression models using age as the underlying timescale were not adjusted for any confounders. Crude rates are per 100,000 person-years. “N” indicates numbers of total observations (i.e. participants who provided repeated measures are treated as separate observations) and “n” indicates numbers of unique participants at baseline. Abbreviations: HR – Hazard Ratio; CI – Confidence Intervals

Group	HR (95% CI)	Total cases(N)	Unique participants(n)	Events(n)	Crude rate
**Myocardial infarction**					
Exercise frequency: None (Reference)	1.00 (1.00, 1.00)	403105	148033	1526	35.6
Exercise frequency: 1-2 times/week	0.89 (0.81, 0.98)	220498	62820	635	27.9
Exercise frequency: 3-4 times/week	0.82 (0.71, 0.94)	92715	24802	246	26
Exercise frequency: 5-6 times/week	0.95 (0.75, 1.20)	22439	6658	74	32.1
Exercise frequency: Almost everyday	0.95 (0.82, 1.11)	45014	15111	197	41.8
**Stroke**					
Exercise frequency: None (Reference)	1.00 (1.00, 1.00)	403105	148033	9669	232.5
Exercise frequency: 1-2 times/week	0.86 (0.82, 0.89)	220498	62820	3330	149
Exercise frequency: 3-4 times/week	0.83 (0.79, 0.88)	92715	24802	1481	160.1
Exercise frequency: 5-6 times/week	0.80 (0.73, 0.89)	22439	6658	389	172.8
Exercise frequency: Almost everyday	0.95 (0.90, 1.01)	45014	15111	1239	272.6
**Hypertension**					
Exercise frequency: None (Reference)	1.00 (1.00, 1.00)	403105	148033	65872	2381.3
Exercise frequency: 1-2 times/week	0.92 (0.90, 0.93)	220498	62820	30585	2112.7
Exercise frequency: 3-4 times/week	0.86 (0.84, 0.88)	92715	24802	12605	2078.6
Exercise frequency: 5-6 times/week	0.87 (0.84, 0.90)	22439	6658	3288	2197.2
Exercise frequency: Almost everyday	0.95 (0.93, 0.97)	45014	15111	7694	2651.1
**Type 2 diabetes**					
Exercise frequency: None (Reference)	1.00 (1.00, 1.00)	403105	148033	27101	1456.3
Exercise frequency: 1-2 times/week	0.92 (0.90, 0.94)	220498	62820	13214	1669.8
Exercise frequency: 3-4 times/week	0.87 (0.84, 0.89)	92715	24802	5415	1743.2
Exercise frequency: 5-6 times/week	0.88 (0.84, 0.93)	22439	6658	1397	1679.2
Exercise frequency: Almost everyday	0.96 (0.93, 1.00)	45014	15111	3282	1736.1

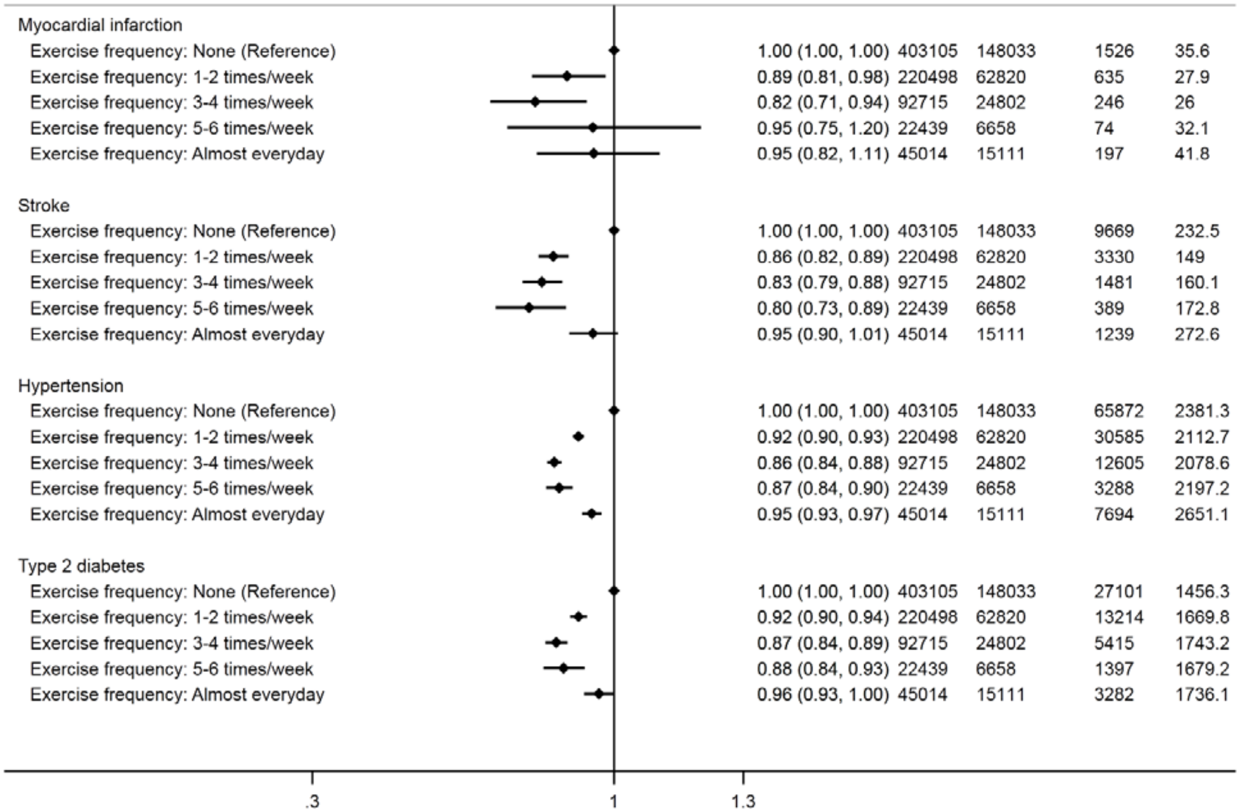

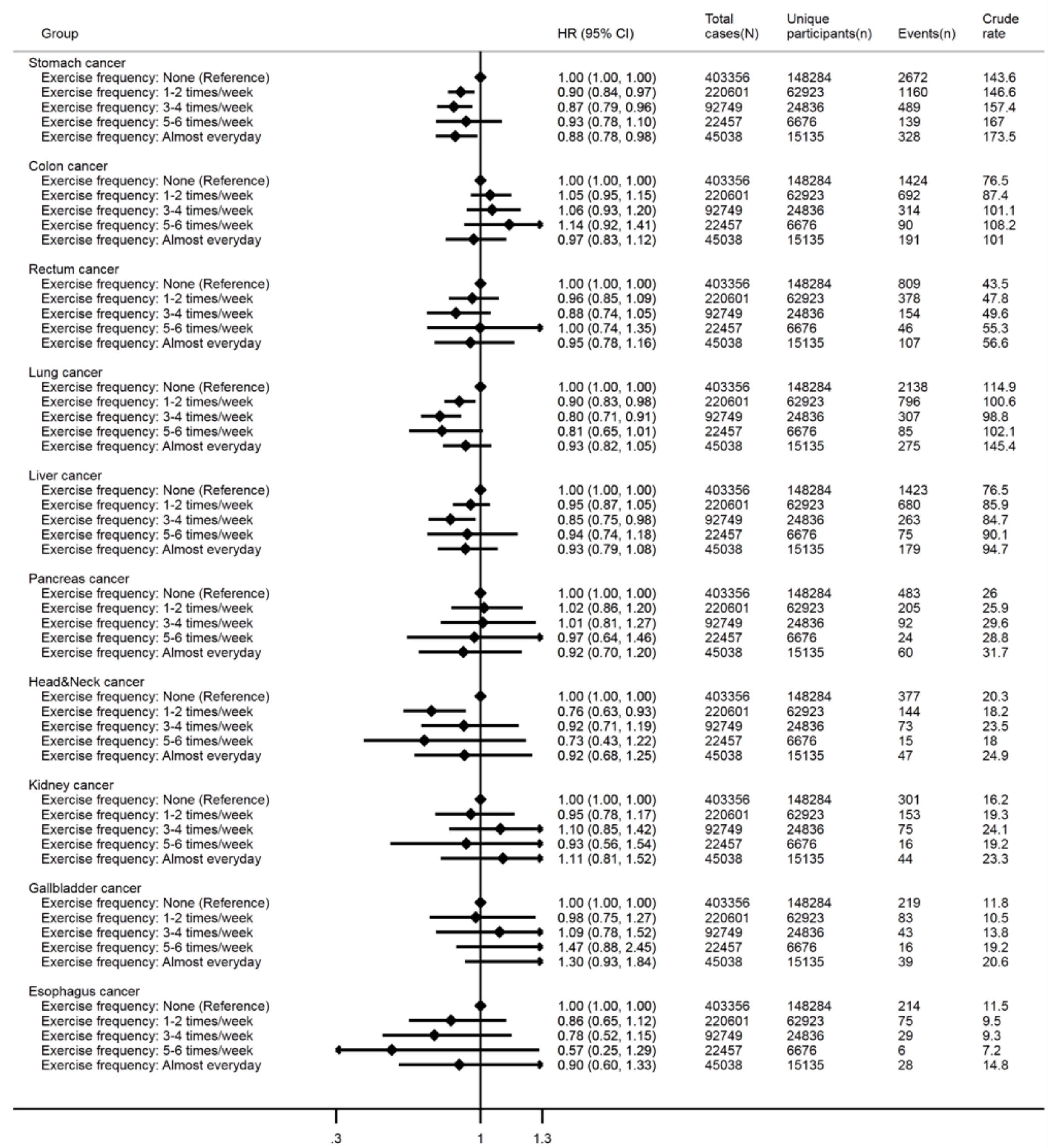

Group	HR (95% CI)	Total cases(N)	Unique participants(n)	Events(n)	Crude rate
**Stomach cancer**					
Exercise frequency: None (Reference)	1.00 (1.00, 1.00)	403356	148284	2672	143.6
Exercise frequency: 1-2 times/week	0.90 (0.84, 0.97)	220601	62923	1160	146.6
Exercise frequency: 3-4 times/week	0.87 (0.79, 0.96)	92749	24836	489	157.4
Exercise frequency: 5-6 times/week	0.93 (0.78, 1.10)	22457	6676	139	167
Exercise frequency: Almost everyday	0.88 (0.78, 0.98)	45038	15135	328	173.5
**Colon cancer**					
Exercise frequency: None (Reference)	1.00 (1.00, 1.00)	403356	148284	1424	76.5
Exercise frequency: 1-2 times/week	1.05 (0.95, 1.15)	220601	62923	692	87.4
Exercise frequency: 3-4 times/week	1.06 (0.93, 1.20)	92749	24836	314	101.1
Exercise frequency: 5-6 times/week	1.14 (0.92, 1.41)	22457	6676	90	108.2
Exercise frequency: Almost everyday	0.97 (0.83, 1.12)	45038	15135	191	101
**Rectum cancer**					
Exercise frequency: None (Reference)	1.00 (1.00, 1.00)	403356	148284	809	43.5
Exercise frequency: 1-2 times/week	0.96 (0.85, 1.09)	220601	62923	378	47.8
Exercise frequency: 3-4 times/week	0.88 (0.74, 1.05)	92749	24836	154	49.6
Exercise frequency: 5-6 times/week	1.00 (0.74, 1.35)	22457	6676	46	55.3
Exercise frequency: Almost everyday	0.95 (0.78, 1.16)	45038	15135	107	56.6
**Lung cancer**					
Exercise frequency: None (Reference)	1.00 (1.00, 1.00)	403356	148284	2138	114.9
Exercise frequency: 1-2 times/week	0.90 (0.83, 0.98)	220601	62923	796	100.6
Exercise frequency: 3-4 times/week	0.80 (0.71, 0.91)	92749	24836	307	98.8
Exercise frequency: 5-6 times/week	0.81 (0.65, 1.01)	22457	6676	85	102.1
Exercise frequency: Almost everyday	0.93 (0.82, 1.05)	45038	15135	275	145.4
**Liver cancer**					
Exercise frequency: None (Reference)	1.00 (1.00, 1.00)	403356	148284	1423	76.5
Exercise frequency: 1-2 times/week	0.95 (0.87, 1.05)	220601	62923	680	85.9
Exercise frequency: 3-4 times/week	0.85 (0.75, 0.98)	92749	24836	263	84.7
Exercise frequency: 5-6 times/week	0.94 (0.74, 1.18)	22457	6676	75	90.1
Exercise frequency: Almost everyday	0.93 (0.79, 1.08)	45038	15135	179	94.7
**Pancreas cancer**					
Exercise frequency: None (Reference)	1.00 (1.00, 1.00)	403356	148284	483	26
Exercise frequency: 1-2 times/week	1.02 (0.86, 1.20)	220601	62923	205	25.9
Exercise frequency: 3-4 times/week	1.01 (0.81, 1.27)	92749	24836	92	29.6
Exercise frequency: 5-6 times/week	0.97 (0.64, 1.46)	22457	6676	24	28.8
Exercise frequency: Almost everyday	0.92 (0.70, 1.20)	45038	15135	60	31.7
**Head&Neck cancer**					
Exercise frequency: None (Reference)	1.00 (1.00, 1.00)	403356	148284	377	20.3
Exercise frequency: 1-2 times/week	0.76 (0.63, 0.93)	220601	62923	144	18.2
Exercise frequency: 3-4 times/week	0.92 (0.71, 1.19)	92749	24836	73	23.5
Exercise frequency: 5-6 times/week	0.73 (0.43, 1.22)	22457	6676	15	18
Exercise frequency: Almost everyday	0.92 (0.68, 1.25)	45038	15135	47	24.9
**Kidney cancer**					
Exercise frequency: None (Reference)	1.00 (1.00, 1.00)	403356	148284	301	16.2
Exercise frequency: 1-2 times/week	0.95 (0.78, 1.17)	220601	62923	153	19.3
Exercise frequency: 3-4 times/week	1.10 (0.85, 1.42)	92749	24836	75	24.1
Exercise frequency: 5-6 times/week	0.93 (0.56, 1.54)	22457	6676	16	19.2
Exercise frequency: Almost everyday	1.11 (0.81, 1.52)	45038	15135	44	23.3
**Gallbladder cancer**					
Exercise frequency: None (Reference)	1.00 (1.00, 1.00)	403356	148284	219	11.8
Exercise frequency: 1-2 times/week	0.98 (0.75, 1.27)	220601	62923	83	10.5
Exercise frequency: 3-4 times/week	1.09 (0.78, 1.52)	92749	24836	43	13.8
Exercise frequency: 5-6 times/week	1.47 (0.88, 2.45)	22457	6676	16	19.2
Exercise frequency: Almost everyday	1.30 (0.93, 1.84)	45038	15135	39	20.6
**Esophagus cancer**					
Exercise frequency: None (Reference)	1.00 (1.00, 1.00)	403356	148284	214	11.5
Exercise frequency: 1-2 times/week	0.86 (0.65, 1.12)	220601	62923	75	9.5
Exercise frequency: 3-4 times/week	0.78 (0.52, 1.15)	92749	24836	29	9.3
Exercise frequency: 5-6 times/week	0.57 (0.25, 1.29)	22457	6676	6	7.2
Exercise frequency: Almost everyday	0.90 (0.60, 1.33)	45038	15135	28	14.8

Supplementary Figure 3. Associations of exercise with incident myocardial infarction, stroke, hypertension and Type 2 diabetes (top panel) and various incident cancer outcomes (bottom panel) after excluding data from the first 2-year follow-up period. Note: Cox regression models using age as the underlying timescale were adjusted for sex, body mass index, systolic blood pressure, fasting glucose levels, total cholesterol levels, family history of heart disease, stroke or hypertension (in models for incident myocardial infarction, stroke and hypertension), diabetes (in models for incident Type 2 diabetes) or cancer (in models for incident cancer outcomes), smoking status and alcohol consumption. Crude rates are per 100,000 person-years. “N”

indicates numbers of total observations (i.e. participants who provided repeated measures are treated as separate observations) and "n" indicates numbers of unique participants at baseline. Abbreviations: HR – Hazard Ratio; CI – Confidence Intervals

For peer review only

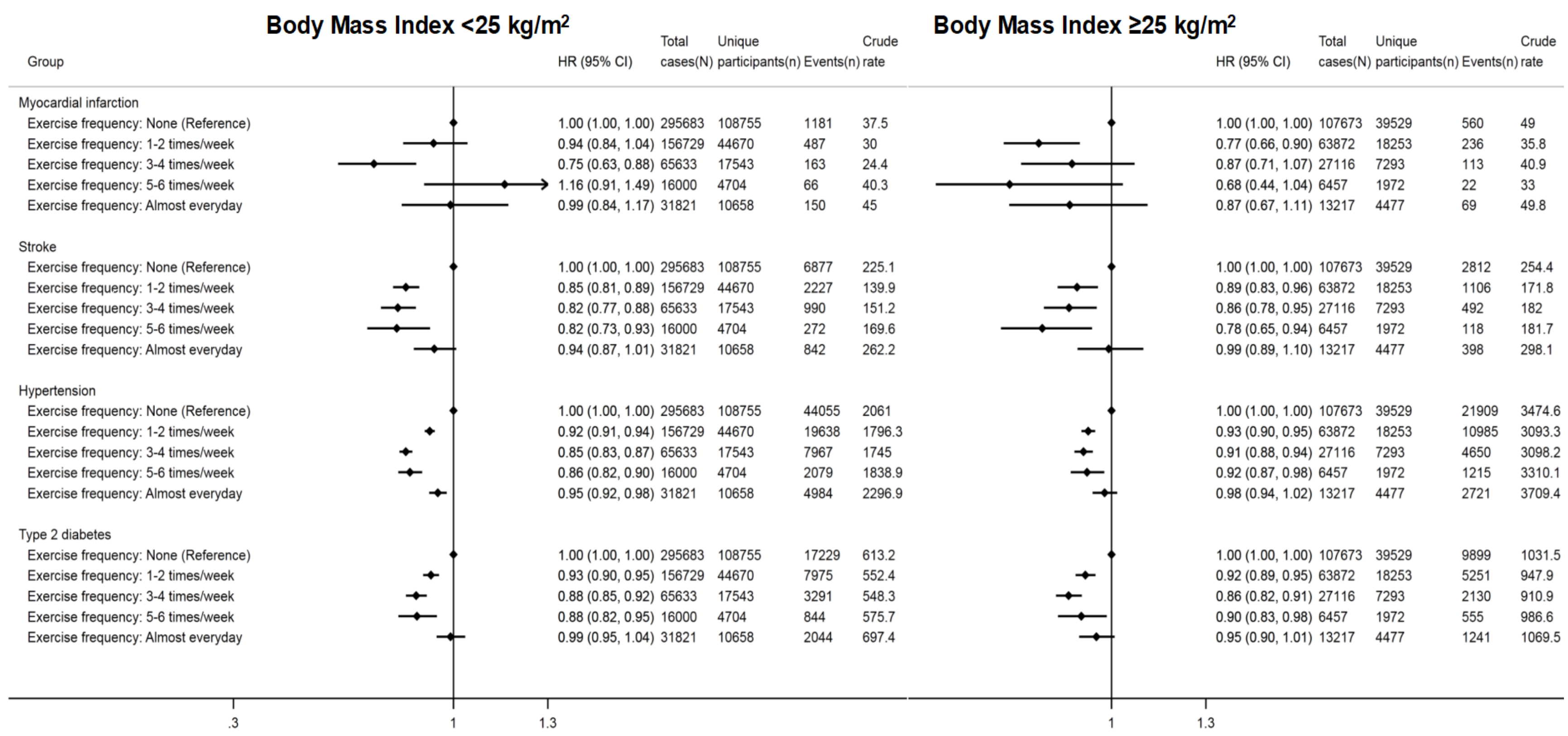
Body Mass Index <25 kg/m²

Group	HR (95% CI)	Total cases(N)	Unique participants(n)	Events(n)	Crude rate
Myocardial infarction					
Exercise frequency: None (Reference)	1.00 (1.00, 1.00)	295683	108755	1181	37.5
Exercise frequency: 1-2 times/week	0.94 (0.84, 1.04)	156729	44670	487	30
Exercise frequency: 3-4 times/week	0.75 (0.63, 0.88)	65633	17543	163	24.4
Exercise frequency: 5-6 times/week	1.16 (0.91, 1.49)	16000	4704	66	40.3
Exercise frequency: Almost everyday	0.99 (0.84, 1.17)	31821	10658	150	45
Stroke					
Exercise frequency: None (Reference)	1.00 (1.00, 1.00)	295683	108755	6877	225.1
Exercise frequency: 1-2 times/week	0.85 (0.81, 0.89)	156729	44670	2227	139.9
Exercise frequency: 3-4 times/week	0.82 (0.77, 0.88)	65633	17543	990	151.2
Exercise frequency: 5-6 times/week	0.82 (0.73, 0.93)	16000	4704	272	169.6
Exercise frequency: Almost everyday	0.94 (0.87, 1.01)	31821	10658	842	262.2
Hypertension					
Exercise frequency: None (Reference)	1.00 (1.00, 1.00)	295683	108755	44055	2061
Exercise frequency: 1-2 times/week	0.92 (0.91, 0.94)	156729	44670	19638	1796.3
Exercise frequency: 3-4 times/week	0.85 (0.83, 0.87)	65633	17543	7967	1745
Exercise frequency: 5-6 times/week	0.86 (0.82, 0.90)	16000	4704	2079	1838.9
Exercise frequency: Almost everyday	0.95 (0.92, 0.98)	31821	10658	4984	2296.9
Type 2 diabetes					
Exercise frequency: None (Reference)	1.00 (1.00, 1.00)	295683	108755	17229	613.2
Exercise frequency: 1-2 times/week	0.93 (0.90, 0.95)	156729	44670	7975	552.4
Exercise frequency: 3-4 times/week	0.88 (0.85, 0.92)	65633	17543	3291	548.3
Exercise frequency: 5-6 times/week	0.88 (0.82, 0.95)	16000	4704	844	575.7
Exercise frequency: Almost everyday	0.99 (0.95, 1.04)	31821	10658	2044	697.4

.3
1
1.3

Body Mass Index ≥25 kg/m²

HR (95% CI)	Total cases(N)	Unique participants(n)	Events(n)	Crude rate
1.00 (1.00, 1.00)	107673	39529	560	49
0.77 (0.66, 0.90)	63872	18253	236	35.8
0.87 (0.71, 1.07)	27116	7293	113	40.9
0.68 (0.44, 1.04)	6457	1972	22	33
0.87 (0.67, 1.11)	13217	4477	69	49.8
1.00 (1.00, 1.00)	107673	39529	2812	254.4
0.89 (0.83, 0.96)	63872	18253	1106	171.8
0.86 (0.78, 0.95)	27116	7293	492	182
0.78 (0.65, 0.94)	6457	1972	118	181.7
0.99 (0.89, 1.10)	13217	4477	398	298.1
1.00 (1.00, 1.00)	107673	39529	21909	3474.6
0.93 (0.90, 0.95)	63872	18253	10985	3093.3
0.91 (0.88, 0.94)	27116	7293	4650	3098.2
0.92 (0.87, 0.98)	6457	1972	1215	3310.1
0.98 (0.94, 1.02)	13217	4477	2721	3709.4
1.00 (1.00, 1.00)	107673	39529	9899	1031.5
0.92 (0.89, 0.95)	63872	18253	5251	947.9
0.86 (0.82, 0.91)	27116	7293	2130	910.9
0.90 (0.83, 0.98)	6457	1972	555	986.6
0.95 (0.90, 1.01)	13217	4477	1241	1069.5

1
1.3

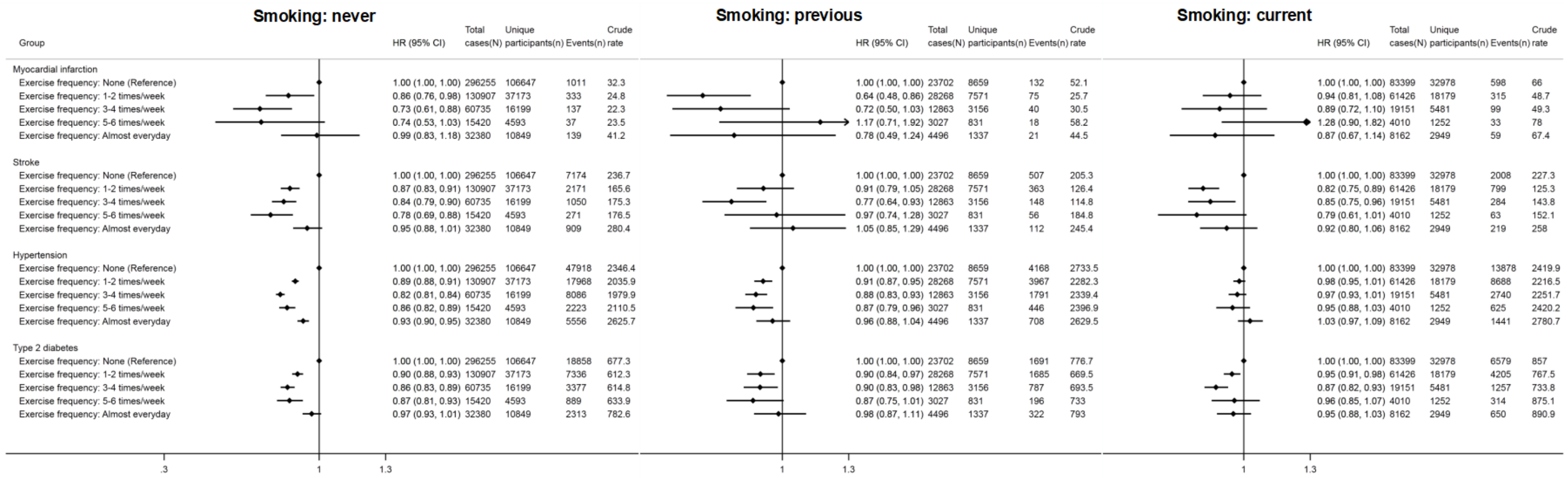
Smoking: never
Group
HR (95% CI)
Total cases(N)
Unique participants(n)
Events(n)
Crude rate
Myocardial infarction
Exercise frequency: None (Reference) 1.00 (1.00, 1.00) 296255 106647 1011 32.3
Exercise frequency: 1-2 times/week 0.86 (0.76, 0.98) 130907 37173 333 24.8
Exercise frequency: 3-4 times/week 0.73 (0.61, 0.88) 60735 16199 137 22.3
Exercise frequency: 5-6 times/week 0.74 (0.53, 1.03) 15420 4593 37 23.5
Exercise frequency: Almost everyday 0.99 (0.83, 1.18) 32380 10849 139 41.2
Stroke
Exercise frequency: None (Reference) 1.00 (1.00, 1.00) 296255 106647 7174 236.7
Exercise frequency: 1-2 times/week 0.87 (0.83, 0.91) 130907 37173 2171 165.6
Exercise frequency: 3-4 times/week 0.84 (0.79, 0.90) 60735 16199 1050 175.3
Exercise frequency: 5-6 times/week 0.78 (0.69, 0.88) 15420 4593 271 176.5
Exercise frequency: Almost everyday 0.95 (0.88, 1.01) 32380 10849 909 280.4
Hypertension
Exercise frequency: None (Reference) 1.00 (1.00, 1.00) 296255 106647 47918 2346.4
Exercise frequency: 1-2 times/week 0.89 (0.88, 0.91) 130907 37173 17968 2035.9
Exercise frequency: 3-4 times/week 0.82 (0.81, 0.84) 60735 16199 8086 1979.9
Exercise frequency: 5-6 times/week 0.86 (0.82, 0.89) 15420 4593 2223 2110.5
Exercise frequency: Almost everyday 0.93 (0.90, 0.95) 32380 10849 5556 2625.7
Type 2 diabetes
Exercise frequency: None (Reference) 1.00 (1.00, 1.00) 296255 106647 18858 677.3
Exercise frequency: 1-2 times/week 0.90 (0.88, 0.93) 130907 37173 7336 612.3
Exercise frequency: 3-4 times/week 0.86 (0.83, 0.89) 60735 16199 3377 614.8
Exercise frequency: 5-6 times/week 0.87 (0.81, 0.93) 15420 4593 889 633.9
Exercise frequency: Almost everyday 0.97 (0.93, 1.01) 32380 10849 2313 782.6
.3
1
1.3
Smoking: previous
HR (95% CI)
Total cases(N)
Unique participants(n)
Events(n)
Crude rate
1.00 (1.00, 1.00) 23702 8659 132 52.1
0.64 (0.48, 0.86) 28268 7571 75 25.7
0.72 (0.50, 1.03) 12863 3156 40 30.5
1.17 (0.71, 1.92) 3027 831 18 58.2
0.78 (0.49, 1.24) 4496 1337 21 44.5
1.00 (1.00, 1.00) 23702 8659 507 205.3
0.91 (0.79, 1.05) 28268 7571 363 126.4
0.77 (0.64, 0.93) 12863 3156 148 114.8
0.97 (0.74, 1.28) 3027 831 56 184.8
1.05 (0.85, 1.29) 4496 1337 112 245.4
1.00 (1.00, 1.00) 23702 8659 4168 2733.5
0.91 (0.87, 0.95) 28268 7571 3967 2282.3
0.88 (0.83, 0.93) 12863 3156 1791 2339.4
0.87 (0.79, 0.96) 3027 831 446 2396.9
0.96 (0.88, 1.04) 4496 1337 708 2629.5
1.00 (1.00, 1.00) 23702 8659 1691 776.7
0.90 (0.84, 0.97) 28268 7571 1685 669.5
0.90 (0.83, 0.98) 12863 3156 787 693.5
0.87 (0.75, 1.01) 3027 831 196 733
0.98 (0.87, 1.11) 4496 1337 322 793
1
1.3
Smoking: current
HR (95% CI)
Total cases(N)
Unique participants(n)
Events(n)
Crude rate
1.00 (1.00, 1.00) 83399 32978 598 66
0.94 (0.81, 1.08) 61426 18179 315 48.7
0.89 (0.72, 1.10) 19151 5481 99 49.3
1.28 (0.90, 1.82) 4010 1252 33 78
0.87 (0.67, 1.14) 8162 2949 59 67.4
1.00 (1.00, 1.00) 83399 32978 2008 227.3
0.82 (0.75, 0.89) 61426 18179 799 125.3
0.85 (0.75, 0.96) 19151 5481 284 143.8
0.79 (0.61, 1.01) 4010 1252 63 152.1
0.92 (0.80, 1.06) 8162 2949 219 258
1.00 (1.00, 1.00) 83399 32978 13878 2419.9
0.98 (0.95, 1.01) 61426 18179 8688 2216.5
0.97 (0.93, 1.01) 19151 5481 2740 2251.7
0.95 (0.88, 1.03) 4010 1252 625 2420.2
1.03 (0.97, 1.09) 8162 2949 1441 2780.7
1.00 (1.00, 1.00) 83399 32978 6579 857
0.95 (0.91, 0.98) 61426 18179 4205 767.5
0.87 (0.82, 0.93) 19151 5481 1257 733.8
0.96 (0.85, 1.07) 4010 1252 314 875.1
0.95 (0.88, 1.03) 8162 2949 650 890.9
1
1.3

review only

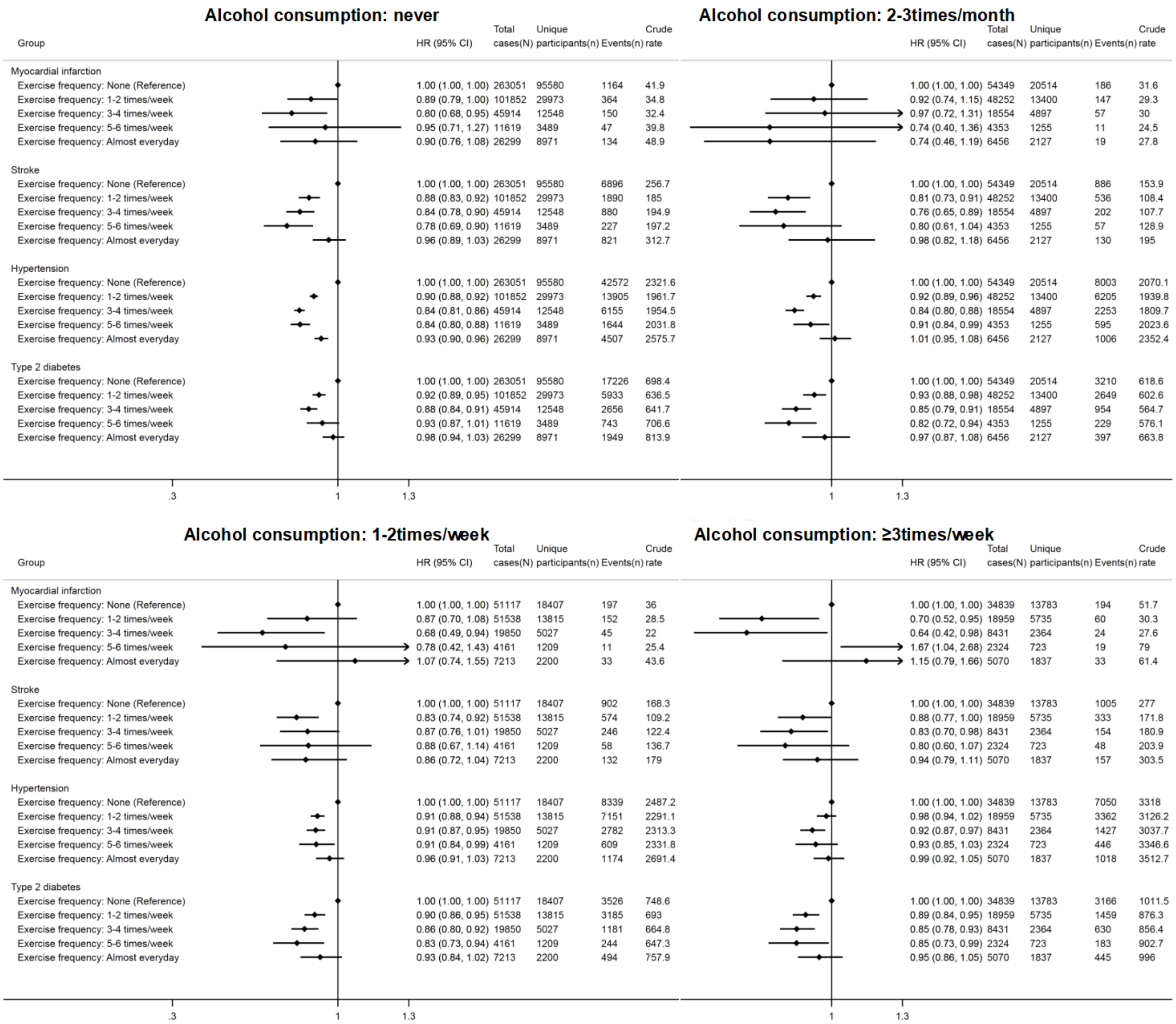
Alcohol consumption: never
Group
HR (95% CI)
Total cases(N)
Unique participants(n)
Events(n)
Crude rate
Myocardial infarction
Exercise frequency: None (Reference) 1.00 (1.00, 1.00) 263051 95580 1164 41.9
Exercise frequency: 1-2 times/week 0.89 (0.79, 1.00) 101852 29973 364 34.8
Exercise frequency: 3-4 times/week 0.80 (0.68, 0.95) 45914 12548 150 32.4
Exercise frequency: 5-6 times/week 0.95 (0.71, 1.27) 11619 3489 47 39.8
Exercise frequency: Almost everyday 0.90 (0.76, 1.08) 26299 8971 134 48.9
Stroke
Exercise frequency: None (Reference) 1.00 (1.00, 1.00) 263051 95580 6896 256.7
Exercise frequency: 1-2 times/week 0.88 (0.83, 0.92) 101852 29973 1890 185
Exercise frequency: 3-4 times/week 0.84 (0.78, 0.90) 45914 12548 880 194.9
Exercise frequency: 5-6 times/week 0.78 (0.69, 0.90) 11619 3489 227 197.2
Exercise frequency: Almost everyday 0.96 (0.89, 1.03) 26299 8971 821 312.7
Hypertension
Exercise frequency: None (Reference) 1.00 (1.00, 1.00) 263051 95580 42572 2321.6
Exercise frequency: 1-2 times/week 0.90 (0.88, 0.92) 101852 29973 13905 1961.7
Exercise frequency: 3-4 times/week 0.84 (0.81, 0.86) 45914 12548 6155 1954.5
Exercise frequency: 5-6 times/week 0.84 (0.80, 0.88) 11619 3489 1644 2031.8
Exercise frequency: Almost everyday 0.93 (0.90, 0.96) 26299 8971 4507 2575.7
Type 2 diabetes
Exercise frequency: None (Reference) 1.00 (1.00, 1.00) 263051 95580 17226 698.4
Exercise frequency: 1-2 times/week 0.92 (0.89, 0.95) 101852 29973 5933 636.5
Exercise frequency: 3-4 times/week 0.88 (0.84, 0.91) 45914 12548 2656 641.7
Exercise frequency: 5-6 times/week 0.93 (0.87, 1.01) 11619 3489 743 706.6
Exercise frequency: Almost everyday 0.98 (0.94, 1.03) 26299 8971 1949 813.9
.3
1
1.3
Alcohol consumption: 2-3times/month
HR (95% CI)
Total cases(N)
Unique participants(n)
Events(n)
Crude rate
1.00 (1.00, 1.00) 54349 20514 186 31.6
0.92 (0.74, 1.15) 48252 13400 147 29.3
0.97 (0.72, 1.31) 18554 4897 57 30
0.74 (0.40, 1.36) 4353 1255 11 24.5
0.74 (0.46, 1.19) 6456 2127 19 27.8
1.00 (1.00, 1.00) 54349 20514 886 153.9
0.81 (0.73, 0.91) 48252 13400 536 108.4
0.76 (0.65, 0.89) 18554 4897 202 107.7
0.80 (0.61, 1.04) 4353 1255 57 128.9
0.98 (0.82, 1.18) 6456 2127 130 195
1.00 (1.00, 1.00) 54349 20514 8003 2070.1
0.92 (0.89, 0.96) 48252 13400 6205 1939.8
0.84 (0.80, 0.88) 18554 4897 2253 1809.7
0.91 (0.84, 0.99) 4353 1255 595 2023.6
1.01 (0.95, 1.08) 6456 2127 1006 2352.4
1.00 (1.00, 1.00) 54349 20514 3210 618.6
0.93 (0.88, 0.98) 48252 13400 2649 602.6
0.85 (0.79, 0.91) 18554 4897 954 564.7
0.82 (0.72, 0.94) 4353 1255 229 576.1
0.97 (0.87, 1.08) 6456 2127 397 663.8
1
1.3
Alcohol consumption: 1-2times/week
Group
HR (95% CI)
Total cases(N)
Unique participants(n)
Events(n)
Crude rate
Myocardial infarction
Exercise frequency: None (Reference) 1.00 (1.00, 1.00) 51117 18407 197 36
Exercise frequency: 1-2 times/week 0.87 (0.70, 1.08) 51538 13815 152 28.5
Exercise frequency: 3-4 times/week 0.68 (0.49, 0.94) 19850 5027 45 22
Exercise frequency: 5-6 times/week 0.78 (0.42, 1.43) 4161 1209 11 25.4
Exercise frequency: Almost everyday 1.07 (0.74, 1.55) 7213 2200 33 43.6
Stroke
Exercise frequency: None (Reference) 1.00 (1.00, 1.00) 51117 18407 902 168.3
Exercise frequency: 1-2 times/week 0.83 (0.74, 0.92) 51538 13815 574 109.2
Exercise frequency: 3-4 times/week 0.87 (0.76, 1.01) 19850 5027 246 122.4
Exercise frequency: 5-6 times/week 0.88 (0.67, 1.14) 4161 1209 58 136.7
Exercise frequency: Almost everyday 0.86 (0.72, 1.04) 7213 2200 132 179
Hypertension
Exercise frequency: None (Reference) 1.00 (1.00, 1.00) 51117 18407 8339 2487.2
Exercise frequency: 1-2 times/week 0.91 (0.88, 0.94) 51538 13815 7151 2291.1
Exercise frequency: 3-4 times/week 0.91 (0.87, 0.95) 19850 5027 2782 2313.3
Exercise frequency: 5-6 times/week 0.91 (0.84, 0.99) 4161 1209 609 2331.8
Exercise frequency: Almost everyday 0.96 (0.91, 1.03) 7213 2200 1174 2691.4
Type 2 diabetes
Exercise frequency: None (Reference) 1.00 (1.00, 1.00) 51117 18407 3526 748.6
Exercise frequency: 1-2 times/week 0.90 (0.86, 0.95) 51538 13815 3185 693
Exercise frequency: 3-4 times/week 0.86 (0.80, 0.92) 19850 5027 1181 664.8
Exercise frequency: 5-6 times/week 0.83 (0.73, 0.94) 4161 1209 244 647.3
Exercise frequency: Almost everyday 0.93 (0.84, 1.02) 7213 2200 494 757.9
.3
1
1.3
Alcohol consumption: ≥3times/week
HR (95% CI)
Total cases(N)
Unique participants(n)
Events(n)
Crude rate
1.00 (1.00, 1.00) 34839 13783 194 51.7
0.70 (0.52, 0.95) 18959 5735 60 30.3
0.64 (0.42, 0.98) 8431 2364 24 27.6
1.67 (1.04, 2.68) 2324 723 19 79
1.15 (0.79, 1.66) 5070 1837 33 61.4
1.00 (1.00, 1.00) 34839 13783 1005 277
0.88 (0.77, 1.00) 18959 5735 333 171.8
0.83 (0.70, 0.98) 8431 2364 154 180.9
0.80 (0.60, 1.07) 2324 723 48 203.9
0.94 (0.79, 1.11) 5070 1837 157 303.5
1.00 (1.00, 1.00) 34839 13783 7050 3318
0.98 (0.94, 1.02) 18959 5735 3362 3126.2
0.92 (0.87, 0.97) 8431 2364 1427 3037.7
0.93 (0.85, 1.03) 2324 723 446 3346.6
0.99 (0.92, 1.05) 5070 1837 1018 3512.7
1.00 (1.00, 1.00) 34839 13783 3166 1011.5
0.89 (0.84, 0.95) 18959 5735 1459 876.3
0.85 (0.78, 0.93) 8431 2364 630 856.4
0.85 (0.73, 0.99) 2324 723 183 902.7
0.95 (0.86, 1.05) 5070 1837 445 996
1
1.3

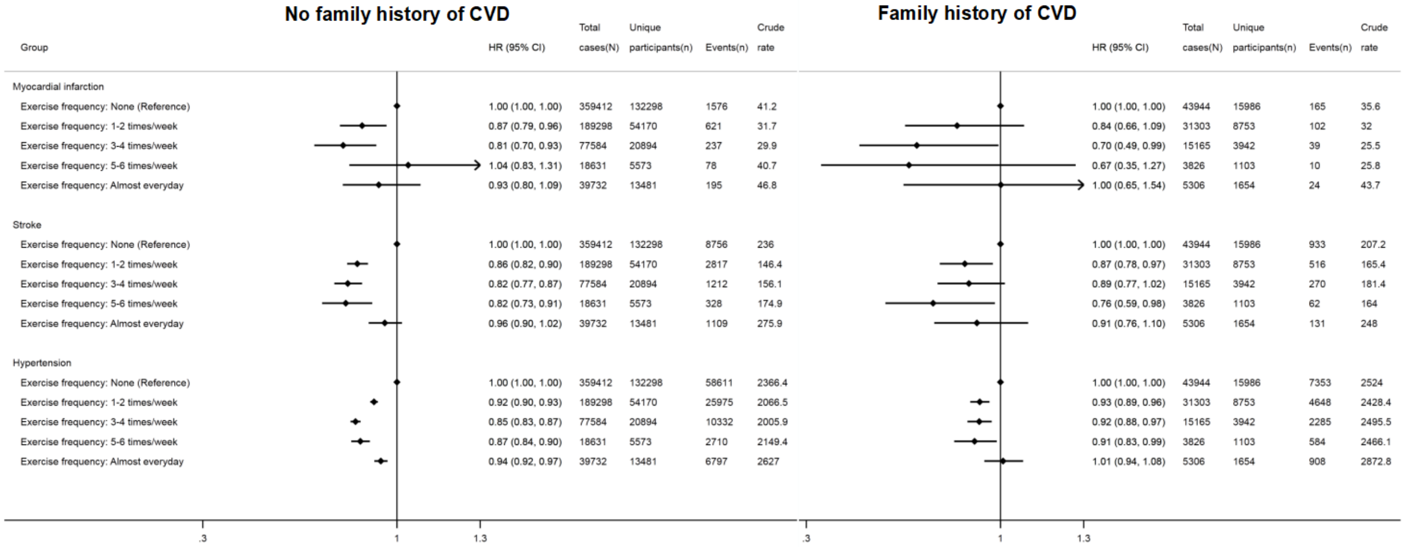

No family history of CVD

Group	HR (95% CI)	Total cases(N)	Unique participants(n)	Events(n)	Crude rate
Myocardial infarction					
Exercise frequency: None (Reference)	1.00 (1.00, 1.00)	359412	132298	1576	41.2
Exercise frequency: 1-2 times/week	0.87 (0.79, 0.96)	189298	54170	621	31.7
Exercise frequency: 3-4 times/week	0.81 (0.70, 0.93)	77584	20894	237	29.9
Exercise frequency: 5-6 times/week	1.04 (0.83, 1.31)	18631	5573	78	40.7
Exercise frequency: Almost everyday	0.93 (0.80, 1.09)	39732	13481	195	46.8
Stroke					
Exercise frequency: None (Reference)	1.00 (1.00, 1.00)	359412	132298	8756	236
Exercise frequency: 1-2 times/week	0.86 (0.82, 0.90)	189298	54170	2817	146.4
Exercise frequency: 3-4 times/week	0.82 (0.77, 0.87)	77584	20894	1212	156.1
Exercise frequency: 5-6 times/week	0.82 (0.73, 0.91)	18631	5573	328	174.9
Exercise frequency: Almost everyday	0.96 (0.90, 1.02)	39732	13481	1109	275.9
Hypertension					
Exercise frequency: None (Reference)	1.00 (1.00, 1.00)	359412	132298	58611	2366.4
Exercise frequency: 1-2 times/week	0.92 (0.90, 0.93)	189298	54170	25975	2066.5
Exercise frequency: 3-4 times/week	0.85 (0.83, 0.87)	77584	20894	10332	2005.9
Exercise frequency: 5-6 times/week	0.87 (0.84, 0.90)	18631	5573	2710	2149.4
Exercise frequency: Almost everyday	0.94 (0.92, 0.97)	39732	13481	6797	2627

Family history of CVD

Group	HR (95% CI)	Total cases(N)	Unique participants(n)	Events(n)	Crude rate
Myocardial infarction					
Exercise frequency: None (Reference)	1.00 (1.00, 1.00)	43944	15986	165	35.6
Exercise frequency: 1-2 times/week	0.84 (0.66, 1.09)	31303	8753	102	32
Exercise frequency: 3-4 times/week	0.70 (0.49, 0.99)	15165	3942	39	25.5
Exercise frequency: 5-6 times/week	0.67 (0.35, 1.27)	3826	1103	10	25.8
Exercise frequency: Almost everyday	1.00 (0.65, 1.54)	5306	1654	24	43.7
Stroke					
Exercise frequency: None (Reference)	1.00 (1.00, 1.00)	43944	15986	933	207.2
Exercise frequency: 1-2 times/week	0.87 (0.78, 0.97)	31303	8753	516	165.4
Exercise frequency: 3-4 times/week	0.89 (0.77, 1.02)	15165	3942	270	181.4
Exercise frequency: 5-6 times/week	0.76 (0.59, 0.98)	3826	1103	62	164
Exercise frequency: Almost everyday	0.91 (0.76, 1.10)	5306	1654	131	248
Hypertension					
Exercise frequency: None (Reference)	1.00 (1.00, 1.00)	43944	15986	7353	2524
Exercise frequency: 1-2 times/week	0.93 (0.89, 0.96)	31303	8753	4648	2428.4
Exercise frequency: 3-4 times/week	0.92 (0.88, 0.97)	15165	3942	2285	2495.5
Exercise frequency: 5-6 times/week	0.91 (0.83, 0.99)	3826	1103	584	2466.1
Exercise frequency: Almost everyday	1.01 (0.94, 1.08)	5306	1654	908	2872.8

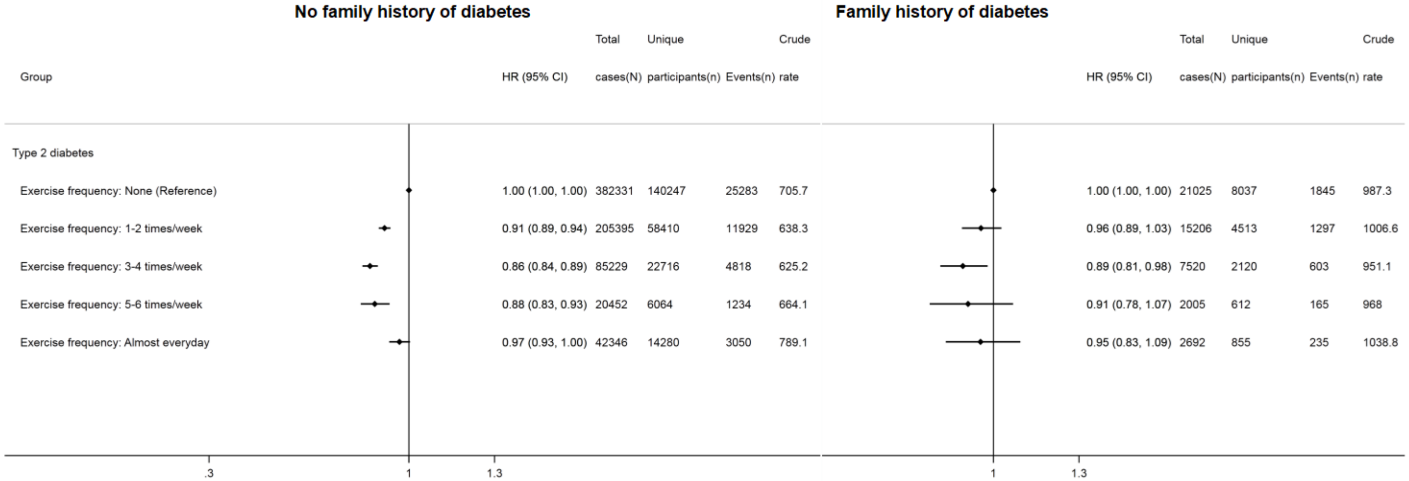

No family history of diabetes

Group	HR (95% CI)	Total cases(N)	Unique participants(n)	Events(n)	Crude rate
Type 2 diabetes					
Exercise frequency: None (Reference)	1.00 (1.00, 1.00)	382331	140247	25283	705.7
Exercise frequency: 1-2 times/week	0.91 (0.89, 0.94)	205395	58410	11929	638.3
Exercise frequency: 3-4 times/week	0.86 (0.84, 0.89)	85229	22716	4818	625.2
Exercise frequency: 5-6 times/week	0.88 (0.83, 0.93)	20452	6064	1234	664.1
Exercise frequency: Almost everyday	0.97 (0.93, 1.00)	42346	14280	3050	789.1

Family history of diabetes

Group	HR (95% CI)	Total cases(N)	Unique participants(n)	Events(n)	Crude rate
Type 2 diabetes					
Exercise frequency: None (Reference)	1.00 (1.00, 1.00)	21025	8037	1845	987.3
Exercise frequency: 1-2 times/week	0.96 (0.89, 1.03)	15206	4513	1297	1006.6
Exercise frequency: 3-4 times/week	0.89 (0.81, 0.98)	7520	2120	603	951.1
Exercise frequency: 5-6 times/week	0.91 (0.78, 1.07)	2005	612	165	968
Exercise frequency: Almost everyday	0.95 (0.83, 1.09)	2692	855	235	1038.8

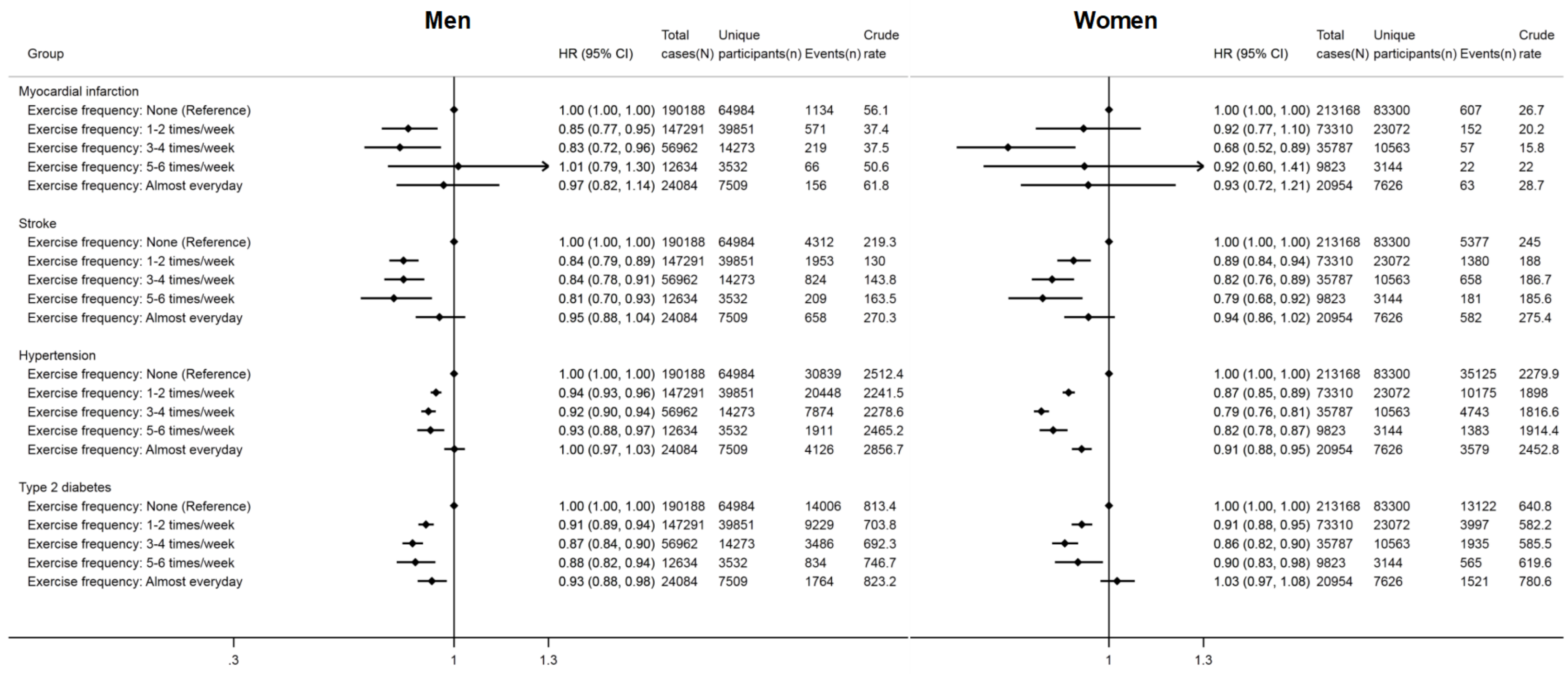

Men

Group	HR (95% CI)	Total cases(N)	Unique participants(n)	Events(n)	Crude rate
Myocardial infarction					
Exercise frequency: None (Reference)	1.00 (1.00, 1.00)	190188	64984	1134	56.1
Exercise frequency: 1-2 times/week	0.85 (0.77, 0.95)	147291	39851	571	37.4
Exercise frequency: 3-4 times/week	0.83 (0.72, 0.96)	56962	14273	219	37.5
Exercise frequency: 5-6 times/week	1.01 (0.79, 1.30)	12634	3532	66	50.6
Exercise frequency: Almost everyday	0.97 (0.82, 1.14)	24084	7509	156	61.8
Stroke					
Exercise frequency: None (Reference)	1.00 (1.00, 1.00)	190188	64984	4312	219.3
Exercise frequency: 1-2 times/week	0.84 (0.79, 0.89)	147291	39851	1953	130
Exercise frequency: 3-4 times/week	0.84 (0.78, 0.91)	56962	14273	824	143.8
Exercise frequency: 5-6 times/week	0.81 (0.70, 0.93)	12634	3532	209	163.5
Exercise frequency: Almost everyday	0.95 (0.88, 1.04)	24084	7509	658	270.3
Hypertension					
Exercise frequency: None (Reference)	1.00 (1.00, 1.00)	190188	64984	30839	2512.4
Exercise frequency: 1-2 times/week	0.94 (0.93, 0.96)	147291	39851	20448	2241.5
Exercise frequency: 3-4 times/week	0.92 (0.90, 0.94)	56962	14273	7874	2278.6
Exercise frequency: 5-6 times/week	0.93 (0.88, 0.97)	12634	3532	1911	2465.2
Exercise frequency: Almost everyday	1.00 (0.97, 1.03)	24084	7509	4126	2856.7
Type 2 diabetes					
Exercise frequency: None (Reference)	1.00 (1.00, 1.00)	190188	64984	14006	813.4
Exercise frequency: 1-2 times/week	0.91 (0.89, 0.94)	147291	39851	9229	703.8
Exercise frequency: 3-4 times/week	0.87 (0.84, 0.90)	56962	14273	3486	692.3
Exercise frequency: 5-6 times/week	0.88 (0.82, 0.94)	12634	3532	834	746.7
Exercise frequency: Almost everyday	0.93 (0.88, 0.98)	24084	7509	1764	823.2

Women

Group	HR (95% CI)	Total cases(N)	Unique participants(n)	Events(n)	Crude rate
Myocardial infarction					
Exercise frequency: None (Reference)	1.00 (1.00, 1.00)	213168	83300	607	26.7
Exercise frequency: 1-2 times/week	0.92 (0.77, 1.10)	73310	23072	152	20.2
Exercise frequency: 3-4 times/week	0.68 (0.52, 0.89)	35787	10563	57	15.8
Exercise frequency: 5-6 times/week	0.92 (0.60, 1.41)	9823	3144	22	22
Exercise frequency: Almost everyday	0.93 (0.72, 1.21)	20954	7626	63	28.7
Stroke					
Exercise frequency: None (Reference)	1.00 (1.00, 1.00)	213168	83300	5377	245
Exercise frequency: 1-2 times/week	0.89 (0.84, 0.94)	73310	23072	1380	188
Exercise frequency: 3-4 times/week	0.82 (0.76, 0.89)	35787	10563	658	186.7
Exercise frequency: 5-6 times/week	0.79 (0.68, 0.92)	9823	3144	181	185.6
Exercise frequency: Almost everyday	0.94 (0.86, 1.02)	20954	7626	582	275.4
Hypertension					
Exercise frequency: None (Reference)	1.00 (1.00, 1.00)	213168	83300	35125	2279.9
Exercise frequency: 1-2 times/week	0.87 (0.85, 0.89)	73310	23072	10175	1898
Exercise frequency: 3-4 times/week	0.79 (0.76, 0.81)	35787	10563	4743	1816.6
Exercise frequency: 5-6 times/week	0.82 (0.78, 0.87)	9823	3144	1383	1914.4
Exercise frequency: Almost everyday	0.91 (0.88, 0.95)	20954	7626	3579	2452.8
Type 2 diabetes					
Exercise frequency: None (Reference)	1.00 (1.00, 1.00)	213168	83300	13122	640.8
Exercise frequency: 1-2 times/week	0.91 (0.88, 0.95)	73310	23072	3997	582.2
Exercise frequency: 3-4 times/week	0.86 (0.82, 0.90)	35787	10563	1935	585.5
Exercise frequency: 5-6 times/week	0.90 (0.83, 0.98)	9823	3144	565	619.6
Exercise frequency: Almost everyday	1.03 (0.97, 1.08)	20954	7626	1521	780.6

Supplementary Figure 4. Results from running Cox regression models examining effect modification of sex, body mass index, smoking, alcohol consumption, and family history of disease in the associations between exercise frequency and incident myocardial infarction, stroke, hypertension and type 2 diabetes. Note: Cox regression models using age as the underlying timescale were adjusted for sex [not in models for effect modification by sex], body mass index [not in models for effect modification by body mass index], systolic blood pressure, fasting glucose levels, total cholesterol levels, family history [not in models for effect modification by family history of respective disease] of heart disease/stroke/hypertension (in models for myocardial infarction, stroke and hypertension), or diabetes (in models for Type 2 diabetes), smoking status [not in models for effect modification by smoking status] and alcohol consumption [not in models for effect modification by alcohol consumption]. Crude rates are per 100,000 person-years. “N” indicates numbers of total observations (i.e. participants who provided repeated measures are treated as separate observations) and “n” indicates numbers of unique participants at baseline. P-values for multiplicative terms – myocardial infarction (p-value = 0.235), stroke (p-value = 0.363), hypertension (p-value = 0.050) and type 2 diabetes (p-value = 0.196) by body mass index;

myocardial infarction (p-value = 0.643), stroke (p-value = 0.661), hypertension (p-value <0.001) and type 2 diabetes (p-value = 0.980) by smoking; myocardial infarction (p-value = 0.300), stroke (p-value = 0.607), hypertension (p-value <0.001) and type 2 diabetes (p-value = 0.081) by alcohol consumption; myocardial infarction (p-value = 0.590), stroke (p-value = 0.505), and hypertension (p-value = 0.029) by family history of cardiovascular disease; type 2 diabetes (p-value = 0.952) by family history of diabetes; and myocardial infarction (p-value = 0.334), stroke (p-value = 0.818), hypertension (p-value <0.001) and type 2 diabetes (p-value = 0.012) by sex.
Abbreviations: HR – Hazard Ratio; CI – Confidence Intervals

For peer review only

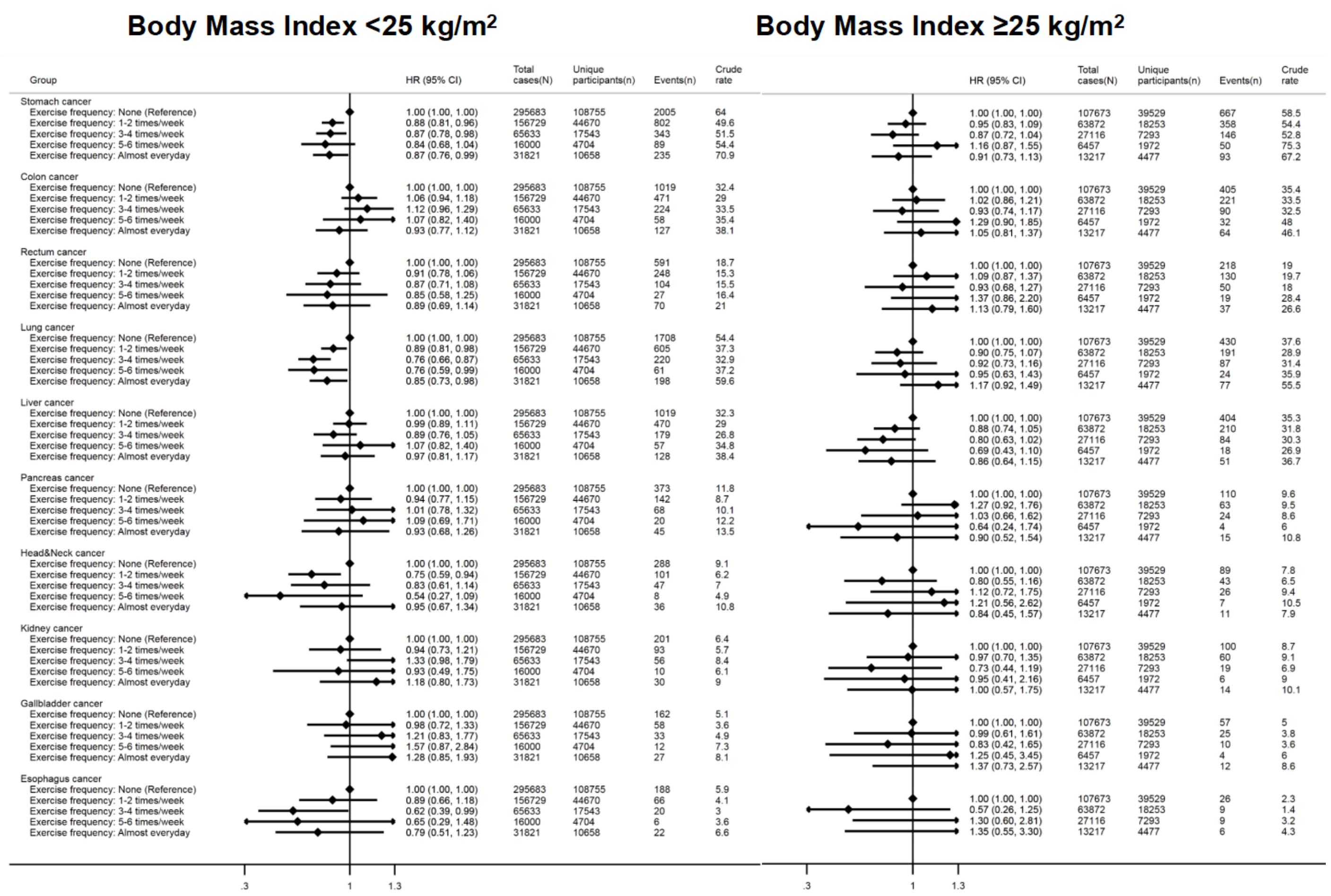
Body Mass Index <25 kg/m²
Group | HR (95% CI) | Total cases(N) | Unique participants(n) | Events(n) | Crude rate
Stomach cancer
Exercise frequency: None (Reference) 1.00 (1.00, 1.00) 295683 108755 2005 64
Exercise frequency: 1-2 times/week 0.88 (0.81, 0.96) 156729 44670 802 49.6
Exercise frequency: 3-4 times/week 0.87 (0.78, 0.98) 65633 17543 343 51.5
Exercise frequency: 5-6 times/week 0.84 (0.68, 1.04) 16000 4704 89 54.4
Exercise frequency: Almost everyday 0.87 (0.76, 0.99) 31821 10658 235 70.9
Colon cancer
Exercise frequency: None (Reference) 1.00 (1.00, 1.00) 295683 108755 1019 32.4
Exercise frequency: 1-2 times/week 1.06 (0.94, 1.18) 156729 44670 471 29
Exercise frequency: 3-4 times/week 1.12 (0.96, 1.29) 65633 17543 224 33.5
Exercise frequency: 5-6 times/week 1.07 (0.82, 1.40) 16000 4704 58 35.4
Exercise frequency: Almost everyday 0.93 (0.77, 1.12) 31821 10658 127 38.1
Rectum cancer
Exercise frequency: None (Reference) 1.00 (1.00, 1.00) 295683 108755 591 18.7
Exercise frequency: 1-2 times/week 0.91 (0.78, 1.06) 156729 44670 248 15.3
Exercise frequency: 3-4 times/week 0.87 (0.71, 1.08) 65633 17543 104 15.5
Exercise frequency: 5-6 times/week 0.85 (0.58, 1.25) 16000 4704 27 16.4
Exercise frequency: Almost everyday 0.89 (0.69, 1.14) 31821 10658 70 21
Lung cancer
Exercise frequency: None (Reference) 1.00 (1.00, 1.00) 295683 108755 1708 54.4
Exercise frequency: 1-2 times/week 0.89 (0.81, 0.98) 156729 44670 605 37.3
Exercise frequency: 3-4 times/week 0.76 (0.66, 0.87) 65633 17543 220 32.9
Exercise frequency: 5-6 times/week 0.76 (0.59, 0.99) 16000 4704 61 37.2
Exercise frequency: Almost everyday 0.85 (0.73, 0.98) 31821 10658 198 59.6
Liver cancer
Exercise frequency: None (Reference) 1.00 (1.00, 1.00) 295683 108755 1019 32.3
Exercise frequency: 1-2 times/week 0.99 (0.89, 1.11) 156729 44670 470 29
Exercise frequency: 3-4 times/week 0.89 (0.76, 1.05) 65633 17543 179 26.8
Exercise frequency: 5-6 times/week 1.07 (0.82, 1.40) 16000 4704 57 34.8
Exercise frequency: Almost everyday 0.97 (0.81, 1.17) 31821 10658 128 38.4
Pancreas cancer
Exercise frequency: None (Reference) 1.00 (1.00, 1.00) 295683 108755 373 11.8
Exercise frequency: 1-2 times/week 0.94 (0.77, 1.15) 156729 44670 142 8.7
Exercise frequency: 3-4 times/week 1.01 (0.78, 1.32) 65633 17543 68 10.1
Exercise frequency: 5-6 times/week 1.09 (0.69, 1.71) 16000 4704 20 12.2
Exercise frequency: Almost everyday 0.93 (0.68, 1.26) 31821 10658 45 13.5
Head&Neck cancer
Exercise frequency: None (Reference) 1.00 (1.00, 1.00) 295683 108755 288 9.1
Exercise frequency: 1-2 times/week 0.75 (0.59, 0.94) 156729 44670 101 6.2
Exercise frequency: 3-4 times/week 0.83 (0.61, 1.14) 65633 17543 47 7
Exercise frequency: 5-6 times/week 0.54 (0.27, 1.09) 16000 4704 8 4.9
Exercise frequency: Almost everyday 0.95 (0.67, 1.34) 31821 10658 36 10.8
Kidney cancer
Exercise frequency: None (Reference) 1.00 (1.00, 1.00) 295683 108755 201 6.4
Exercise frequency: 1-2 times/week 0.94 (0.73, 1.21) 156729 44670 93 5.7
Exercise frequency: 3-4 times/week 1.33 (0.98, 1.79) 65633 17543 56 8.4
Exercise frequency: 5-6 times/week 0.93 (0.49, 1.75) 16000 4704 10 6.1
Exercise frequency: Almost everyday 1.18 (0.80, 1.73) 31821 10658 30 9
Gallbladder cancer
Exercise frequency: None (Reference) 1.00 (1.00, 1.00) 295683 108755 162 5.1
Exercise frequency: 1-2 times/week 0.98 (0.72, 1.33) 156729 44670 58 3.6
Exercise frequency: 3-4 times/week 1.21 (0.83, 1.77) 65633 17543 33 4.9
Exercise frequency: 5-6 times/week 1.57 (0.87, 2.84) 16000 4704 12 7.3
Exercise frequency: Almost everyday 1.28 (0.85, 1.93) 31821 10658 27 8.1
Esophagus cancer
Exercise frequency: None (Reference) 1.00 (1.00, 1.00) 295683 108755 188 5.9
Exercise frequency: 1-2 times/week 0.89 (0.66, 1.18) 156729 44670 66 4.1
Exercise frequency: 3-4 times/week 0.62 (0.39, 0.99) 65633 17543 20 3
Exercise frequency: 5-6 times/week 0.65 (0.29, 1.48) 16000 4704 6 3.6
Exercise frequency: Almost everyday 0.79 (0.51, 1.23) 31821 10658 22 6.6
.3 1 1.3
Body Mass Index ≥25 kg/m²
HR (95% CI) | Total cases(N) | Unique participants(n) | Events(n) | Crude rate
1.00 (1.00, 1.00) 107673 39529 667 58.5
0.95 (0.83, 1.09) 63872 18253 358 54.4
0.87 (0.72, 1.04) 27116 7293 146 52.8
1.16 (0.87, 1.55) 6457 1972 50 75.3
0.91 (0.73, 1.13) 13217 4477 93 67.2
1.00 (1.00, 1.00) 107673 39529 405 35.4
1.02 (0.86, 1.21) 63872 18253 221 33.5
0.93 (0.74, 1.17) 27116 7293 90 32.5
1.29 (0.90, 1.85) 6457 1972 32 48
1.05 (0.81, 1.37) 13217 4477 64 46.1
1.00 (1.00, 1.00) 107673 39529 218 19
1.09 (0.87, 1.37) 63872 18253 130 19.7
0.93 (0.68, 1.27) 27116 7293 50 18
1.37 (0.86, 2.20) 6457 1972 19 28.4
1.13 (0.79, 1.60) 13217 4477 37 26.6
1.00 (1.00, 1.00) 107673 39529 430 37.6
0.90 (0.75, 1.07) 63872 18253 191 28.9
0.92 (0.73, 1.16) 27116 7293 87 31.4
0.95 (0.63, 1.43) 6457 1972 24 35.9
1.17 (0.92, 1.49) 13217 4477 77 55.5
1.00 (1.00, 1.00) 107673 39529 404 35.3
0.88 (0.74, 1.05) 63872 18253 210 31.8
0.80 (0.63, 1.02) 27116 7293 84 30.3
0.69 (0.43, 1.10) 6457 1972 18 26.9
0.86 (0.64, 1.15) 13217 4477 51 36.7
1.00 (1.00, 1.00) 107673 39529 110 9.6
1.27 (0.92, 1.76) 63872 18253 63 9.5
1.03 (0.66, 1.62) 27116 7293 24 8.6
0.64 (0.24, 1.74) 6457 1972 4 6
0.90 (0.52, 1.54) 13217 4477 15 10.8
1.00 (1.00, 1.00) 107673 39529 89 7.8
0.80 (0.55, 1.16) 63872 18253 43 6.5
1.12 (0.72, 1.75) 27116 7293 26 9.4
1.21 (0.56, 2.62) 6457 1972 7 10.5
0.84 (0.45, 1.57) 13217 4477 11 7.9
1.00 (1.00, 1.00) 107673 39529 100 8.7
0.97 (0.70, 1.35) 63872 18253 60 9.1
0.73 (0.44, 1.19) 27116 7293 19 6.9
0.95 (0.41, 2.16) 6457 1972 6 9
1.00 (0.57, 1.75) 13217 4477 14 10.1
1.00 (1.00, 1.00) 107673 39529 57 5
0.99 (0.61, 1.61) 63872 18253 25 3.8
0.83 (0.42, 1.65) 27116 7293 10 3.6
1.25 (0.45, 3.45) 6457 1972 4 6
1.37 (0.73, 2.57) 13217 4477 12 8.6
1.00 (1.00, 1.00) 107673 39529 26 2.3
0.57 (0.26, 1.25) 63872 18253 9 1.4
1.30 (0.60, 2.81) 27116 7293 9 3.2
1.35 (0.55, 3.30) 13217 4477 6 4.3
.3 1 1.3

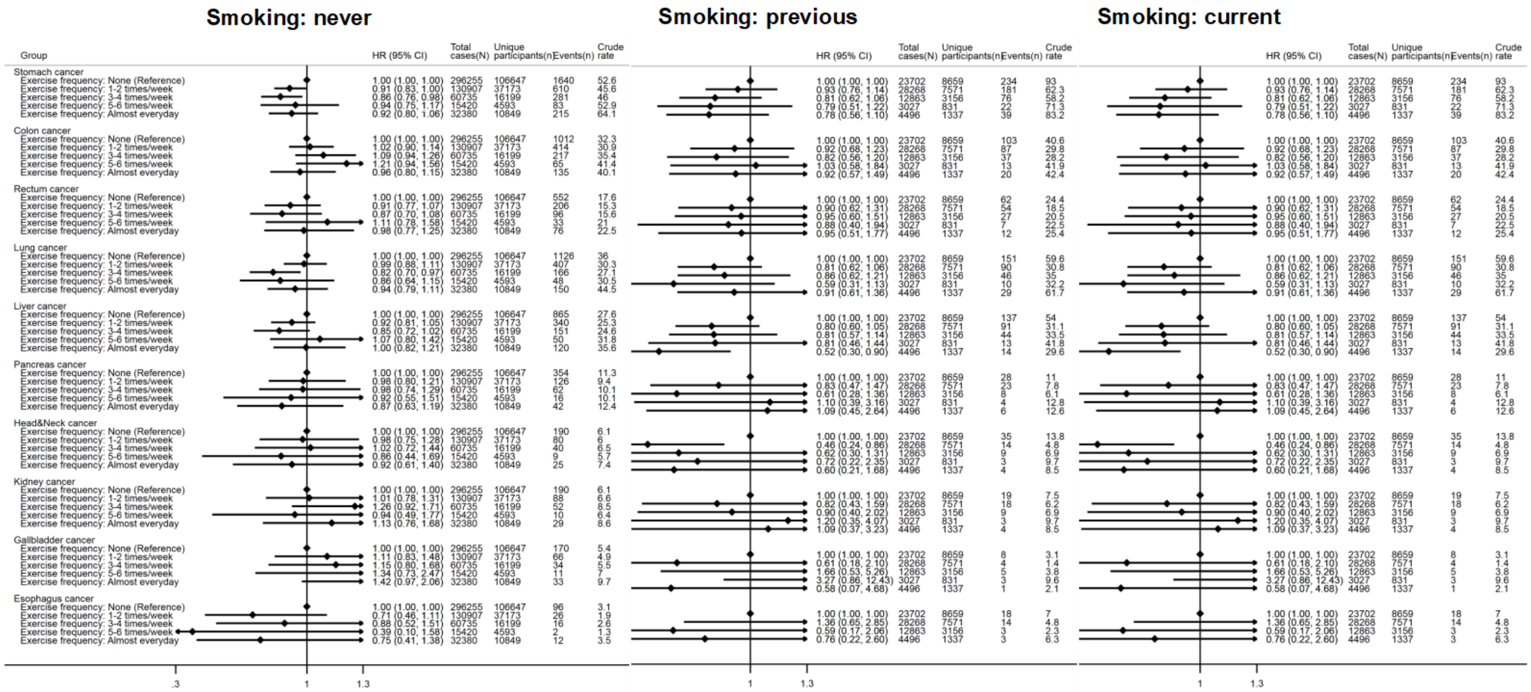

For peer review only - http://bmjopen.bmj.com/site/about/guidelines.xhtml

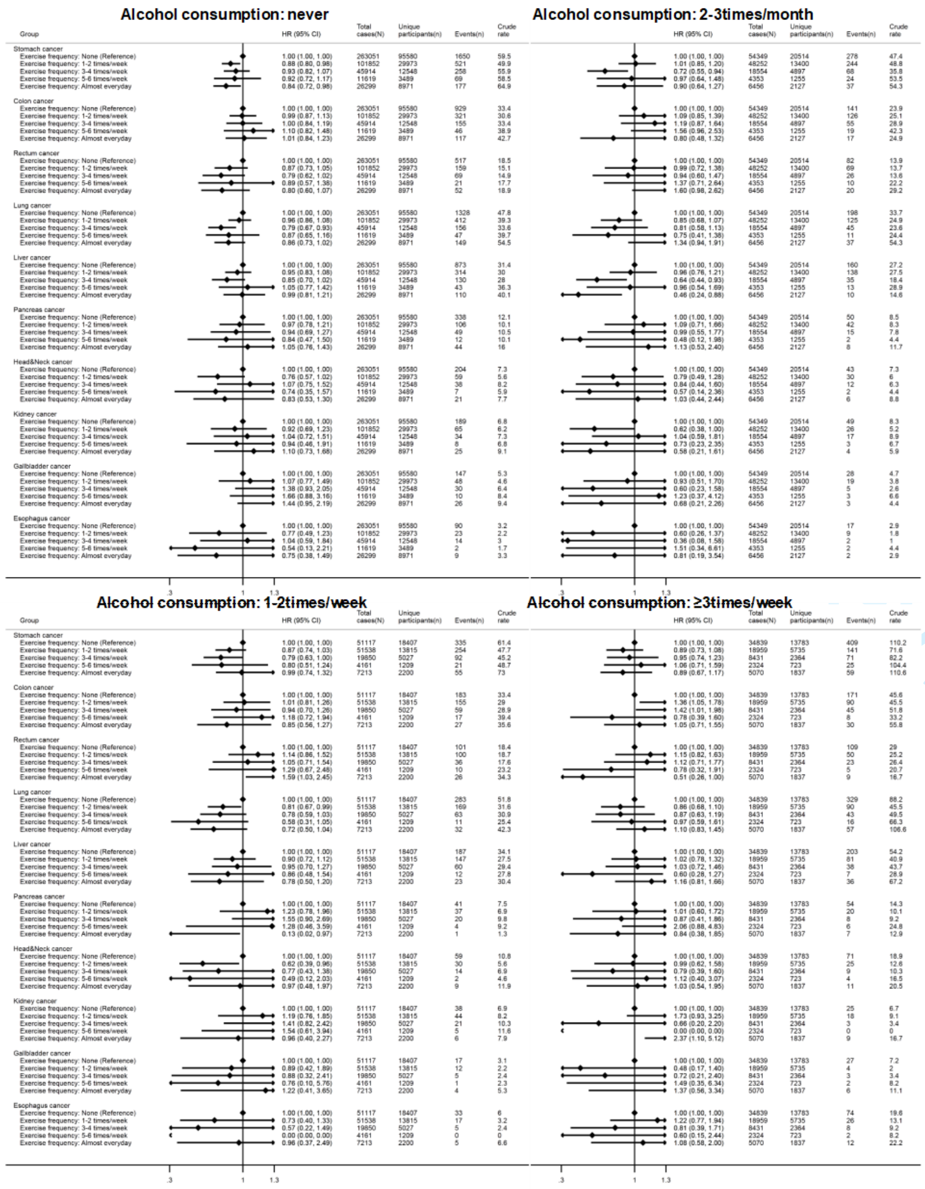
Alcohol consumption: never
Alcohol consumption: 2-3times/month
Alcohol consumption: 1-2times/week
Alcohol consumption: ≥3times/week

No family history of cancer

Group	HR (95% CI)	Total cases(N)	Unique participants(n)	Events(n)	Crude rate
**Stomach cancer**					
Exercise frequency: None (Reference)	1.00 (1.00, 1.00)	351171	128853	2325	62.4
Exercise frequency: 1-2 times/week	0.89 (0.82, 0.96)	186251	53323	937	48.7
Exercise frequency: 3-4 times/week	0.84 (0.76, 0.94)	76877	20575	384	49.1
Exercise frequency: 5-6 times/week	0.92 (0.76, 1.11)	18364	5571	111	58.8
Exercise frequency: Almost everyday	0.89 (0.79, 1.01)	38451	12944	282	70.2
**Colon cancer**					
Exercise frequency: None (Reference)	1.00 (1.00, 1.00)	351171	128853	1247	33.3
Exercise frequency: 1-2 times/week	1.06 (0.96, 1.17)	186251	53323	583	30.2
Exercise frequency: 3-4 times/week	1.01 (0.88, 1.16)	76877	20575	247	31.5
Exercise frequency: 5-6 times/week	1.14 (0.90, 1.45)	18364	5571	74	39.1
Exercise frequency: Almost everyday	1.01 (0.86, 1.18)	38451	12944	171	42.4
**Rectum cancer**					
Exercise frequency: None (Reference)	1.00 (1.00, 1.00)	351171	128853	715	19.1
Exercise frequency: 1-2 times/week	0.98 (0.85, 1.12)	186251	53323	327	16.9
Exercise frequency: 3-4 times/week	0.85 (0.70, 1.03)	76877	20575	124	15.8
Exercise frequency: 5-6 times/week	1.02 (0.74, 1.41)	18364	5571	39	20.6
Exercise frequency: Almost everyday	0.99 (0.80, 1.23)	38451	12944	96	23.7
**Lung cancer**					
Exercise frequency: None (Reference)	1.00 (1.00, 1.00)	351171	128853	1908	51.1
Exercise frequency: 1-2 times/week	0.92 (0.84, 1.01)	186251	53323	689	35.7
Exercise frequency: 3-4 times/week	0.81 (0.71, 0.92)	76877	20575	258	32.9
Exercise frequency: 5-6 times/week	0.80 (0.63, 1.02)	18364	5571	70	36.9
Exercise frequency: Almost everyday	0.94 (0.82, 1.08)	38451	12944	243	60.3
**Liver cancer**					
Exercise frequency: None (Reference)	1.00 (1.00, 1.00)	351171	128853	1271	34
Exercise frequency: 1-2 times/week	0.96 (0.86, 1.06)	186251	53323	588	30.4
Exercise frequency: 3-4 times/week	0.88 (0.77, 1.02)	76877	20575	231	29.4
Exercise frequency: 5-6 times/week	0.91 (0.71, 1.18)	18364	5571	62	32.7
Exercise frequency: Almost everyday	0.94 (0.80, 1.11)	38451	12944	159	39.4
**Pancreas cancer**					
Exercise frequency: None (Reference)	1.00 (1.00, 1.00)	351171	128853	415	11.1
Exercise frequency: 1-2 times/week	1.06 (0.88, 1.28)	186251	53323	171	8.8
Exercise frequency: 3-4 times/week	1.12 (0.88, 1.43)	76877	20575	80	10.2
Exercise frequency: 5-6 times/week	1.03 (0.66, 1.62)	18364	5571	20	10.5
Exercise frequency: Almost everyday	0.97 (0.73, 1.30)	38451	12944	53	13.1
**Head&Neck cancer**					
Exercise frequency: None (Reference)	1.00 (1.00, 1.00)	351171	128853	338	9
Exercise frequency: 1-2 times/week	0.77 (0.62, 0.95)	186251	53323	125	6.5
Exercise frequency: 3-4 times/week	0.88 (0.66, 1.16)	76877	20575	59	7.5
Exercise frequency: 5-6 times/week	0.57 (0.31, 1.08)	18364	5571	10	5.3
Exercise frequency: Almost everyday	0.95 (0.69, 1.31)	38451	12944	43	10.6
**Kidney cancer**					
Exercise frequency: None (Reference)	1.00 (1.00, 1.00)	351171	128853	264	7.1
Exercise frequency: 1-2 times/week	0.89 (0.71, 1.11)	186251	53323	120	6.2
Exercise frequency: 3-4 times/week	1.18 (0.90, 1.55)	76877	20575	66	8.4
Exercise frequency: 5-6 times/week	1.00 (0.59, 1.72)	18364	5571	14	7.4
Exercise frequency: Almost everyday	1.16 (0.83, 1.63)	38451	12944	39	9.7
**Gallbladder cancer**					
Exercise frequency: None (Reference)	1.00 (1.00, 1.00)	351171	128853	200	5.3
Exercise frequency: 1-2 times/week	0.91 (0.69, 1.22)	186251	53323	67	3.5
Exercise frequency: 3-4 times/week	1.11 (0.78, 1.59)	76877	20575	37	4.7
Exercise frequency: 5-6 times/week	1.63 (0.96, 2.77)	18364	5571	15	7.9
Exercise frequency: Almost everyday	1.25 (0.86, 1.81)	38451	12944	33	8.1
**Esophagus cancer**					
Exercise frequency: None (Reference)	1.00 (1.00, 1.00)	351171	128853	193	5.1
Exercise frequency: 1-2 times/week	0.86 (0.64, 1.15)	186251	53323	64	3.3
Exercise frequency: 3-4 times/week	0.82 (0.54, 1.25)	76877	20575	26	3.3
Exercise frequency: 5-6 times/week	0.68 (0.30, 1.53)	18364	5571	6	3.2
Exercise frequency: Almost everyday	0.98 (0.65, 1.47)	38451	12944	27	6.7

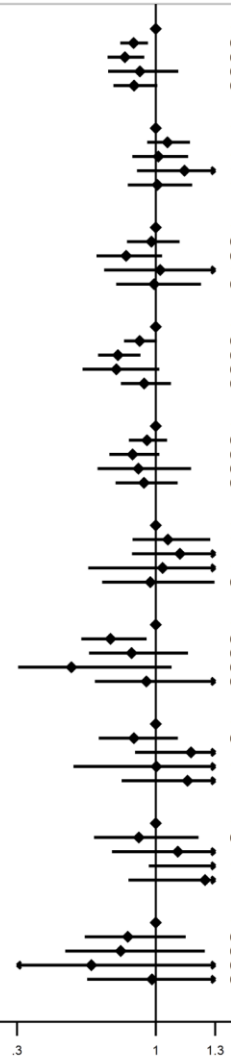

Family history of cancer

Group	HR (95% CI)	Total cases(N)	Unique participants(n)	Events(n)	Crude rate
**Stomach cancer**					
Exercise frequency: None (Reference)	1.00 (1.00, 1.00)	52185	19431	347	63.2
Exercise frequency: 1-2 times/week	1.00 (0.84, 1.18)	34350	9600	223	64
Exercise frequency: 3-4 times/week	1.00 (0.80, 1.25)	15872	4261	105	65.8
Exercise frequency: 5-6 times/week	0.99 (0.68, 1.46)	4093	1105	28	68.2
Exercise frequency: Almost everyday	0.82 (0.60, 1.11)	6587	2191	46	67.8
**Colon cancer**					
Exercise frequency: None (Reference)	1.00 (1.00, 1.00)	52185	19431	177	32.1
Exercise frequency: 1-2 times/week	0.99 (0.77, 1.26)	34350	9600	109	31.1
Exercise frequency: 3-4 times/week	1.26 (0.94, 1.67)	15872	4261	67	41.9
Exercise frequency: 5-6 times/week	1.11 (0.67, 1.86)	4093	1105	16	38.8
Exercise frequency: Almost everyday	0.72 (0.45, 1.15)	6587	2191	20	29.3
**Rectum cancer**					
Exercise frequency: None (Reference)	1.00 (1.00, 1.00)	52185	19431	94	17
Exercise frequency: 1-2 times/week	0.85 (0.60, 1.21)	34350	9600	51	14.5
Exercise frequency: 3-4 times/week	1.02 (0.67, 1.54)	15872	4261	30	18.7
Exercise frequency: 5-6 times/week	0.88 (0.41, 1.90)	4093	1105	7	16.9
Exercise frequency: Almost everyday	0.68 (0.36, 1.28)	6587	2191	11	16.1
**Lung cancer**					
Exercise frequency: None (Reference)	1.00 (1.00, 1.00)	52185	19431	230	41.7
Exercise frequency: 1-2 times/week	0.78 (0.61, 0.98)	34350	9600	107	30.5
Exercise frequency: 3-4 times/week	0.77 (0.56, 1.05)	15872	4261	49	30.6
Exercise frequency: 5-6 times/week	0.87 (0.51, 1.47)	4093	1105	15	36.2
Exercise frequency: Almost everyday	0.84 (0.58, 1.23)	6587	2191	32	46.9
**Liver cancer**					
Exercise frequency: None (Reference)	1.00 (1.00, 1.00)	52185	19431	152	27.5
Exercise frequency: 1-2 times/week	0.92 (0.71, 1.20)	34350	9600	92	26.2
Exercise frequency: 3-4 times/week	0.69 (0.47, 1.01)	15872	4261	32	19.9
Exercise frequency: 5-6 times/week	1.06 (0.60, 1.87)	4093	1105	13	31.4
Exercise frequency: Almost everyday	0.83 (0.52, 1.32)	6587	2191	20	29.2
**Pancreas cancer**					
Exercise frequency: None (Reference)	1.00 (1.00, 1.00)	52185	19431	68	12.3
Exercise frequency: 1-2 times/week	0.81 (0.53, 1.24)	34350	9600	34	9.7
Exercise frequency: 3-4 times/week	0.58 (0.31, 1.08)	15872	4261	12	7.5
Exercise frequency: 5-6 times/week	0.70 (0.25, 1.92)	4093	1105	4	9.6
Exercise frequency: Almost everyday	0.62 (0.29, 1.36)	6587	2191	7	10.2
**Head&Neck cancer**					
Exercise frequency: None (Reference)	1.00 (1.00, 1.00)	52185	19431	39	7.1
Exercise frequency: 1-2 times/week	0.72 (0.41, 1.26)	34350	9600	19	5.4
Exercise frequency: 3-4 times/week	1.18 (0.64, 2.19)	15872	4261	14	8.7
Exercise frequency: 5-6 times/week	1.59 (0.63, 4.06)	4093	1105	5	12.1
Exercise frequency: Almost everyday	0.72 (0.26, 2.03)	6587	2191	4	5.8
**Kidney cancer**					
Exercise frequency: None (Reference)	1.00 (1.00, 1.00)	52185	19431	37	6.7
Exercise frequency: 1-2 times/week	1.27 (0.79, 2.06)	34350	9600	33	9.4
Exercise frequency: 3-4 times/week	0.72 (0.35, 1.51)	15872	4261	9	5.6
Exercise frequency: 5-6 times/week	0.61 (0.15, 2.55)	4093	1105	2	4.8
Exercise frequency: Almost everyday	0.82 (0.32, 2.10)	6587	2191	5	7.3
**Gallbladder cancer**					
Exercise frequency: None (Reference)	1.00 (1.00, 1.00)	52185	19431	19	3.4
Exercise frequency: 1-2 times/week	1.44 (0.73, 2.84)	34350	9600	16	4.5
Exercise frequency: 3-4 times/week	1.06 (0.42, 2.68)	15872	4261	6	3.7
Exercise frequency: 5-6 times/week	0.61 (0.08, 4.60)	4093	1105	1	2.4
Exercise frequency: Almost everyday	1.82 (0.72, 4.60)	6587	2191	6	8.7
**Esophagus cancer**					
Exercise frequency: None (Reference)	1.00 (1.00, 1.00)	52185	19431	21	3.8
Exercise frequency: 1-2 times/week	0.79 (0.38, 1.68)	34350	9600	11	3.1
Exercise frequency: 3-4 times/week	0.51 (0.15, 1.74)	15872	4261	3	1.9
Exercise frequency: 5-6 times/week	0.00 (0.00, 0.00)	4093	1105	0	0
Exercise frequency: Almost everyday	0.27 (0.04, 2.03)	6587	2191	1	1.5

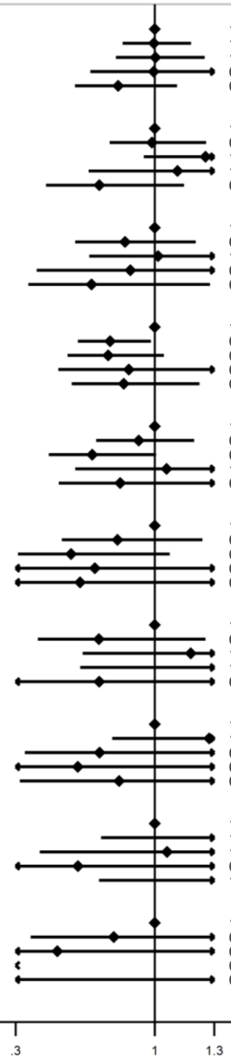

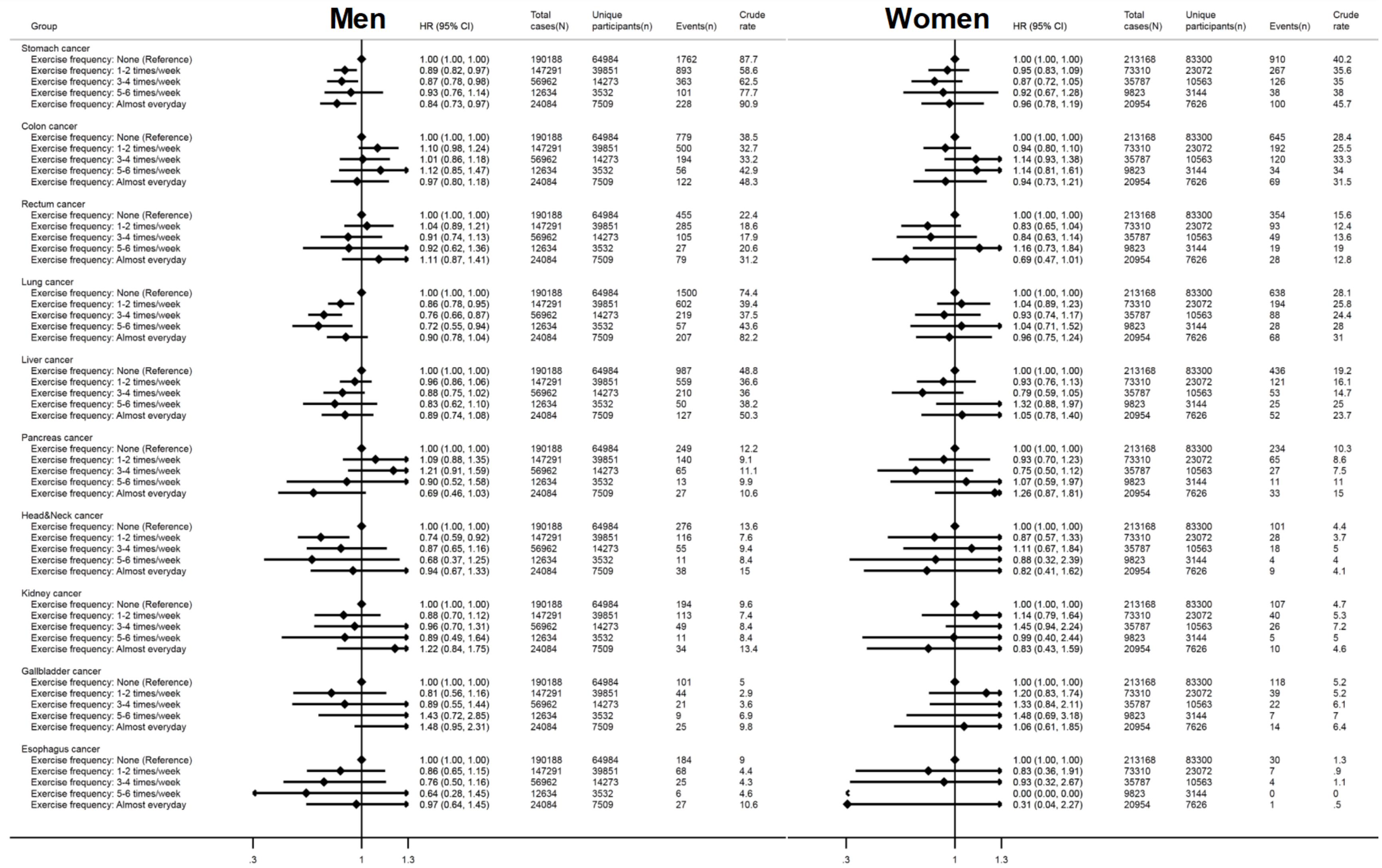

Supplementary Figure 5. Results from running Cox regression models examining effect modification of sex, body mass index, smoking, alcohol consumption, and family history of disease in the associations between exercise frequency and various incident cancer outcomes. Note: Cox regression models using age as the underlying timescale were adjusted for sex [not in models for effect

modification by sex], body mass index [not in models for effect modification by body mass index], systolic blood pressure, fasting glucose levels, total cholesterol levels, family history of cancer [not in models for effect modification by family history of cancer], smoking status [not in models for effect modification by smoking status] and alcohol consumption [not in models for effect modification by alcohol consumption]. Crude rates are per 100,000 person-years. “N” indicates numbers of total observations (i.e. participants who provided repeated measures are treated as separate observations) and “n” indicates numbers of unique participants at baseline. P-values for multiplicative terms – stomach cancer (p-value = 0.274), colon cancer (p-value = 0.640), rectum cancer (p-value = 0.107), lung cancer (p-value = 0.016), liver cancer (p-value = 0.129), pancreas cancer (p-value = 0.860), head & neck cancer (p-value = 0.488), kidney cancer (p-value = 0.285), gallbladder cancer (p-value = 0.970), and esophagus cancer (p-value = 0.263) by body mass index; stomach cancer (p-value = 0.699), colon cancer (p-value = 0.932), rectum cancer (p-value = 0.610), lung cancer (p-value = 0.492), liver cancer (p-value = 0.405), pancreas cancer (p-value = 0.338), head & neck cancer (p-value = 0.562), kidney cancer (p-value = 0.280), gallbladder cancer (p-value = 0.295), and esophagus cancer (p-value = 0.292) by smoking ; stomach cancer (p-value = 0.655), colon cancer (p-value = 0.977), rectum cancer (p-value = 0.433), lung cancer (p-value = 0.387), liver cancer (p-value = 0.704), pancreas cancer (p-value = 0.711), head & neck cancer (p-value = 1.000), kidney cancer (p-value = 0.336), gallbladder cancer (p-value = 0.350), and esophagus cancer (p-value = 0.550) by alcohol consumption ; stomach cancer (p-value = 0.996), colon cancer (p-value = 0.399), rectum cancer (p-value = 0.478), lung cancer (p-value = 0.427), liver cancer (p-value = 0.337), pancreas cancer (p-value = 0.086), head & neck cancer (p-value = 0.712), kidney cancer (p-value = 0.319), gallbladder cancer (p-value = 0.766), and esophagus cancer (p-value = 0.098) by family history of cancer; and stomach cancer (p-value = 0.405), colon cancer (p-value = 0.957), rectum cancer (p-value = 0.106), lung cancer (p-value = 0.063), liver cancer (p-value = 0.278), pancreas cancer (p-value = 0.265), head & neck cancer (p-value = 0.907), kidney cancer (p-value = 0.967), gallbladder cancer (p-value = 0.548), and esophagus cancer (p-value = 0.256) by sex. Abbreviations: HR – Hazard Ratio; CI – Confidence Intervals

For peer review only

BMJ Open

Exercise and incidence of myocardial infarction, stroke, hypertension, type 2 diabetes and site-specific cancers: A prospective cohort study of 257,854 adults in South Korea.

Journal:	*BMJ Open*
Manuscript ID	bmjopen-2018-025590.R2
Article Type:	Research
Date Submitted by the Author:	05-Dec-2018
Complete List of Authors:	Kim, Youngwon; University of Utah, Department of Health, Kinesiology, and Recreation; University of Cambridge School of Clinical Medicine, MRC Epidemiology Unit<br>Sharp, Stephen; University of Cambridge, MRC Epidemiology Unit<br>Hwang, Se-mi; Yonsei University, Department of Epidemiology and Health Promotion, Graduate School of Public Health<br>Jee, Sun Ha; Graduate School of Public Health, Yonsei University, Epidemiology
**Primary Subject Heading**:	Epidemiology
Secondary Subject Heading:	Public health
Keywords:	exercise, non-communicable disease, cohort, Epidemiology < TROPICAL MEDICINE, cardiovascular disease, Hypertension < CARDIOLOGY
Note: The following files were submitted by the author for peer review, but cannot be converted to PDF. You must view these files (e.g. movies) online.	
Figure 1 jpg.jpg	

SCHOLARONE™
Manuscripts

Title: Exercise and incidence of myocardial infarction, stroke, hypertension, type 2 diabetes and site-specific cancers: A prospective cohort study of 257,854 adults in South Korea.

Authors: Youngwon Kim,[1,2] Stephen J. Sharp,[2] Se-mi Hwang,[1,3] Sun Ha Jee[3]

Affiliations:

[1] Department of Health, Kinesiology, and Recreation, College of Health, University of Utah, UT, USA

[2] MRC Epidemiology Unit, University of Cambridge School of Clinical Medicine, Cambridge, UK

[3] Institute for Health Promotion, Department of Epidemiology and Health Promotion, Graduate School of Public Health, Yonsei University, Seoul, Republic of Korea.

Full Addresses:

[1] Department of Health, Kinesiology, and Recreation, College of Health, University of Utah. 250 South 1850 East Room 204, Salt Lake City, UT, USA, 84112

[2] MRC Epidemiology Unit, University of Cambridge School of Clinical Medicine, Box 285 Institute of Metabolic Science, Cambridge Biomedical Campus, Cambridge, Cambridgeshire, UK CB2 0QQ

[3] Department of Epidemiology and Health Promotion, Graduate School of Public Health, Yonsei University, 50-1 Yonsei-ro, Seodaemun-gu, Seoul, Korea 03722

Corresponding Author:

Name: Sun Ha Jee, Ph.D

Mailing Address: Department of Epidemiology and Health Promotion, Graduate School of Public Health, Yonsei University, 50-1 Yonsei-ro, Seodaemun-gu, Seoul, Korea 03722

E-mail Information: jsunha@yuhs.ac

Telephone: +82-2-2228-1523

Fax: +82-2-365-5118

Word count for Abstract: 250

Word count for Main text: 2787

For peer review only

Abstract

Objective: The objective of this study was to examine the longitudinal associations of exercise frequency with the incidence of myocardial infarction, stroke, hypertension, type 2 diabetes and 10 different cancer outcomes.

Design: A prospective cohort study.

Setting: Physical examination data linked with the entire South Korean population's health insurance system: from 2002 to 2015

Participants: 257,854 South Korean adults who provided up to 7 repeat-measures of exercise (defined as exercises causing sweat) and confounders.

Primary outcome measures: Each disease incidence was defined using both fatal and non-fatal health records (a median follow-up period of 13 years).

Results: Compared with no exercise category, the middle categories of exercise frequency (3-4 or 5-6 times/week) showed the lowest risk of myocardial infarction (hazard ratio[HR]: 0.79; 95% confidence interval[CI]: 0.70-0.90), stroke (HR: 0.80; 95%CI: 0.73-0.89), hypertension (HR: 0.86; 95%CI: 0.85-0.88), type 2 diabetes (HR: 0.87; 95%CI: 0.84-0.89), stomach (HR: 0.87; 95%CI: 0.79-0.96), lung (HR: 0.80; 95%CI: 0.71-0.91), liver (HR: 0.85; 95%CI: 0.75-0.98) and head & neck cancers (HR: 0.76; 95%CI: 0.63-0.93; for 1-2 times/week), exhibiting J-shaped associations. There was, in general, little evidence of effect modification by body mass index, smoking, alcohol consumption, family history of disease, and sex in these associations.

Conclusions: Moderate levels of sweat-inducing exercise showed the lowest risk of myocardial infarction, stroke, hypertension, type 2 diabetes, stomach, lung, liver and head & neck cancers. Public health and lifestyle interventions should, therefore, promote moderate levels of sweat-causing exercise as a behavioural prevention strategy for non-communicable diseases in a wider population of East Asians.

For peer review only

Keywords: exercise, non-communicable disease, cohort, epidemiology, cardiovascular disease, hypertension, cancer

Article Summary

Strengths and limitations of this study

- This study is the first to investigate the longitudinal associations of exercise with various cardiovascular disease and cancer incidence using a large-scale cohort dataset of South Korean adults (n=257,854) who provided up to 7 repeated measures of exercise and all confounders in order to minimise the risk of regression dilution.
- A limitation is that no strong inference can be drawn about the exercise-incident disease relationships.
- Findings of this study may not be generalizable to adult populations of other ethnic origins.

Introduction

Prevention and control of non-communicable diseases is a contemporary global public health priority. At present, 40 million deaths per year, which accounts for nearly 70% of total deaths globally, are attributable to non-communicable diseases.[1,2] Moreover, the number of deaths due to non-communicable diseases, such as cardiovascular disease,[3] hypertension,[4] diabetes[5] and cancer,[6] has increased dramatically over the past few decades, although age-standardised cardiovascular disease and cancer rates as well as systolic blood pressure levels[7] have declined.[8,9] However, trends in these disease traits have varied across different populations, particularly with less favourable changes observed in East Asian populations compared with Western populations. For example, the prevalence of diabetes[10] has increased more rapidly, while the age-standardised prevalence of cardiovascular disease[3]

For peer review only

and systolic blood pressure levels[7] have fallen less steeply in East Asians in comparison with Westerners.

In addition, adults in East Asia tend to have higher prevalence of physical inactivity,[11] which is one of the four target behaviours (including unhealthy diet, tobacco use and harmful use of alcohol) that have been set as the global focus to reduce the risk of non-communicable diseases.[12] The beneficial impacts of increased physical activity on various non-communicable outcomes have been demonstrated by numerous previous investigations. However, the majority of previous research has been predicated on evidence from Western populations, thereby limiting its application to other populations including East Asians. As such, little is currently known about levels of physical activity including exercise in relation to non-communicable diseases in East Asian populations as compared with Western populations.[13] Another critical gap in the existing literature is the use of data measured only at a single point in time (i.e., baseline), in which case physical activity or exercise levels are assumed to remain constant over time. This methodology, therefore, precludes the fact that individuals' physical activity or exercise levels change with time, and hence may increase the potential for regression dilution.[14] Furthermore, it is well-known that temporal changes occur in other traditional behavioural and metabolic risk factors for non-communicable diseases, such as adiposity levels,[15,16] smoking,[17] glucose levels[18] and total cholesterol levels,[19] exhibiting different patterns of changes between East Asian and Western populations. Nevertheless, no previous research of East Asians or Westerners took into account changes in these risk markers in understanding the relationships between physical activity and non-communicable diseases. Moreover, the dose-response relationship between physical activity and various non-communicable disease outcomes has remained unclear in East Asians. Therefore, the purpose of this research was to explore the dose-response relationships between exercise frequency and various types of incident non-communicable diseases, such as myocardial infarction, stroke, hypertension, type 2 diabetes and site-specific cancers,

For peer review only

using a large-scale prospective cohort of South Korean adults with multiple repeated measures of exercise frequency and other risk markers.

Methods

Study design and participants

This study is based on data from the National Health Insurance Service - Health Screening (NHIS-HEALS) cohort dataset,[20] which is a nationally representative random sample (stratified by 2 groups of sex [males and females], 18 groups of age ranges [less than 1 year, 1-4 years, every 5 years between 5-79 years, and more than 80 years], 3 groups of employment status [insured employees, self-employed individuals and medical aid beneficiaries] and 41 groups of income levels [upper 20% for insured employees, lower 20% for insured self-employed individuals and the lowest level for medical aid beneficiaries][21]) of over 500,000 South Korean adults aged 40-79 years between 2002 and 2003 made available by the NHIS. The NHIS is a single health insurance system in South Korea, which manages and maintains information on the entire South Korean population's healthcare utilization; it is mandatory for all South Koreans to take part in the national health insurance system. The NHIS is also responsible for maintaining national health examination programs involving data from general health examinations of all insured employees, self-employed individuals and medical aid beneficiaries aged over 40 years; it is recommended for them to perform the health examination at least every two years. The health examination involves collection of information on body composition, blood profiles, blood pressure, self-reported lifestyles, self-reported physician-diagnosed disease, and self-reported family history of disease.

The NHIS-HEALS cohort includes a wide variety of information collected between 2002 and 2015: health examination data and demographic and eligibility data (e.g., in-patient and out-patient hospital records, medical bill, health insurance and medical aid beneficiaries). In the present analysis, we utilised health examination data collected between 2002 and 2008 to define the exercise frequency and all confounders. There was a change in the type of self-

For peer review only

report methods in 2009; hence, health examination data collected in or after 2009 were not considered in the analysis due to the inability to harmonise variables. However, we used full follow-up data accrued from 2002 until 2015. This research was approved by the Institutional Review Board (4-2017-0051) of the Yonsei University's Severance Hospital in Republic of Korea.

Exposure

The primary exposure variable of this study was exercise frequency, assessed using questionnaires administered during the health examinations. The specific question asked was "How many times per week do you engage in exercise that causes sweating?" Participants were asked to choose only one of the following 5 possible answers: None, 1-2 times/week, 3-4 times/week, 5-6 times/week and almost every day.

Outcomes

In the present study, we evaluated 14 incident disease outcomes, namely, myocardial infarction; stroke, hypertension; type 2 diabetes mellitus; and stomach, colon, rectum, lung, liver, head & neck, pancreatic, kidney, gall bladder and esophagus cancers. Participants' in-patient and out-patient hospital records (i.e., non-fatal status) and death records (i.e., fatal status) obtained through linkage with Statistics Korea were both classified according to the International Classification of Disease (ICD)-10 codes to classify different incidence types (Supplementary Table 1). Additionally, blood pressure (e.g., systolic ≥140 mm Hg, diastolic ≥90 mm Hg) and fasting glucose levels (e.g., ≥126 mg/dL), both of which were measured during physical examinations, were used in conjunction with physicians' diagnosis information and ICD-10 codes to define incident hypertension and type 2 diabetes, respectively. Each incident disease outcome was defined as the first occurrence of either non-fatal or fatal respective disease cases. Incident disease cases were adjudicated using hospital and death records collected through December 31st, 2015. The median follow-up was 13.0 years (interquartile range: 10.2-11.3 years)

For peer review only

Other covariates

The following covariates were included as confounders in the analyses: sex, body mass index (weight in kilograms (kg) divided by height in meters squared (m^2)), systolic blood pressure, fasting glucose, total cholesterol, family history of heart disease, stroke or hypertension [only in models for incident myocardial infarction, stroke or hypertension], family history of diabetes [only in models for type 2 diabetes], family history of cancer [only in models for incident cancer outcomes], smoking status (never, previously, currently) and alcohol consumption (never, 2-3 times/month, 1-2 times/week, ≥3 times/week).

Statistical analysis

Analyses were performed to summarise descriptive statistics (e.g., means, standard deviations, frequency, and proportions) of each covariate and incident disease outcome for all participants and by exercise frequency category. Cox regression with age as the underlying time scale was used to estimate the associations of exercise frequency with each incident disease outcome, with adjustment for all the above-mentioned confounders as well as without any adjustment. Hazard ratios along with corresponding 95% confidence intervals were calculated to evaluate relative risk of each incident disease outcome. Data were structured to enable the inclusion of exercise frequency and all confounders from both baseline and up to 6 repeated measures as time-updated covariates. This approach takes into account changes in exercise frequency as well as each confounder over time in relation to disease incidence. Individuals who reported no exercise served as a reference group for all comparisons. Effect modification by body mass index (<25, ≥25kg^2/m), smoking status, alcohol consumption, family history of disease and sex was also examined based on Wald tests of interaction terms in the fully adjusted models for each incident disease outcome. Visual inspections of log-log plots provided support for the assumptions of proportional hazards for all covariates. A sensitivity analysis where incident disease cases occurring during the first 2 years of follow-up were removed was performed to address reverse

For peer review only

causality. Analyses were performed in Stata/SE Version 14 (StataCorp LP, College Station, TX).

Patient and Public Involvement.

Neither patients nor members of the public were involved in this study.

Results

Of an initial sample of 512,190 individuals, 74,931 had missing data on at least one of the model covariates, and 179,405 had self-reported physician-diagnosed heart attack, stroke, hypertension (additionally, systolic ≥140 mm Hg or diastolic ≥90 mm Hg), diabetes (additionally, fasting glucose levels ≥126 mg/dL) or cancer at baseline, respectively. Excluding these individuals resulted in a final sample for analysis of 257,854 individuals (Figure 1).

Individuals provided up to 7 measures of exercise frequency and each confounder (i.e., baseline plus 6 repeated measures). Participants' characteristics at baseline are summarised in Table 1. Supplementary Table 2 summarises participants' characteristics at each repeat assessment. Individuals in the categories of 1-2, 3-4 or 5-6 times/week of exercise were slightly younger, but showed higher proportions of family history of disease and lower proportions of never smoking or drinking alcohol, compared with those in the categories of none or almost every day of exercise. Across the seven time points (Supplementary Figure 1), the proportion of individuals who reported no exercise decreased while the proportion who reported 1-2 or 3-4 times/week of exercise increased; there were no noticeable changes for the categories of 5-6 times/week or almost every day of exercise.

Overall, J-shaped associations were found between exercise frequency and incident myocardial infarction, stroke, hypertension and type 2 diabetes. Hazard ratios for these diseases were lowest in the middle categories of exercise frequency (e.g., 3-4 or 5-6 times/week) (Figure 2). There were no associations for the most frequent exercise category

For peer review only

(e.g., almost every day) with the incidence of myocardial infarction, stroke and type 2 diabetes.

J-shaped associations were also found for incident stomach, lung, liver and head & neck cancers (Figure 3). Higher exercise frequencies (e.g., 1-2, 3-4 times/week and almost every day) were associated with lower hazards of incident stomach cancer. No statistical significance was observed for incident colon, rectum, pancreas, kidney, gallbladder and esophagus cancers. Crude event rates per 100,000 person-years in the middle categories of exercise frequency were relatively lower for incident rectum, and esophagus cancers, but higher for incident pancreas, kidney and gallbladder cancers. Cox regression models with no adjustment for confounders (Supplementary Figure 2) and a sensitivity analysis (Supplementary Figure 3) in which incident cases occurring in the first 2 years of follow-up were removed both revealed nearly identical patterns of associations as the main analyses.

Figure 4 shows comparisons of results that showed statistical significance for multiplicative interaction terms between exercise frequency and each incident disease outcome. Strong J-shaped associations for incident hypertension were identified at each level of body mass index. J-shaped associations of exercise frequency with incident hypertension were strong only in the more favourable levels of smoking (e.g., never, previously) and alcohol consumption (e.g., never, 2-3 times/month, 1-2 times/week); no or weak associations were identified in the most harmful level of smoking (e.g., current smokers) and alcohol consumption (e.g., ≥3 times/week). J-shaped associations were evident at all levels of family history of CVD and sex for incident hypertension, and sex for incident type 2 diabetes. Exercise frequency was associated with incident lung cancer in non-obese individuals, but there was no evidence of association in obese individuals. All comparisons stratified by each potential effect modifier are presented in Supplementary Figures 4 and 5.

Discussion

For peer review only

This is the first investigation examining the prospective associations of exercise with various incident non-communicable disease outcomes using multiple repeated measures of covariates in East Asian populations. We identified J-shaped associations of sweat-inducing exercise with incident myocardial infarction, stroke, hypertension, type 2 diabetes, stomach, lung, liver and head & neck cancers, with the greatest benefits being observed in the middle categories of exercise frequency (e.g., 3-4 or 5-6 times/week): 1-2 times/week for head & neck cancer. These findings provide two important clinical and public health implications. First, prevention and management of non-communicable diseases in East Asians may benefit considerably from employing an exercise promotion approach in the context of combined non-communicable disease prevention. Mechanism research indicates that cardiovascular disease and type 2 diabetes have similar biological pathways relating to exercise,[22,23] so an integrated prevention approach can be applied to control and manage these two diseases at a minimum.[5] Moreover, regular participation in exercise can induce favourable changes in intermediate cardiometabolic risk markers,[24] which are important predictors of typical non-communicable diseases. Hence, promoting exercise has great potential to act as an integrative behavioural strategy for preventing and controlling various non-communicable diseases simultaneously in East Asian populations.

Second, individuals who engage in exercise 3-4 or 5-6 times/week, rather than every day, may be able to reduce their risk of developing myocardial infarction, stroke, hypertension, type 2 diabetes, stomach, lung and liver cancers: 1-2 times/week for head & neck cancer. Similar J-shaped associations between high intensity exercise (e.g., running) and cardiovascular disease risk have also been reported in previous cohort studies of Western[25,26] and Japanese adults.[27] Nevertheless, the present study as well as previous research[25-27] found that the risk of developing cardiovascular events in individuals who had the highest level of exercise was not noticeably higher compared with those who had the lowest level of exercise. No previous research in East Asians has found such J-shaped relationships between exercise or physical activity and other incident disease outcomes such as hypertension,[28-32] diabetes[33-40] and different type of cancers.[41-47] However, previous meta-

For peer review only

analyses of cohort studies comprising predominantly Westerners found leisure-time physical activity to have curvilinear (but not J-shaped) associations with the incidence of type 2 diabetes,[48] and linear associations with the incidence of hypertension[49] and various site-specific cancers (liver, lung, head & neck, kidney, colon, rectal, bladder, gastric cardia, breast, endometrial, myeloid leukemia, myeloma, esophageal adenocarcinoma).[50] While additional research is needed to confirm the J-shaped associations of exercise with various incident diseases in other samples of East Asians, findings of this research provide a strong rationale for development and implementation of public health policies and clinical trials aimed at promoting a moderate level of sweat-causing exercise to minimise the risk of myocardial infarction, stroke, hypertension, type 2 diabetes, stomach, lung, liver and head & neck cancers.

Another finding of this research is that associations of sweat-inducing exercise with hypertension were modified by body mass index, smoking, alcohol consumption, family history of cardiovascular disease and sex: lung cancer by body mass index and type 2 diabetes by sex. Notably, exercise frequency was not associated with hypertension in individuals who are smokers or drinking alcohol ≥3 times/week (except for 3-4 times/week of exercise). This observation provides some evidence that the harmful impacts of smoking or binge drinking on hypertension[51-53] may not be offset completely by exercise. This, in turn, appears to advocate for the need for implementing a combined hypertension prevention strategy targeting promotion of exercise in conjunction with smoking cessation and reductions in alcohol consumption in East Asians.[13] For lung cancer, the null associations in individuals with body mass index ≥25 may be indicative of potential residual confounding through reported bias in smoking behaviours. Nonetheless, there was little evidence for effect modification for other disease comparisons, highlighting the importance of promoting exercise for the prevention of various non-communicable diseases in individuals at different levels of body mass index, smoking, alcohol consumption, family history of disease and sex.

For peer review only

This study has several notable strengths. First, we used data from a large prospective cohort study in which exercise and other risk markers were assessed on multiple occasions (up to 7 times). Nearly 84% and 5% of the full participants provided 1 and 6 repeated measures of all covariates, respectively. Compelling evidence indicates that the risk of regression dilution can be reduced using repeated measures of exposure and confounders.[14] Moreover, we examined the dose-response relationship of exercise frequency with a wide variety of specific types of incident non-communicable disease outcomes simultaneously using in-patient and out-patient diagnosis data as well as mortality data. The large sample size (n=257,854) is another strength.

This study has some limitations. Findings of this study may not be generalizable to adult populations of other ethnic origins. Due to the observational nature of this research, no strong inference can be drawn about the exercise-incident disease relationships. In addition, the accuracy of hospital admission records is uncertain, although the accuracy of death records from Statistics Korea was found to be 92% in previous research.[54] No information about medication use was available in the cohort data, so we could not use it as a potential confounder and another condition when defining disease status (e.g., hypertension, type 2 diabetes) at both baseline and follow-up. Furthermore, no exercise duration was assessed; hence, inference was made purely based on exercise frequency. Moreover, ICD-10 codes for sex-specific cancers (e.g., prostate and breast cancers) were masked due to the data management policy set forth by the NHIS, so it was not possible to examine such cancers in the present study. The lack of data on diet, which is another behavioural risk marker for non-communicable diseases[12] is another limitation. Moreover, a sizeable proportion (n=74,931; 14.6%) of individuals were excluded due to the missing information on the covariates. Another limitation is that the measurement methods to assess the covariates were not standardized across the different medical institutes participating in the NHIS-HEALS cohort.

Conclusion

For peer review only

Individuals who engaged in sweat-inducing exercise around 3-6 times/week (as opposed to every day) generally had the lowest risk of developing myocardial infarction, stroke, hypertension, type 2 diabetes, stomach, lung, liver and head & neck cancers. These findings were generally applicable to different sub-populations as stratified by body mass index, smoking, alcohol consumption, family history of disease, and sex. Public health and lifestyle interventions should promote a moderate level of sweat-inducing exercise as a behavioural strategy for prevention and control of non-communicable diseases in a wider population of East Asians.

Author Contributions

YK designed this study, performed statistical analysis, and drafted an initial version of the manuscript. SJS, SMH and SHJ all contributed to conceptualizing the study idea and developing the analytical plans, and provided assistance with statistical analysis. All authors critically reviewed, approved of the final version of the manuscript, and agreed to be responsible for all facets of this work.

Acknowledgements

None.

Funding

This work was supported by the National R&D Program for Cancer Control, Ministry of Health & Welfare, Republic of Korea (Grant 1631020 to S.H.J.), The Korean Health Technology R&D Project, Ministry of Health & Welfare, Republic of Korea (Grant HI14C2686 to S.H.J.), and the Medical Research Council (Grant MC_UU_12015/1 to S.J.S.). The funders had no role in study design, data collection and analysis, decision to publish, or preparation of the manuscript.

Conflicts of interest

None declared.

For peer review only

Patient consent

Not required.

Ethics approval

This research was approved by the Institutional Review Board (4-2017-0051) of the Yonsei University's Severance Hospital in Republic of Korea.

Data sharing statement

Data sharing is not applicable because no informed consent for data sharing was obtained from the participants.

For peer review only

References

1 Mortality, G. B. D. & Causes of Death, C. Global, regional, and national life expectancy, all-cause mortality, and cause-specific mortality for 249 causes of death, 1980-2015: a systematic analysis for the Global Burden of Disease Study 2015. *Lancet* **388**, 1459-1544, doi:10.1016/S0140-6736(16)31012-1 (2016).

2 Lakerveld, J., Bot, S. D., van der Ploeg, H. P. & Nijpels, G. The effects of a lifestyle intervention on leisure-time sedentary behaviors in adults at risk: The Hoorn Prevention Study, a randomized controlled trial. *Prev Med* **57**, 351-356, doi:10.1016/j.ypmed.2013.06.011 (2013).

3 Roth, G. A. *et al.* Global, Regional, and National Burden of Cardiovascular Diseases for 10 Causes, 1990 to 2015. *J Am Coll Cardiol* **70**, 1-25, doi:10.1016/j.jacc.2017.04.052 (2017).

4 Forouzanfar, M. H. *et al.* Global Burden of Hypertension and Systolic Blood Pressure of at Least 110 to 115 mm Hg, 1990-2015. *Jama* **317**, 165-182, doi:10.1001/jama.2016.19043 (2017).

5 *World Health Organization Global report on diabetes*. (World Health Organization, 2016).

6 Global Burden of Disease Cancer, C. *et al.* Global, Regional, and National Cancer Incidence, Mortality, Years of Life Lost, Years Lived With Disability, and Disability-Adjusted Life-years for 32 Cancer Groups, 1990 to 2015: A Systematic Analysis for the Global Burden of Disease Study. *JAMA Oncol* **3**, 524-548, doi:10.1001/jamaoncol.2016.5688 (2017).

7 Danaei, G. *et al.* National, regional, and global trends in systolic blood pressure since 1980: systematic analysis of health examination surveys and epidemiological studies with 786 country-years and 5.4 million participants. *Lancet* **377**, 568-577, doi:10.1016/S0140-6736(10)62036-3 (2011).

8 Roth, G. A. *et al.* Global and regional patterns in cardiovascular mortality from 1990 to 2013. *Circulation* **132**, 1667-1678, doi:10.1161/CIRCULATIONAHA.114.008720 (2015).

9 Death, G. C. Global, regional, and national age-sex specific mortality for 264 causes of death, 1980-2016: a systematic analysis for the Global Burden of Disease Study 2016 (vol 390, pg 1151, 2017). *Lancet* **390**, E38-E38 (2017).

10 NCD-, N. R. F. C. NCD Risk Factor Collaboration (NCD-RisC). Worldwide trends in diabetes since 1980: a pooled analysis of 751 population-based studies with 4.4 million participants (vol 387, pg 1513, 2016). *Lancet* **389**, E2-E2, doi:10.1016/S0140-6736(16)32060-8 (2017).

11 Ku, P. W., Fox, K. R., McKenna, J. & Peng, T. L. Prevalence of leisure-time physical activity in Taiwanese adults: results of four national surveys, 2000-2004. *Preventive medicine* **43**, 454-457, doi:10.1016/j.ypmed.2006.04.011 (2006).

12 World Health Organization. Global status report on noncommunicable diseases 2010. Geneva, World Health Organization, 2011.

13 Kokubo, Y. Prevention of hypertension and cardiovascular diseases: a comparison of lifestyle factors in Westerners and East Asians. *Hypertension* **63**, 655-660, doi:10.1161/HYPERTENSIONAHA.113.00543 (2014).

14 Hutcheon, J. A., Chiolero, A. & Hanley, J. A. Random measurement error and regression dilution bias. *Bmj* **340**, c2289, doi:10.1136/bmj.c2289 (2010).

15 Ng, M. *et al.* Global, regional, and national prevalence of overweight and obesity in children and adults during 1980-2013: a systematic analysis for the Global Burden of Disease Study 2013. *Lancet* **384**, 766-781, doi:10.1016/S0140-6736(14)60460-8 (2014).

16 Lear, S. A. *et al.* Visceral adipose tissue accumulation differs according to ethnic background: results of the Multicultural Community Health Assessment Trial (M-CHAT). *Am J Clin Nutr* **86**, 353-359 (2007).

17 (ed Geneva: World Health Organization) (2015).

18 Chan, J. C. *et al.* Diabetes in Asia: epidemiology, risk factors, and pathophysiology. *Jama* **301**, 2129-2140, doi:10.1001/jama.2009.726 (2009).

For peer review only

19 Nguyen, H. N., Fujiyoshi, A., Abbott, R. D. & Miura, K. Epidemiology of cardiovascular risk factors in Asian countries. *Circulation journal : official journal of the Japanese Circulation Society* **77**, 2851-2859 (2013).

20 Seong, S. C. *et al.* Cohort profile: the National Health Insurance Service-National Health Screening Cohort (NHIS-HEALS) in Korea. *BMJ open* **7**, e016640, doi:10.1136/bmjopen-2017-016640 (2017).

21 Lee, J., Lee, J. S., Park, S. H., Shin, S. A. & Kim, K. Cohort Profile: The National Health Insurance Service-National Sample Cohort (NHIS-NSC), South Korea. *Int J Epidemiol* **46**, e15, doi:10.1093/ije/dyv319 (2017).

22 Pedersen, B. K. Anti-inflammatory effects of exercise: role in diabetes and cardiovascular disease. *Eur J Clin Invest* **47**, 600-611, doi:10.1111/eci.12781 (2017).

23 Stern, M. P. Diabetes and cardiovascular disease. The "common soil" hypothesis. *Diabetes* **44**, 369-374 (1995).

24 *Global Recommendations on Physical Activity for Health*. (2010).

25 Lee, D. C. *et al.* Leisure-Time Running Reduces All-Cause and Cardiovascular Mortality Risk. *J Am Coll Cardiol* **64**, 472-481, doi:10.1016/j.jacc.2014.04.058 (2014).

26 Tanasescu, M. *et al.* Exercise type and intensity in relation to coronary heart disease in men. *Jama* **288**, 1994-2000 (2002).

27 Kubota, Y. *et al.* Daily Total Physical Activity and Incident Stroke: The Japan Public Health Center-Based Prospective Study. *Stroke* **48**, 1730-1736, doi:10.1161/STROKEAHA.117.017560 (2017).

28 Lu, Y. *et al.* Lifestyle and Risk of Hypertension: Follow-Up of a Young Pre-Hypertensive Cohort. *Int J Med Sci* **12**, 605-612, doi:10.7150/ijms.12446 (2015).

29 Sun, Z. *et al.* Incidence and predictors of hypertension among rural Chinese adults: results from Liaoning province. *Ann Fam Med* **8**, 19-24, doi:10.1370/afm.1018 (2010).

30 Gu, D. *et al.* Incidence and predictors of hypertension over 8 years among Chinese men and women. *J Hypertens* **25**, 517-523, doi:10.1097/HJH.0b013e328013e7f4 (2007).

31 Lee, S. *et al.* Cardiorespiratory fitness attenuates metabolic risk independent of abdominal subcutaneous and visceral fat in men. *Diabetes Care* **28**, 895-901, doi:DOI 10.2337/diacare.28.4.895 (2005).

32 Nakanishi, N. & Suzuki, K. Daily life activity and the risk of developing hypertension in middle-aged Japanese men. *Archives of internal medicine* **165**, 214-220, doi:10.1001/archinte.165.2.214 (2005).

33 Fan, S. *et al.* Physical Activity Level and Incident Type 2 Diabetes among Chinese Adults. *Med Sci Sport Exer* **47**, 751-756, doi:10.1249/Mss.0000000000000471 (2015).

34 Joseph, J. J. *et al.* Physical activity, sedentary behaviors and the incidence of type 2 diabetes mellitus: the Multi-Ethnic Study of Atherosclerosis (MESA). *Bmj Open Diab Res Ca* **4**, doi:ARTN e00018510.1136/bmjdrc-2015-000185 (2016).

35 Xu, F. *et al.* Joint Associations of Physical Activity and Hypertension with the Development of Type 2 Diabetes among Urban Men and Women in Mainland China. *Plos One* **9**, doi:ARTN e8871910.1371/journal.pone.0088719 (2014).

36 Xu, F. *et al.* Joint impact of physical activity and family history on the development of diabetes among urban adults in Mainland China: a pooled analysis of community-based prospective cohort studies. *Asia Pac J Public Health* **27**, NP372-381, doi:10.1177/1010539512443700 (2015).

37 Shi, L. *et al.* Physical activity, smoking, and alcohol consumption in association with incidence of type 2 diabetes among middle-aged and elderly Chinese men. *Plos One* **8**, e77919, doi:10.1371/journal.pone.0077919 (2013).

38 Villegas, R. *et al.* Physical activity and the incidence of type 2 diabetes in the Shanghai women's health study. *Int J Epidemiol* **35**, 1553-1562, doi:10.1093/ije/dyl209 (2006).

For peer review only

39 Lee, D. C. *et al.* Physical Activity and Body Mass Index and Their Associations With the Development of Type 2 Diabetes in Korean Men. *Am J Epidemiol* **176**, 43-51, doi:10.1093/aje/kwr471 (2012).
40 Honda, T. *et al.* Leisure-time, occupational, and commuting physical activity and risk of type 2 diabetes in Japanese workers: a cohort study. *Bmc Public Health* **15**, doi:ARTN 100410.1186/s12889-015-2362-5 (2015).

41 Inoue, M. *et al.* Daily total physical activity level and total cancer risk in men and women: results from a large-scale population-based cohort study in Japan. *Am J Epidemiol* **168**, 391-403, doi:10.1093/aje/kwn146 (2008).
42 Lee, K. J. *et al.* Physical activity and risk of colorectal cancer in Japanese men and women: the Japan Public Health Center-based prospective study. *Cancer causes & control : CCC* **18**, 199-209, doi:10.1007/s10552-006-0098-3 (2007).
43 Luo, J. *et al.* Body mass index, physical activity and the risk of pancreatic cancer in relation to smoking status and history of diabetes: a large-scale population-based cohort study in Japan--the JPHC study. *Cancer causes & control : CCC* **18**, 603-612, doi:10.1007/s10552-007-9002-z (2007).
44 Suzuki, S. *et al.* Effect of physical activity on breast cancer risk: findings of the Japan collaborative cohort study. *Cancer epidemiology, biomarkers & prevention : a publication of the American Association for Cancer Research, cosponsored by the American Society of Preventive Oncology* **17**, 3396-3401, doi:10.1158/1055-9965.EPI-08-0497 (2008).
45 Eaglehouse, Y. L. *et al.* Physical activity, sedentary time, and risk of colorectal cancer: the Singapore Chinese Health Study. *Eur J Cancer Prev* **26**, 469-475, doi:10.1097/CEJ.0000000000000369 (2017).
46 Mok, Y., Jeon, C., Lee, G. J. & Jee, S. H. Physical Activity Level and Colorectal Cancer Mortality. *Asia Pac J Public Health* **28**, 638-647, doi:10.1177/1010539516661761 (2016).
47 Yun, Y. H. *et al.* Dietary preference, physical activity, and cancer risk in men: national health insurance corporation study. *Bmc Cancer* **8**, 366, doi:10.1186/1471-2407-8-366 (2008).
48 Smith, A. D., Crippa, A., Woodcock, J. & Brage, S. Physical activity and incident type 2 diabetes mellitus: a systematic review and dose-response meta-analysis of prospective cohort studies. *Diabetologia* **59**, 2527-2545, doi:10.1007/s00125-016-4079-0 (2016).
49 Liu, X. J. *et al.* Dose-Response Association Between Physical Activity and Incident Hypertension A Systematic Review and Meta-Analysis of Cohort Studies. *Hypertension* **69**, 813-+, doi:10.1161/Hypertensionaha.116.08994 (2017).
50 Moore, S. C. *et al.* Association of Leisure-Time Physical Activity With Risk of 26 Types of Cancer in 1.44 Million Adults. *JAMA internal medicine* **176**, 816-825, doi:10.1001/jamainternmed.2016.1548 (2016).
51 Stranges, S. *et al.* Relationship of alcohol drinking pattern to risk of hypertension: a population-based study. *Hypertension* **44**, 813-819, doi:10.1161/01.HYP.0000146537.03103.f2 (2004).
52 Niskanen, L. *et al.* Inflammation, abdominal obesity, and smoking as predictors of hypertension. *Hypertension* **44**, 859-865, doi:10.1161/01.HYP.0000146691.51307.84 (2004).
53 Halperin, R. O., Gaziano, J. M. & Sesso, H. D. Smoking and the risk of incident hypertension in middle-aged and older men. *Am J Hypertens* **21**, 148-152, doi:10.1038/ajh.2007.36 (2008).
54 Won, T., Kang, B., Im, T. & Choi, H. The study of accuracy of death statistics. *J Korean Soc Emerg Med* **18**, 256–262 (2007).

Figure Legends

Figure 1. A flow diagram. Note: “N” indicates numbers of total observations (i.e. participants who provided repeated measures are treated as separate observations) and “n” indicates numbers of unique participants at baseline. Data without missingness and prevalence of major diseases were used to create final analysis datasets for different incident disease outcomes;

For peer review only

while the number of unique participants at baseline is the same for all incident disease outcomes, the total number of observations varied due to the nature of time-updated covariate analyses (i.e. censoring of subsequent time-updated covariates when an incident disease case occurs before the end date of repeated measures). Data were obtained from a prospective cohort, which has been established by linking physical examination data of over half a million South Korean adults (2002-2015) with the entire South Korean population's health insurance system.

Figure 2. Associations of exercise frequency with various incident cardiovascular disease outcomes. Cox regression models with age as the underlying timescale were adjusted for sex, body mass index, systolic blood pressure, fasting glucose levels, total cholesterol levels, family history of heart disease, stroke or hypertension (in models for myocardial infarction, stroke and hypertension) or diabetes (in models for Type 2 diabetes), smoking status and alcohol consumption. Crude rates are per 100,000 person-years. "N" indicates numbers of total observations (i.e. participants who provided repeated measures are treated as separate observations) and "n" indicates numbers of unique participants at baseline. Data were obtained from a prospective cohort, which has been established by linking physical examination data of over half a million Korean adults (2002-2015) with the entire South Korean population's health insurance system. Abbreviations: HR – Hazard Ratio; CI – Confidence Intervals

Figure 3. Associations of exercise frequency with various incident cancer outcomes. Cox regression models with age as the underlying timescale were adjusted for sex, body mass index, systolic blood pressure, fasting glucose levels, total cholesterol levels, family history of cancer, smoking status and alcohol consumption. Crude rates are per 100,000 person-years. "N" indicates numbers of total observations (i.e. participants who provided repeated measures are treated as separate observations) and "n" indicates numbers of unique participants at baseline. Data were obtained from a prospective cohort, which has been established by linking physical examination data of over half a million South Korean adults (2002-2015) with the entire South Korean population's health insurance system. Abbreviations: HR – Hazard Ratio; CI – Confidence Intervals

Figure 4. Results from assessment of effect modification of sex, body mass index, smoking, alcohol consumption, and family history of disease in the associations between exercise frequency and each incident disease outcome. Only associations for which the multiplicative interaction terms were statistically significant are presented. Cox regression models with age as the underlying timescale were adjusted for sex [not in models for effect modification by sex], body mass index [not in models for effect modification by body mass index], systolic blood pressure, fasting glucose levels, total cholesterol levels, family history [not in models for effect modification by family history of respective disease] of heart disease/stroke/hypertension (in models for myocardial infarction, stroke and hypertension), diabetes (in models for Type 2 diabetes) or cancer (in models for each cancer), smoking status [not in models for effect modification by smoking status] and alcohol consumption [not in models for effect modification by alcohol consumption]. Crude rates are per 100,000 person-years. "N" indicates numbers of total observations (i.e. participants who provided repeated measures are treated as separate observations) and "n" indicates numbers of unique participants at baseline. P-values for multiplicative interactions with exercise frequency are as follows; Outcome – hypertension: body mass index (0.049), smoking (<0.001), alcohol consumption (<0.001), family history of cardiovascular disease (0.029) and sex (<0.001); Outcome - lung cancer: body mass index (0.016), and; Outcome - type 2 diabetes: sex (0.012). Data were obtained from a prospective cohort, which has been established by linking physical examination data of over half a million South Korean adults (2002-2015) with the entire South

For peer review only

Korean population's health insurance system. Abbreviations: HR – Hazard Ratio; CI – Confidence Intervals

For peer review only

Table 1. Characteristics of the participants at baseline.

Variables	All (n=257,854)	Exercise frequency				
		None (n=148,284)	1-2 times/week (n=62,923)	3-4 times/week (n=24,836)	5-6 times/week (n=6,676)	Almost everyday (n=15,135)
Sex, %						
Men	50.5%	43.8%	63.3%	57.5%	52.9%	49.6%
Women	49.5%	56.2%	36.7%	42.5%	47.1%	50.4%
Age, years	50.7 (8.7)	51.5 (9.2)	49.0 (7.5)	49.3 (7.5)	49.9 (7.8)	53.5 (9.3)
Body Mass Index, kg^2/m	23.5 (2.8)	23.3 (2.9)	23.6 (2.7)	23.7 (2.6)	23.7 (2.6)	23.7 (2.7)
Systolic blood pressure, mm Hg	116.8 (11.2)	116.6 (11.3)	117.1 (11.0)	116.8 (11.1)	116.8 (11.2)	117.3 (11.3)
Diastolic blood pressure, mm Hg	73.4 (7.9)	73.1 (8.0)	73.8 (7.8)	73.5 (7.9)	73.4 (8.0)	73.4 (7.9)
Fasting glucose levels, mg/dL	90.2 (12.3)	90.1 (12.4)	90.4 (12.2)	89.9 (11.9)	90.2 (12.3)	90.2 (12.4)
Total cholesterol, mg/dL	197.0 (36.7)	196.5 (37.1)	197.7 (36.2)	197.8 (35.9)	197.8 (36.1)	197.4 (36.8)
Family history of heart disease, stroke or hypertension, %	12.2%	10.8%	13.9%	15.9%	16.5%	10.9%
Family history of cancer, %	14.2%	13.1%	15.3%	17.2%	16.6%	14.5%
Family history of diabetes, %	6.3%	5.4%	7.2%	8.5%	9.2%	5.7%
Smoking status, %						
Never	68.1%	71.9%	59.1%	65.2%	68.8%	71.7%
Previously	8.4%	5.8%	12.0%	12.7%	12.5%	8.8%
Currently	23.6%	22.2%	28.9%	22.1%	18.8%	19.5%
Alcohol Consumption, %						
Never	58.4%	64.5%	47.6%	50.5%	52.2%	59.3%
2-3 times/month	16.4%	13.8%	21.3%	19.7%	18.8%	14.1%
1-2 times/week	15.8%	12.4%	22.0%	20.2%	18.1%	14.5%
≥3 times/week	9.5%	9.3%	9.1%	9.5%	10.8%	12.1%
Incident myocardial infarction, n (%)	3,047 (1.2)	1,741 (1.2)	723 (1.1)	276 (1.1)	88 (1.3)	219 (1.4)
Incident stroke, n (%)	16,134 (6.3)	9,689 (6.5)	3,333 (5.3)	1,482 (6.0)	390 (5.8)	1,240 (8.2)
Incident hypertension, n (%)	120,203 (46.6)	65,964 (44.5)	30,623 (48.7)	12,617 (50.8)	3,294 (49.3)	7,705 (50.9)
Incident Type 2 diabetes, n (%)	50,459 (19.6)	27,128 (18.3)	10,666 (6.5)	5,421 (21.8)	1,399 (21.0)	3,285 (21.7)
Incident stomach cancer, n (%)	4,788 (1.9)	2,672 (1.8)	13,226 (21.0)	489 (2.0)	139 (2.1)	328 (2.2)
Incident colon cancer, n (%)	2,711 (1.1)	1,424 (1.0)	1,160 (1.8)	314 (1.3)	90 (1.3)	191 (1.3)
Incident rectum cancer, n (%)	1,494 (0.6)	809 (0.6)	692 (1.1)	154 (0.6)	46 (0.6)	107 (0.7)
Incident lung cancer, n (%)	3,601 (1.4)	2,138 (1.4)	796 (1.3)	307 (1.2)	85 (1.3)	275 (1.8)
Incident liver cancer, n (%)	2,620 (1.0)	1,423 (1.0)	680 (1.1)	263 (1.1)	75 (1.1)	179 (1.2)
Incident pancreas cancer, n (%)	864 (0.3)	483 (0.3)	205 (0.3)	92 (0.4)	24 (0.4)	60 (0.4)
Incident head & neck cancer, n (%)	656 (0.3)	377 (0.3)	144 (0.2)	73 (0.3)	15 (0.2)	47 (0.3)
Incident kidney cancer, n (%)	589 (0.2)	301 (0.2)	153 (0.2)	75 (0.3)	16 (0.2)	44 (0.3)
Incident gallbladder cancer, n (%)	400 (0.2)	219 (0.1)	83 (0.1)	43 (0.2)	16 (0.2)	39 (0.3)
Incident esophagus cancer, n (%)	352 (0.1)	214 (0.1)	75 (0.1)	29 (0.1)	6 (0.09)	28 (0.2)
Median follow-up period, years (interquartile range)	13.0 (12.2, 13.3)	13.0 (12.2, 13.3)	13.0 (12.2, 13.3)	13.0 (12.2, 13.3)	12.7 (12.2, 13.3)	12.6 (12.2, 13.3)

Note: Values presented are means unless indicated as an 'n'. Values in parentheses are standard deviations unless otherwise indicated. Data were obtained from a prospective cohort, which has been established by linking physical examination data of over half a million South Korean adults (2002-2015) with the entire South Korean population's health insurance system.

For peer review only

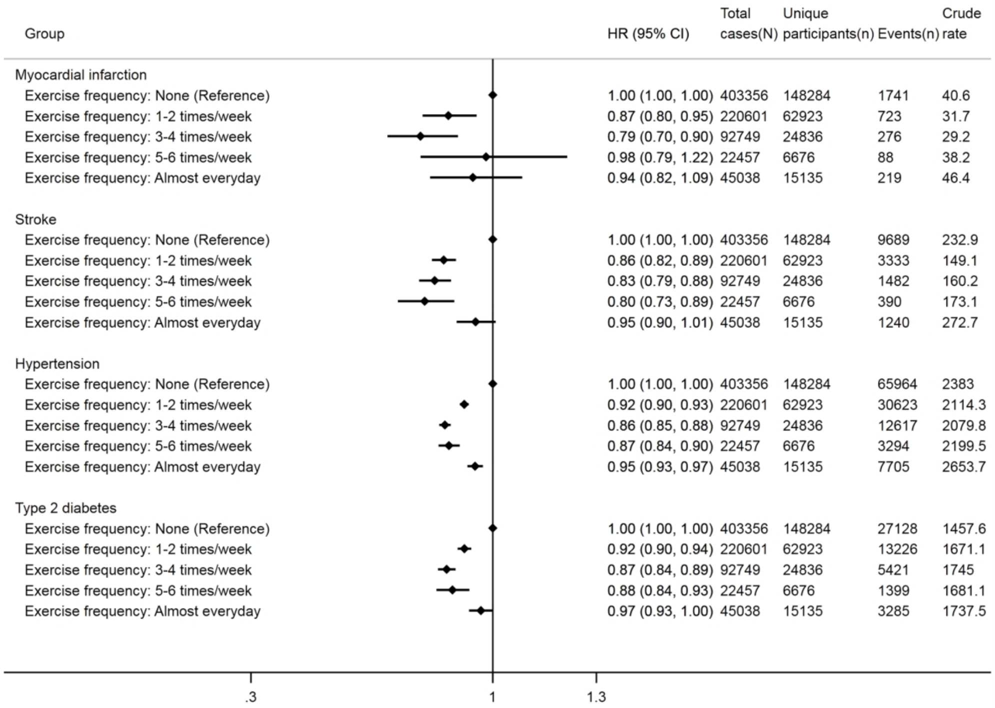

Figure 2. Associations of exercise frequency with various incident cardiovascular disease outcomes. Cox regression models with age as the underlying timescale were adjusted for sex, body mass index, systolic blood pressure, fasting glucose levels, total cholesterol levels, family history of heart disease, stroke or hypertension (in models for myocardial infarction, stroke and hypertension) or diabetes (in models for Type 2 diabetes), smoking status and alcohol consumption. Crude rates are per 100,000 person-years. “N” indicates numbers of total observations (i.e. participants who provided repeated measures are treated as separate observations) and “n” indicates numbers of unique participants at baseline. Data were obtained from a prospective cohort, which has been established by linking physical examination data of over half a million Korean adults (2002-2015) with the entire South Korean population’s health insurance system. Abbreviations: HR – Hazard Ratio; CI – Confidence Intervals

209x159mm (300 x 300 DPI)

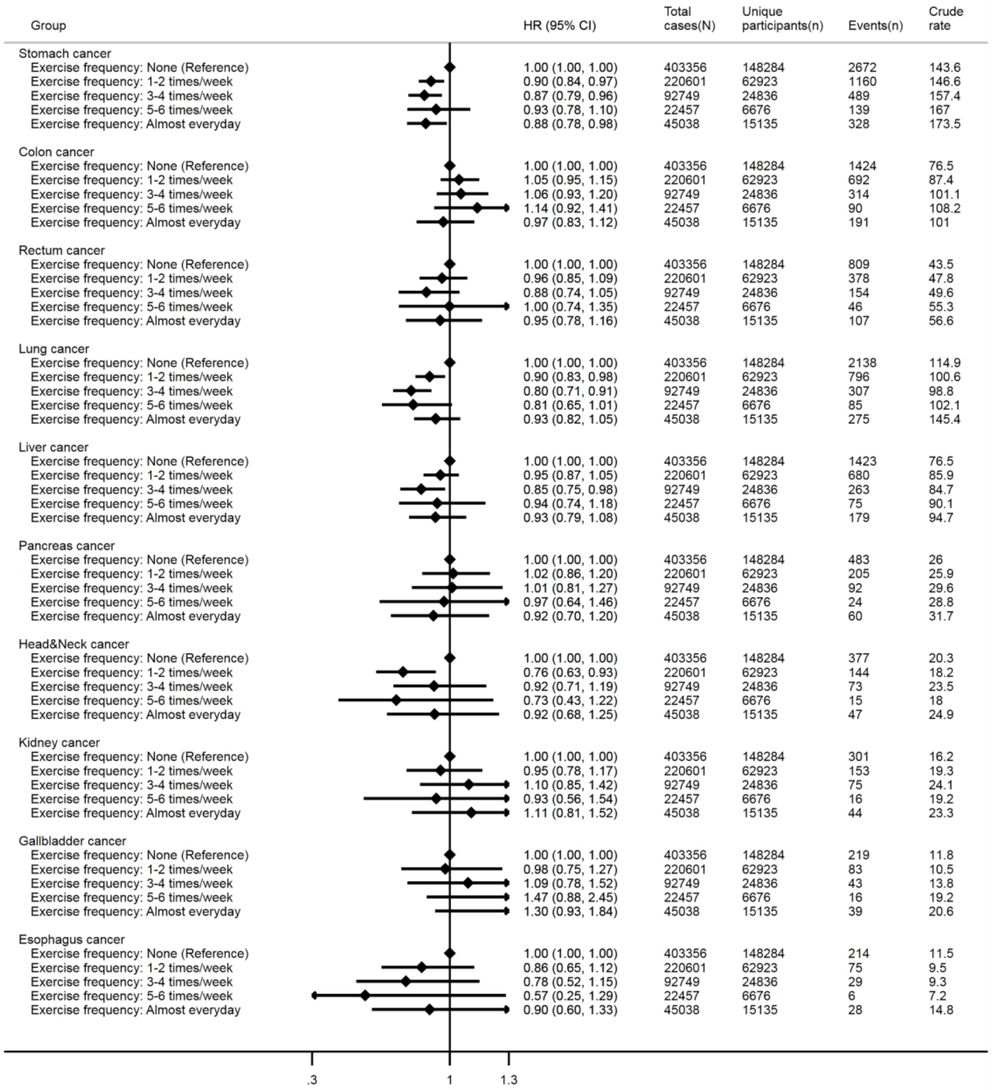

Figure 3. Associations of exercise frequency with various incident cancer outcomes. Cox regression models with age as the underlying timescale were adjusted for sex, body mass index, systolic blood pressure, fasting glucose levels, total cholesterol levels, family history of cancer, smoking status and alcohol consumption. Crude rates are per 100,000 person-years. "N" indicates numbers of total observations (i.e. participants who provided repeated measures are treated as separate observations) and "n" indicates numbers of unique participants at baseline. Data were obtained from a prospective cohort, which has been established by linking physical examination data of over half a million South Korean adults (2002-2015) with the entire South Korean population's health insurance system. Abbreviations: HR – Hazard Ratio; CI – Confidence Intervals

268x297mm (300 x 300 DPI)

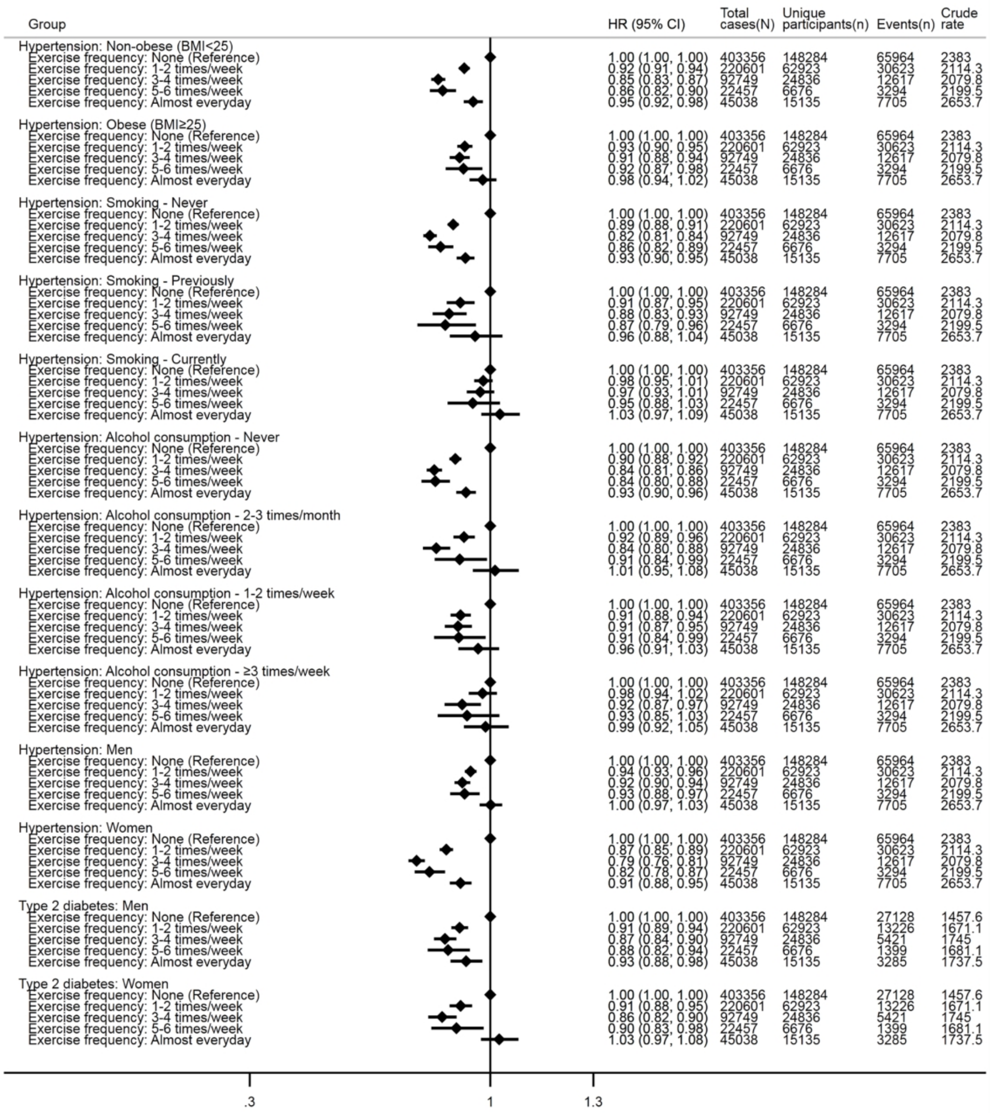

Figure 4. Results from assessment of effect modification of sex, body mass index, smoking, alcohol consumption, and family history of disease in the associations between exercise frequency and each incident disease outcome. Only associations for which the multiplicative interaction terms were statistically significant are presented. Cox regression models with age as the underlying timescale were adjusted for sex [not in models for effect modification by sex], body mass index [not in models for effect modification by body mass index], systolic blood pressure, fasting glucose levels, total cholesterol levels, family history [not in models for effect modification by family history of respective disease] of heart disease/stroke/hypertension (in models for myocardial infarction, stroke and hypertension), diabetes (in models for Type 2 diabetes) or cancer (in models for each cancer), smoking status [not in models for effect modification by smoking status] and alcohol consumption [not in models for effect modification by alcohol consumption]. Crude rates are per 100,000 person-years. "N" indicates numbers of total observations (i.e. participants who provided repeated measures are treated as separate observations) and "n" indicates numbers of unique participants at baseline. P-values for multiplicative interactions with exercise frequency are as follows; Outcome – hypertension: body mass index (0.049), smoking (<0.001), alcohol consumption (<0.001), family history of cardiovascular disease (0.029) and sex (<0.001); Outcome - lung cancer: body mass index (0.016), and; Outcome - type 2 diabetes: sex (0.012)¬¬¬¬¬¬¬. Data were obtained from a prospective cohort, which has been established by linking physical examination data of over half a million South Korean adults (2002-

2015) with the entire South Korean population's health insurance system. Abbreviations: HR – Hazard Ratio; CI – Confidence Intervals

Supplementary Table 1. Definitions for classification of incident disease outcomes

Incident disease outcome	Definition
Myocardial infarction	ICD 10 codes I21-I23
Stroke	ICD 10 codes I60-I64
Hypertension	ICD 10 codes I10, I11 and I15, systolic blood pressure ≥140 mm Hg, diastolic blood pressure ≥90 mm Hg, or physician diagnosis
Type 2 diabetes	ICD 10 codes E11, fasting glucose ≥126 mg/dL, or physician diagnosis
Stomach cancer	ICD 10 codes C16
Colon cancer	ICD 10 codes C18
Rectum cancer	ICD 10 codes C20
Lung cancer	ICD 10 codes C34
Liver cancer	ICD 10 codes C22
Pancreas cancer	ICD 10 codes C25
Head & neck cancer	ICD 10 codes C00-C06, C09-C14 and C32
Kidney cancer	ICD 10 codes C64
Gallbladder cancer	ICD 10 codes C23
Esophagus cancer	ICD 10 codes C15

Abbreviations: ICD – International Classification of Disease

For peer review only

Supplementary Table 2. Participants' characteristics at 1st, 2nd, 3rd, 4th, 5th and 6th repeat-visit

Repeat visit	Variables	All (n=215,295)	Exercise frequency				
1st			None (n=112,156)	1-2 times/week (n=57,777)	3-4 times/week (n=25,390)	5-6 times/week (n= 6,443)	Almost everyday (n=13,529)
	Sex, %						
	Men	51.1%	45.0%	61.8%	55.3%	50.6%	48.8%
	Women	48.9%	55.0%	38.2%	44.7%	49.4%	51.2%
	Age, years	52.7 (8.6)	53.6 (9.2)	50.9 (7.4)	51.3 (7.5)	52.3 (8.0)	55.6 (9.1)
	Body Mass Index, kg^2/m	23.5 (2.8)	23.4 (2.9)	23.6 (2.7)	23.7 (2.6)	23.6 (2.6)	23.7 (2.7)
	Systolic blood pressure, mm Hg	120.5 (14.4)	120.6 (14.7)	120.4 (13.9)	120.0 (13.9)	119.6 (13.9)	121.4 (14.6)
	Diastolic blood pressure, mm Hg	75.7 (9.9)	75.6 (9.9)	75.9 (9.8)	75.5 (9.8)	75.2 (9.8)	75.7 (9.8)
	Fasting glucose levels, mg/dL	92.6 (18.5)	92.6 (18.8)	92.7 (17.4)	92.4 (17.8)	92.8 (21.0)	93.3 (20.2)
	Total cholesterol, mg/dL	197.3 (36.2)	197.0 (36.8)	197.3 (35.6)	197.9 (35.1)	197.4 (35.9)	197.8 (36.7)
	Family history of heart disease, stroke or hypertension, %	12.6%	10.7%	14.2%	16.7%	16.7%	12.0%
	Family history of cancer, %	14.3%	12.7%	15.5%	17.4%	19.2%	14.5%
	Family history of diabetes, %	6.0%	5.0%	6.7%	8.2%	9.0%	5.8%
	Smoking status, %						
	Never	70.7%	74.8%	62.4%	68.9%	71.7%	74.8%
	Previously	8.6%	5.8%	11.9%	12.4%	11.9%	8.8%
	Currently	20.8%	19.4%	25.7%	18.7%	16.3%	16.4%
	Alcohol Consumption, %						
	Never	59.8%	66.9%	49.2%	52.7%	54.9%	61.8%
	2-3 times/month	15.6%	12.7%	20.5%	18.6%	18.2%	12.8%
	1-2 times/week	15.8%	11.8%	21.7%	19.9%	17.0%	14.8%
	≥3 times/week	8.8%	8.7%	8.6%	8.7%	10.0%	10.6%

For peer review only

Supplementary Table 2. Participants' characteristics at 1st, 2nd, 3rd, 4th, 5th and 6th repeat-visit (cont.)

Repeat visit	Variables	All (n=150,845)	Exercise frequency				
2nd			None (n=73,660)	1-2 times/week (n=44,211)	3-4 times/week (n=19,593)	5-6 times/week (n=4,771)	Almost everyday (n= 8,610)
	Sex, %						
	Men	54.2%	47.0%	64.6%	57.9%	53.8%	53.5%
	Women	45.8%	53.0%	35.4%	42.1%	46.2%	46.5%
	Age, years	53.3 (8.3)	54.2 (8.9)	51.7 (7.2)	52.3 (7.4)	53.4 (7.8)	56.5 (9.1)
	Body Mass Index, kg^2/m	23.5 (2.7)	23.4 (2.8)	23.6 (2.6)	23.7 (2.6)	23.6 (2.6)	23.6 (2.7)
	Systolic blood pressure, mm Hg	120.9 (14.1)	121.0 (14.5)	120.7 (13.7)	120.4 (13.6)	120.3 (13.7)	122.0 (14.5)
	Diastolic blood pressure, mm Hg	75.8 (9.6)	75.8 (9.7)	76.0 (9.5)	75.5 (9.4)	75.4 (9.4)	76.0 (9.8)
	Fasting glucose levels, mg/dL	93.2 (16.9)	93.1 (17.4)	93.5 (17.0)	93.1 (15.5)	93.5 (15.9)	93.7 (16.2)
	Total cholesterol, mg/dL	197.6 (35.9)	197.7 (36.5)	197.4 (35.4)	197.8 (35.2)	196.7 (34.9)	198.1 (36.5)
	Family history of heart disease, stroke or hypertension, %	13.4%	11.2%	14.6%	17.5%	18.3%	13.1%
	Family history of cancer, %	14.9%	12.9%	16.4%	17.4%	19.7%	15.4%
	Family history of diabetes, %	6.3%	5.2%	7.1%	8.2%	9.0%	6.1%
	Smoking status, %						
	Never	71.0%	76.5%	62.3%	68.7%	71.2%	74.2%
	Previously	9.0%	5.5%	12.5%	13.1%	13.1%	9.4%
	Currently	20.0%	18.0%	25.3%	18.2%	15.8%	16.5%
	Alcohol Consumption, %						
	Never	59.0%	67.6%	48.1%	51.9%	54.1%	60.0%
	2-3 times/month	16.1%	12.6%	20.9%	19.0%	18.9%	13.6%
	1-2 times/week	16.7%	12.1%	22.8%	20.7%	17.5%	15.8%
	≥3 times/week	8.1%	7.8%	8.2%	8.3%	9.5%	10.7%

For peer review only

Supplementary Table 2. Participants' characteristics at 1st, 2nd, 3rd, 4th, 5th and 6th repeat-visit (cont.)

Repeat visit	Variables	All (n=80,639)	Exercise frequency				
3rd			None (n= 36,123)	1-2 times/week (n=26,505)	3-4 times/week (n=11,432)	5-6 times/week (n=2,391)	Almost everyday (n=4,188)
	Sex, %						
	Men	63.1%	53.9%	73.0%	68.6%	64.5%	63.4%
	Women	36.9%	46.1%	27.0%	31.4%	35.6%	36.7%
	Age, years	52.3 (7.2)	52.9 (7.8)	51.2 (6.2)	51.9 (6.6)	52.8 (7.0)	55.2 (8.4)
	Body Mass Index, kg^2/m	23.5 (2.7)	23.3 (2.8)	23.6 (2.6)	23.7 (2.5)	23.7 (2.6)	23.7 (2.7)
	Systolic blood pressure, mm Hg	121.1 (13.6)	120.9 (13.9)	120.9 (13.4)	121.0 (13.2)	120.6 (13.3)	122.0 (14.0)
	Diastolic blood pressure, mm Hg	76.1 (9.4)	76.0 (9.5)	76.4 (9.4)	76.1 (9.1)	75.6 (9.5)	76.3 (9.6)
	Fasting glucose levels, mg/dL	93.7 (16.8)	93.3 (17.1)	94.1 (16.7)	93.9 (15.9)	94.1 (16.5)	94.5 (17.0)
	Total cholesterol, mg/dL	197.1 (35.2)	197.1 (35.5)	197.4 (34.8)	196.9 (34.7)	196.5 (35.2)	196.7 (35.4)
	Family history of heart disease, stroke or hypertension, %	13.5%	11.4%	14.8%	16.7%	18.6%	11.7%
	Family history of cancer, %	14.8%	13.0%	15.8%	17.8%	18.9%	14.7%
	Family history of diabetes, %	6.4%	5.3%	7.0%	8.0%	9.5%	6.2%
	Smoking status, %						
	Never	66.6%	73.8%	57.6%	63.9%	65.5%	68.8%
	Previously	10.7%	6.1%	14.0%	15.5%	16.6%	12.4%
	Currently	22.8%	20.2%	28.4%	20.6%	17.9%	18.7%
	Alcohol Consumption, %						
	Never	53.6%	64.2%	42.5%	47.2%	47.4%	53.9%
	2-3 times/month	18.5%	14.0%	23.7%	20.8%	21.4%	15.8%
	1-2 times/week	19.5%	14.0%	25.6 %	22.8%	20.7%	18.8%
	≥3 times/week	8.4%	7.8%	8.3%	9.2%	10.5%	11.5%

For peer review only

Supplementary Table 2. Participants' characteristics at 1st, 2nd, 3rd, 4th, 5th and 6th repeat-visit (cont.)

Repeat visit	Variables	All (n=40,910)	Exercise frequency				
4th			None (n=17,678)	1-2 times/week (n=14,624)	3-4 times/week (n= 5,685)	5-6 times/week (n=1,094)	Almost everyday (n=1,829)
	Sex, %						
	Men	72.3%	61.6%	81.1%	81.5%	78.2%	73.9%
	Women	27.7%	38.4%	18.9%	18.5%	21.9%	26.1%
	Age, years	50.9 (5.4)	51.2 (5.5)	50.6 (5.1)	50.7 (5.3)	51.0 (5.6)	52.6 (6.3)
	Body Mass Index, kg^2/m	23.5 (2.7)	23.3 (2.7)	23.7 (2.6)	23.8 (2.6)	23.8 (2.5)	23.7 (2.6)
	Systolic blood pressure, mm Hg	121.4 (13.3)	120.9 (13.5)	121.7 (13.0)	121.5 (12.8)	121.5 (13.1)	122.7 (13.6)
	Diastolic blood pressure, mm Hg	76.6 (9.2)	76.2 (9.3)	77.0 (9.2)	76.8 (8.9)	76.8 (9.4)	77.2 (9.5)
	Fasting glucose levels, mg/dL	93.9 (17.8)	93.3 (17.9)	94.4 (17.9)	94.3 (17.1)	94.0 (16.0)	94.9 (18.8)
	Total cholesterol, mg/dL	196.8 (35.0)	197.2 (35.5)	196.7 (34.6)	196.6 (34.2)	195.0 (34.3)	196.5 (34.9)
	Family history of heart disease, stroke or hypertension, %	12.3%	10.4%	13.7%	14.2%	16.3%	11.6%
	Family history of cancer, %	13.8%	12.6%	14.7%	15.0%	16.3%	13.5%
	Family history of diabetes, %	5.7%	4.8%	6.2%	6.9%	7.5%	6.7%
	Smoking status, %						
	Never	60.6%	69.5%	51.9%	55.5%	57.3%	62.0%
	Previously	12.2%	6.9%	15.1%	18.7%	18.7%	15.7%
	Currently	27.2%	23.7%	33.0%	25.8%	24.0%	22.4%
	Alcohol Consumption, %						
	Never	48.1%	60.0%	38.0%	39.1%	39.9%	45.4%
	2-3 times/month	20.5%	15.5%	24.9%	24.3%	24.0%	19.7%
	1-2 times/week	22.4%	16.2%	28.4%	26.3%	22.6%	22.9%
	≥3 times/week	9.0%	8.2%	8.7%	10.3%	13.6%	12.0%

For peer review only

Supplementary Table 2. Participants' characteristics at 1st, 2nd, 3rd, 4th, 5th and 6th repeat-visit (cont.)

Repeat visit	Variables	All (n= 26,356)	Exercise frequency				
5th			None (n=10,771)	1-2 times/week (n=9,807)	3-4 times/week (n=3,884)	5-6 times/week (n=724)	Almost everyday (n=1,170)
	Sex, %						
	Men	73.7%	62.6%	81.6%	82.4%	81.2%	76.9%
	Women	26.3%	37.4%	18.4%	17.6%	18.8%	23.0%
	Age, years	51.1 (4.9)	51.4 (5.1)	50.8 (4.7)	51.0 (4.7)	51.4 (5.0)	52.1 (5.6)
	Body Mass Index, kg^2/m	23.5 (2.6)	23.3 (2.7)	23.7 (2.6)	23.8 (2.4)	23.7 (2.5)	23.8 (2.6)
	Systolic blood pressure, mm Hg	121.4 (13.0)	121.0 (13.4)	121.8 (12.9)	121.6 (12.6)	120.8 (12.9)	121.5 (12.7)
	Diastolic blood pressure, mm Hg	76.6 (9.1)	76.2 (9.1)	77.1 (9.0)	76.8 (8.8)	76.7 (8.6)	76.4 (9.0)
	Fasting glucose levels, mg/dL	94.3 (17.6)	93.7 (18.1)	94.6 (17.3)	94.7 (16.6)	94.6 (15.9)	95.4 (18.7)
	Total cholesterol, mg/dL	197.1 (34.5)	197.7 (34.8)	196.7 (34.2)	196.4 (33.8)	195.2 (34.2)	198.2 (37.0)
	Family history of heart disease, stroke or hypertension, %	12.6%	11.3%	13.4%	14.3%	13.8%	10.9%
	Family history of cancer, %	14.3%	13.2%	15.1%	14.9%	17.8%	13.5%
	Family history of diabetes, %	5.9%	4.9%	6.4%	6.7%	7.2%	8.6%
	Smoking status, %						
	Never	58.9%	68.1%	50.8%	54.4%	57.0%	58.5%
	Previously	13.1%	7.1%	16.0%	19.3%	19.8%	19.7%
	Currently	28.0%	24.8%	33.2%	26.2%	23.2%	21.9%
	Alcohol Consumption, %						
	Never	46.3%	57.9%	37.6%	38.3%	41.3%	42.9%
	2-3 times/month	21.7%	16.7%	25.8%	24.7%	22.2%	22.3%
	1-2 times/week	23.4%	17.4%	28.0%	27.3%	26.0%	25.0%
	≥3 times/week	8.6%	8.1%	8.5%	9.8%	10.5%	9.7%

For peer review only

Supplementary Table 2. Participants' characteristics at 1st, 2nd, 3rd, 4th, 5th and 6th repeat-visit (cont.)

Repeat visit	Variables	All (n= 12,302)	Exercise frequency				
6th			None (n= 4,684)	1-2 times/week (n=4,754)	3-4 times/week (n=1,929)	5-6 times/week (n=358)	Almost everyday (n=577)
	Sex, %						
	Men	76.2%	64.2%	83.8%	84.7%	80.7%	80.4%
	Women	23.8%	35.8%	16.2%	15.3%	19.3%	19.6%
	Age, years	51.3 (4.5)	51.6 (4.7)	51.0 (4.2)	51.0 (4.4)	51.2 (4.3)	52.0 (5.1)
	Body Mass Index, kg^2/m	23.6 (2.6)	23.3 (2.7)	23.7 (2.6)	23.8 (2.4)	23.6 (2.2)	23.7 (2.5)
	Systolic blood pressure, mm Hg	121.5 (12.8)	121.2 (13.0)	121.6 (12.6)	121.7 (12.6)	121.0 (12.5)	122.1 (12.3)
	Diastolic blood pressure, mm Hg	76.7 (9.0)	76.4 (9.0)	77.0 (8.9)	77.0 (9.0)	76.0 (8.7)	76.3 (9.0)
	Fasting glucose levels, mg/dL	94.6 (18.5)	93.7 (18.8)	95.1 (18.7)	94.9 (16.6)	95.1 (18.4)	95.7 (20.1)
	Total cholesterol, mg/dL	197.1 (34.5)	197.9 (35.0)	197.1 (34.2)	195.8 (34.3)	192.0 (32.2)	197.4 (34.3)
	Family history of heart disease, stroke or hypertension, %	13.2%	11.7%	13.7%	15.5%	14.3%	12.7%
	Family history of cancer, %	14.5%	12.9%	15.5%	15.7%	15.1%	14.9%
	Family history of diabetes, %	6.4%	5.5%	6.4%	7.3%	7.3%	9.5%
	Smoking status, %						
	Never	56.9%	66.6%	48.7%	53.6%	57.3%	57.4%
	Previously	14.0%	7.0%	17.3%	20.4%	16.8%	21.1%
	Currently	29.1%	26.5%	34.1%	26.0%	26.0%	21.5%
	Alcohol Consumption, %						
	Never	43.7%	56.5%	35.0%	36.4%	40.5%	37.8%
	2-3 times/month	22.8%	17.0%	27.2%	24.9%	24.6%	24.8%
	1-2 times/week	24.8%	17.6%	30.0%	28.3%	26.5%	27.4%
	≥3 times/week	8.8%	8.8%	7.9%	10.4%	8.4%	10.1%

For peer review only

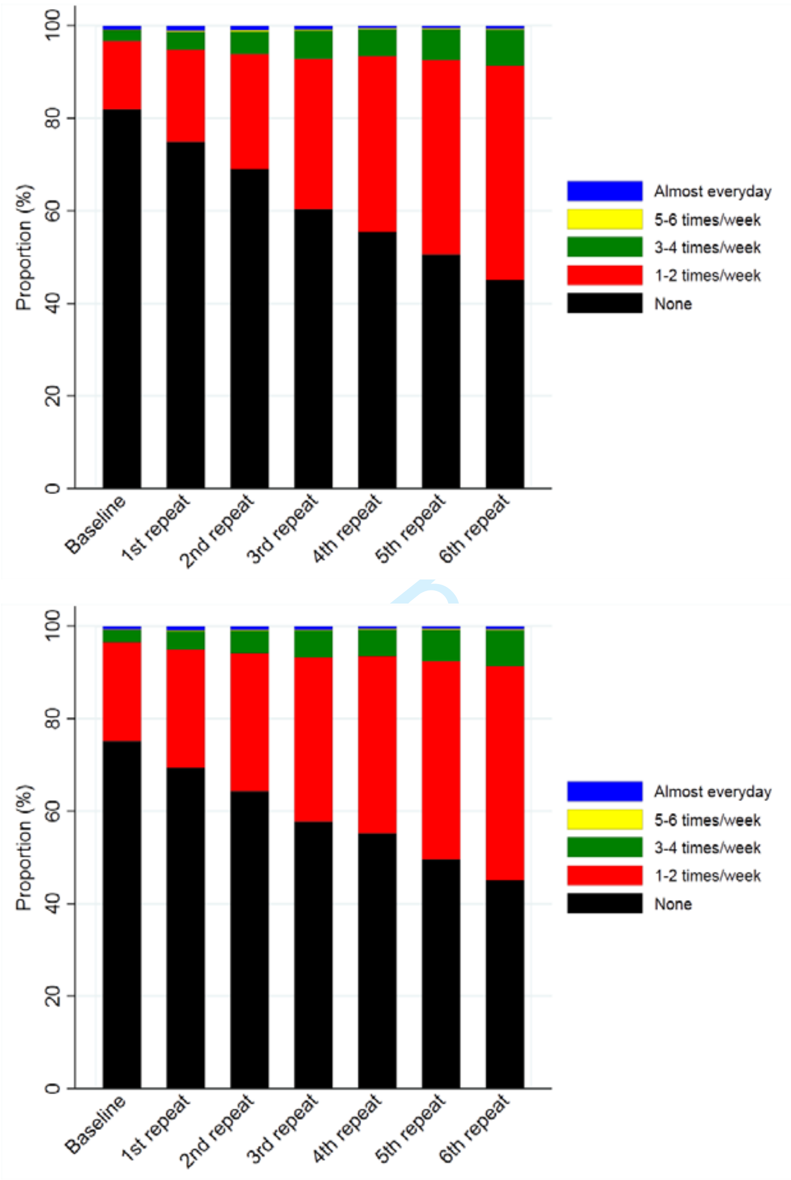

Supplementary Figure 1. Changes in proportions of exercise frequency categories across 7 time points in all 257,854 individuals who provided data at each respective assessment visit (top panel), and 12,302 individuals who provided data from all 7 assessment visits (bottom panel).

For peer review only - http://bmjopen.bmj.com/site/about/guidelines.xhtml

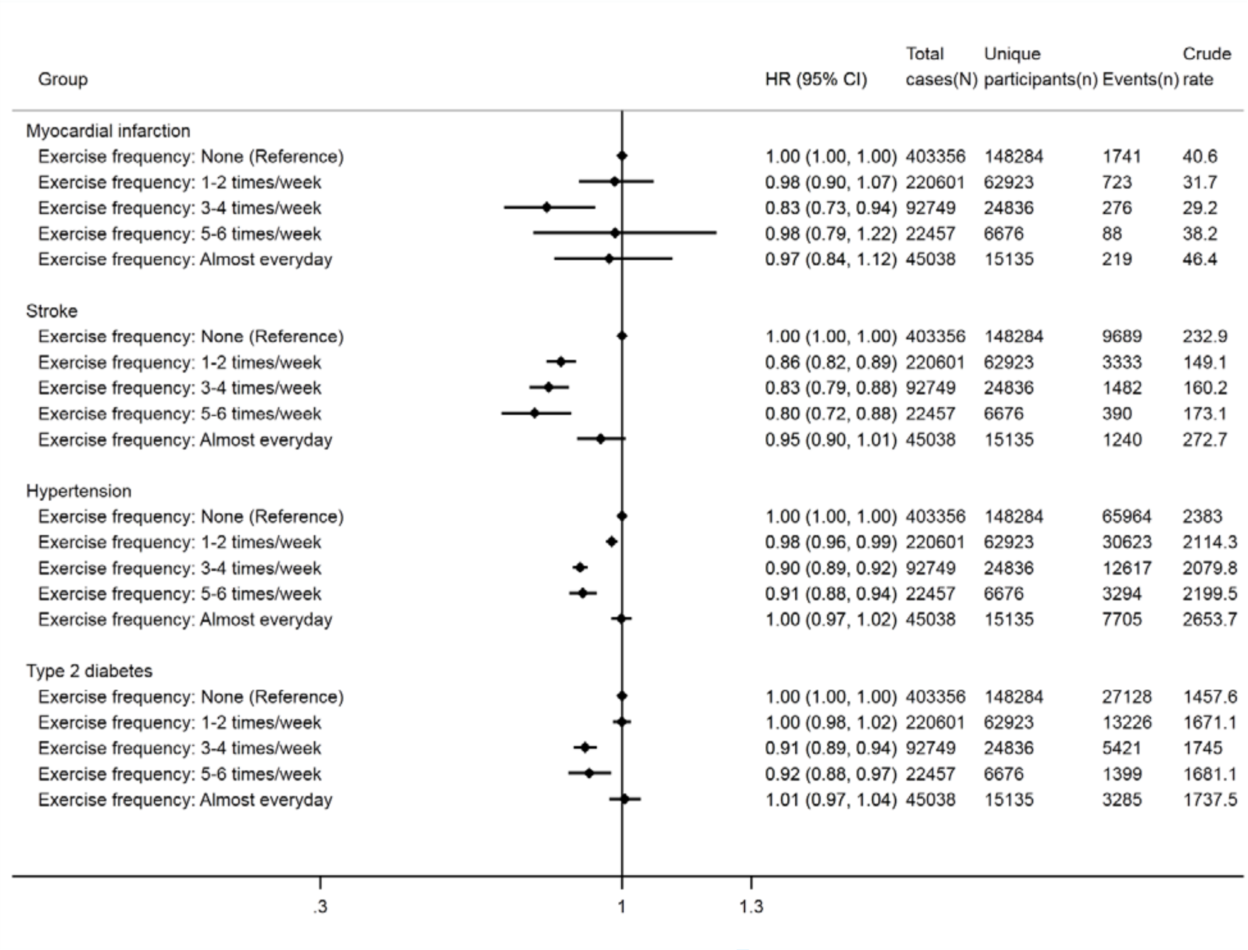
Group
HR (95% CI)
Total cases(N)
Unique participants(n)
Events(n)
Crude rate
Myocardial infarction
Exercise frequency: None (Reference) 1.00 (1.00, 1.00) 403356 148284 1741 40.6
Exercise frequency: 1-2 times/week 0.98 (0.90, 1.07) 220601 62923 723 31.7
Exercise frequency: 3-4 times/week 0.83 (0.73, 0.94) 92749 24836 276 29.2
Exercise frequency: 5-6 times/week 0.98 (0.79, 1.22) 22457 6676 88 38.2
Exercise frequency: Almost everyday 0.97 (0.84, 1.12) 45038 15135 219 46.4
Stroke
Exercise frequency: None (Reference) 1.00 (1.00, 1.00) 403356 148284 9689 232.9
Exercise frequency: 1-2 times/week 0.86 (0.82, 0.89) 220601 62923 3333 149.1
Exercise frequency: 3-4 times/week 0.83 (0.79, 0.88) 92749 24836 1482 160.2
Exercise frequency: 5-6 times/week 0.80 (0.72, 0.88) 22457 6676 390 173.1
Exercise frequency: Almost everyday 0.95 (0.90, 1.01) 45038 15135 1240 272.7
Hypertension
Exercise frequency: None (Reference) 1.00 (1.00, 1.00) 403356 148284 65964 2383
Exercise frequency: 1-2 times/week 0.98 (0.96, 0.99) 220601 62923 30623 2114.3
Exercise frequency: 3-4 times/week 0.90 (0.89, 0.92) 92749 24836 12617 2079.8
Exercise frequency: 5-6 times/week 0.91 (0.88, 0.94) 22457 6676 3294 2199.5
Exercise frequency: Almost everyday 1.00 (0.97, 1.02) 45038 15135 7705 2653.7
Type 2 diabetes
Exercise frequency: None (Reference) 1.00 (1.00, 1.00) 403356 148284 27128 1457.6
Exercise frequency: 1-2 times/week 1.00 (0.98, 1.02) 220601 62923 13226 1671.1
Exercise frequency: 3-4 times/week 0.91 (0.89, 0.94) 92749 24836 5421 1745
Exercise frequency: 5-6 times/week 0.92 (0.88, 0.97) 22457 6676 1399 1681.1
Exercise frequency: Almost everyday 1.01 (0.97, 1.04) 45038 15135 3285 1737.5
.3
1
1.3

w only

Group	HR (95% CI)	Total cases(N)	Unique participants(n)	Events(n)	Crude rate
**Stomach cancer**					
Exercise frequency: None (Reference)	1.00 (1.00, 1.00)	403356	148284	2672	143.6
Exercise frequency: 1-2 times/week	1.01 (0.95, 1.09)	220601	62923	1160	146.6
Exercise frequency: 3-4 times/week	0.94 (0.85, 1.03)	92749	24836	489	157.4
Exercise frequency: 5-6 times/week	0.99 (0.83, 1.17)	22457	6676	139	167
Exercise frequency: Almost everyday	0.93 (0.83, 1.04)	45038	15135	328	173.5
**Colon cancer**					
Exercise frequency: None (Reference)	1.00 (1.00, 1.00)	403356	148284	1424	76.5
Exercise frequency: 1-2 times/week	1.13 (1.03, 1.24)	220601	62923	692	87.4
Exercise frequency: 3-4 times/week	1.12 (0.99, 1.27)	92749	24836	314	101.1
Exercise frequency: 5-6 times/week	1.20 (0.97, 1.48)	22457	6676	90	108.2
Exercise frequency: Almost everyday	1.02 (0.87, 1.18)	45038	15135	191	101
**Rectum cancer**					
Exercise frequency: None (Reference)	1.00 (1.00, 1.00)	403356	148284	809	43.5
Exercise frequency: 1-2 times/week	1.07 (0.94, 1.21)	220601	62923	378	47.8
Exercise frequency: 3-4 times/week	0.95 (0.80, 1.13)	92749	24836	154	49.6
Exercise frequency: 5-6 times/week	1.07 (0.79, 1.43)	22457	6676	46	55.3
Exercise frequency: Almost everyday	1.01 (0.82, 1.23)	45038	15135	107	56.6
**Lung cancer**					
Exercise frequency: None (Reference)	1.00 (1.00, 1.00)	403356	148284	2138	114.9
Exercise frequency: 1-2 times/week	0.97 (0.89, 1.05)	220601	62923	796	100.6
Exercise frequency: 3-4 times/week	0.81 (0.72, 0.91)	92749	24836	307	98.8
Exercise frequency: 5-6 times/week	0.80 (0.65, 1.00)	22457	6676	85	102.1
Exercise frequency: Almost everyday	0.94 (0.83, 1.07)	45038	15135	275	145.4
**Liver cancer**					
Exercise frequency: None (Reference)	1.00 (1.00, 1.00)	403356	148284	1423	76.5
Exercise frequency: 1-2 times/week	1.07 (0.97, 1.17)	220601	62923	680	85.9
Exercise frequency: 3-4 times/week	0.91 (0.80, 1.04)	92749	24836	263	84.7
Exercise frequency: 5-6 times/week	0.99 (0.78, 1.24)	22457	6676	75	90.1
Exercise frequency: Almost everyday	0.99 (0.84, 1.15)	45038	15135	179	94.7
**Pancreas cancer**					
Exercise frequency: None (Reference)	1.00 (1.00, 1.00)	403356	148284	483	26
Exercise frequency: 1-2 times/week	1.05 (0.89, 1.24)	220601	62923	205	25.9
Exercise frequency: 3-4 times/week	1.03 (0.82, 1.29)	92749	24836	92	29.6
Exercise frequency: 5-6 times/week	0.98 (0.65, 1.48)	22457	6676	24	28.8
Exercise frequency: Almost everyday	0.93 (0.71, 1.22)	45038	15135	60	31.7
**Head&Neck cancer**					
Exercise frequency: None (Reference)	1.00 (1.00, 1.00)	403356	148284	377	20.3
Exercise frequency: 1-2 times/week	0.86 (0.71, 1.05)	220601	62923	144	18.2
Exercise frequency: 3-4 times/week	0.96 (0.75, 1.24)	92749	24836	73	23.5
Exercise frequency: 5-6 times/week	0.75 (0.44, 1.25)	22457	6676	15	18
Exercise frequency: Almost everyday	0.96 (0.71, 1.29)	45038	15135	47	24.9
**Kidney cancer**					
Exercise frequency: None (Reference)	1.00 (1.00, 1.00)	403356	148284	301	16.2
Exercise frequency: 1-2 times/week	1.10 (0.90, 1.34)	220601	62923	153	19.3
Exercise frequency: 3-4 times/week	1.20 (0.93, 1.54)	92749	24836	75	24.1
Exercise frequency: 5-6 times/week	0.98 (0.59, 1.62)	22457	6676	16	19.2
Exercise frequency: Almost everyday	1.17 (0.85, 1.60)	45038	15135	44	23.3
**Gallbladder cancer**					
Exercise frequency: None (Reference)	1.00 (1.00, 1.00)	403356	148284	219	11.8
Exercise frequency: 1-2 times/week	0.97 (0.75, 1.25)	220601	62923	83	10.5
Exercise frequency: 3-4 times/week	1.09 (0.79, 1.52)	92749	24836	43	13.8
Exercise frequency: 5-6 times/week	1.48 (0.89, 2.45)	22457	6676	16	19.2
Exercise frequency: Almost everyday	1.32 (0.94, 1.86)	45038	15135	39	20.6
**Esophagus cancer**					
Exercise frequency: None (Reference)	1.00 (1.00, 1.00)	403356	148284	214	11.5
Exercise frequency: 1-2 times/week	0.91 (0.70, 1.19)	220601	62923	75	9.5
Exercise frequency: 3-4 times/week	0.76 (0.51, 1.12)	92749	24836	29	9.3
Exercise frequency: 5-6 times/week	0.56 (0.25, 1.26)	22457	6676	6	7.2
Exercise frequency: Almost everyday	0.93 (0.62, 1.37)	45038	15135	28	14.8

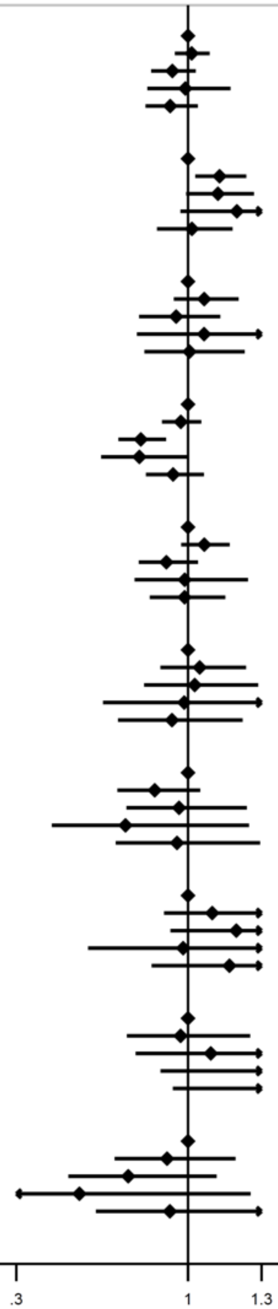

Supplementary Figure 2. Associations of exercise with incident myocardial infarction, stroke, hypertension and Type 2 diabetes (top panel) and various incident cancer outcomes (bottom panel). Note: Cox regression models using age as the underlying timescale were not adjusted for any confounders. Crude rates are per 100,000 person-years. "N" indicates numbers of total observations (i.e. participants who provided repeated measures are treated as separate observations) and "n" indicates numbers of unique participants at baseline. Abbreviations: HR – Hazard Ratio; CI – Confidence Intervals

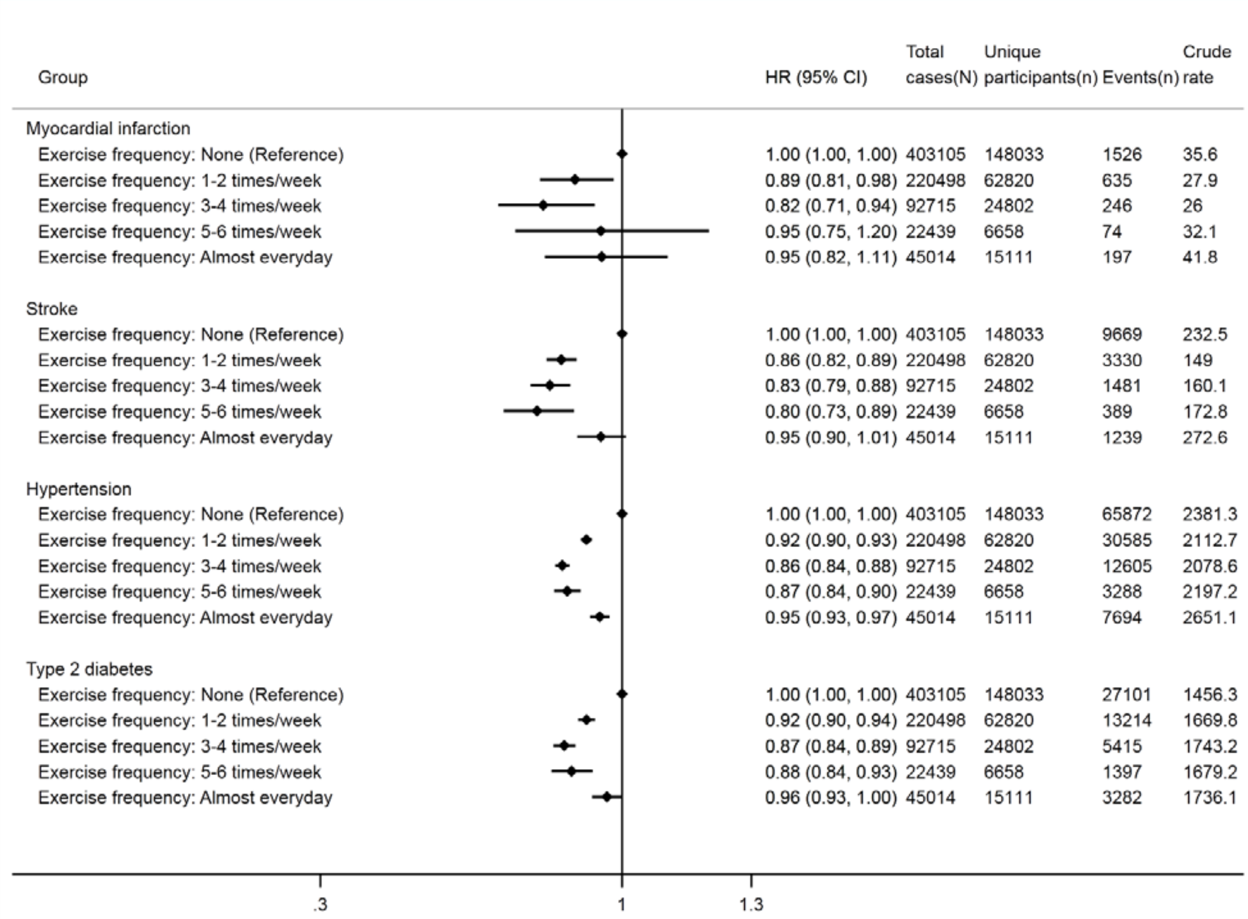

For peer review only - http://bmjopen.bmj.com/site/about/guidelines.xhtml

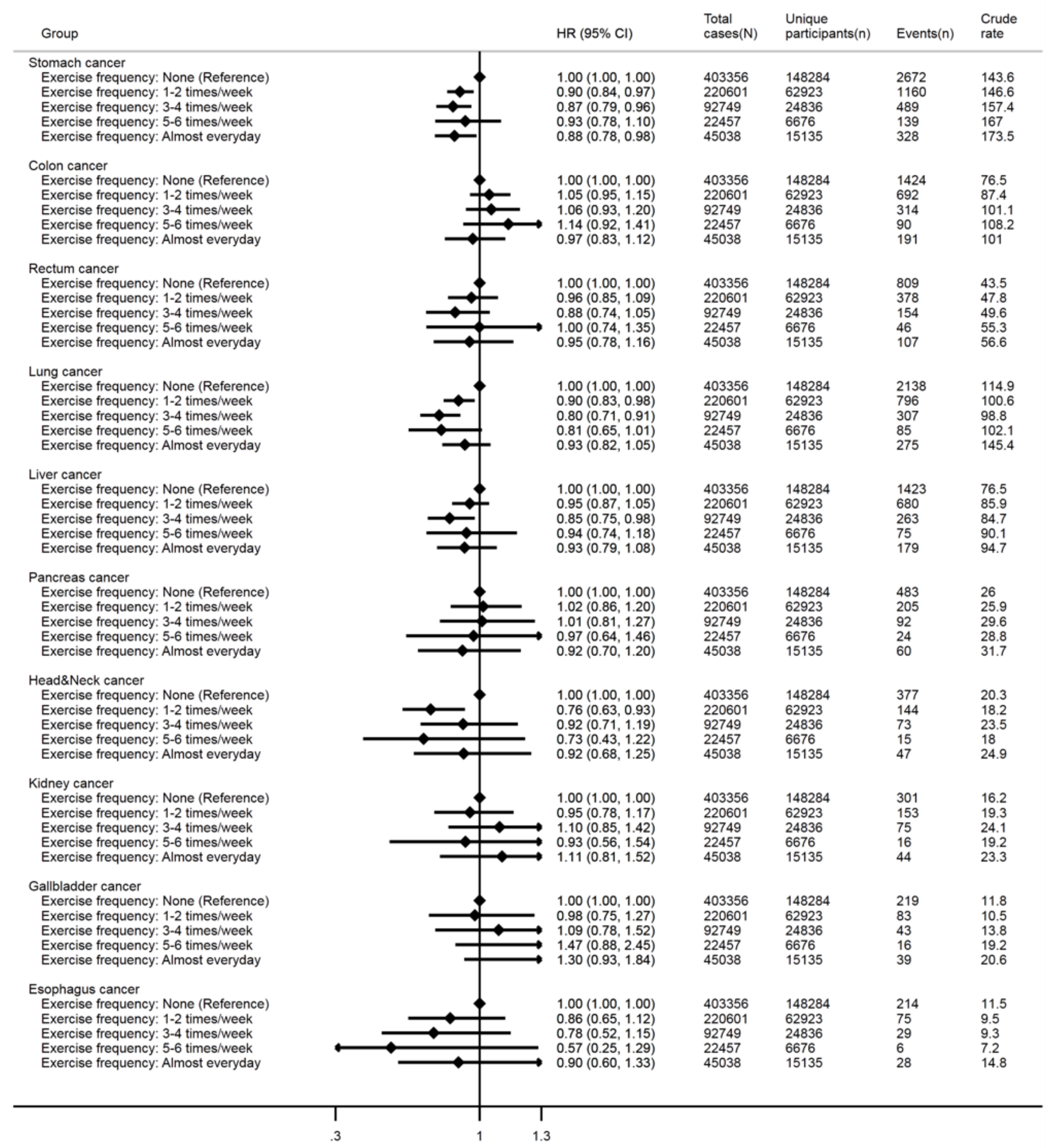

Supplementary Figure 3. Associations of exercise with incident myocardial infarction, stroke, hypertension and Type 2 diabetes (top panel) and various incident cancer outcomes (bottom panel) after excluding data from the first 2-year follow-up period. Note: Cox regression models using age as the underlying timescale were adjusted for sex, body mass index, systolic blood pressure, fasting glucose levels, total cholesterol levels, family history of heart disease, stroke or hypertension (in models for incident myocardial infarction, stroke and hypertension), diabetes (in models for incident Type 2 diabetes) or cancer (in models for incident cancer outcomes), smoking status and alcohol consumption. Crude rates are per 100,000 person-years. “N”

indicates numbers of total observations (i.e. participants who provided repeated measures are treated as separate observations) and “n” indicates numbers of unique participants at baseline. Abbreviations: HR – Hazard Ratio; CI – Confidence Intervals

For peer review only

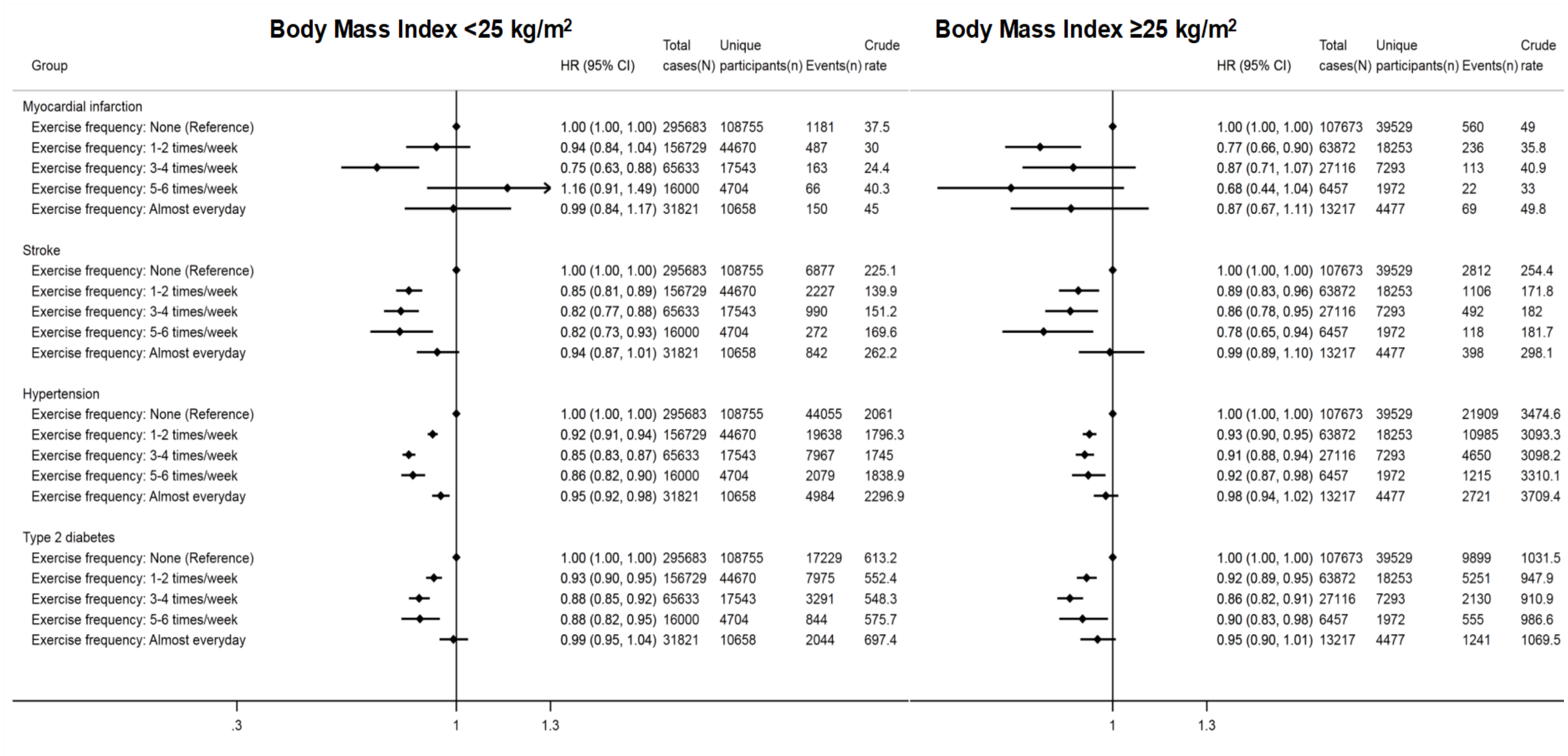
Body Mass Index <25 kg/m2
Group | HR (95% CI) | Total cases(N) | Unique participants(n) | Events(n) | Crude rate
Myocardial infarction
Exercise frequency: None (Reference) 1.00 (1.00, 1.00) 295683 108755 1181 37.5
Exercise frequency: 1-2 times/week 0.94 (0.84, 1.04) 156729 44670 487 30
Exercise frequency: 3-4 times/week 0.75 (0.63, 0.88) 65633 17543 163 24.4
Exercise frequency: 5-6 times/week 1.16 (0.91, 1.49) 16000 4704 66 40.3
Exercise frequency: Almost everyday 0.99 (0.84, 1.17) 31821 10658 150 45
Stroke
Exercise frequency: None (Reference) 1.00 (1.00, 1.00) 295683 108755 6877 225.1
Exercise frequency: 1-2 times/week 0.85 (0.81, 0.89) 156729 44670 2227 139.9
Exercise frequency: 3-4 times/week 0.82 (0.77, 0.88) 65633 17543 990 151.2
Exercise frequency: 5-6 times/week 0.82 (0.73, 0.93) 16000 4704 272 169.6
Exercise frequency: Almost everyday 0.94 (0.87, 1.01) 31821 10658 842 262.2
Hypertension
Exercise frequency: None (Reference) 1.00 (1.00, 1.00) 295683 108755 44055 2061
Exercise frequency: 1-2 times/week 0.92 (0.91, 0.94) 156729 44670 19638 1796.3
Exercise frequency: 3-4 times/week 0.85 (0.83, 0.87) 65633 17543 7967 1745
Exercise frequency: 5-6 times/week 0.86 (0.82, 0.90) 16000 4704 2079 1838.9
Exercise frequency: Almost everyday 0.95 (0.92, 0.98) 31821 10658 4984 2296.9
Type 2 diabetes
Exercise frequency: None (Reference) 1.00 (1.00, 1.00) 295683 108755 17229 613.2
Exercise frequency: 1-2 times/week 0.93 (0.90, 0.95) 156729 44670 7975 552.4
Exercise frequency: 3-4 times/week 0.88 (0.85, 0.92) 65633 17543 3291 548.3
Exercise frequency: 5-6 times/week 0.88 (0.82, 0.95) 16000 4704 844 575.7
Exercise frequency: Almost everyday 0.99 (0.95, 1.04) 31821 10658 2044 697.4
.3 1 1.3
Body Mass Index ≥25 kg/m2
HR (95% CI) | Total cases(N) | Unique participants(n) | Events(n) | Crude rate
1.00 (1.00, 1.00) 107673 39529 560 49
0.77 (0.66, 0.90) 63872 18253 236 35.8
0.87 (0.71, 1.07) 27116 7293 113 40.9
0.68 (0.44, 1.04) 6457 1972 22 33
0.87 (0.67, 1.11) 13217 4477 69 49.8
1.00 (1.00, 1.00) 107673 39529 2812 254.4
0.89 (0.83, 0.96) 63872 18253 1106 171.8
0.86 (0.78, 0.95) 27116 7293 492 182
0.78 (0.65, 0.94) 6457 1972 118 181.7
0.99 (0.89, 1.10) 13217 4477 398 298.1
1.00 (1.00, 1.00) 107673 39529 21909 3474.6
0.93 (0.90, 0.95) 63872 18253 10985 3093.3
0.91 (0.88, 0.94) 27116 7293 4650 3098.2
0.92 (0.87, 0.98) 6457 1972 1215 3310.1
0.98 (0.94, 1.02) 13217 4477 2721 3709.4
1.00 (1.00, 1.00) 107673 39529 9899 1031.5
0.92 (0.89, 0.95) 63872 18253 5251 947.9
0.86 (0.82, 0.91) 27116 7293 2130 910.9
0.90 (0.83, 0.98) 6457 1972 555 986.6
0.95 (0.90, 1.01) 13217 4477 1241 1069.5
1 1.3

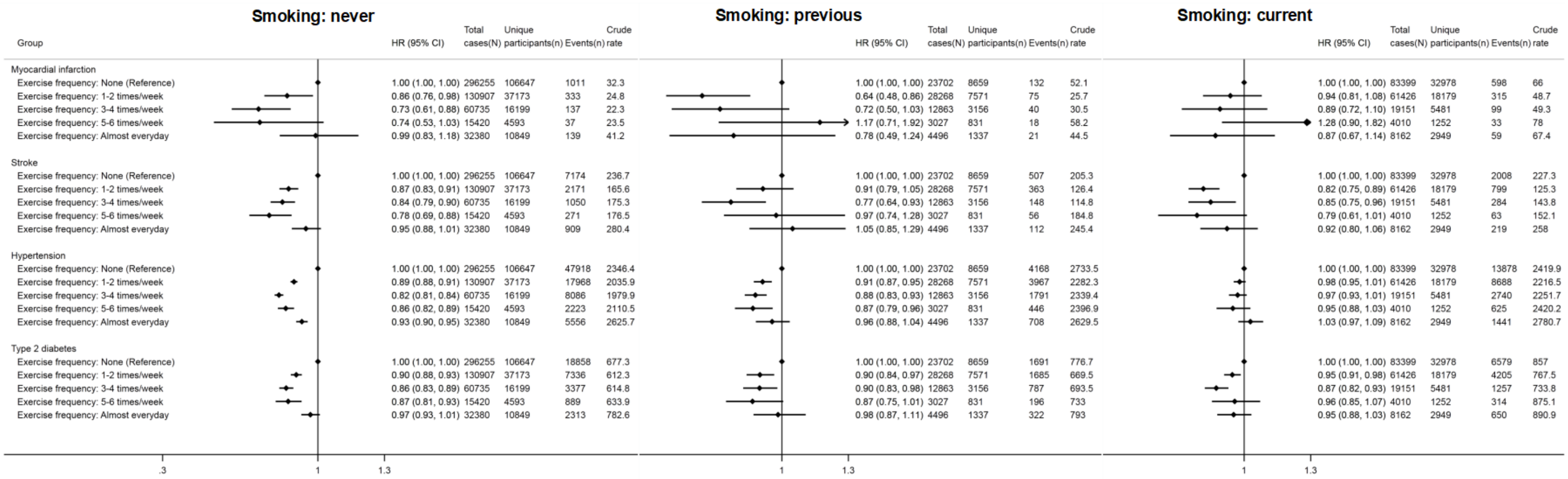

For peer review only - http://bmjopen.bmj.com/site/about/guidelines.xhtml

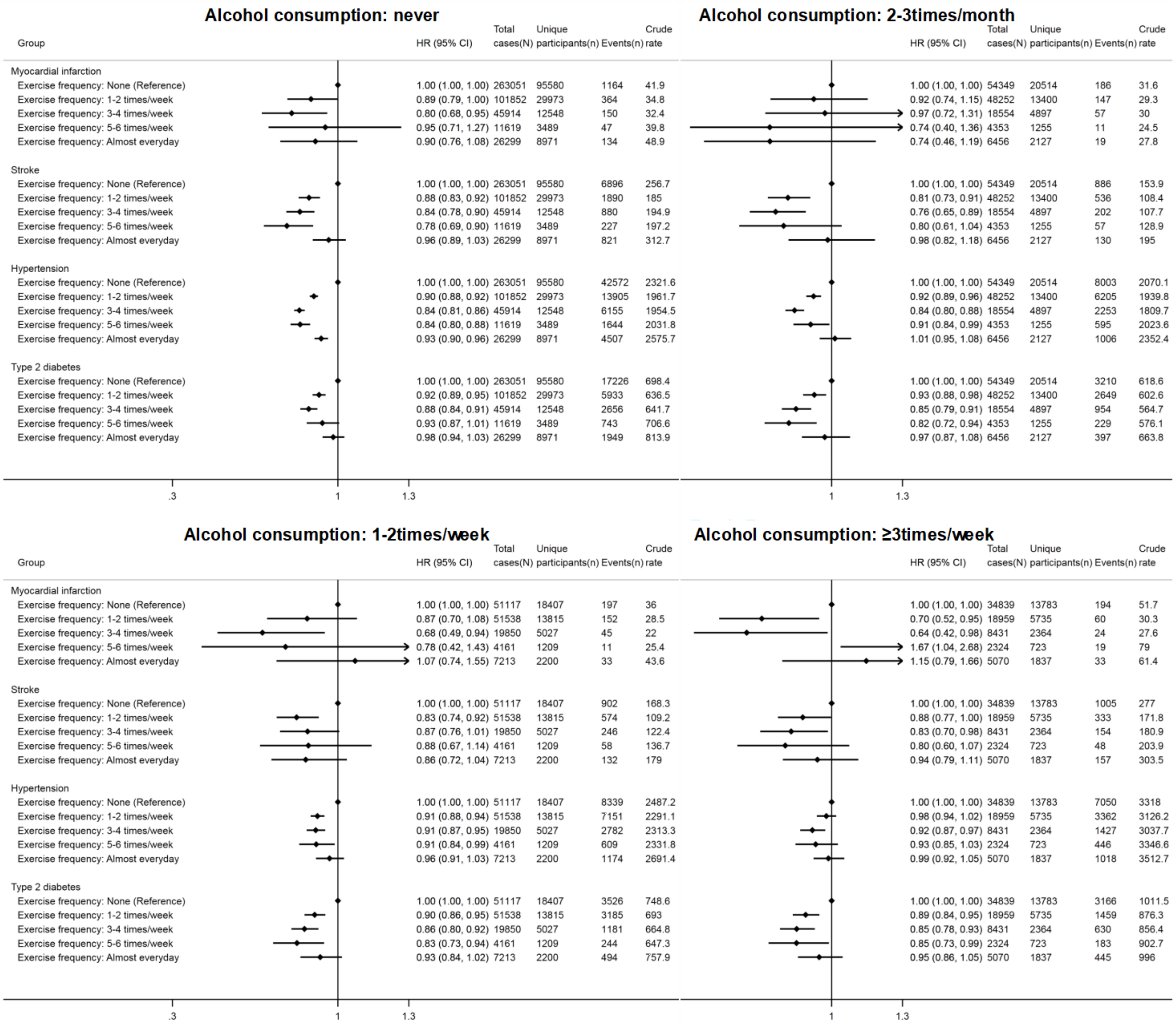
Alcohol consumption: never
Group | HR (95% CI) | Total cases(N) | Unique participants(n) | Events(n) | Crude rate
Myocardial infarction
Exercise frequency: None (Reference) 1.00 (1.00, 1.00) 263051 95580 1164 41.9
Exercise frequency: 1-2 times/week 0.89 (0.79, 1.00) 101852 29973 364 34.8
Exercise frequency: 3-4 times/week 0.80 (0.68, 0.95) 45914 12548 150 32.4
Exercise frequency: 5-6 times/week 0.95 (0.71, 1.27) 11619 3489 47 39.8
Exercise frequency: Almost everyday 0.90 (0.76, 1.08) 26299 8971 134 48.9
Stroke
Exercise frequency: None (Reference) 1.00 (1.00, 1.00) 263051 95580 6896 256.7
Exercise frequency: 1-2 times/week 0.88 (0.83, 0.92) 101852 29973 1890 185
Exercise frequency: 3-4 times/week 0.84 (0.78, 0.90) 45914 12548 880 194.9
Exercise frequency: 5-6 times/week 0.78 (0.69, 0.90) 11619 3489 227 197.2
Exercise frequency: Almost everyday 0.96 (0.89, 1.03) 26299 8971 821 312.7
Hypertension
Exercise frequency: None (Reference) 1.00 (1.00, 1.00) 263051 95580 42572 2321.6
Exercise frequency: 1-2 times/week 0.90 (0.88, 0.92) 101852 29973 13905 1961.7
Exercise frequency: 3-4 times/week 0.84 (0.81, 0.86) 45914 12548 6155 1954.5
Exercise frequency: 5-6 times/week 0.84 (0.80, 0.88) 11619 3489 1644 2031.8
Exercise frequency: Almost everyday 0.93 (0.90, 0.96) 26299 8971 4507 2575.7
Type 2 diabetes
Exercise frequency: None (Reference) 1.00 (1.00, 1.00) 263051 95580 17226 698.4
Exercise frequency: 1-2 times/week 0.92 (0.89, 0.95) 101852 29973 5933 636.5
Exercise frequency: 3-4 times/week 0.88 (0.84, 0.91) 45914 12548 2656 641.7
Exercise frequency: 5-6 times/week 0.93 (0.87, 1.01) 11619 3489 743 706.6
Exercise frequency: Almost everyday 0.98 (0.94, 1.03) 26299 8971 1949 813.9
.3 1 1.3
Alcohol consumption: 2-3times/month
HR (95% CI) | Total cases(N) | Unique participants(n) | Events(n) | Crude rate
1.00 (1.00, 1.00) 54349 20514 186 31.6
0.92 (0.74, 1.15) 48252 13400 147 29.3
0.97 (0.72, 1.31) 18554 4897 57 30
0.74 (0.40, 1.36) 4353 1255 11 24.5
0.74 (0.46, 1.19) 6456 2127 19 27.8
1.00 (1.00, 1.00) 54349 20514 886 153.9
0.81 (0.73, 0.91) 48252 13400 536 108.4
0.76 (0.65, 0.89) 18554 4897 202 107.7
0.80 (0.61, 1.04) 4353 1255 57 128.9
0.98 (0.82, 1.18) 6456 2127 130 195
1.00 (1.00, 1.00) 54349 20514 8003 2070.1
0.92 (0.89, 0.96) 48252 13400 6205 1939.8
0.84 (0.80, 0.88) 18554 4897 2253 1809.7
0.91 (0.84, 0.99) 4353 1255 595 2023.6
1.01 (0.95, 1.08) 6456 2127 1006 2352.4
1.00 (1.00, 1.00) 54349 20514 3210 618.6
0.93 (0.88, 0.98) 48252 13400 2649 602.6
0.85 (0.79, 0.91) 18554 4897 954 564.7
0.82 (0.72, 0.94) 4353 1255 229 576.1
0.97 (0.87, 1.08) 6456 2127 397 663.8
1 1.3
Alcohol consumption: 1-2times/week
Group | HR (95% CI) | Total cases(N) | Unique participants(n) | Events(n) | Crude rate
Myocardial infarction
Exercise frequency: None (Reference) 1.00 (1.00, 1.00) 51117 18407 197 36
Exercise frequency: 1-2 times/week 0.87 (0.70, 1.08) 51538 13815 152 28.5
Exercise frequency: 3-4 times/week 0.68 (0.49, 0.94) 19850 5027 45 22
Exercise frequency: 5-6 times/week 0.78 (0.42, 1.43) 4161 1209 11 25.4
Exercise frequency: Almost everyday 1.07 (0.74, 1.55) 7213 2200 33 43.6
Stroke
Exercise frequency: None (Reference) 1.00 (1.00, 1.00) 51117 18407 902 168.3
Exercise frequency: 1-2 times/week 0.83 (0.74, 0.92) 51538 13815 574 109.2
Exercise frequency: 3-4 times/week 0.87 (0.76, 1.01) 19850 5027 246 122.4
Exercise frequency: 5-6 times/week 0.88 (0.67, 1.14) 4161 1209 58 136.7
Exercise frequency: Almost everyday 0.86 (0.72, 1.04) 7213 2200 132 179
Hypertension
Exercise frequency: None (Reference) 1.00 (1.00, 1.00) 51117 18407 8339 2487.2
Exercise frequency: 1-2 times/week 0.91 (0.88, 0.94) 51538 13815 7151 2291.1
Exercise frequency: 3-4 times/week 0.91 (0.87, 0.95) 19850 5027 2782 2313.3
Exercise frequency: 5-6 times/week 0.91 (0.84, 0.99) 4161 1209 609 2331.8
Exercise frequency: Almost everyday 0.96 (0.91, 1.03) 7213 2200 1174 2691.4
Type 2 diabetes
Exercise frequency: None (Reference) 1.00 (1.00, 1.00) 51117 18407 3526 748.6
Exercise frequency: 1-2 times/week 0.90 (0.86, 0.95) 51538 13815 3185 693
Exercise frequency: 3-4 times/week 0.86 (0.80, 0.92) 19850 5027 1181 664.8
Exercise frequency: 5-6 times/week 0.83 (0.73, 0.94) 4161 1209 244 647.3
Exercise frequency: Almost everyday 0.93 (0.84, 1.02) 7213 2200 494 757.9
.3 1 1.3
Alcohol consumption: ≥3times/week
HR (95% CI) | Total cases(N) | Unique participants(n) | Events(n) | Crude rate
1.00 (1.00, 1.00) 34839 13783 194 51.7
0.70 (0.52, 0.95) 18959 5735 60 30.3
0.64 (0.42, 0.98) 8431 2364 24 27.6
1.67 (1.04, 2.68) 2324 723 19 79
1.15 (0.79, 1.66) 5070 1837 33 61.4
1.00 (1.00, 1.00) 34839 13783 1005 277
0.88 (0.77, 1.00) 18959 5735 333 171.8
0.83 (0.70, 0.98) 8431 2364 154 180.9
0.80 (0.60, 1.07) 2324 723 48 203.9
0.94 (0.79, 1.11) 5070 1837 157 303.5
1.00 (1.00, 1.00) 34839 13783 7050 3318
0.98 (0.94, 1.02) 18959 5735 3362 3126.2
0.92 (0.87, 0.97) 8431 2364 1427 3037.7
0.93 (0.85, 1.03) 2324 723 446 3346.6
0.99 (0.92, 1.05) 5070 1837 1018 3512.7
1.00 (1.00, 1.00) 34839 13783 3166 1011.5
0.89 (0.84, 0.95) 18959 5735 1459 876.3
0.85 (0.78, 0.93) 8431 2364 630 856.4
0.85 (0.73, 0.99) 2324 723 183 902.7
0.95 (0.86, 1.05) 5070 1837 445 996
1 1.3

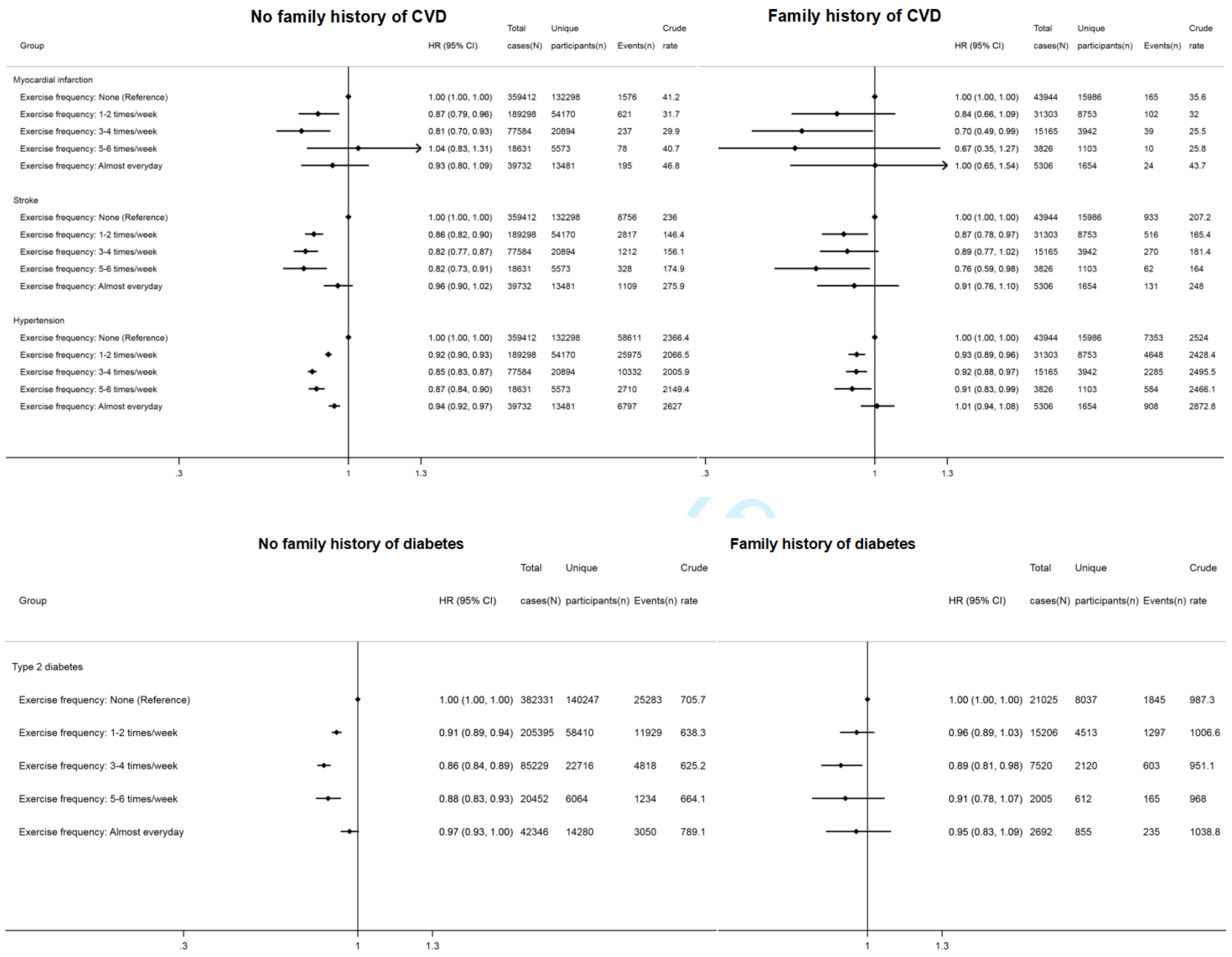
No family history of CVD
Group
HR (95% CI)
Total cases(N)
Unique participants(n)
Events(n)
Crude rate
Myocardial infarction
Exercise frequency: None (Reference) 1.00 (1.00, 1.00) 359412 132298 1576 41.2
Exercise frequency: 1-2 times/week 0.87 (0.79, 0.96) 189298 54170 621 31.7
Exercise frequency: 3-4 times/week 0.81 (0.70, 0.93) 77584 20894 237 29.9
Exercise frequency: 5-6 times/week 1.04 (0.83, 1.31) 18631 5573 78 40.7
Exercise frequency: Almost everyday 0.93 (0.80, 1.09) 39732 13481 195 46.8
Stroke
Exercise frequency: None (Reference) 1.00 (1.00, 1.00) 359412 132298 8756 236
Exercise frequency: 1-2 times/week 0.86 (0.82, 0.90) 189298 54170 2817 146.4
Exercise frequency: 3-4 times/week 0.82 (0.77, 0.87) 77584 20894 1212 156.1
Exercise frequency: 5-6 times/week 0.82 (0.73, 0.91) 18631 5573 328 174.9
Exercise frequency: Almost everyday 0.96 (0.90, 1.02) 39732 13481 1109 275.9
Hypertension
Exercise frequency: None (Reference) 1.00 (1.00, 1.00) 359412 132298 58611 2366.4
Exercise frequency: 1-2 times/week 0.92 (0.90, 0.93) 189298 54170 25975 2066.5
Exercise frequency: 3-4 times/week 0.85 (0.83, 0.87) 77584 20894 10332 2005.9
Exercise frequency: 5-6 times/week 0.87 (0.84, 0.90) 18631 5573 2710 2149.4
Exercise frequency: Almost everyday 0.94 (0.92, 0.97) 39732 13481 6797 2627
.3
1
1.3
Family history of CVD
HR (95% CI)
Total cases(N)
Unique participants(n)
Events(n)
Crude rate
1.00 (1.00, 1.00) 43944 15986 165 35.6
0.84 (0.66, 1.09) 31303 8753 102 32
0.70 (0.49, 0.99) 15165 3942 39 25.5
0.67 (0.35, 1.27) 3826 1103 10 25.8
1.00 (0.65, 1.54) 5306 1654 24 43.7
1.00 (1.00, 1.00) 43944 15986 933 207.2
0.87 (0.78, 0.97) 31303 8753 516 165.4
0.89 (0.77, 1.02) 15165 3942 270 181.4
0.76 (0.59, 0.98) 3826 1103 62 164
0.91 (0.76, 1.10) 5306 1654 131 248
1.00 (1.00, 1.00) 43944 15986 7353 2524
0.93 (0.89, 0.96) 31303 8753 4648 2428.4
0.92 (0.88, 0.97) 15165 3942 2285 2495.5
0.91 (0.83, 0.99) 3826 1103 584 2466.1
1.01 (0.94, 1.08) 5306 1654 908 2872.8
.3
1
1.3
No family history of diabetes
Group
HR (95% CI)
Total cases(N)
Unique participants(n)
Events(n)
Crude rate
Type 2 diabetes
Exercise frequency: None (Reference) 1.00 (1.00, 1.00) 382331 140247 25283 705.7
Exercise frequency: 1-2 times/week 0.91 (0.89, 0.94) 205395 58410 11929 638.3
Exercise frequency: 3-4 times/week 0.86 (0.84, 0.89) 85229 22716 4818 625.2
Exercise frequency: 5-6 times/week 0.88 (0.83, 0.93) 20452 6064 1234 664.1
Exercise frequency: Almost everyday 0.97 (0.93, 1.00) 42346 14280 3050 789.1
.3
1
1.3
Family history of diabetes
HR (95% CI)
Total cases(N)
Unique participants(n)
Events(n)
Crude rate
1.00 (1.00, 1.00) 21025 8037 1845 987.3
0.96 (0.89, 1.03) 15206 4513 1297 1006.6
0.89 (0.81, 0.98) 7520 2120 603 951.1
0.91 (0.78, 1.07) 2005 612 165 968
0.95 (0.83, 1.09) 2692 855 235 1038.8
1
1.3

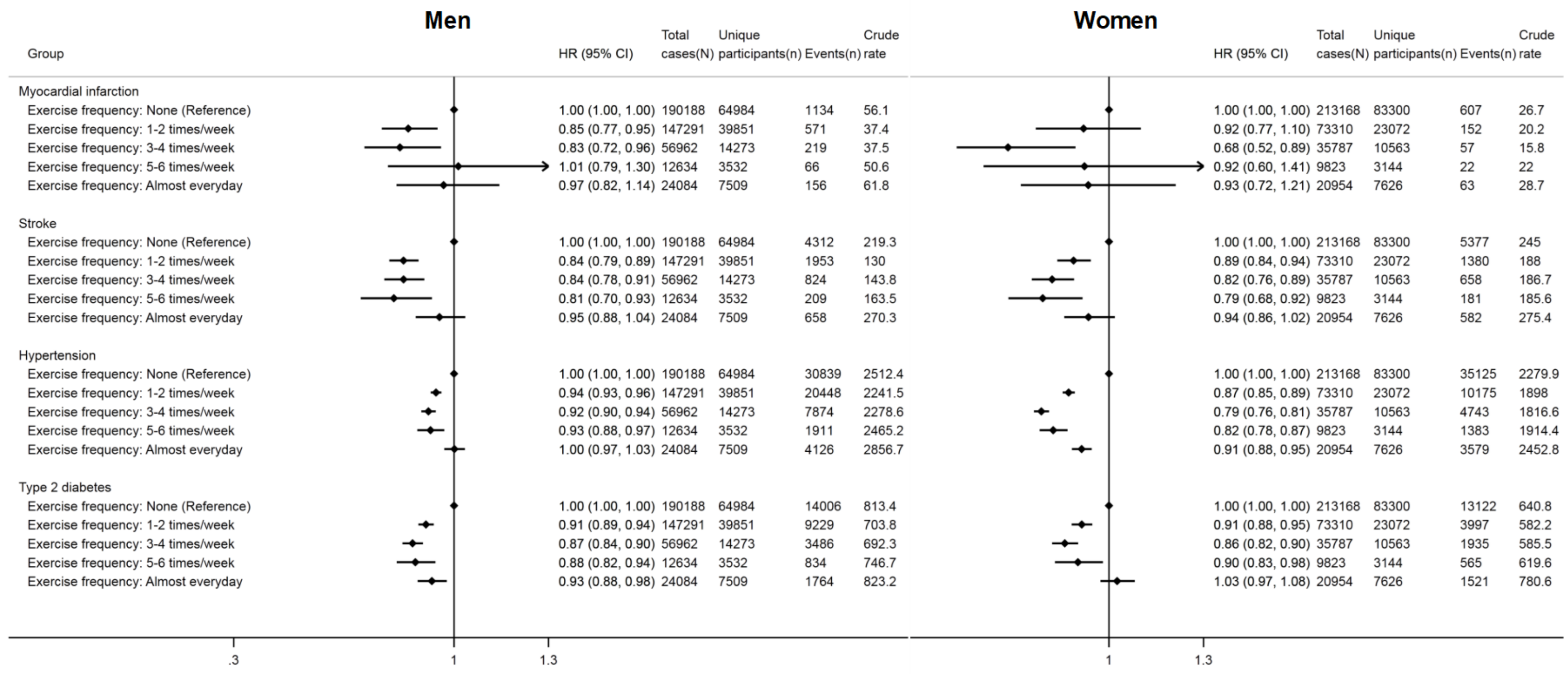

Men

Group	HR (95% CI)	Total cases(N)	Unique participants(n)	Events(n)	Crude rate
Myocardial infarction					
Exercise frequency: None (Reference)	1.00 (1.00, 1.00)	190188	64984	1134	56.1
Exercise frequency: 1-2 times/week	0.85 (0.77, 0.95)	147291	39851	571	37.4
Exercise frequency: 3-4 times/week	0.83 (0.72, 0.96)	56962	14273	219	37.5
Exercise frequency: 5-6 times/week	1.01 (0.79, 1.30)	12634	3532	66	50.6
Exercise frequency: Almost everyday	0.97 (0.82, 1.14)	24084	7509	156	61.8
Stroke					
Exercise frequency: None (Reference)	1.00 (1.00, 1.00)	190188	64984	4312	219.3
Exercise frequency: 1-2 times/week	0.84 (0.79, 0.89)	147291	39851	1953	130
Exercise frequency: 3-4 times/week	0.84 (0.78, 0.91)	56962	14273	824	143.8
Exercise frequency: 5-6 times/week	0.81 (0.70, 0.93)	12634	3532	209	163.5
Exercise frequency: Almost everyday	0.95 (0.88, 1.04)	24084	7509	658	270.3
Hypertension					
Exercise frequency: None (Reference)	1.00 (1.00, 1.00)	190188	64984	30839	2512.4
Exercise frequency: 1-2 times/week	0.94 (0.93, 0.96)	147291	39851	20448	2241.5
Exercise frequency: 3-4 times/week	0.92 (0.90, 0.94)	56962	14273	7874	2278.6
Exercise frequency: 5-6 times/week	0.93 (0.88, 0.97)	12634	3532	1911	2465.2
Exercise frequency: Almost everyday	1.00 (0.97, 1.03)	24084	7509	4126	2856.7
Type 2 diabetes					
Exercise frequency: None (Reference)	1.00 (1.00, 1.00)	190188	64984	14006	813.4
Exercise frequency: 1-2 times/week	0.91 (0.89, 0.94)	147291	39851	9229	703.8
Exercise frequency: 3-4 times/week	0.87 (0.84, 0.90)	56962	14273	3486	692.3
Exercise frequency: 5-6 times/week	0.88 (0.82, 0.94)	12634	3532	834	746.7
Exercise frequency: Almost everyday	0.93 (0.88, 0.98)	24084	7509	1764	823.2

Women

Group	HR (95% CI)	Total cases(N)	Unique participants(n)	Events(n)	Crude rate
Myocardial infarction					
Exercise frequency: None (Reference)	1.00 (1.00, 1.00)	213168	83300	607	26.7
Exercise frequency: 1-2 times/week	0.92 (0.77, 1.10)	73310	23072	152	20.2
Exercise frequency: 3-4 times/week	0.68 (0.52, 0.89)	35787	10563	57	15.8
Exercise frequency: 5-6 times/week	0.92 (0.60, 1.41)	9823	3144	22	22
Exercise frequency: Almost everyday	0.93 (0.72, 1.21)	20954	7626	63	28.7
Stroke					
Exercise frequency: None (Reference)	1.00 (1.00, 1.00)	213168	83300	5377	245
Exercise frequency: 1-2 times/week	0.89 (0.84, 0.94)	73310	23072	1380	188
Exercise frequency: 3-4 times/week	0.82 (0.76, 0.89)	35787	10563	658	186.7
Exercise frequency: 5-6 times/week	0.79 (0.68, 0.92)	9823	3144	181	185.6
Exercise frequency: Almost everyday	0.94 (0.86, 1.02)	20954	7626	582	275.4
Hypertension					
Exercise frequency: None (Reference)	1.00 (1.00, 1.00)	213168	83300	35125	2279.9
Exercise frequency: 1-2 times/week	0.87 (0.85, 0.89)	73310	23072	10175	1898
Exercise frequency: 3-4 times/week	0.79 (0.76, 0.81)	35787	10563	4743	1816.6
Exercise frequency: 5-6 times/week	0.82 (0.78, 0.87)	9823	3144	1383	1914.4
Exercise frequency: Almost everyday	0.91 (0.88, 0.95)	20954	7626	3579	2452.8
Type 2 diabetes					
Exercise frequency: None (Reference)	1.00 (1.00, 1.00)	213168	83300	13122	640.8
Exercise frequency: 1-2 times/week	0.91 (0.88, 0.95)	73310	23072	3997	582.2
Exercise frequency: 3-4 times/week	0.86 (0.82, 0.90)	35787	10563	1935	585.5
Exercise frequency: 5-6 times/week	0.90 (0.83, 0.98)	9823	3144	565	619.6
Exercise frequency: Almost everyday	1.03 (0.97, 1.08)	20954	7626	1521	780.6

Supplementary Figure 4. Results from running Cox regression models examining effect modification of sex, body mass index, smoking, alcohol consumption, and family history of disease in the associations between exercise frequency and incident myocardial infarction, stroke, hypertension and type 2 diabetes. Note: Cox regression models using age as the underlying timescale were adjusted for sex [not in models for effect modification by sex], body mass index [not in models for effect modification by body mass index], systolic blood pressure, fasting glucose levels, total cholesterol levels, family history [not in models for effect modification by family history of respective disease] of heart disease/stroke/hypertension (in models for myocardial infarction, stroke and hypertension), or diabetes (in models for Type 2 diabetes), smoking status [not in models for effect modification by smoking status] and alcohol consumption [not in models for effect modification by alcohol consumption]. Crude rates are per 100,000 person-years. “N” indicates numbers of total observations (i.e. participants who provided repeated measures are treated as separate observations) and “n” indicates numbers of unique participants at baseline. P-values for multiplicative terms – myocardial infarction (p-value = 0.235), stroke (p-value = 0.363), hypertension (p-value = 0.050) and type 2 diabetes (p-value = 0.196) by body mass index;

myocardial infarction (p-value = 0.643), stroke (p-value = 0.661), hypertension (p-value <0.001) and type 2 diabetes (p-value = 0.980) by smoking; myocardial infarction (p-value = 0.300), stroke (p-value = 0.607), hypertension (p-value <0.001) and type 2 diabetes (p-value = 0.081) by alcohol consumption; myocardial infarction (p-value = 0.590), stroke (p-value = 0.505), and hypertension (p-value = 0.029) by family history of cardiovascular disease; type 2 diabetes (p-value = 0.952) by family history of diabetes; and myocardial infarction (p-value = 0.334), stroke (p-value = 0.818), hypertension (p-value <0.001) and type 2 diabetes (p-value = 0.012) by sex.
Abbreviations: HR – Hazard Ratio; CI – Confidence Intervals

For peer review only

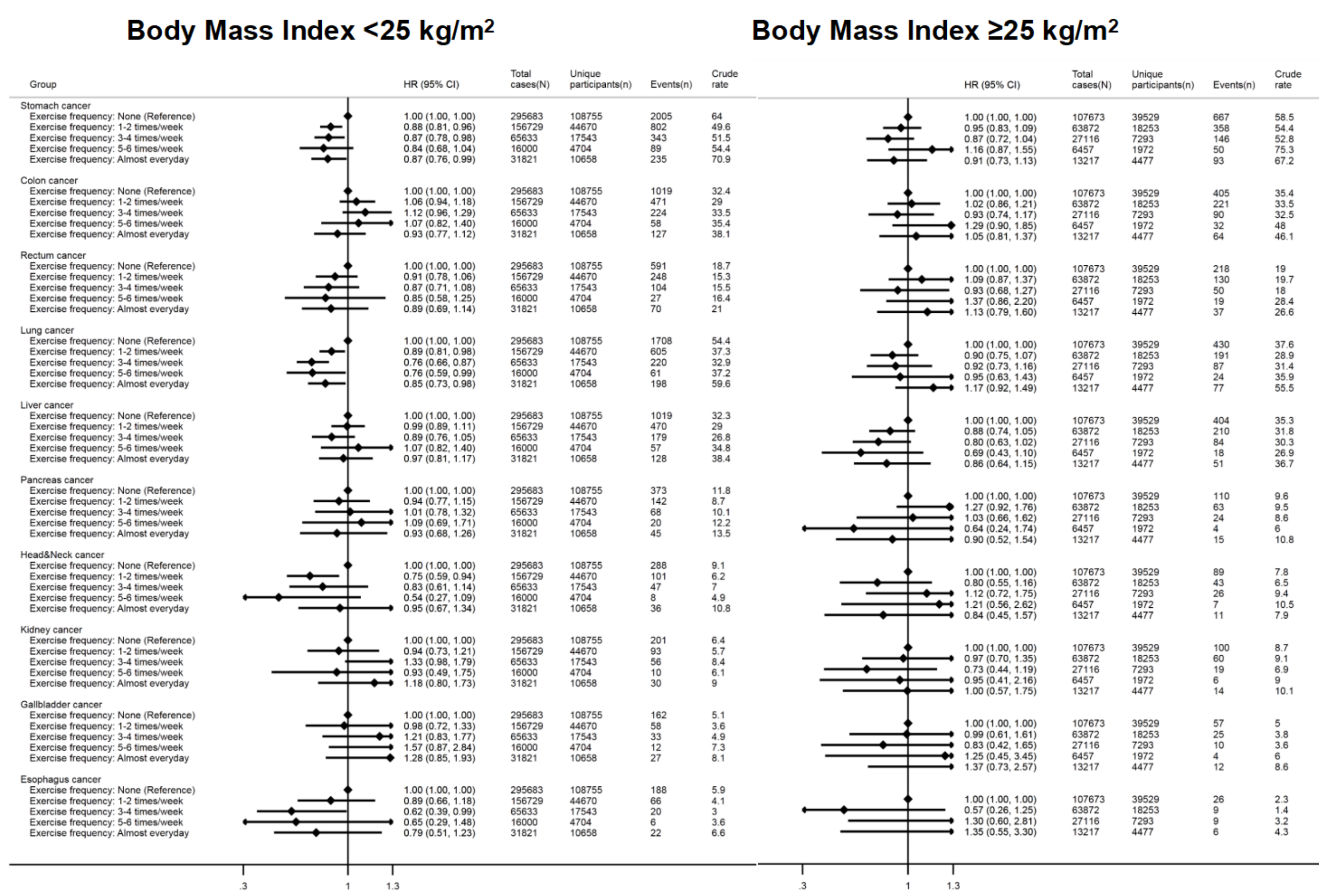
Body Mass Index <25 kg/m2
Group | HR (95% CI) | Total cases(N) | Unique participants(n) | Events(n) | Crude rate
Stomach cancer
Exercise frequency: None (Reference) 1.00 (1.00, 1.00) 295683 108755 2005 64
Exercise frequency: 1-2 times/week 0.88 (0.81, 0.96) 156729 44670 802 49.6
Exercise frequency: 3-4 times/week 0.87 (0.78, 0.98) 65633 17543 343 51.5
Exercise frequency: 5-6 times/week 0.84 (0.68, 1.04) 16000 4704 89 54.4
Exercise frequency: Almost everyday 0.87 (0.76, 0.99) 31821 10658 235 70.9
Colon cancer
Exercise frequency: None (Reference) 1.00 (1.00, 1.00) 295683 108755 1019 32.4
Exercise frequency: 1-2 times/week 1.06 (0.94, 1.18) 156729 44670 471 29
Exercise frequency: 3-4 times/week 1.12 (0.96, 1.29) 65633 17543 224 33.5
Exercise frequency: 5-6 times/week 1.07 (0.82, 1.40) 16000 4704 58 35.4
Exercise frequency: Almost everyday 0.93 (0.77, 1.12) 31821 10658 127 38.1
Rectum cancer
Exercise frequency: None (Reference) 1.00 (1.00, 1.00) 295683 108755 591 18.7
Exercise frequency: 1-2 times/week 0.91 (0.78, 1.06) 156729 44670 248 15.3
Exercise frequency: 3-4 times/week 0.87 (0.71, 1.08) 65633 17543 104 15.5
Exercise frequency: 5-6 times/week 0.85 (0.58, 1.25) 16000 4704 27 16.4
Exercise frequency: Almost everyday 0.89 (0.69, 1.14) 31821 10658 70 21
Lung cancer
Exercise frequency: None (Reference) 1.00 (1.00, 1.00) 295683 108755 1708 54.4
Exercise frequency: 1-2 times/week 0.89 (0.81, 0.98) 156729 44670 605 37.3
Exercise frequency: 3-4 times/week 0.76 (0.66, 0.87) 65633 17543 220 32.9
Exercise frequency: 5-6 times/week 0.76 (0.59, 0.99) 16000 4704 61 37.2
Exercise frequency: Almost everyday 0.85 (0.73, 0.98) 31821 10658 198 59.6
Liver cancer
Exercise frequency: None (Reference) 1.00 (1.00, 1.00) 295683 108755 1019 32.3
Exercise frequency: 1-2 times/week 0.99 (0.89, 1.11) 156729 44670 470 29
Exercise frequency: 3-4 times/week 0.89 (0.76, 1.05) 65633 17543 179 26.8
Exercise frequency: 5-6 times/week 1.07 (0.82, 1.40) 16000 4704 57 34.8
Exercise frequency: Almost everyday 0.97 (0.81, 1.17) 31821 10658 128 38.4
Pancreas cancer
Exercise frequency: None (Reference) 1.00 (1.00, 1.00) 295683 108755 373 11.8
Exercise frequency: 1-2 times/week 0.94 (0.77, 1.15) 156729 44670 142 8.7
Exercise frequency: 3-4 times/week 1.01 (0.78, 1.32) 65633 17543 68 10.1
Exercise frequency: 5-6 times/week 1.09 (0.69, 1.71) 16000 4704 20 12.2
Exercise frequency: Almost everyday 0.93 (0.68, 1.26) 31821 10658 45 13.5
Head&Neck cancer
Exercise frequency: None (Reference) 1.00 (1.00, 1.00) 295683 108755 288 9.1
Exercise frequency: 1-2 times/week 0.75 (0.59, 0.94) 156729 44670 101 6.2
Exercise frequency: 3-4 times/week 0.83 (0.61, 1.14) 65633 17543 47 7
Exercise frequency: 5-6 times/week 0.54 (0.27, 1.09) 16000 4704 8 4.9
Exercise frequency: Almost everyday 0.95 (0.67, 1.34) 31821 10658 36 10.8
Kidney cancer
Exercise frequency: None (Reference) 1.00 (1.00, 1.00) 295683 108755 201 6.4
Exercise frequency: 1-2 times/week 0.94 (0.73, 1.21) 156729 44670 93 5.7
Exercise frequency: 3-4 times/week 1.33 (0.98, 1.79) 65633 17543 56 8.4
Exercise frequency: 5-6 times/week 0.93 (0.49, 1.75) 16000 4704 10 6.1
Exercise frequency: Almost everyday 1.18 (0.80, 1.73) 31821 10658 30 9
Gallbladder cancer
Exercise frequency: None (Reference) 1.00 (1.00, 1.00) 295683 108755 162 5.1
Exercise frequency: 1-2 times/week 0.98 (0.72, 1.33) 156729 44670 58 3.6
Exercise frequency: 3-4 times/week 1.21 (0.83, 1.77) 65633 17543 33 4.9
Exercise frequency: 5-6 times/week 1.57 (0.87, 2.84) 16000 4704 12 7.3
Exercise frequency: Almost everyday 1.28 (0.85, 1.93) 31821 10658 27 8.1
Esophagus cancer
Exercise frequency: None (Reference) 1.00 (1.00, 1.00) 295683 108755 188 5.9
Exercise frequency: 1-2 times/week 0.89 (0.66, 1.18) 156729 44670 66 4.1
Exercise frequency: 3-4 times/week 0.62 (0.39, 0.99) 65633 17543 20 3
Exercise frequency: 5-6 times/week 0.65 (0.29, 1.48) 16000 4704 6 3.6
Exercise frequency: Almost everyday 0.79 (0.51, 1.23) 31821 10658 22 6.6
.3 1 1.3
Body Mass Index ≥25 kg/m2
HR (95% CI) | Total cases(N) | Unique participants(n) | Events(n) | Crude rate
1.00 (1.00, 1.00) 107673 39529 667 58.5
0.95 (0.83, 1.09) 63872 18253 358 54.4
0.87 (0.72, 1.04) 27116 7293 146 52.8
1.16 (0.87, 1.55) 6457 1972 50 75.3
0.91 (0.73, 1.13) 13217 4477 93 67.2
1.00 (1.00, 1.00) 107673 39529 405 35.4
1.02 (0.86, 1.21) 63872 18253 221 33.5
0.93 (0.74, 1.17) 27116 7293 90 32.5
1.29 (0.90, 1.85) 6457 1972 32 48
1.05 (0.81, 1.37) 13217 4477 64 46.1
1.00 (1.00, 1.00) 107673 39529 218 19
1.09 (0.87, 1.37) 63872 18253 130 19.7
0.93 (0.68, 1.27) 27116 7293 50 18
1.37 (0.86, 2.20) 6457 1972 19 28.4
1.13 (0.79, 1.60) 13217 4477 37 26.6
1.00 (1.00, 1.00) 107673 39529 430 37.6
0.90 (0.75, 1.07) 63872 18253 191 28.9
0.92 (0.73, 1.16) 27116 7293 87 31.4
0.95 (0.63, 1.43) 6457 1972 24 35.9
1.17 (0.92, 1.49) 13217 4477 77 55.5
1.00 (1.00, 1.00) 107673 39529 404 35.3
0.88 (0.74, 1.05) 63872 18253 210 31.8
0.80 (0.63, 1.02) 27116 7293 84 30.3
0.69 (0.43, 1.10) 6457 1972 18 26.9
0.86 (0.64, 1.15) 13217 4477 51 36.7
1.00 (1.00, 1.00) 107673 39529 110 9.6
1.27 (0.92, 1.76) 63872 18253 63 9.5
1.03 (0.66, 1.62) 27116 7293 24 8.6
0.64 (0.24, 1.74) 6457 1972 4 6
0.90 (0.52, 1.54) 13217 4477 15 10.8
1.00 (1.00, 1.00) 107673 39529 89 7.8
0.80 (0.55, 1.16) 63872 18253 43 6.5
1.12 (0.72, 1.75) 27116 7293 26 9.4
1.21 (0.56, 2.62) 6457 1972 7 10.5
0.84 (0.45, 1.57) 13217 4477 11 7.9
1.00 (1.00, 1.00) 107673 39529 100 8.7
0.97 (0.70, 1.35) 63872 18253 60 9.1
0.73 (0.44, 1.19) 27116 7293 19 6.9
0.95 (0.41, 2.16) 6457 1972 6 9
1.00 (0.57, 1.75) 13217 4477 14 10.1
1.00 (1.00, 1.00) 107673 39529 57 5
0.99 (0.61, 1.61) 63872 18253 25 3.8
0.83 (0.42, 1.65) 27116 7293 10 3.6
1.25 (0.45, 3.45) 6457 1972 4 6
1.37 (0.73, 2.57) 13217 4477 12 8.6
1.00 (1.00, 1.00) 107673 39529 26 2.3
0.57 (0.26, 1.25) 63872 18253 9 1.4
1.30 (0.60, 2.81) 27116 7293 9 3.2
1.35 (0.55, 3.30) 13217 4477 6 4.3
.3 1 1.3

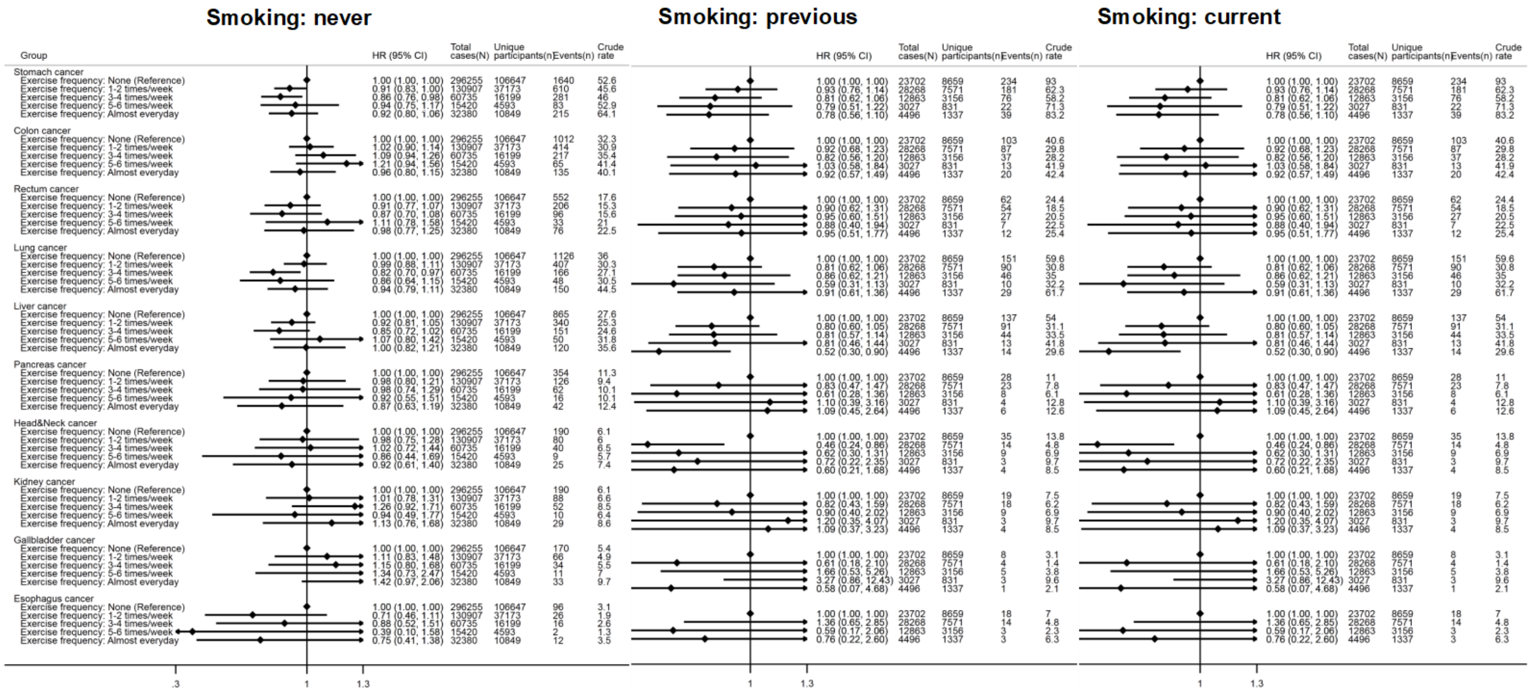
Smoking: never
Smoking: previous
Smoking: current
Group
HR (95% CI)
Total cases(N)
Unique participants(n)
Events(n)
Crude rate
Stomach cancer
Colon cancer
Rectum cancer
Lung cancer
Liver cancer
Pancreas cancer
Head&Neck cancer
Kidney cancer
Gallbladder cancer
Esophagus cancer
Exercise frequency: None (Reference)
Exercise frequency: 1-2 times/week
Exercise frequency: 3-4 times/week
Exercise frequency: 5-6 times/week
Exercise frequency: Almost everyday
.3
1
1.3

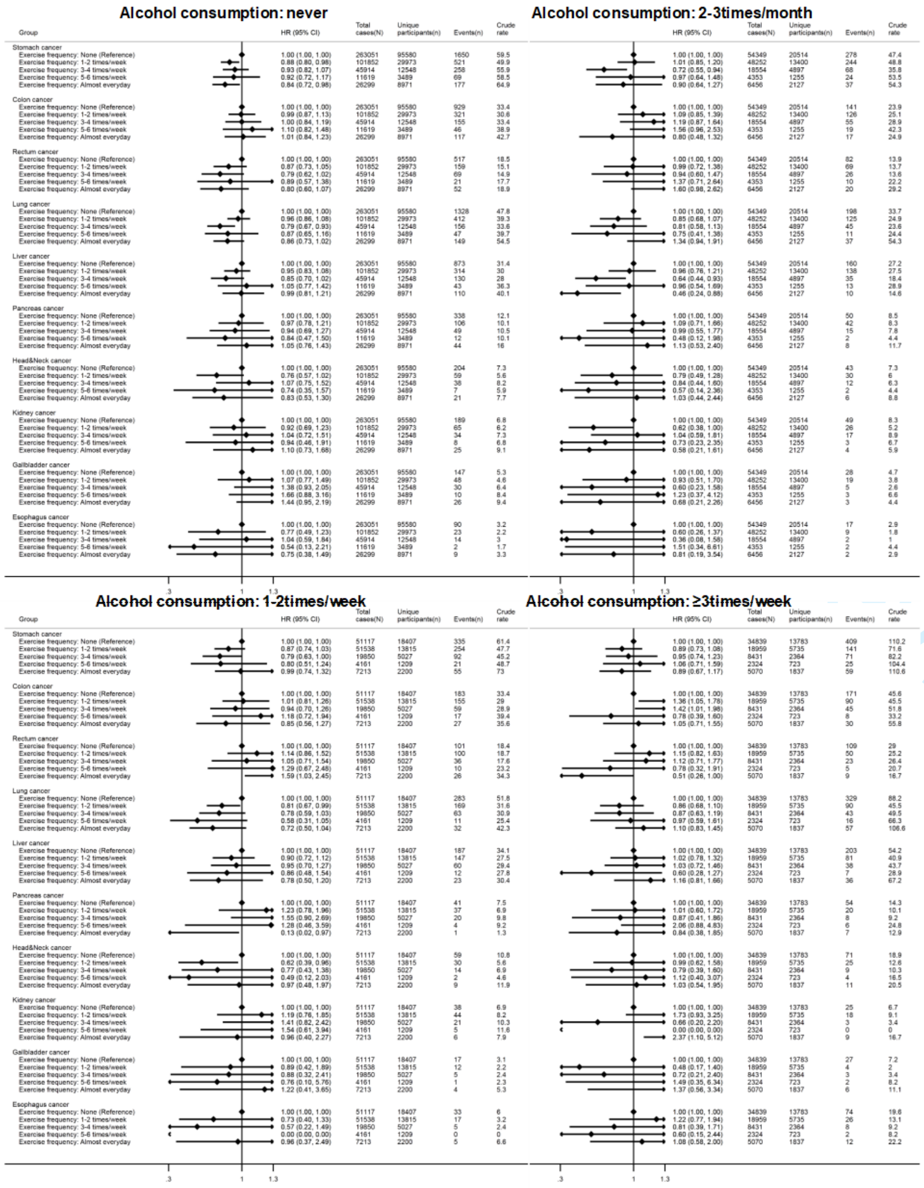

For peer review only

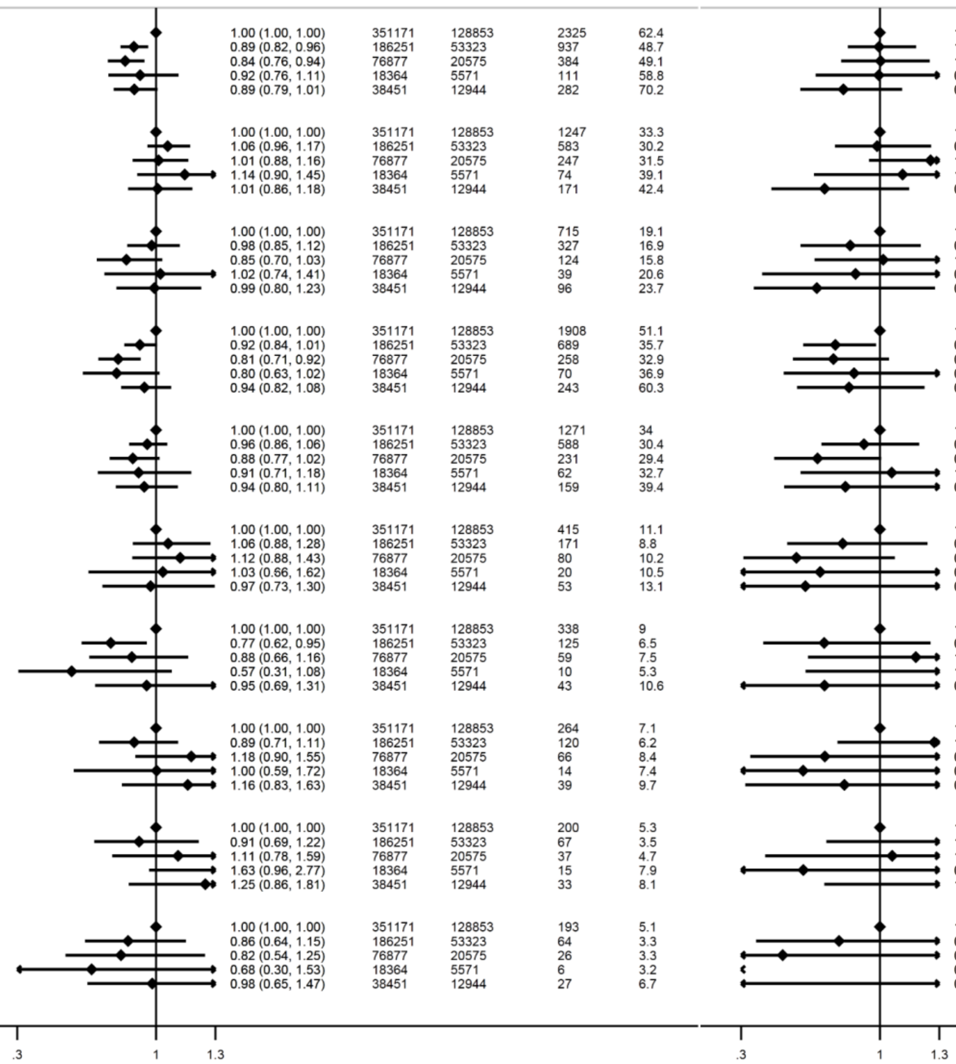

No family history of cancer

Group	HR (95% CI)	Total cases(N)	Unique participants(n)	Events(n)	Crude rate
**Stomach cancer**					
Exercise frequency: None (Reference)	1.00 (1.00, 1.00)	351171	128853	2325	62.4
Exercise frequency: 1-2 times/week	0.89 (0.82, 0.96)	186251	53323	937	48.7
Exercise frequency: 3-4 times/week	0.84 (0.76, 0.94)	76877	20575	384	49.1
Exercise frequency: 5-6 times/week	0.92 (0.76, 1.11)	18364	5571	111	58.8
Exercise frequency: Almost everyday	0.89 (0.79, 1.01)	38451	12944	282	70.2
**Colon cancer**					
Exercise frequency: None (Reference)	1.00 (1.00, 1.00)	351171	128853	1247	33.3
Exercise frequency: 1-2 times/week	1.06 (0.96, 1.17)	186251	53323	583	30.2
Exercise frequency: 3-4 times/week	1.01 (0.88, 1.16)	76877	20575	247	31.5
Exercise frequency: 5-6 times/week	1.14 (0.90, 1.45)	18364	5571	74	39.1
Exercise frequency: Almost everyday	1.01 (0.86, 1.18)	38451	12944	171	42.4
**Rectum cancer**					
Exercise frequency: None (Reference)	1.00 (1.00, 1.00)	351171	128853	715	19.1
Exercise frequency: 1-2 times/week	0.98 (0.85, 1.12)	186251	53323	327	16.9
Exercise frequency: 3-4 times/week	0.85 (0.70, 1.03)	76877	20575	124	15.8
Exercise frequency: 5-6 times/week	1.02 (0.74, 1.41)	18364	5571	39	20.6
Exercise frequency: Almost everyday	0.99 (0.80, 1.23)	38451	12944	96	23.7
**Lung cancer**					
Exercise frequency: None (Reference)	1.00 (1.00, 1.00)	351171	128853	1908	51.1
Exercise frequency: 1-2 times/week	0.92 (0.84, 1.01)	186251	53323	689	35.7
Exercise frequency: 3-4 times/week	0.81 (0.71, 0.92)	76877	20575	258	32.9
Exercise frequency: 5-6 times/week	0.80 (0.63, 1.02)	18364	5571	70	36.9
Exercise frequency: Almost everyday	0.94 (0.82, 1.08)	38451	12944	243	60.3
**Liver cancer**					
Exercise frequency: None (Reference)	1.00 (1.00, 1.00)	351171	128853	1271	34
Exercise frequency: 1-2 times/week	0.96 (0.86, 1.06)	186251	53323	588	30.4
Exercise frequency: 3-4 times/week	0.88 (0.77, 1.02)	76877	20575	231	29.4
Exercise frequency: 5-6 times/week	0.91 (0.71, 1.18)	18364	5571	62	32.7
Exercise frequency: Almost everyday	0.94 (0.80, 1.11)	38451	12944	159	39.4
**Pancreas cancer**					
Exercise frequency: None (Reference)	1.00 (1.00, 1.00)	351171	128853	415	11.1
Exercise frequency: 1-2 times/week	1.06 (0.88, 1.28)	186251	53323	171	8.8
Exercise frequency: 3-4 times/week	1.12 (0.88, 1.43)	76877	20575	80	10.2
Exercise frequency: 5-6 times/week	1.03 (0.66, 1.62)	18364	5571	20	10.5
Exercise frequency: Almost everyday	0.97 (0.73, 1.30)	38451	12944	53	13.1
**Head&Neck cancer**					
Exercise frequency: None (Reference)	1.00 (1.00, 1.00)	351171	128853	338	9
Exercise frequency: 1-2 times/week	0.77 (0.62, 0.95)	186251	53323	125	6.5
Exercise frequency: 3-4 times/week	0.88 (0.66, 1.16)	76877	20575	59	7.5
Exercise frequency: 5-6 times/week	0.57 (0.31, 1.08)	18364	5571	10	5.3
Exercise frequency: Almost everyday	0.95 (0.69, 1.31)	38451	12944	43	10.6
**Kidney cancer**					
Exercise frequency: None (Reference)	1.00 (1.00, 1.00)	351171	128853	264	7.1
Exercise frequency: 1-2 times/week	0.89 (0.71, 1.11)	186251	53323	120	6.2
Exercise frequency: 3-4 times/week	1.18 (0.90, 1.55)	76877	20575	66	8.4
Exercise frequency: 5-6 times/week	1.00 (0.59, 1.72)	18364	5571	14	7.4
Exercise frequency: Almost everyday	1.16 (0.83, 1.63)	38451	12944	39	9.7
**Gallbladder cancer**					
Exercise frequency: None (Reference)	1.00 (1.00, 1.00)	351171	128853	200	5.3
Exercise frequency: 1-2 times/week	0.91 (0.69, 1.22)	186251	53323	67	3.5
Exercise frequency: 3-4 times/week	1.11 (0.78, 1.59)	76877	20575	37	4.7
Exercise frequency: 5-6 times/week	1.63 (0.96, 2.77)	18364	5571	15	7.9
Exercise frequency: Almost everyday	1.25 (0.86, 1.81)	38451	12944	33	8.1
**Esophagus cancer**					
Exercise frequency: None (Reference)	1.00 (1.00, 1.00)	351171	128853	193	5.1
Exercise frequency: 1-2 times/week	0.86 (0.64, 1.15)	186251	53323	64	3.3
Exercise frequency: 3-4 times/week	0.82 (0.54, 1.25)	76877	20575	26	3.3
Exercise frequency: 5-6 times/week	0.68 (0.30, 1.53)	18364	5571	6	3.2
Exercise frequency: Almost everyday	0.98 (0.65, 1.47)	38451	12944	27	6.7

Family history of cancer

Group	HR (95% CI)	Total cases(N)	Unique participants(n)	Events(n)	Crude rate
**Stomach cancer**					
Exercise frequency: None (Reference)	1.00 (1.00, 1.00)	52185	19431	347	63.2
Exercise frequency: 1-2 times/week	1.00 (0.84, 1.18)	34350	9600	223	64
Exercise frequency: 3-4 times/week	1.00 (0.80, 1.25)	15872	4261	105	65.8
Exercise frequency: 5-6 times/week	0.99 (0.68, 1.46)	4093	1105	28	68.2
Exercise frequency: Almost everyday	0.82 (0.60, 1.11)	6587	2191	46	67.8
**Colon cancer**					
Exercise frequency: None (Reference)	1.00 (1.00, 1.00)	52185	19431	177	32.1
Exercise frequency: 1-2 times/week	0.99 (0.77, 1.26)	34350	9600	109	31.1
Exercise frequency: 3-4 times/week	1.26 (0.94, 1.67)	15872	4261	67	41.9
Exercise frequency: 5-6 times/week	1.11 (0.67, 1.86)	4093	1105	16	38.8
Exercise frequency: Almost everyday	0.72 (0.45, 1.15)	6587	2191	20	29.3
**Rectum cancer**					
Exercise frequency: None (Reference)	1.00 (1.00, 1.00)	52185	19431	94	17
Exercise frequency: 1-2 times/week	0.85 (0.60, 1.21)	34350	9600	51	14.5
Exercise frequency: 3-4 times/week	1.02 (0.67, 1.54)	15872	4261	30	18.7
Exercise frequency: 5-6 times/week	0.88 (0.41, 1.90)	4093	1105	7	16.9
Exercise frequency: Almost everyday	0.68 (0.36, 1.28)	6587	2191	11	16.1
**Lung cancer**					
Exercise frequency: None (Reference)	1.00 (1.00, 1.00)	52185	19431	230	41.7
Exercise frequency: 1-2 times/week	0.78 (0.61, 0.98)	34350	9600	107	30.5
Exercise frequency: 3-4 times/week	0.77 (0.56, 1.05)	15872	4261	49	30.6
Exercise frequency: 5-6 times/week	0.87 (0.51, 1.47)	4093	1105	15	36.2
Exercise frequency: Almost everyday	0.84 (0.58, 1.23)	6587	2191	32	46.9
**Liver cancer**					
Exercise frequency: None (Reference)	1.00 (1.00, 1.00)	52185	19431	152	27.5
Exercise frequency: 1-2 times/week	0.92 (0.71, 1.20)	34350	9600	92	26.2
Exercise frequency: 3-4 times/week	0.69 (0.47, 1.01)	15872	4261	32	19.9
Exercise frequency: 5-6 times/week	1.06 (0.60, 1.87)	4093	1105	13	31.4
Exercise frequency: Almost everyday	0.83 (0.52, 1.32)	6587	2191	20	29.2
**Pancreas cancer**					
Exercise frequency: None (Reference)	1.00 (1.00, 1.00)	52185	19431	68	12.3
Exercise frequency: 1-2 times/week	0.81 (0.53, 1.24)	34350	9600	34	9.7
Exercise frequency: 3-4 times/week	0.58 (0.31, 1.08)	15872	4261	12	7.5
Exercise frequency: 5-6 times/week	0.70 (0.25, 1.92)	4093	1105	4	9.6
Exercise frequency: Almost everyday	0.62 (0.29, 1.36)	6587	2191	7	10.2
**Head&Neck cancer**					
Exercise frequency: None (Reference)	1.00 (1.00, 1.00)	52185	19431	39	7.1
Exercise frequency: 1-2 times/week	0.72 (0.41, 1.26)	34350	9600	19	5.4
Exercise frequency: 3-4 times/week	1.18 (0.64, 2.19)	15872	4261	14	8.7
Exercise frequency: 5-6 times/week	1.59 (0.63, 4.06)	4093	1105	5	12.1
Exercise frequency: Almost everyday	0.72 (0.26, 2.03)	6587	2191	4	5.8
**Kidney cancer**					
Exercise frequency: None (Reference)	1.00 (1.00, 1.00)	52185	19431	37	6.7
Exercise frequency: 1-2 times/week	1.27 (0.79, 2.06)	34350	9600	33	9.4
Exercise frequency: 3-4 times/week	0.72 (0.35, 1.51)	15872	4261	9	5.6
Exercise frequency: 5-6 times/week	0.61 (0.15, 2.55)	4093	1105	2	4.8
Exercise frequency: Almost everyday	0.82 (0.32, 2.10)	6587	2191	5	7.3
**Gallbladder cancer**					
Exercise frequency: None (Reference)	1.00 (1.00, 1.00)	52185	19431	19	3.4
Exercise frequency: 1-2 times/week	1.44 (0.73, 2.84)	34350	9600	16	4.5
Exercise frequency: 3-4 times/week	1.06 (0.42, 2.68)	15872	4261	6	3.7
Exercise frequency: 5-6 times/week	0.61 (0.08, 4.60)	4093	1105	1	2.4
Exercise frequency: Almost everyday	1.82 (0.72, 4.60)	6587	2191	6	8.7
**Esophagus cancer**					
Exercise frequency: None (Reference)	1.00 (1.00, 1.00)	52185	19431	21	3.8
Exercise frequency: 1-2 times/week	0.79 (0.38, 1.68)	34350	9600	11	3.1
Exercise frequency: 3-4 times/week	0.51 (0.15, 1.74)	15872	4261	3	1.9
Exercise frequency: 5-6 times/week	0.00 (0.00, 0.00)	4093	1105	0	0
Exercise frequency: Almost everyday	0.27 (0.04, 2.03)	6587	2191	1	1.5

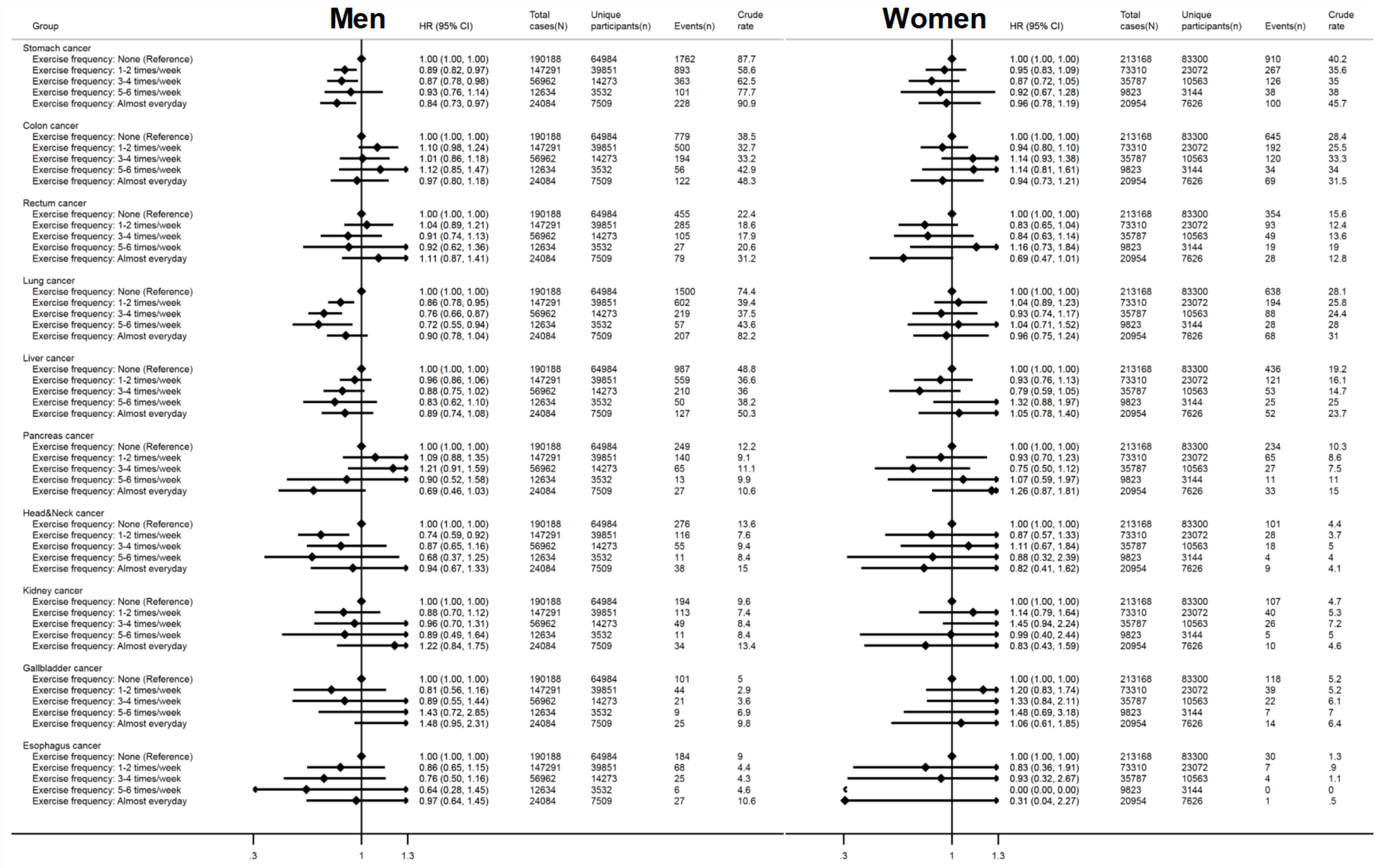

Supplementary Figure 5. Results from running Cox regression models examining effect modification of sex, body mass index, smoking, alcohol consumption, and family history of disease in the associations between exercise frequency and various incident cancer outcomes. Note: Cox regression models using age as the underlying timescale were adjusted for sex [not in models for effect

modification by sex], body mass index [not in models for effect modification by body mass index], systolic blood pressure, fasting glucose levels, total cholesterol levels, family history of cancer [not in models for effect modification by family history of cancer], smoking status [not in models for effect modification by smoking status] and alcohol consumption [not in models for effect modification by alcohol consumption]. Crude rates are per 100,000 person-years. “N” indicates numbers of total observations (i.e. participants who provided repeated measures are treated as separate observations) and “n” indicates numbers of unique participants at baseline. P-values for multiplicative terms – stomach cancer (p-value = 0.274), colon cancer (p-value = 0.640), rectum cancer (p-value = 0.107), lung cancer (p-value = 0.016), liver cancer (p-value = 0.129), pancreas cancer (p-value = 0.860), head & neck cancer (p-value = 0.488), kidney cancer (p-value = 0.285), gallbladder cancer (p-value = 0.970), and esophagus cancer (p-value = 0.263) by body mass index; stomach cancer (p-value = 0.699), colon cancer (p-value = 0.932), rectum cancer (p-value = 0.610), lung cancer (p-value = 0.492), liver cancer (p-value = 0.405), pancreas cancer (p-value = 0.338), head & neck cancer (p-value = 0.562), kidney cancer (p-value = 0.280), gallbladder cancer (p-value = 0.295), and esophagus cancer (p-value = 0.292) by smoking ; stomach cancer (p-value = 0.655), colon cancer (p-value = 0.977), rectum cancer (p-value = 0.433), lung cancer (p-value = 0.387), liver cancer (p-value = 0.704), pancreas cancer (p-value = 0.711), head & neck cancer (p-value = 1.000), kidney cancer (p-value = 0.336), gallbladder cancer (p-value = 0.350), and esophagus cancer (p-value = 0.550) by alcohol consumption ; stomach cancer (p-value = 0.996), colon cancer (p-value = 0.399), rectum cancer (p-value = 0.478), lung cancer (p-value = 0.427), liver cancer (p-value = 0.337), pancreas cancer (p-value = 0.086), head & neck cancer (p-value = 0.712), kidney cancer (p-value = 0.319), gallbladder cancer (p-value = 0.766), and esophagus cancer (p-value = 0.098) by family history of cancer; and stomach cancer (p-value = 0.405), colon cancer (p-value = 0.957), rectum cancer (p-value = 0.106), lung cancer (p-value = 0.063), liver cancer (p-value = 0.278), pancreas cancer (p-value = 0.265), head & neck cancer (p-value = 0.907), kidney cancer (p-value = 0.967), gallbladder cancer (p-value = 0.548), and esophagus cancer (p-value = 0.256) by sex. Abbreviations: HR – Hazard Ratio; CI – Confidence Intervals

For peer review only
